# Association and Linkage Analysis of Aluminum Tolerance Genes in Maize

**DOI:** 10.1371/journal.pone.0009958

**Published:** 2010-04-01

**Authors:** Allison M. Krill, Matias Kirst, Leon V. Kochian, Edward S. Buckler, Owen A. Hoekenga

**Affiliations:** 1 United States Department of Agriculture-Agricultural Research Service, Robert W. Holley Center for Agriculture and Health, Ithaca, New York, United States of America; 2 Cornell University, Department of Plant Breeding and Genetics, Ithaca, New York, United States of America; 3 Institute for Genomic Diversity, Cornell University, Ithaca, New York, United States of America; 4 University of Florida, School of Forest Resources and Conservation, Gainesville, Florida, United States of America; 5 Cornell University, Department of Plant Biology, Ithaca, New York, United States of America; East Carolina University, United States of America

## Abstract

**Background:**

Aluminum (Al) toxicity is a major worldwide constraint to crop productivity on acidic soils. Al becomes soluble at low pH, inhibiting root growth and severely reducing yields. Maize is an important staple food and commodity crop in acidic soil regions, especially in South America and Africa where these soils are very common. Al exclusion and intracellular tolerance have been suggested as two important mechanisms for Al tolerance in maize, but little is known about the underlying genetics.

**Methodology:**

An association panel of 282 diverse maize inbred lines and three F_2_ linkage populations with approximately 200 individuals each were used to study genetic variation in this complex trait. Al tolerance was measured as net root growth in nutrient solution under Al stress, which exhibited a wide range of variation between lines. Comparative and physiological genomics-based approaches were used to select 21 candidate genes for evaluation by association analysis.

**Conclusions:**

Six candidate genes had significant results from association analysis, but only four were confirmed by linkage analysis as putatively contributing to Al tolerance: *Zea mays Alt_SB_ like* (*ZmASL*), *Zea mays aluminum-activated malate transporter2* (*ALMT2*), *S-adenosyl-L-homocysteinase (SAHH)*, *and Malic Enzyme* (*ME*). These four candidate genes are high priority subjects for follow-up biochemical and physiological studies on the mechanisms of Al tolerance in maize. Immediately, elite haplotype-specific molecular markers can be developed for these four genes and used for efficient marker-assisted selection of superior alleles in Al tolerance maize breeding programs.

## Introduction

Aluminum (Al) toxicity from acidic soil is a major constraint to worldwide crop production. Al, one of the most abundant elements in the soil, is solubilized as Al^3+^ under acidic soil conditions. This form of Al is highly toxic to plant roots. Approximately 30% of the worlds ice-free soils are acidic, 17% of which are considered arable [1]. Maize has become one of the most important grain crops grown on acidic soils due to its demand as a food crop and its ability to tolerate Al [1]. Up to a 70% reduction in maize yields have been seen in these regions due to Al toxicity [1]–[3]. Acid precipitation and intensive agricultural practices such as overuse of ammonia fertilizers accelerate the natural process of soil acidification, especially in the tropical and subtropical regions [4]. Soil amelioration with compounds such as lime can be used to temporarily neutralize the topsoil. However, this is not a feasible option for resource poor farmers or for subsoil acidity, and is not an economically or agronomically sustainable solution. Investing in the production of Al tolerant maize varieties and alternative management practices can contribute greatly to increased yield and sustainable crop production from acidic soils [5], [6]. Therefore, an understanding of the genetic and molecular mechanisms underlying Al tolerance in maize is essential to accelerate the development of Al tolerant varieties.

The toxic effects of acid soil result from an interaction between pH and elements in the soil. Several metals, including Al and Mn, become soluble at and below pH 5.5, which causes stress in the plant. In a neutral or basic environment, Al is found in insoluble divalent and monovalent forms of Al-oxides or Al-hydroxides, but the soluble trivalent Al^3+^ ion becomes the dominant species in an acidic environment [4]. Al^3+^ disrupts many physiological processes in plants through both apoplastic and symplastic interactions, but exact mechanisms remain elusive [7], [8]. The root apex is the most sensitive part of the plant to Al because it is the site of cell division and expansion for the root [9], [10]. Al-induced inhibition of root growth is the primary symptom of Al toxicity [7], [9]. Reduction in root growth and function leads to increased susceptibility to other stresses, primarily drought and mineral deficiencies, due to the limited capacity of Al-intoxicated roots to acquire sufficient water and nutrition from the soil. There have been numerous mechanisms proposed for Al toxicity, but it is likely from the disruption of a number of different processes. One important site of Al^3+^ intoxication is the cell wall of the root apex [11]. In response to Al^3+^ exposure, callose formation seals off the cell walls, increasing rigidity, decreasing extensibility and preventing further transport into the cell [12]. Al^3+^ displaces Mg^2+^ and Ca^2+^, which are required for ATPases, cell signaling, and altering or inactivating the function of many proteins [9], [13]. Other possible mechanisms of Al toxicity include interference with the cytoskeleton, promotion of lipid peroxidation and blocking of Ca^2+^ channels [14], [15]. Specifically for maize grown in the field, Al intoxication causes several stress related physiological effects, including stunting, reduced number of ears per plant, delayed flowering, and reduced biomass and total yield [3], [5].

Plants have developed several mechanisms for dealing with Al toxicity, which can be classified as either external or internal tolerance mechanisms [13]. External mechanisms include differential binding of Al to the cell wall, selective permeability of the plasma membrane, formation of a plant induced pH barrier in the rhizosphere, and root exudation of chelating compounds, such as organic acids (OA) or phenolic compounds. Internal mechanisms include chelation of Al in the cytosol, compartmentalization in the vacuole, Al-binding proteins, Al tolerant enzyme isoforms, and elevated enzyme activity [4], [13]. Most Al tolerance research has focused on Al induced root exudation of OA to chelate Al in the rhizosphere, where non-toxic complexes can be formed between Al and an OA such as citrate. Root exudation of OAs is a widespread response to Al in both monocots and dicots [16], [17]. This mechanism has been shown to play a role in Al tolerance in several species though the activation of anion transporters in the plasma membrane [16]–[20].

Maize has considerable genetic variation in levels of Al tolerance, but clear physiological bases and molecular mechanisms for this tolerance remain elusive. Physiological studies found that OA exudation contributes to maize Al tolerance, but is not the only mechanism, as some Al sensitive varieties have been shown to exude high amounts of OA from the roots [21]. Differences in cell wall pectin content and degree of methylation have also been suggested to contribute to Al tolerance in maize [22]. Understanding the mechanisms of Al tolerance can accelerate the efforts to identify and incorporate superior genes and alleles into maize breeding programs. Recurrent selection has been used to develop Al tolerant maize populations with yields as much as 200% greater than susceptible lines [3], [23]. However, a strong genetic by environment (GxE) interaction and relatively low heritability of Al tolerance in maize complicates selection and has made substantial progress difficult [3], [5], [24].

The genetic variation for Al tolerance in maize indicates it is a complex trait, involving many genes and physiological processes [3], [24], [25]. Several QTL studies examined Al tolerance in maize, and suggest that about 6 loci account for ∼60% of the variation in tolerance levels [21], [26]–[28]. However, QTL in these different biparental populations are not shared, suggesting genetic heterogeneity [29]. This is not unreasonable, as the first two populations were constructed from South American maize varieties and the latter from North American lines. Transgressive segregation is seen in these three biparental mapping populations indicative of additive and/or interaction effects among alleles contributed by the two parents. Al stress was likely a powerful selective force during maize domestication and early improvement, as maize exhibits regional adaption to various levels of Al toxicity [4].

Biparental crosses used in linkage mapping, in which one or a few loci controlling Al tolerance may segregate, provide limited insight into the analysis of complex traits in general [29]. Linkage mapping has strong statistical power and is useful for understanding how and to what extent allelic effects are dependent on one another, but provides low genetic resolution unless the population is very large [30]. Alternatively, association mapping is a method for high-resolution mapping of QTL based on linkage disequilibrium (LD), and is useful for dissecting complex traits controlled by multiple QTL in species where LD decays rapidly [29], [30]. Unlike linkage mapping, where only two alleles are evaluated, association mapping evaluates a greater number of alleles in a broader population. Linkage mapping uses shared inheritance of polymorphism and linked markers within families of known ancestry. Association mapping takes advantage of the historic recombination of several hundred lines, to identify common genes contributing to the trait of interest. The LD structure of the gene is essential in association mapping. This approach allows evaluation of genes from smaller sampled regions, within the range of LD decay, instead of requiring complete candidate gene sequencing. This method requires three data types: phenotypic trait information, genotypic data from or near the gene of interest and an understanding of population structure within the test panel. Beyond the requirement for prior molecular knowledge, the other principal disadvantage of association mapping is that spurious marker trait associations can arise from population structure. However, we can identify many of these false positive results via a mixed linear model (MLM) approach, which takes population structure and varietal relatedness into account [31]. The combination of association mapping and linkage mapping can provide both the power and resolution needed for detecting QTL of interest.

In this study, we used an integrated approach combining association mapping with linkage mapping to identify and evaluate candidate Al tolerance genes in maize. Without positively identified mechanisms or biochemical pathways involved in Al tolerance, selection of candidate genes requires knowledge based on previous studies and proposed mechanisms. We tested 21 candidate genes for association with Al tolerance, in a maize diversity panel of 282 inbred lines, using the MLM approach discussed earlier [31]–[34]. Candidate genes were screened in a subset of 27 diverse lines (DL), selected to be representative of the genetic and phenotypic diversity in the association panel, in order to identify highly polymorphic regions for further association studies. Due to strong GxE effects in field studies of Al tolerance, selection or testing of tolerance in pots of acid soil or hydroponics solutions is a quick and efficient way to determine tolerant and sensitive lines in maize while controlling for environmental effects [35]. Al tolerance levels were measured, as net root growth (NRG) in nutrient solution containing a toxic level of Al [36]. Several genes were found to be associated with NRG under Al stress and subsequently confirmed using linkage analysis.

## Results

### Phenotypic data

**Figure 1:**
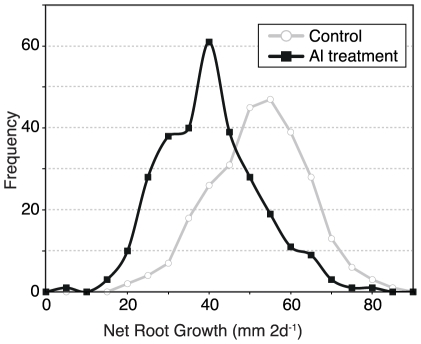
Distribution of Net Root Growth (NRG) in the Maize Association Panel. Bins reflect grouping the inbred lines by 5 mm 2 d^−1^ increments of root growth under both Al stress (circles) and control (squares) treatments. Reported values are Least Squares Means calculated from five replicate experiments for Al stress treatment or three replicate experiments for control treatment.

Phenotypic data for Al tolerance in the maize association panel was collected as net root growth (NRG) in a hydroponic nutrient solution with or without a toxic level of Al^3+^ [37]. Al stress measurements were taken before and after 2 days of stress in a hydroponic solution containing {27 µM Al^3+^} at pH 4.0. A control treatment was carried out over the same time period in an identical hydroponic solution, containing no Al^3+^. A wide range of tolerance levels is seen in this panel for both control NRG and Al treated NRG (Figure 1 and Table S1). Mean NRG under control treatment and under Al stress was 50.04±11.58 mm and 37.79±11.91 mm, respectively. Differences between the two groups were highly significant (p = 1.5×10^−27^). Mean correlation between replications was 42.5% in Al stress treatments and 37.3% in control treatment replications.

Narrow sense heritability (h^2^) for NRG in the Diversity Panel, calculated using the relatedness (K) matrix, was between 30 and 32% in the Al stress environment and 22% without Al stress. Broad sense heritability (H^2^) for NRG under Al stress and without Al stress was 41% and 37%, respectively. These heritability estimates for net root growth in seedlings are similar to those observed in maize breeding programs for enhanced tolerance to low pH [5].

### Genotypic data

Genotypic data for association mapping came from polymorphisms identified in candidate gene sequences. Genes were chosen based one of two factors: as responsive to Al-stress treatments according to gene expression analysis or by sequence similarity to Al tolerance genes found in other species (Table 1). Genes throughout the remainder of this study are referred to by the Gene ID listed in Table 1. Thirteen candidate genes were identified as differentially responsive to Al stress treatments in root tips, from Al tolerant and Al sensitive maize lines, in previous studies [38]. Eight candidate genes were chosen by comparative genomics based on their contribution to Al tolerance in related grass species. *TaALMT1* (*Aluminum activated malate transporter*) is the major Al tolerance gene in wheat (*Triticum aestivum*) and is the first true Al tolerance gene identified in any plant [39]. Seven maize genes homologous to *TaALMT1* were examined and are referred to as *ZmALMTx*. One gene homologous to *Alt_SB_*_,_ the major Al tolerance gene in *Sorghum bicolor*, is referred to as *ZmASL* (*Zea mays Alt_S__B_ – like*) [20]. The selection of genes using a comparative genomics approach is based on evidence suggesting many agronomically important traits, such as Al tolerance, may be controlled by orthologous loci in related grasses or more distant species [40]. For example, genes related to *TaALMT1* from wheat have been demonstrated as Al tolerance genes in Arabidopsis and rye [18], [39], [41], [42], while genes related to *Alt_SB_* from sorghum have been demonstrated as Al tolerance genes in Arabidopsis and barley [20], [40], [42], [43].

**Table 1:** Candidate Al tolerance genes evaluated by association mapping.

Gene ID	Gene Name	MAGI ref seq#	Length (bp)	Lines (#)	Sites (#)	Chr (#)	ctg (#)	BIN (#)
ME	Malic Enzyme	3.1_47445	626	255	12	6	282	6.05
FE	Iron-responsive transporter-like	3.1_61976	503	240	11	2	106	2.08
ANTI	Major facilitator superfamily antiporter	3.1_69188	549	246	14	6	285	6.05
ABC	ABC transporter-like protein	3.1_80359	373	251	21	2	106	2.08
ISL	Isocitrate Lyase	3.1_108586	526	228	6	7	322	7.03
AUX1	Amino acid permease AUX1	3.1_112316	526	206	23	2	106	2.08
SAHH	SAH hydrolase	4.0_116767	500	206	6	4	160	4.03
P450	Cytochrome P450	4.0_145633	786	246	14	3	138	3.06
PME	Pectin methylesterase	4.0_158804	454	270	3	1	49	1.08
PI3K	Phosphatidylinositol 3-kinase	4.0_112182	590	254	10	4	172	4.05
OO2	Germin2 (oxalate oxidase)	4.0_67335	729	182	26	10	399	10.03
IDH	Isocitrate dehydrogenase	4.0_48631	1061	253	14	4	173	4.05
FUM	Fumerase	4.0_35824	646	269	8	1	14	1.04
AL1	ZmALMT1	3.1_92675	476	182	31	10	412	10.04
AL2	ZmALMT2	3.1_93496	487	199	17	10	412	10.04
AL3	ZmALMT3	3.1_92049	633	193	9	5	252	5.07
AL5	ZmALMT5	3.1_811363	743	215	24	10	412	10.044
AL8	ZmALMT8	3.1_90876	504	208	26	5	247	5.06
AL9	ZmALMT9	3.1_6591	288	263	2	5	214	5.03
AL16	ZmALMT16	3.1_36360	HAP*	285	1	10	415	10.06
ASL	ZmASL (region 1)	3.1_41691	1179	278	32	1	9	1.02
ASL	ZmASL (region 2)	3.1_41691	715	240	21	1	9	1.02

Thirteen genes were selected from gene expression analysis, while another eight came from comparative genomics. Genes were identified from genome survey sequence contigs created by the MAGI Project. MAGI build version and reference number are reported. “Length” describes the total length of sequence used for polymorphic site identification. “# Lines” refers to the number of entries with sufficient information to include in the association analysis. “# Sites” refers to polymorphisms that occurred at greater than 10% frequency. Physical-genetic map locations for each candidate gene are reported according to chromosome, genomic sequencing contig and genetic map bin. Genetic bins that appear in bold represent those under previously reported Al tolerance QTL. Gene AL16 was evaluated by a large indel (*HAP) rather than by gene sequence. Gene ASL underwent two rounds of sequence analysis.

Information regarding the genes used in association mapping is shown in Table 1. A region of high polymorphism in each gene (based on preliminary sequencing in the 27 DL) was sequenced in the association panel. Polymorphisms with frequency ≥10% were extracted from the sequences for analysis, giving a total of 331 sites across all genes and an average of 15 sites per gene (Table S2). Reference sequence, length of sequence, number of lines with sufficient quality sequence and the physical map location of each gene are shown [44], [45]. Given the heterogeneity in the rates of LD decay for these genes, size of the genes, and the possibility for distant regulatory elements, these polymorphism surveys are not intended to be comprehensive surveys of polymorphism. Instead, the sequencing results presented here are a representative sample that enables us to efficiently screen a large number of loci and identify markers with strong associations to Al stress tolerance.

### Association mapping

The mixed linear model (MLM) was used for association mapping [31]. The MLM accounts for multiple levels of relatedness, defined as population structure (Q) and a pairwise kinship matrix (K), to control for both Type I and Type II errors [31]. A General Linear Model (GLM) including Q was also tested. Both models, GLM and MLM, were applied to NRG under Al stress and NRG under no Al stress. NRG under no Al stress was also used as a fixed effect covariate in the MLM model, Q+K+C (Table 2 & Table S3). This model was used to evaluate relative root growth, which is frequently used as a measurement of Al tolerance.

**Table 2:** Evaluation of association mapping results by ANOVA.

Candidate Genes	# Sites	GLM (Q)	MLM (QK)	MLM (QKC)	Max r^2^ (Model)
ME	12	0	0	3	1.3% (QKC)
ISL	6	0	0	0	1.2% (QKC)
SAHH	6	0	0	2	2.1% (QKC)
AL2	17	1	2	0	2.7% (QK)
ASL	53	26	10	5	2.0% (QK)
PME	3	0	1	1	1.6% (QK)
FDR p<0.01		40%	24%	34%	
h^2^		n/a	0.30	0.32	
H^2^			0.41		

GLM and MLM analyses were used to evaluate the 21 candidate Al tolerance genes, using the net root growth trait collected from Al treated plants. These models incorporated the population structure (Q) of the Diversity Panel, the relative kinship (K) of the Diversity Panel and net root growth of the Diversity Panel grown without Al stress as a fixed effect covariate (C). The GLM model used only factor Q, while the MLM models used factors Q+K and Q+K+C. Six candidate genes gave significant results and are shown, with the number of significant sites (p<0.01) identified per locus for each model. The maximum value for variance explained by a marker within a gene in any model is reported. False Discovery Rates were empirically calculated for each model based on 1,095 random SNPs throughout the genome and are expressed as percentages. Narrow (h^2^) and broad sense (H^2^) heritability estimates were generated for each trait based upon variance estimates from the MLM.

Six genes had statistically significant associations (p≤0.01) with NRG under Al stress and were selected for further study in F_2_ linkage populations: *malic enzyme (ME)*; *isocitrate lyase (ISL)*; *SAH hydrolase (SAHH)*; *ZmALMT2 (ALMT2)*; *ZmASL (ASL)*; *pectin methylesterase (PME)* (Table 2). Complete results from the MLM analysis can be found in Table S3. *ISL* was statistically significant at a less stringent value (p<0.05) for Al stress. In order to estimate the number of expected false positives due to multiple testing of sites, a false discovery rate (FDR) was calculated for each model using 1,095 random SNPs throughout the genome. FDR allows for the comparison of significant sites in our candidate genes to those we would expect to see by random chance alone. Based on the FDR values for the MLMs, about 24% of the sites under the Q+K model and 34% under the Q+K+C model under Al stress could be accounted for by false positives. Given this high rate for false discovery, it is crucial to test the connection between the six genes with putative association to Al stress tolerance using an independent line of reasoning.

### Linkage mapping

**Figure 2:**
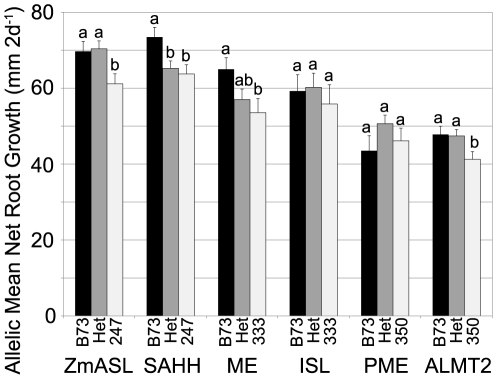
Linkage mapping validation of candidate Al tolerance genes. Six candidate Al tolerance genes were evaluated using three F_2_ linkage populations: B73×CML247, B73×CML333, and B73×NC350. Mean Net Root Growth values for each allelic state are reported, abbreviated as B73 for the B73 homozygous class, Het for the heterozygous class, and the numerical portion of the non-B73 parent name for the other homozygous class; error bars reflect standard error. Student's t-test was used to evaluate differences between allelic classes within each F_2_; differences significant at p<0.05 are indicated with letter codes.

If the association analysis truly identified Al tolerance genes, then the associated SNPs should explain significant variance for Al tolerance in segregating populations. Linkage mapping could therefore be used to test the results of association mapping. Linkage to Al tolerance was tested for the six genes listed in Table 2 using three F_2_ populations. F_2_ populations were phenotyped in the same manner as the association panel and genotyped for the sites of interest (Table S4). These F_2_ populations were constructed so that each would segregate for polymorphisms associated with two putative Al tolerance loci: *ZmASL* and *SAHH* within B73×CML247; *ME* and *ISL* within B73×CML333; *PME* and *ZmALMT2* within B73×NC350 (Figure 2). A comparison of means for each allelic class suggested that the polymorphisms tested at *ZmASL*, *SAHH*, *ME*, and *ZmALMT2* were significantly associated with Al tolerance (Figure 2). However, allelic means for *ISL* and *PME* were equivalent no matter the state, suggesting that the polymorphisms tested were not associated with Al tolerance. Linkage was tested by GLM for the 4 putative Al tolerance genes, assuming complete dominance (*ZmASL*, *SAHH* and *ZmALMT2*) or additive gene action (*ME*; Table 3). These results indicate that small effect (3–6% variance explained) QTL exist for Al tolerance at these four loci. No significant interactions between Al tolerance genes were found, suggesting that epistasis is not at work. The identification of *ISL* and *PME* as Al tolerance gene based on association mapping were likely false positives, as there was no linkage to Al tolerance differences with the polymorphisms tested in F_2_ populations, and is consistent with our expectations based on the FDR calculation.

**Table 3:** Evaluation of linkage mapping results by ANOVA.

Factor	DF	SS	F	P
ALMT2 (dom)	1	1241.43	6.73	0.0106
Error	125	19482.91		
Model Total	126	20531.35	Adjusted r^2^ =	0.043

Factor	DF	SS	F	P
ME (add)	2	2721.62	3.29	0.0403
Error	127	52484.35		
Model Total	129	55205.97	Adjusted r^2^ =	0.034

Factor	DF	SS	F	P
SAHH (dom)	1	1903.55	8.58	0.0040
Error	129	28631.73		
Model Total	130	30535.28	Adjusted r^2^ =	0.055

Factor	DF	SS	F	P
ZmASL (dom)	1	2037.51	8.44	0.0044
Error	124	29951.96		
Model Total	125	31989.47	Adjusted r^2^ =	0.056

Factor	DF	SS	F	P
SAHH (dom)	1	1445.72	6.94	0.0097
ZmASL (dom)	1	1586.91	7.62	0.0068
Error	106	22085.66		
Model Total	108	25684.81	8.57	0.0004
			Adjusted r^2^ =	0.123

GLM analysis was used to evaluate whether SNP markers within candidate Al tolerance genes explained significance variance for Al tolerance observed in F_2_ populations. Gene action was modeled as either additive (“add”) or dominant (“dom”) based on allelic means. The variance explained by each significant SNP is reported. As both *SAHH* and *ZmASL* were significantly associated with Al tolerance for the B73×CML247 population, a summary model is reported. DF: Degrees of Freedom; SS: Sum of Squares; F: F ratio; P: P value.

The four genes with significant association and linkage to Al tolerance, *Zea mays Alt_SB_-like* (*ZmASL*), *S-adenosyl-L-homocysteinase* (*SAHH*), *Malic Enzyme* (*ME*), and *ZmALMT2* (*ALMT2*), are described in further detail below. All genes possessed more than one statistically significant polymorphism associated with Al tolerance differences. The complete coding sequences for the *ZmASL*, *SAHH* and *ME* genes were characterized in the 27 DL to look for other regions of interest such as non synonymous sites, alternative splicing, and protein structure modifications. *ALMT2* was not sequenced in the 27 DL subset due to constraints caused by abundant paralogs within the *ZmALMT* family. Individual sites in these genes explain only about 2% of the phenotypic variance in the association panel, but confer 13%–20% increase in NRG.

### Al tolerance gene: *ZmASL*

**Figure 3:**
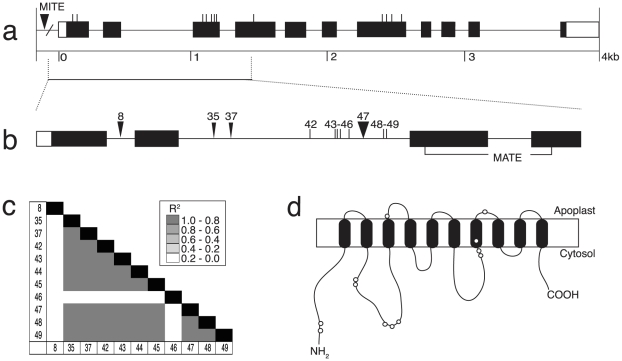
Characterization of *ZmASL*. (**a**) Predicted gene structure for the *ZmASL* locus is shown, with exons as black boxes, introns as thin lines, and UTRs as open boxes. The approximate location of 12 amino acid substitutions or additions that occur at greater than 10% frequency among alleles are shown with vertical lines above the exons, based on complete *ZmASL* sequencing performed in the 27 DL subset. (**b**) A focus region of *ZmASL* was sequenced in the association panel. The polymorphisms that were identified as significant by the association analyses are shown – SNPs as vertical lines, indels as triangles – and are referred to by number. The conserved MATE domain is highlighted in exons 3 and 4. (**c**) Linkage disequilibrium plot for the eleven significant polymorphisms. High linkage disequilibrium exists between nine of the eleven associated polymorphisms. (**d**) An estimate for the transmembrane structure of *ZmASL*, where open circles indicate the approximate locations for the 12 amino acid substitution/insertions detected within the gene.

*ZmASL*, which is highly similar to the Al-activated citrate transporter from sorghum, is described in Figure 3. Figure 3A shows the gene organization for *ZmASL*, including exons, introns and nonsynonymous sites, based on the genomic sequence of the 27 DL. Total length of *ZmASL* sequenced in the 27 DL was about 6 kb, including 11 exons, both 5′ and 3′ UTRs and an upstream region containing a 300 bp MITE insertion. The common polymorphisms (frequencies ≥10%), which are responsible for 12 amino acid substitutions, are shown. The 43 rare amino acid substitutions, insertions or deletions (<10% frequency) are not shown. Many of the rare polymorphisms are found in only one of the 27 DL (CML247 was responsible for 21 sites).

Two minimally overlapping regions of this gene were sequenced in the association panel, covering a total of ∼1.7 kb (Figure 3B). These regions represent the first three exons and part of the fourth, and were selected as they were highly polymorphic for both synonymous and nonsynonymous sites, including 7 of the 12 common amino acid substitutions. Based on this sequence the remaining 5 amino acid substitutions were inferred from haplotype structure. The MITE insertion in the 5′ UTR was also scored in the panel. Altered gene expression in the *Alt_SB_* gene is associated with the number of MITE insertions in the regulatory region of that gene [20]. However, the MITE found in *ZmASL* was not associated with NRG.

We detected 11 sites that were significantly associated with NRG under Al stress in the MLM models, as shown in Figure 3B. All of the significant sites occur in introns. A 120 bp indel (site #47) in the second intron showed the highest statistical significance and was in high LD with several of the other significant sites. A total of three independent sites (R^2^<0.2) were significantly associated with NRG in the region sequenced (Figure 3C). Each significant site in the Al stress statistical models explains between 1.5 and 2.7% of the total phenotypic variance observed in the association panel. However, the most significant site has an effect estimate that increases NRG 16% over the two days of Al stress. The 120 bp indel (site #47) was used for the linkage analysis in the B73×CML247 F_2_ population, where it was correlated with a 15% increase in NRG. The superior allele found in B73 appeared to be fully dominant to the inferior allele found in CML247 (Figure 2).

Figure 3D shows the predicted transmembrane protein structure of this gene containing 10 putative transmembrane domains [46]. The approximate locations of the 12 common amino acid substitutions on the protein are shown. However, none of these polymorphic sites were significantly associated with NRG under Al stress.

### Al tolerance gene: *SAHH*

**Figure 4:**
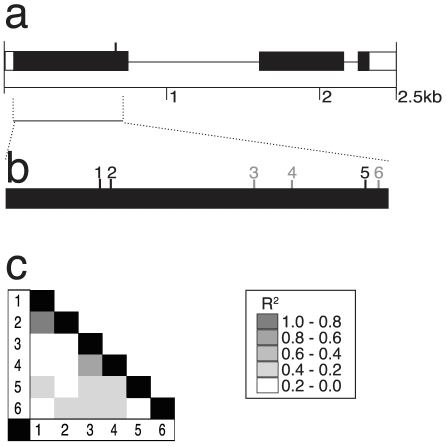
Characterization of *SAHH*. (**a**) Predicted gene structure for the *SAHH* locus is shown, with exons as black boxes, introns as thin lines, and UTRs as open boxes. A single amino acid substitution was detected from complete gene sequencing in the 27 DL subset and is indicated by the vertical line in the first exon. (**b**) A focus region of *SAHH* was sequenced in the association panel. Six SNPs were detected in the association panel and are referred to by number. Polymorphisms 1, 2 and 5 were identified as significantly associated with aluminum tolerance differences and are shown in black; non-significant sites are shown in gray. Site #5 corresponds to the triallelic SNP that causes the single amino acid substitution detected. (**c**) Linkage disequilibrium plot for all polymorphisms detected in the focus region at *SAHH*. High linkage disequilibrium exists between sites 1 and 2, while relatively low linkage disequilibrium exists through the rest of the gene.

The complete predicted coding sequence for *SAHH* was sequenced in the 27 DL. This 2.5 kb region includes three exons and the 3′ UTR (Figure 4A). Only one amino acid substitution was observed in the 27 DL gene sequences and is encoded by a triallelic SNP (#5). The region sequenced in the association panel spanned most of the first exon, including this amino acid substitution (Figure 4B). We observed 6 SNPs and no indels in this portion of the first exon. Two nonsynonymous SNPs (#1 and #2), in high LD (R^2^≥0.8), were significant for NRG under the Q+K+C model (Figures 4B & 4C). The triallelic SNP (#5) was significant at the p<0.05 level, and leads to either a synonymous (Glu for Glu) or conservative (Asp for Glu) amino acid substitution. The triallelic SNP was in moderate LD with associated SNP #1 and in little or no LD with SNP #2. The two highly significant sites (#1/#2 and #5) explain between 1.8 and 2.1% of the phenotypic variation and confer up to a 13% increase in NRG under Al stress.

Instead of utilizing one of the associated SNPs, an indel polymorphism identified in the first intron during whole gene sequencing was used for the linkage analysis. The choice of the indel provided us a simple PCR based assay for genotyping and took advantage of the difference in genetic resolution between association mapping and linkage mapping. Far fewer recombination events were captured in the F_2_ population than in the association panel, thus an indel that was not scored in the complete association panel was equally useful for linkage analysis. This indel was correlated with a 13% increase in NRG in the B73×CML247 F_2_ population (Figure 2), the same relative increase as we attributed to *SAHH* by association mapping. The inferior allele of *SAHH* found in CML247 was fully dominant to the superior allele from B73 (Figure 2).

### Al tolerance gene: *ME*

**Figure 5:**
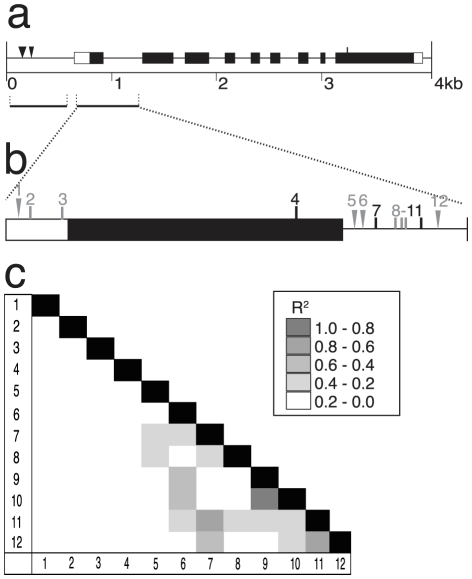
Characterization of *ME*. (**a**) Predicted gene structure for the *ME* locus is shown, with exons as black boxes, introns as thin lines, and UTRs as open boxes. Two indels and an SNP were detected in sequencing the complete gene in 27 DL subset. (**b**) A focus region of *ME* was sequenced in the association panel. Four indels and eight SNPs were detected; three SNPs were significantly associated with aluminum tolerance and are shown in black. (**c**) Linkage disequilibrium plot for all polymorphisms detected within the focus region at *ME*. No linkage disequilibrium exists within the 5′ end of the focus region, while moderate linkage disequilibrium exists among several of the 3′ end sites.

The complete predicted gene sequence for *ME*, including both 5′ and 3′ UTRs and a farther 5′ region with two large insertions was sequenced in the 27 DL (approximately 5 kb; Figure 5A). Like *SAHH*, we saw very little nucleotide diversity in the *ME* sequences – only one amino acid substitution was seen in more than one line, located in the last exon. Three rare amino acid substitutions were seen in one line. The 5′ UTR, the first exon, and most of the first intron were sequenced in the association panel (Figure 5B). Three sites (#4, #7, and #11) were associated with NRG under Al stress, two in high LD were found in the second intron and one independent site in the first exon (Figure 5C). The most significant independent site (#4) explains 1.4% of the variance in the association panel, which translates to an 18.4% increase in NRG under Al stress. We also examined the far upstream region in the association panel, which contained two large indels, but no significant associations were found.

Site #1, an indel in the first intron, was used for the linkage analysis of the B73×CML333 F_2_ population. The superior B73 allele of *ME* was correlated with a 21% increase in NRG, similar to the effect seen in the association mapping. The heterozygous class was intermediate in phenotypic effect, unlike that seen with *ZmASL* or *SAHH*, suggesting that the mode of action was additive rather than dominant.

### Al tolerance gene: *ALMT2*

**Figure 6:**
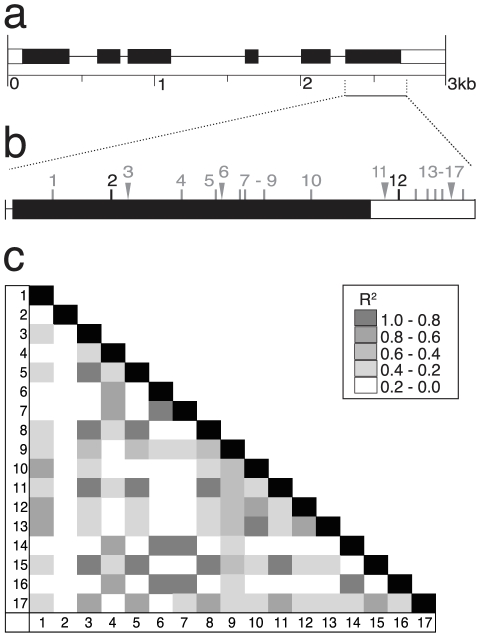
Characterization of *ZmALMT2*. (**a**) Predicted gene structure for the *ZmALMT2* locus is shown, with exons as black boxes, introns as thin lines, and UTRs as open boxes. For ease of presentation, polymorphisms detected in the 27 DL complete gene sequencing are not shown. (**b**) A focus region of *ZmALMT2* was sequenced in the association panel. Four indels and thirteen SNPs were detected; two SNPs were significantly associated with aluminum tolerance and are shown in black (#2, #12). (**c**) Linkage disequilibrium plot for all polymorphisms detected within the focus region at *ZmALMT2*. Site 2 is associated with aluminum tolerance differences but is in linkage equilibrium with all other sites, while site 12 has moderate linkage disequilibrium with many sites within the focus region.

We evaluated seven members of the *ZmALMT* gene family by association analysis. Only *ZmALMT2 (ALMT2)* gave a significant result (Figure 6). The gene model shown in Figure 6A is based on B73 sequence information only because of sequencing constraints due to paralogs within the *ZmALMT* family. Two SNPs in this gene (#2 and #12) were associated with NRG under the Q+K model (Figure 6B). These SNPs were independent of each other (R^2^<0.2), although LD was moderate to extensive between most of the SNPs found at this gene (Figure 6C). The most significant SNP explains 2.7% of the variation in the panel and confers a 20.2% increase in root growth.

Site #11, an indel that was not associated with Al tolerance, was used for the linkage analysis in the B73×NC350 F_2_ population. Like *ZmASL* and *SAHH*, the superior allele found in B73 was fully dominant to the allele found in the other parent. However, unlike *ZmASL* and *SAHH*, the enhancement in NRG correlated with *ALMT2* was somewhat smaller (15%) in the linkage population than expected from the association population (20.2%).

## Discussion

We found four new genes that may contribute to Al tolerance in maize by integrating several approaches. Candidate genes were selected based on comparative genomics and gene expression analysis, which we evaluated with association and linkage mapping. Two of these genes, *ZmALMT2* and *ZmASL*, are very similar to Al-activated organic transporters that play crucial roles in determining Al tolerance in other species [20], [39]–[43]. Whether these new maize genes are also membrane transporters has yet to be determined. *ME* and *SAHH* are involved in several central metabolism reactions and speculations on their contribution to Al tolerance can be made based on previous studies [47], [48]. The low heritability and complex nature of Al stress tolerance makes it challenging for both genetic improvement and genetic dissection. This complexity highlights the value for molecular markers for use in breeding programs for Al tolerance. To maximize their efficacy, molecular markers should be closely linked to major tolerance loci, so that markers are highly concordant with the desired genotypes. Given the limited amount of DNA sequence obtained for association mapping within many of the genes we investigated, we cannot positively identify these particular polymorphisms as causative without further investigation. However, they are most likely in LD with causative sites or contribute to an allelic series controlling Al tolerance, and therefore will be highly useful as markers for selection of Al tolerance materials. We demonstrated that using polymorphisms identified during gene sequencing as markers for linkage analysis allowed us to confirm the identification for four of the six putative Al tolerance genes. We utilized sites that were both significantly associated with Al tolerance differences in the association panel and sites not significantly associated, taking advantage of the difference in genetic resolution between association and linkage mapping. It was important to use linkage mapping to test the genes identified from association mapping, as we predicted a high rate for false discovery based on empirically calculating an FDR. Each of the Al tolerance loci produced similar phenotypic effects in both the association panel and F_2_ populations (13–20% increases in NRG). While none of these new Al tolerance genes represent major effect QTL, combining multiple small QTL can make a significant impact to enhance the desired trait. In the B73×CML247 F_2_ population, combining the elite alleles of *ZmASL* and *SAHH* enhanced net root growth by 30% (Tables 3 and S4).

*ZmASL* (*Zea mays Alt_S__B_like*) is a maize gene homologous to *Alt_SB_*, the major Al tolerance gene from sorghum and is a member of the Multidrug And Toxic Compound Extrusion (MATE) family of transporters [20]. Both proteins are predicted to contain 10 putative transmembrane domains. It is unknown whether the *ZmASL* gene mediates Al-activated root citrate efflux, as is the role of *Alt_SB_* in sorghum. Although many sites in *ZmASL* were associated with NRG under Al stress, none were amino acid substitutions. The significant sites we detected may be in LD with regulatory elements of the gene, as is the case in *Alt_SB_*, where polymorphisms in the promoter help to determine the level of gene expression [20]. *ZmASL* contained the most significant independent sites of any gene tested, but also contained extensive LD among many of the other significant sites. Fortunately, future experiments to evaluate the relationship of *ZmASL* with Al tolerance will be relatively straightforward given the presumed gene function.

*SAHH*, *S-adenosyl-L-homocysteine hydrolase*, is an enzyme that removes the feedback inhibitor of SAM (*S-adenosylmethionine*) mediated methylation in any organism [47]. Any enzymatic process that requires high rates of SAM-mediated methylation will also require high SAHH activity, including DNA/RNA modification, nucleic acid metabolism, and synthesis of cell wall constituents [49]. *SAHH* has a high degree of sequence conservation among eukaryotes [49]. In plants, *SAHH* is a cytokinin binding protein in plants, induced by auxin and cytokinin, and has been associated with salt-stress response in spinach and sugar beets and viral resistance in Arabidopsis [47], [50]. The isoform of maize *SAHH* we examined was previously found to be highly expressed in root tips under Al stress [38]. The connection of SAHH to Al tolerance could come through any of several mechanisms due to the broad range of processes the enzyme is involved in. However, given recent reports on the correlation pectin methylation in cell walls with Al tolerance and Al exclusion, it is certainly possible the *SAHH* contributes to Al tolerance differences via cell wall modification [22], [51].

*NADP-ME (ME)* catalyzes the conversion of malate to pyruvate. The maize *ME* examined in this study was the cytosolic rather than plastidic isoform of the enzyme. Maize *Cyt-ME* is highly similar to *Cyt-ME* found other in C3 and C4 plant species. This isoform was found to be expressed in the embryo and emerging roots, with expression responsive to hypoxia and drought [48]. High malate and other organic acid concentrations are optimal for activity of the cytosolic isoform and not inhibitory as is the case in plastidic isoforms of NADP-ME [48]. There is strong evidence that Al-activated release of malate underlies wheat Al tolerance [9], [17], [39]. Malate appears to chelate and detoxify Al in the apical rhizosphere or the apoplastic space. ME may help regulate malate concentration in the cytosol, which could connect to Al tolerance either through OA efflux or internal detoxification of Al via Al-OA chelation.

*ME* was unusual among the genes we examined as the results from linkage and association studies were opposite in direction, while still both highly significant. In the association mapping, three significant sites were identified – a site in the first exon (SNP #4), which was in linkage equilibrium with all other sites, and two sites in the second intron (SNP #7 and SNP #11), which were in high linkage disequilibrium with all of nearby the SNPs (Figure 5c). Based on these sites, we predicted that the B73 allele would be inferior to the CML333 allele. However, in the linkage mapping B73 was superior to CML333 (Figure 2). One possible explanation is that an allelic series exists at *ME* that was not observed in the polymorphic sites studied in the association panel. However, an allelic series could be detected in the larger linkage blocks of the segregating population. We see evidence of allelic series in several other candidate genes studies in maize, such as *su1* and *LcyE*, that also exhibit these inconsistencies between association and linkage mapping [52].

*ALMT2* is related to transport proteins that have been found to contribute to Al tolerance in *Triticum aestivum*, *Arabidopsis thaliana*, and *Brassica napus*, and are either activated or show enhanced malate efflux in response to external Al^3+^ [18], [39], [53]–[56]. It is proposed that binding of Al^3+^ to the transporter induces a conformational change, opening the anion channel [55], [56]. However, not all ALMT family proteins are Al-activated or important for Al tolerance processes. *AtALMT9* encodes a vacuolar malate transporter, instead of being localized to the plasma membrane like *AtALMT1* [18]. Unlike *AtALMT1*, *AtALMT9* is completely unresponsive to Al treatment [56]. The first *ZmALMT* family member to be characterized, *ZmALMT1*, transports inorganic anions and not malate, and is not activated by exogenous Al^3+^ [54]. Based on its transport properties and expression, *ZmALMT1* was determined not to be involved in maize Al tolerance. This is consistent with the results from the association analysis, as *ZmALMT1* was not associated with NRG under Al stress. Only *ZmALMT2* was found to be significant for Al tolerance of the seven *ZmALMT* genes that we evaluated by association analysis. Future work on *ZmALMT2* will include a biophysical characterization of the protein to verify that it does encode an Al-activated OA transporter.

In summary, we used association mapping to evaluate twenty-one candidate Al tolerance genes. Linkage mapping was used to test six putative Al tolerance genes found from association mapping; this was especially important given the high predicted FDR for the association mapping. Linkage mapping supported four of the six genes as true Al tolerance genes. These four genes, *ZmASL*, *ZmALMT2*, *ME and SAHH*, are excellent candidates for future laboratory and field-based studies on Al tolerance in maize. Although the most significant polymorphisms explain less than 3% of the variation seen in the association panel, our best marker can increase NRG up to 20%. If this increased root growth transfers to field trials, integration of these markers could substantially improve maize root growth and overall maize yield under Al toxic conditions.

## Materials and Methods

### Germplasm

The maize association population has been previously described [31], [34]. Linkage mapping experiments were conducted with three independent F_2_ populations derived from B73 and one of three other inbred lines from the maize association population (CML247, CML333, NC350). Non-B73 parents were selected on the basis of genotype information for the candidate Al tolerance genes.

### Phenotypic analysis

Maize seeds were germinated in either autoclaved sand or on filter paper, moistened with deionized water, for 3–5 d at 28°C in continuous darkness. Seedlings were rinsed and placed into sample cups suspended in 8L vessels containing a nutrient solution without Al^3+^ at pH 4.0, for 1 d, for acclimation to hydroponic conditions [37]. When plants were placed into hydroponic culture, secondary roots were removed to promote measurement of primary seminal root growth only. Tubs were aerated and plant grown under controlled environmental conditions (26°C day/24°C night, 16 h/8 h photoperiod). After 24 hrs of acclimation, initial root growth (IRG) measurements were taken using rulers with millimeter precision and solutions were replaced with Magnavaca nutrient solution containing {27 µM Al^3+^} at pH 4.0 (Al stress treatments) or Magnavaca nutrient solution containing no Al^3+^ at pH 4.0 (control treatments), for 2 d. After 2 d of Al stress final root growth (FRG) measurements were taken. Net root growth (NRG) was calculated as FRG – IRG.

Five replicate experiments were performed for Al stress treatments, while three replicates were performed for the control treatment. In each experiment, 3–4 individuals for each of the 282 inbred varieties in the association panel were phenotyped in each replicate experiment. Least squares means (LSmean) for both traits were calculated in SAS version 9.1 for Windows (SAS Institute Inc., Cary, NC, USA) and used as the phenotypic values in all models (Table S1).

F_2_ linkage populations were phenotyped in a similar manner, with {27 µM Al^3+^} at pH 4.0, with the modification that 200 F_2_ individuals were evaluated for each cross plus parental checks (n = 10). Leaf tissue was collected for DNA extraction and genotypic analysis after FRG measurements. Measurements of NRG under control and stress treatments are found in Table S4.

### Genotypes and candidate genes

All DNA was isolated using a standard CTAB extraction method [57]. DNA sequence analysis was performed using the BigDye® Terminator Cycle Sequencing kit according to manufacturer's instructions (Applied Biosystems, Foster City, CA, USA) and resolved on an ABI3730 Capillary Sequencer at the Cornell University Life Sciences Core Laboratory Center. Twenty-one candidate genes were successfully amplified from the 27 DL subset of the association panel, using 2 or more 600 bp amplicons. The amplicon with highest nucleotide diversity was selected for sequencing in the full association panel; all DNA sequences have been submitted to GenBank as entries GF102441 through GF107318 (4,878 sequences). Sequences were assembled using Biolign 4.0.7 [58]. These genes are named in Table 1; informative polymorphisms are listed in Table S2.

F_2_ populations were genotyped only for the loci that were expected to segregate in each cross. Molecular markers were developed from sequence analysis of each locus and evaluated using standard PCR methods on agarose gels for indels or by fluorescently labeled primers for SNPs (Table S5). Marker data was collected and organized using Genemapper software V4.0 (Applied Biosystems, Foster City, CA, USA).

Primers were designed based on reference sequences obtained from the Maize Assembled Genomic Island (MAGI) Database [45]. Genes of interest were placed on the physical-genetic map of maize using the BLAST tool implemented by the Maize Genome Sequencing Project [42]. Gene architecture predictions were made using the FGenesH tool as implemented by Softberry [46].

### Statistical tests

TASSEL 1.9.6 was used to evaluate linkage disequilibrium (LD), extract polymorphic sites, calculate narrow sense heritability, and perform General and Mixed Linear Models (GLM, MLM) with incorporation of trait data, population structure (Q) and kinship matrix (K) [59]. All other statistical analyses were done using SAS version 9.1. A t-test was used to analyze differences between NRG in the association panel under control and Al stress.

### Association mapping

The MLM approach and estimation of the kinship matrix (K) has been previously described [31]. Population structure estimates (Q) have been previously described [34]. The complete results from MLM appear as Table S3.

The mixed model used, for vector of phenotypes, y, is:
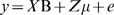
where all fixed effects are modeled in the 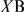 term, including genotypes and Q. Random effects are modeled in the 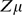 term, including the matrix of kinship coeffiecients, K, and vector of polygene background effects. 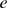 is a vector of residual effects. This model is referred to as the Q+K model. Addition of Control NRG as a fixed effect covariate in the model is referred to as the Q+K+C model.

Polymorphic sites tested, SNPs and indels, that occurred ≥10% were extracted from aligned sequence data. A total of 331 sites were across 21 genes were used (Table S2). Sites for the AUX1 locus were reduced to only those not in complete LD (R^2^ = 1) due to an excessive (73) number of sites in LD. Lines with quality scores less than 60% were discarded.

### FDR

In order to account for expected false positives present due to multiple testing, a False Discovery Rate (FDR) was calculated using 1095 SNPs that occur randomly across the maize genome [60]. 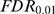 was calculated as:
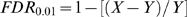
Where 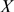 is the proportion of sig sites from the candidate genes ≤ the significance value specified (P<0.01). 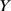 is the proportion of sig sites from the 1095 random SNPs ≤ the significance value specified. Significant sites were calculated from GLM or MLM using NRG LSmeans as the trait value.

### Heritability

Marker based narrow sense heritability (h^2^) was calculated in TASSEL using the kinship matrix (K) as a parent-offspring regression. Broad sense heritability (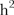) was calculated in SAS as:
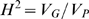
where 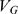 is the total genotype variance and 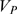 is the total phenotypic variance.

## Supporting Information

Table S1 Net seminal root growth data. Least Squares means were calculated for net root growth (mm 2d-1) for the association panel in the Al-stress condition (“Lmeans-Al treatment”, based on 5 replicate experiments) and control condition (“Lsmean-control”, based on 3 replicate experiments).
(0.03 MB PDF)

Table S2 Sequence polymorphisms utilized for association analysis. Polymorphic sites (SNPs and indels) were identified in each of the 21 candidate Al tolerance genes across the 282 member association panel. SNPs are coded as nucleotides (ACGT), indels are coded as numbers (e.g., 0 vs. 2), while missing data appear as N.
(0.61 MB PDF)

Table S3 Mixed Linear Model (MLM) based association analysis. MLM analysis was used to evaluate the importance for each polymorphic site in every candidate Al tolerance gene for NRG. Al-stress and control growth conditions were evaluated separately. All results are reported here.
(0.18 MB PDF)

Table S4 Validation of association mapping via linkage mapping. Association mapping results were validated using linkage mapping of F_2_ populations segregating for the candidate Al tolerance genes. This table reports phenotypic and genotypic information for the linkage experiments.
(0.05 MB PDF)

Table S5 PCR primers utilized for linkage mapping.
(0.02 MB PDF)
