# Supplementary material for: Association and Linkage Analysis of Aluminum Tolerance Genes in Maize
Source: PLoS One. 2010 Apr 1;5(4):e9958. doi: 10.1371/journal.pone.0009958 (PMC2848604; doi:10.1371/journal.pone.0009958)
Supplement: Table S1 — Net seminal root growth data. Least Squares means were calculated for net root growth (mm 2d-1) for the association panel in the Al-stress condition (“Lmeans-Al treatment”, based on 5 replicate experiments) and control condition (“Lsmean-control”, based on 3 replicate experiments). (0.03 MB PDF) [file pone.0009958.s001.pdf]

TAXA	Lsmean-control	Lsmean-Al treatment
811	36.38	34.22
3316	.	16.42
3811	47.5	27.7
4226	23.5	28.8
4722	.	3.67
A188	29.67	60.3
A214N	23.17	18.1
A239	47.67	42
A272	.	31.92
A4415	.	31.92
A554	66.83	36.8
A556	30	28.7
A6	32.88	27.53
A619	51.33	39.6
A632	32.17	29.2
A634	46	43.9
A635	48.88	24.37
A641	47.33	36.7
A654	64.33	48.3
A659	63	77.4
A661	61.67	50
A679	44.83	24.1
A680	35	35.3
A682	53.63	43.07
AB28A	50.5	33.4
B10	.	53.7
B103	62.33	45
B104	67.33	46.1
B105	60.17	35.9
B109	50.5	30.2
B115	52.4	48.62
B14A	40.88	31.2
B164	56.17	23.5
B2	.	35.65
B37	51.17	41.33
B46	47.88	24.7
B52	.	11.92
B57	39.17	37.6
B64	33.38	39.7
B68	28	25
B73	34.67	31.5
B73HTRHM	43.17	40.4
B75	48.17	54
B76	44	41.3
B77	33.33	41.1
B79	59.4	36.78
B84	44.17	27.7
B97	63	17.6
C103	50.67	27.2
C123	51	33
C164	.	26.2
C49	38	28
CH70130	69.67	53.3
CH9	59.17	48.6
CI1872	41.33	29.7
CI21E	39.33	38.1
CI28A	62.17	47

CI31A	24.33	27.1
CI3A	34.5	32.9
CI44	30	21.05
CI64	33.83	23.45
CI66	34.67	41.1
CI7	.	31.15
CI90C	58.67	24.1
CI91B	78.5	60.55
CM105	32.67	35.7
CM174	36.9	46.28
CM37	42.5	48.88
CM7	32.67	34
CML10	65.4	62.85
CML103	62.4	54.28
CML108	54.5	43.3
CML11	66.5	67.6
CML14	47.5	24
CML154Q	70.33	22.4
CML157Q	47.33	50.1
CML158Q	55	48
CML218	50.67	37.5
CML220	50.17	31.1
CML227	.	40.7
CML228	51	26.7
CML238	39.33	41.5
CML247	47.63	37.95
CML254	.	44.67
CML258	34.83	21.4
CML261	58.33	29.88
CML277	64.33	39.2
CML281	.	15.15
CML287	26.33	28
CML311	.	10.42
CML314	59.67	50.4
CML321	49.13	27.45
CML322	.	19.42
CML323	25.17	30.8
CML328	56.33	23.4
CML331	.	36.65
CML332	64.5	34.9
CML333	51.67	43.9
CML341	66.5	46
CML38	49.88	37.07
CML45	52.5	43.7
CML5	.	37.92
CML52	37	23.5
CML61	.	27.15
CML69	59.33	51.9
CML77	60.67	39.8
CML91	47.67	37
CML92	75.17	36.8
CMV3	42	37.4
CO106	.	21.92
CO125	39.5	27.95
CO255	48.83	39.7
D940Y	73.5	37.2
DE1	64.33	41
DE2	58.33	43.1

DE3	59.17	42.7
DE811	56	43.8
E2558W	57.33	47.2
EP1	48.33	37.05
F2	35.33	35.72
F2834T	63	49.3
F44	79.5	52.8
F6	59.67	49.2
F7	45.5	37.8
GA209	39.33	27.9
GT112	46.9	20.45
H100	50.4	23.5
H105W	37.4	15.45
H49	68.17	45.1
H84	44.67	45
H91	51.17	36.6
H95	49.4	26.95
H99	54.5	32.4
Hi27	63.33	37.1
HP301	44.9	34.78
Hy	38.4	37.28
I137TN	38.5	40.05
I205	.	24.67
I-29	44.83	28
IA2132	55.83	24.3
Ia5125	33.83	35.95
IDS28	54	51.2
IDS69	59.4	36.62
IDS91	54.33	45
IL101	56.67	48.1
IL14H	56.83	42.8
IL677A	39.17	40.3
K148	57	43.9
K4	38.83	35.6
K55	56.63	37.2
K64	52.38	41.2
Ki11	38.17	34.4
Ki14	52.67	42.5
Ki2021	61.13	47.57
Ki21	35	37.2
Ki3	51.67	28.55
Ki43	42.33	30.8
Ki44	49.5	40.4
KY21	63	58
Ky226	.	24.92
Ky228	.	38.17
L317	65.5	55.45
L578	61	44.3
M14	48.5	31.3
M162W	18.4	52.78
M37W	39.33	41.1
MEF15655	57.83	34.08
MO17	44.67	27.7
MO18W	29.5	23
MO1W	43.5	38.5
MO24W	54.33	42.5
MO44	58.33	34.9
MO45	46.67	33.4

MO46	52.67	26.7
MO47	58.5	35.9
MOG	44.13	54.07
MP339	38.5	57
MS1334	41	39.3
MS153	46.33	27.4
MS71	48.67	48.1
MT42	43.33	50.1
N192	44.33	51.5
N28HT	50.33	37
N6	45.83	46.21
N7A	51.17	32.2
NC222	46.67	46.3
NC230	54.83	40.3
NC232	62.67	50.1
NC236	58.83	49.5
NC238	64.17	63.8
NC250	38.5	30.3
NC258	48.83	59.2
NC260	42.83	25
NC262	48.67	37.6
NC264	65	63.3
NC268	33.17	26.88
NC290A	41.33	49.1
NC292	51.5	32.72
NC294	46.83	36.8
NC296	56	27.4
NC296A	54.38	24.71
NC298	42.5	22.6
NC300	50	29.8
NC302	46.17	30
NC306	18.4	32.95
NC308	38.5	35.22
NC310	50	38.2
NC312	44	31.88
NC316	42	33.2
NC318	53.5	59
NC320	69.67	67.1
NC322	55.17	59.55
NC324	44.83	28.6
NC326	51	37.2
NC328	43.33	38.7
NC33	52.83	26.59
NC330	54.33	35.55
NC332	61.5	59.55
NC334	63.5	62.22
NC336	32.4	24.6
NC338	21.5	32.9
NC340	51.4	30.12
NC342	49.5	44.7
NC344	57.4	60.08
NC346	52.5	27.5
NC348	40.4	37.28
NC350	44.4	26.78
NC352	31.4	12.58
NC354	49	25.6
NC356	39.83	26.8
NC358	51.83	32.8

NC360	43.83	33.7
NC362	45.33	42.9
NC364	58.33	48.4
NC366	60	57.7
NC368	61.83	41.7
NC370	74.5	64.38
NC372	52	37.05
ND246	63.33	38.8
Oh40B	45.38	24.57
OH43	56.4	15.1
OH43E	48.4	18.45
OH603	36.17	23.8
OH7B	55.67	18
OS420	46	31.1
P39	50.67	26.4
PA762	52.4	18.95
PA875	58.17	71.2
PA880	60.67	38.4
PA91	66.17	41.2
Q6199	41.9	24.5
R168	54.33	41.6
R177	39	27.2
R229	43.38	34.07
R4	59	52
SA24	62.83	41
SC213R	62.17	53.1
SC357	49.83	53.6
SC55	47.33	37.3
SD40	58	20.1
SD44	68	37.8
Sg1533	37.33	28.2
SG18	39.67	30.6
T232	47.63	34.82
T234	47.83	32.1
T8	66.5	59.4
TX303	73.5	37.2
TX601	53.5	38
TZI10	46	26.5
TZi11	58.5	60.8
TZi16	56.17	52.2
TZI18	52.83	30.2
TZi25	52.67	37.8
TZI8	71.38	67.57
TZi9	57	41.4
U267Y	45.38	36.07
VA102	44.33	48.4
VA14	56.5	49.3
VA17	54.5	48.1
VA22	63	56.6
VA26	.	46.92
VA35	84.63	55.82
VA59	65.83	35.9
VA85	34	33.95
VA99	54.67	50.9
VAW6	59	39
W117HT	55.83	38.6
W401	70.4	46.62
W64A	47.33	39.9

WD	38.83	33.2
WF9	52.83	47
Yu796NS	49	39.9
