# Supplementary material for: Association and Linkage Analysis of Aluminum Tolerance Genes in Maize
Source: PLoS One. 2010 Apr 1;5(4):e9958. doi: 10.1371/journal.pone.0009958 (PMC2848604; doi:10.1371/journal.pone.0009958)
Supplement: Table S2 — Sequence polymorphisms utilized for association analysis. Polymorphic sites (SNPs and indels) were identified in each of the 21 candidate Al tolerance genes across the 282 member association panel. SNPs are coded as nucleotides (ACGT), indels are coded as numbers (e.g., 0 vs. 2), while missing data appear as N. (0.61 MB PDF) [file pone.0009958.s002.pdf]

taxa	AUX01	AUX02	AUX03	AUX04	AUX05	AUX06	AUX07	AUX08	AUX09	AUX10	AUX11
TEST	T	A	2	C	C	A	G	G	G	C	G
3316	T	A	2	C	C	A	G	G	G	C	G
3811	C	G	2	A	G	G	G	A	A	C	G
4226	N	N	N	N	N	N	N	N	N	N	N
4722	C	G	0	A	A	G	G	A	A	C	T
A188	N	N	N	N	N	N	N	N	N	N	N
A214N	C	G	0	A	A	G	G	A	A	C	T
A239	C	G	2	A	A	G	A	G	G	C	C
A272	N	N	N	N	N	N	N	N	N	N	N
A4415	N	N	N	N	N	N	N	N	N	N	N
A554	T	A	2	C	C	A	G	G	G	C	G
A556	C	G	2	A	A	G	G	T	G	C	G
A6	T	A	2	C	C	A	G	G	G	C	G
A619	T	A	2	C	C	A	G	G	G	C	G
A632	N	N	N	N	N	N	N	N	N	N	N
A634	C	G	0	A	A	G	G	A	A	C	T
A635	C	G	2	A	A	G	A	G	G	C	C
A641	C	G	0	A	A	G	G	A	A	C	T
A654	C	G	2	A	A	G	A	G	G	C	C
A659	T	A	2	C	C	A	G	G	G	C	G
A661	C	G	2	C	C	G	G	-	A	A	G
A679	N	N	N	N	N	N	N	N	N	N	N
A680	C	G	0	A	A	G	G	A	A	C	T
A682	C	G	2	A	A	G	G	T	G	C	G
AB28A	C	G	2	A	G	G	G	A	A	C	G
B10	C	G	2	A	A	G	A	G	G	C	C
B103	C	G	2	A	A	G	A	G	G	C	C
B104	N	N	N	N	N	N	N	N	N	N	N
B105	N	G	2	A	A	A	G	N	N	N	N
B109	C	G	0	A	A	G	G	A	A	C	T
B115	N	N	N	N	N	N	N	N	N	N	N
B14A	C	G	0	A	A	G	G	A	A	C	T
B164	C	G	2	A	A	G	G	T	G	C	G
B2	C	G	2	A	A	G	A	G	G	C	C
B37	C	G	2	A	A	A	G	G	G	T	G
B46	C	G	2	A	A	G	A	G	G	C	C
B52	N	N	N	N	N	N	N	N	N	N	N
B57	C	G	2	A	A	G	A	G	G	C	C
B64	N	N	N	N	N	N	N	N	N	N	N
B68	C	G	0	A	A	G	G	A	A	C	T
B73	C	G	0	A	A	G	G	A	A	C	T
B73HTRHM	C	G	0	A	A	G	G	A	A	C	T
B75	C	G	2	A	A	T	G	A	A	C	G
B76	C	G	2	A	A	A	G	G	G	T	G
B77	C	G	2	A	A	A	G	G	G	T	G
B79	C	G	2	A	A	G	A	G	G	C	C
B84	C	G	0	A	A	G	G	A	A	C	T
B97	C	G	2	A	A	A	G	G	G	T	G
C103	C	G	2	A	A	G	A	G	G	C	C
C123	C	G	2	A	A	G	A	G	G	C	C
C49	C	G	0	A	A	G	G	A	A	C	T
CH70130	C	G	2	A	A	G	A	G	G	C	C
CH9	C	G	2	A	A	G	A	G	G	C	C
CI7	C	G	2	A	A	G	A	G	G	C	C
CI1872	N	-	2	C	C	A	G	G	G	C	G
CI21E	N	N	N	N	N	N	N	N	N	N	N

CI28A	C	G	2	A	A	G	A	G	G	C	C
CI31A	C	G	0	A	A	G	G	A	A	C	T
CI3A	T	A	2	C	C	A	G	G	G	C	G
CI64	T	A	2	C	C	A	G	G	G	C	G
CI66	N	N	N	N	N	N	N	N	N	N	N
CI90C	C	G	0	A	A	G	G	A	N	N	N
CI91B	C	G	2	A	A	G	G	T	G	C	G
CM105	C	G	0	A	A	G	G	A	A	C	T
CM174	C	G	0	A	A	G	G	A	A	C	T
CM37	N	N	N	N	N	N	N	N	N	N	N
CM7	C	G	0	A	A	G	G	A	A	C	T
CML10	N	N	N	N	N	N	N	N	N	N	N
CML103	N	N	N	N	N	N	N	N	N	N	N
CML108	C	G	2	A	A	G	G	T	G	C	G
CML11	N	N	N	N	N	N	N	N	N	N	N
CML14	C	G	0	A	A	G	G	A	A	C	T
CML154Q	C	G	2	A	A	G	A	G	G	C	C
CML157Q	T	A	2	C	C	A	G	G	G	C	G
CML158Q	C	G	0	A	A	G	G	A	A	C	T
CML218	N	N	N	N	N	N	N	N	N	N	N
CML220	C	G	0	A	A	G	G	A	A	C	T
CML227											
CML228	N	N	N	N	N	N	N	N	N	N	N
CML238	C	G	0	A	A	G	G	A	A	C	T
CML247	N	N	N	N	N	N	N	N	N	N	N
CML254	C	G	0	A	A	G	G	A	N	N	N
CML258	N	N	N	N	N	N	N	N	N	N	N
CML261	C	G	0	A	A	G	G	A	A	C	T
CML264	C	G	0	A	A	G	G	A	A	C	T
CML277	C	G	0	A	A	G	G	A	A	C	T
CML281	N	N	N	N	N	N	N	N	N	N	N
CML287	C	G	0	A	A	G	G	A	A	C	T
CML311	C	G	0	A	A	G	G	A	A	C	T
CML314	C	G	2	A	A	G	A	G	G	C	C
CML321	T	A	2	C	C	A	G	G	G	C	G
CML322	C	G	2	A	A	G	A	G	G	C	C
CML323	N	N	N	N	N	N	N	N	N	N	N
CML328	C	G	2	A	G	G	G	N	N	N	N
CML331	N	G	2	A	A	G	A	G	G	C	C
CML332	N	N	N	N	N	N	N	N	N	N	N
CML333	C	G	0	A	A	G	G	A	A	C	T
CML341	N	G	0	A	A	G	G	A	N	N	N
CML38	N	N	N	N	N	N	N	N	N	N	N
CML45	C	G	0	A	A	G	G	A	A	C	T
CML5	N	N	N	N	N	N	N	N	N	N	N
CML52	N	N	N	N	N	N	N	N	N	N	N
CML61	N	N	N	N	N	N	N	N	N	N	N
CML69	C	G	0	A	A	G	G	A	A	C	T
CML77	N	N	N	N	N	N	N	N	N	N	N
CML91	N	N	N	N	N	N	N	N	N	N	N
CML92	N	N	N	N	N	N	N	N	N	N	N
CMV3	T	A	2	C	C	A	G	G	G	C	G
CO106	N	N	N	N	N	N	N	N	N	N	N
CO125	C	G	0	A	A	G	G	A	A	C	T
CO255	C	G	2	C	C	G	G	-	A	A	G
D940Y	N	N	N	N	N	N	N	N	N	N	N
DE1	C	G	2	A	A	G	A	G	G	C	C

DE2	C	G	2	A	A	G	A	G	G	C	C
DE3	N	N	N	N	N	N	N	N	N	N	N
DE811	C	G	0	A	A	G	G	A	A	C	T
E2558W	C	G	0	A	A	G	G	A	A	C	T
EP1	C	G	2	A	A	G	A	G	G	C	C
F2834T	T	A	2	C	C	A	G	G	G	C	G
F44	T	A	2	C	C	A	G	G	G	C	G
F6	T	A	2	C	C	A	G	G	G	C	G
F7	C	G	2	C	C	G	G	-	A	A	G
GA209	N	N	N	N	N	N	N	N	N	N	N
GT112	C	G	0	A	A	G	G	A	A	C	T
H105W	N	A	2	C	C	A	G	G	G	C	G
H49	N	N	N	N	N	N	N	N	N	N	N
H84	C	G	2	A	A	A	G	G	G	T	G
H91	C	G	0	A	A	G	G	A	A	C	T
H95	N	A	2	C	C	A	G	G	G	C	G
H99	N	N	N	N	N	N	N	N	N	N	N
Hi27	N	N	N	N	N	N	N	N	N	N	N
HP301	C	G	0	A	A	G	G	A	A	C	T
HY	N	A	2	C	C	A	G	G	G	C	G
I137TN	N	N	N	N	N	N	N	N	N	N	N
I205	T	A	2	C	C	A	G	G	G	C	G
I29	T	A	2	C	C	A	G	G	G	C	G
IA2132	C	G	2	A	A	A	G	G	G	T	G
IA5125	C	G	0	A	A	G	G	A	A	C	T
IDS28	C	G	2	A	A	G	G	T	G	C	G
IDS69	C	G	0	A	A	G	G	A	A	C	T
IDS91	C	G	0	A	A	G	G	A	A	C	T
IL101	C	G	0	A	A	G	G	A	A	C	T
IL14H	C	G	2	A	A	G	G	T	G	C	G
IL677A	C	G	2	A	A	T	G	A	A	C	G
K148	T	A	2	C	C	A	G	G	G	C	G
K4	T	A	2	C	C	A	G	G	G	C	G
K55	N	N	N	N	N	N	N	N	N	N	N
K64	T	A	2	C	C	A	G	G	G	C	G
Ki11	C	G	2	A	G	G	G	A	A	C	G
Ki14	C	G	0	A	A	G	G	A	A	C	T
Ki2021	C	G	0	A	A	G	G	A	A	C	T
Ki21	N	N	N	N	N	N	N	N	N	N	N
Ki3	C	G	0	A	A	G	G	A	A	C	T
Ki43	N	N	N	N	N	N	N	N	N	N	N
Ki44	N	N	N	N	N	N	N	N	N	N	N
KY21	N	N	N	N	N	N	N	N	N	N	N
Ky226	N	N	N	N	N	N	N	N	N	N	N
Ky228	N	N	N	N	C	A	G	G	G	C	G
L317	C	G	2	A	A	A	G	G	G	T	G
L578	T	A	2	C	C	A	G	G	G	C	G
M14	T	A	2	C	C	A	G	G	G	C	G
M162W	C	G	0	A	A	G	G	A	A	C	T
M37W	N	N	N	N	N	N	N	N	N	N	N
MEF15655	C	G	0	A	A	G	G	A	A	C	T
MO17	C	G	2	A	A	G	G	T	G	C	G
MO18W	C	G	2	A	A	G	A	G	G	C	C
MO1W	C	G	2	A	A	G	A	G	G	C	C
MO24W	C	G	2	A	G	G	G	A	A	C	G
MO44	C	G	2	A	A	A	G	G	G	T	G
MO45	N	N	N	N	N	N	N	N	N	N	N

MO46	N	N	N	N	N	N	N	N	N	N	N
MO47	C	G	2	A	A	A	G	G	G	T	G
MOG	C	G	2	A	A	A	G	G	G	T	G
MP339	C	G	2	A	A	G	A	G	G	C	C
MS1334	C	G	0	A	A	G	G	A	A	C	T
MS153	C	G	0	A	A	G	G	A	A	C	T
MS71	N	N	N	A	A	G	A	N	N	N	N
MT42	C	G	2	A	A	G	G	T	G	C	G
N192	C	G	0	A	A	G	G	A	A	C	T
N28HT	C	G	2	A	A	G	A	G	G	C	C
N6	N	G	0	A	A	G	N	N	N	N	N
N7A	N	N	N	N	N	N	N	N	N	N	N
NC222	N	N	2	C	C	A	G	G	G	C	G
NC230	C	G	2	C	C	G	G	-	A	A	G
NC232	C	G	2	A	A	G	A	G	G	C	C
NC236	N	N	N	N	N	N	N	N	N	N	N
NC238	N	N	N	N	N	N	N	N	N	N	N
NC250	C	G	2	A	A	A	G	G	G	T	G
NC258	C	G	2	A	A	G	A	G	G	C	C
NC260	N	N	N	N	G	G	N	N	N	N	N
NC262	C	G	2	A	G	G	G	A	A	C	G
NC264	N	N	N	N	N	N	N	N	N	N	N
NC290A	C	G	2	A	G	G	G	A	A	C	G
NC294	C	G	0	A	A	G	G	A	A	C	T
NC296	N	N	N	N	N	N	N	N	N	N	N
NC296A	C	G	2	A	A	A	G	G	G	T	G
NC298	C	G	0	A	A	G	G	A	A	C	T
NC300	C	G	0	A	A	G	G	A	A	C	T
NC302	N	N	N	N	N	N	N	N	N	N	N
NC304	N	N	N	N	N	N	N	N	N	N	N
NC306	C	G	0	A	A	G	G	A	A	C	T
NC310	C	G	0	A	A	G	G	A	A	C	T
NC314	C	G	2	A	A	A	G	G	G	T	G
NC318	C	G	2	A	A	G	A	G	G	C	C
NC320	C	G	2	A	A	G	A	G	G	C	C
NC324	C	G	2	A	A	A	G	G	G	T	G
NC326	C	G	0	A	A	G	G	A	A	C	T
NC328	C	G	0	A	A	G	G	A	A	C	T
NC33	C	G	2	A	A	G	A	G	G	C	C
NC336	C	G	0	A	A	G	G	A	A	C	T
NC338	N	N	N	N	N	N	N	N	N	N	N
NC340	T	A	2	C	C	A	G	G	G	C	G
NC342	N	N	N	N	N	N	N	N	N	N	N
NC344	N	N	N	N	N	N	N	N	N	N	N
NC346	C	G	2	A	A	G	A	G	G	C	C
NC348	C	G	0	A	A	G	G	A	A	C	T
NC350	N	N	N	N	N	N	N	N	N	N	N
NC352	C	G	0	A	A	G	G	A	A	C	T
NC354	C	G	0	A	A	G	G	A	A	C	T
NC356	T	A	2	C	C	A	G	G	G	C	G
NC358	T	A	2	C	C	A	G	G	G	C	G
NC360	C	G	2	A	G	G	G	A	A	C	G
NC362	C	G	0	A	A	G	G	A	A	C	T
NC364	C	G	0	A	A	G	G	A	A	C	T
NC366	C	G	2	A	A	A	G	G	G	T	G
NC368	C	G	2	A	A	A	G	G	G	T	G
ND246	C	G	0	A	A	G	G	A	A	C	T

OH40B	C	G	2	A	A	A	G	G	G	T	G
OH43	N	N	N	N	C	A	G	G	G	C	G
OH43E	C	G	2	A	A	G	A	G	G	C	C
OH603	N	N	N	N	N	N	N	N	N	N	N
OH7B	C	G	2	A	A	G	A	G	G	C	C
Os420	C	G	2	A	A	G	A	G	G	C	C
P39	C	G	2	A	A	G	G	T	G	C	G
PA762	C	G	2	A	A	A	G	G	G	T	G
PA875	N	N	N	N	N	N	N	N	N	N	N
PA880	N	N	N	N	N	N	N	N	N	N	N
PA91	C	G	2	A	A	G	A	G	G	C	C
Q6199	N	N	N	N	N	N	N	N	N	N	N
R168	C	G	2	A	A	G	A	G	G	C	C
R177	T	A	2	C	C	A	G	G	G	C	G
R229	T	A	2	C	C	A	G	G	G	C	G
R4	T	A	2	C	C	A	G	G	G	C	G
SA24	N	N	N	N	N	N	N	N	N	N	N
SC213R	N	N	N	N	N	N	N	N	N	N	N
SC357	C	G	0	A	A	G	G	A	A	C	T
SC55	N	N	N	N	N	N	N	N	N	N	N
SD40	C	G	2	A	A	G	G	T	G	C	G
SD44	C	G	2	A	A	G	A	G	G	C	C
SG1533	C	G	0	A	A	G	G	A	N	N	N
SG18	C	G	0	A	A	G	G	A	A	C	T
T232	C	G	0	A	A	G	G	A	A	C	T
T234	N	N	N	N	N	N	N	N	N	N	N
T8	N	N	N	N	N	N	N	N	N	N	N
Tx303	N	N	N	N	N	N	N	N	N	N	N
TX601	N	N	N	N	N	N	N	N	N	N	N
TZI10	N	N	N	N	N	N	N	N	N	N	N
TZI11	N	N	N	N	N	N	N	N	N	N	N
TZI16	T	A	2	C	C	A	G	G	G	C	G
TZI18	N	N	N	N	N	N	N	N	N	N	N
TZI25	C	G	0	A	A	G	G	A	A	C	T
TZI8	C	G	2	A	A	G	A	G	G	C	C
TZI9	N	N	N	N	N	N	N	N	N	N	N
U267Y	C	G	2	A	A	G	A	G	G	C	C
VA102	C	G	2	A	A	G	A	G	G	C	C
VA14	N	G	2	A	A	G	A	G	N	N	N
VA17	C	G	2	A	A	G	A	G	G	C	C
VA22	C	G	2	A	A	G	A	G	G	C	C
VA26	T	A	2	C	C	A	G	G	G	C	G
VA35	N	N	N	N	N	N	N	N	N	N	N
VA59	C	G	2	A	A	G	A	G	G	C	C
VA85	T	A	2	C	C	A	G	G	G	C	G
VA99	C	G	2	A	A	G	A	G	G	C	C
VAW6	N	N	N	N	N	N	N	N	N	N	N
W117HT	C	G	2	A	A	G	A	G	G	C	C
W153R	N	N	N	N	N	N	N	N	N	N	N
W182B	T	A	2	C	C	A	G	G	G	C	G
W22	T	A	2	C	C	A	G	G	G	C	G
W22R	T	A	2	C	C	A	G	G	G	C	G
W64A	C	G	2	A	A	G	A	G	G	C	C
WD	T	A	2	C	C	A	G	G	G	C	G
WF9	C	G	2	A	A	G	A	G	G	C	C
YU796NS	C	G	2	A	A	G	A	G	G	C	C

AUX12	AUX13	AUX14	AUX15	AUX16	AUX17	AUX18	AUX19	AUX20	AUX21	AUX22	AUX23
1	A	2	C	G	G	T	G	C	G	T	C
0	A	2	C	G	G	T	G	C	G	T	C
0	T	1	C	T	T	C	A	C	A	N	N
N	N	N	N	N	N	N	N	N	N	N	N
0	T	1	C	T	G	C	G	T	G	C	T
N	N	N	N	N	N	N	N	N	N	N	N
0	T	1	C	T	G	C	G	T	G	C	T
1	T	1	T	T	T	C	G	C	G	G	T
N	N	N	N	N	N	N	N	N	N	N	N
N	N	N	N	N	N	N	N	N	N	N	N
0	A	2	C	G	G	T	G	C	G	T	C
1	T	1	C	T	G	C	G	C	G	C	T
0	A	2	C	G	G	T	G	C	G	T	C
0	A	2	C	G	G	T	G	C	G	T	C
N	N	N	N	N	N	N	N	N	N	N	N
0	T	1	C	T	G	C	G	T	G	C	T
1	T	1	T	T	T	C	G	C	G	G	T
0	T	1	C	T	G	C	G	T	G	C	T
1	T	1	T	T	T	C	G	C	G	G	T
0	A	2	C	G	G	T	G	C	G	T	C
0	C	2	C	A	G	T	G	C	G	T	A
N	N	N	N	N	N	N	N	N	N	N	N
0	T	1	C	T	G	C	G	T	G	C	T
1	T	1	C	T	G	C	G	C	G	C	T
0	T	1	C	T	T	C	G	C	A	C	T
1	T	1	T	T	T	C	G	C	G	G	T
1	T	1	T	T	T	C	G	C	G	G	T
N	N	N	N	N	N	N	N	N	N	N	N
N	N	N	N	N	N	N	N	N	N	N	N
0	T	1	C	T	G	C	G	T	G	C	T
N	N	N	N	N	N	N	N	N	N	N	N
0	T	1	C	T	G	C	N	N	N	N	N
1	T	1	C	T	G	C	G	C	G	C	T
1	T	1	T	T	T	C	G	C	G	G	T
1	T	1	C	T	G	T	A	C	A	G	T
1	T	1	T	T	T	C	G	C	G	G	T
N	N	N	N	N	N	N	N	N	N	N	N
1	T	1	T	T	T	C	G	N	N	N	N
N	N	N	N	N	N	N	N	N	N	N	N
0	T	1	C	T	G	C	G	T	G	C	T
0	T	1	C	T	G	C	G	T	G	C	T
0	T	1	C	T	G	C	G	T	G	C	T
0	T	1	T	T	G	C	G	C	G	C	T
1	T	1	C	T	G	T	A	C	A	G	T
1	T	1	C	T	G	T	A	C	A	G	T
1	T	1	T	T	T	C	G	C	G	G	T
0	T	1	C	T	G	C	G	T	G	C	T
1	T	1	C	T	G	T	A	C	A	G	T
1	T	1	T	T	T	C	G	C	G	G	T
1	T	1	T	T	T	C	G	C	G	G	T
0	T	1	C	T	G	C	G	N	N	N	N
1	T	1	T	T	T	C	G	C	G	G	T
1	T	1	T	T	T	C	G	C	G	G	T
1	T	1	T	T	T	C	G	C	G	G	T
0	A	2	C	N	N	N	N	N	N	N	N
N	N	N	N	N	N	N	N	N	N	N	N

1	T	1	T	T	T	C	G	C	G	G	T
0	T	1	C	T	G	C	G	T	G	C	T
0	A	2	C	G	G	T	G	C	G	T	C
0	A	2	C	G	G	T	G	C	G	T	C
N	N	N	N	N	N	N	N	N	N	N	N
N	N	N	N	N	N	N	N	N	N	N	N
1	T	1	C	T	G	C	G	C	G	C	T
0	T	1	C	T	G	C	G	T	G	C	T
0	T	1	C	T	G	C	G	T	G	C	T
N	N	N	N	N	N	N	N	N	N	N	N
0	T	1	C	T	G	C	G	T	G	C	T
N	N	N	N	N	N	N	N	N	N	N	N
N	N	N	N	N	N	N	N	N	N	N	N
1	T	1	C	T	G	C	G	C	G	C	T
N	N	N	N	N	N	N	N	N	N	N	N
0	T	1	C	T	G	C	G	T	G	C	T
1	T	1	T	T	T	C	G	C	G	G	T
0	A	2	C	G	G	T	G	C	G	T	C
0	T	1	C	T	G	C	G	N	N	N	N
N	N	N	N	N	N	N	N	N	N	N	N
1	T	1	C	T	G	C	G	T	G	C	T
N	N	N	N	N	N	N	N	N	N	N	N
1	T	1	C	T	G	C	G	T	G	C	T
N	N	N	N	N	N	N	N	N	N	N	N
N	N	N	N	N	N	N	N	N	N	N	N
N	N	N	N	N	N	N	N	N	N	N	N
0	T	1	C	T	G	C	G	T	G	C	T
0	T	1	C	T	G	C	G	T	G	C	T
0	T	1	C	T	G	C	G	T	G	C	T
N	N	N	N	N	N	N	N	N	N	N	N
0	T	1	C	T	G	C	G	T	G	C	T
0	T	0	C	T	G	C	G	T	G	C	T
1	T	1	T	T	T	N	N	N	N	N	N
0	N	N	N	N	N	N	N	N	N	N	N
1	T	1	T	T	T	C	G	T	G	G	T
N	N	N	N	N	N	N	N	N	N	N	N
N	N	N	N	N	N	N	N	N	N	N	N
1	T	1	T	T	T	C	G	T	G	N	N
N	N	N	N	N	N	N	N	N	N	N	N
0	T	1	C	T	G	C	G	T	G	C	T
N	N	N	N	N	N	N	N	N	N	N	N
N	N	N	N	N	N	N	N	N	N	N	N
0	T	1	C	T	G	C	G	T	G	C	T
N	N	N	N	N	N	N	N	N	N	N	N
N	N	N	N	N	N	N	N	N	N	N	N
N	N	N	N	N	N	N	N	N	N	N	N
0	T	1	C	T	G	C	G	T	G	C	T
N	N	N	N	N	N	N	N	N	N	N	N
N	N	N	N	N	N	N	N	N	N	N	N
N	N	N	N	N	N	N	N	N	N	N	N
0	A	2	C	G	G	T	G	C	G	T	C
N	N	N	N	N	N	N	N	N	N	N	N
0	T	1	C	T	G	C	G	T	G	C	T
0	C	2	C	A	G	T	G	C	G	T	A
N	N	N	N	N	N	N	N	N	N	N	N
1	T	1	T	T	T	C	G	N	N	N	N

1	T	1	T	T	T	C	G	C	G	G	T
N	N	N	N	N	N	N	N	N	N	N	N
0	T	1	C	T	G	C	G	T	G	C	T
1	T	0	C	T	G	C	G	T	G	C	T
1	T	1	T	T	T	C	G	C	G	G	T
0	A	2	C	G	G	T	G	C	G	T	C
0	A	2	C	G	G	T	G	C	G	T	C
0	A	2	C	G	G	T	G	C	G	T	C
0	C	2	C	A	G	T	G	C	G	T	A
N	N	N	N	N	N	N	N	N	N	N	N
1	T	1	C	T	G	C	G	T	G	C	T
0	A	2	C	G	G	T	N	N	N	N	N
N	N	N	N	N	N	N	N	N	N	N	N
1	T	1	C	T	G	T	A	C	A	G	T
0	T	1	C	T	G	C	G	T	G	C	T
0	A	2	C	G	G	T	G	C	G	T	C
N	N	N	N	N	N	N	N	N	N	N	N
N	N	N	N	N	N	N	N	N	N	N	N
0	T	1	C	T	G	C	G	T	G	C	T
0	A	2	C	G	G	T	G	N	N	N	N
N	N	N	N	N	N	N	N	N	N	N	N
0	A	2	C	G	G	T	G	C	G	T	C
0	A	2	C	G	G	T	G	C	G	T	C
1	T	1	C	T	G	T	A	C	A	G	T
0	T	1	C	T	G	C	G	T	G	C	T
1	T	1	C	T	G	C	G	C	G	C	T
0	T	1	C	T	G	C	G	T	G	N	N
0	T	1	C	T	G	C	G	T	G	C	T
0	T	1	C	T	G	C	G	T	G	C	T
1	T	1	C	T	G	C	G	C	G	C	T
0	T	1	T	T	G	C	G	C	G	C	T
0	A	2	C	G	G	T	G	C	G	T	C
0	A	2	C	G	G	T	G	C	G	T	C
N	N	N	N	N	N	N	N	N	N	N	N
0	A	2	C	G	G	T	G	C	G	T	C
0	T	1	C	T	T	C	G	C	A	C	T
0	T	1	C	T	G	C	G	T	G	C	T
0	T	1	C	T	G	C	G	T	G	C	T
N	N	N	N	N	N	N	N	N	N	N	N
0	T	1	C	T	G	C	G	T	G	C	T
N	N	N	N	N	N	N	N	N	N	N	N
N	N	N	N	N	N	N	N	N	N	N	N
N	N	N	N	N	N	N	N	N	N	N	N
N	N	N	N	N	N	N	N	N	N	N	N
0	A	2	C	G	G	T	G	C	G	N	N
1	T	1	C	T	G	T	A	C	A	G	T
0	A	2	C	G	G	T	G	C	G	T	C
0	A	2	C	G	G	T	G	C	G	T	C
1	T	0	C	T	G	C	G	T	G	C	T
N	N	N	N	N	N	N	N	N	N	N	N
0	T	1	C	T	G	C	G	T	G	C	T
1	T	1	C	T	G	C	G	C	G	C	T
1	T	1	T	T	T	C	G	C	G	G	T
1	T	1	T	T	T	C	G	C	G	G	T
0	T	1	C	T	T	C	G	C	A	C	T
1	T	1	C	T	G	T	A	C	A	G	T
N	N	N	N	N	N	N	N	N	N	N	N

N	N	N	N	N	N	N	N	N	N	N	N
1	T	1	C	T	G	T	A	C	A	G	T
1	T	1	C	T	G	T	A	C	A	G	T
1	T	1	T	T	T	C	G	C	G	G	T
0	T	0	N	N	N	N	N	N	N	N	N
0	T	1	C	T	G	C	G	T	G	C	T
N	N	N	N	N	N	N	N	N	N	N	N
1	T	1	C	T	G	C	G	C	G	C	T
0	T	1	C	T	G	C	G	T	G	C	T
1	T	1	T	T	T	C	G	C	G	G	T
N	N	N	N	N	N	N	N	N	N	N	N
N	N	N	N	N	N	N	N	N	N	N	N
0	A	2	C	G	G	T	G	C	G	T	C
0	C	2	C	A	G	T	G	C	G	T	A
1	T	1	T	T	T	C	G	C	G	G	T
N	N	N	N	N	N	N	N	N	N	N	N
N	N	N	N	N	N	N	N	N	N	N	N
1	T	1	C	T	G	T	A	C	A	G	T
1	T	1	T	T	T	C	G	C	G	G	T
N	N	N	N	N	N	N	N	N	N	N	N
0	T	1	C	T	T	C	G	C	A	C	T
N	N	N	N	N	N	N	N	N	N	N	N
0	T	1	C	T	T	C	G	C	A	C	T
0	T	1	N	N	N	N	N	N	N	N	N
N	N	N	N	N	N	N	N	N	N	N	N
1	T	1	C	T	G	T	A	C	A	G	T
0	T	1	C	T	G	C	G	T	G	C	T
0	T	0	C	T	G	C	G	T	G	C	T
N	N	N	N	N	N	N	N	N	N	N	N
N	N	N	N	N	N	N	N	N	N	N	N
0	T	1	C	T	G	C	G	T	G	C	T
0	T	1	C	T	G	C	G	T	G	C	T
1	T	1	C	T	G	T	A	C	A	G	T
1	T	1	T	T	T	C	G	C	G	G	T
1	T	1	T	T	T	C	G	C	G	G	T
1	T	1	C	T	G	T	A	C	A	G	T
0	T	1	C	T	G	C	G	T	G	C	T
0	T	1	C	T	G	C	G	T	G	C	T
1	T	1	T	T	T	C	G	C	G	G	T
1	T	1	C	T	G	C	G	N	N	N	N
N	N	N	N	N	N	N	N	N	N	N	N
0	A	2	C	G	G	T	G	C	G	T	C
N	N	N	N	N	N	N	N	N	N	N	N
N	N	N	N	N	N	N	N	N	N	N	N
1	T	1	T	T	T	C	G	C	G	G	T
0	T	1	C	T	G	C	G	T	G	C	T
N	N	N	N	N	N	N	N	N	N	N	N
1	T	1	C	T	G	C	G	T	G	C	T
0	T	1	C	T	G	C	G	T	G	C	T
0	A	2	C	G	G	T	G	C	G	T	C
0	A	2	C	G	G	T	G	C	G	T	C
0	T	1	C	T	T	C	G	C	A	C	T
0	T	0	C	T	G	C	G	T	G	C	T
0	T	0	C	T	G	C	G	T	G	C	T
1	T	1	C	T	G	T	A	C	A	G	T
1	T	1	C	T	G	T	A	C	A	G	T
0	T	1	C	T	G	C	G	T	G	C	T

1	T	1	C	T	G	T	A	C	A	G	T
0	A	2	C	N	N	N	N	N	N	N	N
1	T	1	T	T	T	C	G	C	G	G	T
N	N	N	N	N	N	N	N	N	N	N	N
1	T	1	T	T	T	C	G	C	G	G	T
1	T	1	T	T	T	C	G	C	G	G	T
1	T	1	C	T	G	C	G	C	G	C	T
1	T	1	C	T	G	T	A	C	A	G	T
N	N	N	N	N	N	N	N	N	N	N	N
N	N	N	N	N	N	N	N	N	N	N	N
1	T	1	T	T	T	C	G	C	G	G	T
N	N	N	N	N	N	N	N	N	N	N	N
1	T	1	T	T	T	C	G	C	G	G	T
0	A	2	C	G	G	T	G	C	G	T	C
0	A	2	C	G	G	T	G	C	G	T	C
0	A	2	C	G	G	T	G	C	G	T	C
N	N	N	N	N	N	N	N	N	N	N	N
N	N	N	N	N	N	N	N	N	N	N	N
0	T	1	N	N	N	N	N	N	N	N	N
N	N	N	N	N	N	N	N	N	N	N	N
1	T	1	C	T	G	C	G	C	G	C	T
1	T	1	T	T	T	C	N	N	N	N	N
N	N	N	N	N	N	N	N	N	N	N	N
0	T	1	C	T	G	C	G	T	G	C	T
0	T	1	C	T	G	C	G	T	G	C	T
N	N	N	N	N	N	N	N	N	N	N	N
N	N	N	N	N	N	N	N	N	N	N	N
N	N	N	N	N	N	N	N	N	N	N	N
N	N	N	N	N	N	N	N	N	N	N	N
N	N	N	N	N	N	N	N	N	N	N	N
N	N	N	N	N	N	N	N	N	N	N	N
0	A	2	C	N	N	N	N	N	N	N	N
N	N	N	N	N	N	N	N	N	N	N	N
0	T	1	C	T	G	C	G	T	G	C	T
1	T	1	T	T	T	C	G	T	G	G	T
N	N	N	N	N	N	N	N	N	N	N	N
1	T	1	T	T	T	C	G	C	G	G	T
1	T	1	T	T	T	C	G	C	G	G	T
N	N	N	N	N	N	N	N	N	N	N	N
1	T	1	T	T	T	C	G	C	G	G	T
1	T	1	T	T	T	C	G	C	G	G	T
0	A	2	C	G	G	T	G	C	G	T	C
N	N	N	N	N	N	N	N	N	N	N	N
1	T	1	T	T	T	C	G	C	G	G	T
0	A	2	C	G	G	T	G	C	G	T	C
1	T	1	T	T	T	C	G	C	G	G	T
N	N	N	N	N	N	N	N	N	N	N	N
1	T	1	T	T	T	C	G	C	G	G	T
N	N	N	N	N	N	N	N	N	N	N	N
0	A	2	C	G	G	T	G	C	G	T	C
0	A	2	C	G	G	T	G	C	G	T	C
0	A	2	C	G	G	T	G	C	G	T	C
1	T	1	T	T	T	C	G	C	G	G	T
0	A	2	C	G	G	T	G	C	G	T	C
1	T	1	T	T	T	C	G	C	G	G	T
1	T	1	T	T	T	C	G	C	G	G	T

AI9_1	AI9_2	ASL1	ASL2	ASL3	ASL4	ASL5	ASL6	ASL7	ASL8	ASL9	ASL10	ASL11	ASL12	ASL13
A	G	C	G	3	9	C	C	G	1	G	8	G	1	A
A	G	C	G	3	9	C	C	G	1	G	8	G	1	A
A	G	C	T	0	0	G	G	C	0	A	8	C	2	A
G	A	C	G	3	9	C	C	G	1	G	8	G	1	A
G	A	N	N	N	N	N	N	N	N	N	N	N	N	N
A	G	C	T	N	0	C	G	C	1	A	8	G	2	A
A	G	T	T	3	9	C	C	G	1	G	0	G	2	T
A	G	C	G	3	9	C	C	G	1	G	8	G	1	A
A	G	C	T	N	0	G	G	C	0	A	8	C	2	A
A	G	C	T	0	9	G	G	C	0	A	8	C	2	A
G	A	C	G	3	9	C	C	G	1	G	8	G	1	A
A	G	C	T	0	0	G	G	C	0	A	8	C	2	A
A	G	T	T	0	0	G	G	C	0	A	8	G	1	A
A	G	C	G	3	9	C	C	G	1	G	8	G	1	A
A	G	T	T	3	9	C	C	G	1	G	0	G	2	T
N	G	T	T	3	9	C	C	G	1	G	0	G	2	T
N	N	T	T	3	9	C	C	G	1	G	0	G	2	T
A	G	N	N	3	9	C	C	G	1	G	0	G	2	T
N	N	C	T	0	9	G	G	C	0	A	8	C	2	A
A	G	C	T	0	0	G	G	C	0	A	8	C	2	A
G	A	C	T	0	9	G	G	C	0	A	8	C	2	A
A	G	C	G	3	9	C	C	G	1	G	8	G	1	A
A	G	C	G	3	9	C	C	G	1	G	8	G	1	A
A	G	C	G	3	9	C	C	G	1	G	8	G	1	A
A	G	C	G	3	9	C	C	G	1	G	8	G	1	A
A	G	C	G	3	9	C	C	G	1	G	8	G	1	A
G	A	C	G	3	9	C	C	G	1	G	8	G	1	A
A	G	C	G	3	9	C	C	G	1	G	8	G	1	A
G	A	N	N	N	N	N	N	N	N	N	N	N	N	N
N	N	C	G	3	9	C	C	G	1	G	8	G	1	A
A	G	C	T	0	9	G	G	C	0	A	8	C	2	A
A	G	N	N	N	N	N	N	N	N	N	N	N	N	N
A	G	N	N	0	0	G	G	C	0	A	8	C	2	A
N	N	N	N	N	N	N	N	N	N	N	N	N	N	N
A	G	C	G	3	9	C	C	G	1	G	8	G	1	A
A	G	C	T	0	9	G	G	C	0	A	8	C	2	A
G	A	N	N	N	9	G	G	C	0	A	8	C	2	A
A	G	C	G	3	9	C	C	G	1	G	8	G	1	A
A	G	N	N	N	N	N	N	N	N	N	N	G	2	T
N	N	N	N	N	N	N	N	N	N	N	N	N	N	N
A	G	C	G	3	9	C	C	G	1	G	8	G	1	A
A	G	C	G	3	9	C	C	G	1	G	8	G	1	A
A	G	C	T	0	0	G	G	C	0	A	8	C	2	A
A	G	C	G	3	9	C	C	G	1	G	8	G	1	A
A	G	C	T	0	0	G	G	C	0	A	8	C	2	A
A	G	C	T	0	0	G	G	C	0	A	8	C	2	A
A	G	C	G	3	9	C	C	G	1	G	8	G	1	A
A	G	C	T	0	9	G	G	C	0	A	8	C	2	A
A	G	C	G	3	9	C	C	G	1	G	8	G	1	A
A	G	C	G	3	9	C	C	G	1	G	8	G	1	A
G	A	C	G	3	9	C	C	G	1	G	8	G	1	A
A	G	C	G	3	9	C	C	G	1	G	8	G	1	A
N	N	T	T	0	0	G	G	C	0	A	8	G	1	A
A	G	C	G	3	9	C	C	G	1	G	8	G	1	A
A	G	-	T	0	0	G	G	C	0	A	8	G	1	A
A	G	N	N	N	0	G	G	C	0	A	8	C	2	A

A	G	C	T	0	0	G	G	C	0	A	8	C	2	A
G	A	C	T	0	9	G	G	C	0	A	8	C	2	A
A	G	N	N	N	N	N	N	N	N	N	N	N	N	N
A	G	C	T	0	0	G	G	C	0	A	8	C	2	A
A	G	C	T	0	9	G	G	C	0	A	8	C	2	A
A	G	C	G	3	9	C	C	G	1	G	8	G	1	A
A	G	C	G	3	9	C	C	G	1	G	8	G	1	A
A	G	C	T	0	9	G	G	C	0	A	8	C	2	A
A	G	C	T	0	9	G	G	C	0	A	8	C	2	A
N	N	C	G	3	9	C	C	G	1	G	8	G	1	A
A	G	N	N	N	9	G	G	C	0	A	8	C	2	A
A	G	C	T	0	9	G	G	C	0	A	8	C	2	A
A	G	C	T	0	9	G	G	C	0	A	8	C	2	A
A	G	C	G	3	9	C	-	-	1	G	8	G	1	A
A	G	N	N	N	N	N	N	N	N	N	N	N	N	N
A	G	T	T	3	9	C	C	G	1	G	0	G	2	T
A	G	N	N	N	N	N	N	N	N	N	N	N	N	N
G	A	N	N	0	0	G	G	C	0	A	8	C	2	A
A	G	C	T	0	0	G	G	C	0	A	8	C	2	A
N	N	N	N	N	0	G	G	C	0	A	8	C	2	A
A	G	T	T	0	0	G	G	C	0	A	8	G	1	A

A	G	C	G	3	9	C	C	G	1	G	8	G	1	A
A	G	C	G	3	9	C	C	G	1	G	8	G	1	A
A	G	T	T	0	0	G	G	C	0	A	8	G	1	A
A	G	C	T	0	9	G	G	C	0	A	8	C	2	A
A	G	T	T	3	9	C	C	G	1	G	0	G	2	T
A	G	C	T	0	9	G	G	C	0	A	8	C	2	A
A	G	T	T	3	9	C	C	G	1	G	0	G	2	T
A	G	C	G	3	9	C	C	G	1	G	8	G	1	A
A	G	N	N	N	N	N	N	N	N	N	N	N	N	N
A	G	C	G	3	9	C	C	G	1	G	8	G	1	A
N	N	N	N	3	9	C	C	G	1	G	8	G	1	A
A	G	N	N	N	N	N	N	N	N	N	N	N	N	N
A	G	N	N	N	N	N	N	N	N	N	N	N	N	N
A	G	T	T	3	9	C	C	G	1	G	0	G	2	T
A	G	C	G	3	9	C	C	G	1	G	8	G	1	A
A	G	C	G	3	9	C	C	G	1	G	8	G	1	A
A	G	T	T	3	9	C	C	G	1	G	0	G	2	T
A	G	C	T	0	0	G	G	C	0	A	8	C	2	A
A	G	C	G	3	9	C	C	G	1	G	8	G	1	A
A	G	N	N	3	9	C	C	G	1	G	8	G	1	A
A	G	C	G	3	0	G	G	C	0	A	8	C	2	A
A	G	N	N	3	0	C	G	C	0	A	8	C	2	A
A	G	N	N	N	N	N	N	N	N	N	N	N	N	N
A	G	C	G	3	9	C	C	G	1	G	8	G	1	A
A	G	C	G	3	9	C	C	G	1	G	8	G	1	A
A	G	N	N	N	N	N	N	N	N	N	N	C	2	A
A	G	C	T	0	9	G	G	C	0	A	8	C	2	A
A	G	C	G	3	9	C	C	G	1	G	0	G	2	T
A	G	C	T	0	9	G	G	C	0	A	8	C	2	A
A	G	C	T	0	9	G	G	C	0	A	8	C	2	A
G	A	C	T	0	0	G	G	C	0	A	8	C	2	A
G	A	C	T	0	0	G	G	C	0	A	8	C	2	A
A	G	C	G	3	9	C	C	G	1	G	8	G	1	A
A	G	C	G	3	9	C	C	G	1	G	8	G	1	A
G	A	T	T	3	9	C	C	G	1	G	0	G	2	T

N	N	N	N	N	N	N	N	N	N	N	N	N	N	N
N	N	N	N	N	N	N	N	N	N	N	N	N	N	N
A	G	N	N	N	N	N	N	N	N	N	N	N	N	N
G	A	C	T	0	9	G	G	C	0	A	8	C	2	A
A	G	C	G	3	9	C	C	G	1	G	8	G	1	A
G	A	N	N	N	9	G	G	C	0	A	8	C	2	A
A	G	N	N	0	0	G	G	C	0	A	8	C	2	A
A	G	C	T	0	9	G	G	C	0	A	8	C	2	A
A	G	C	T	0	9	G	G	C	0	A	8	C	2	A
A	G	N	N	N	N	N	N	N	N	N	N	G	1	A
A	G	T	T	3	9	C	C	G	1	G	0	G	2	T
A	G	T	T	3	9	C	C	G	1	G	0	G	2	T
A	G	T	T	0	0	G	G	C	0	A	8	G	1	A
A	G	C	G	3	9	C	C	G	1	G	8	G	1	A
A	G	N	N	N	N	C	C	G	1	G	0	G	2	T
A	G	N	N	N	N	N	N	N	N	N	N	N	N	N
A	G	N	N	N	N	N	N	N	N	N	N	N	N	N
A	G	T	T	3	9	C	C	G	1	G	8	G	1	A
G	A	C	G	3	9	C	C	G	1	G	8	G	1	A
A	G	N	N	N	N	N	N	N	N	N	N	N	N	N
A	G	N	N	3	9	G	G	C	0	A	8	C	2	A
A	G	T	T	3	9	C	C	G	1	G	0	G	2	T
N	N	C	G	3	9	C	C	G	1	G	8	G	1	A
A	G	C	T	0	9	G	G	C	0	A	8	C	2	A
A	G	C	G	3	9	C	C	G	1	G	8	G	1	A
G	A	C	G	3	9	C	C	G	1	G	8	G	1	A
N	N	C	G	3	9	C	C	G	1	G	8	G	1	A
N	N	C	G	3	9	C	C	G	1	G	8	G	1	A
A	G	C	T	0	9	G	G	C	0	A	8	C	2	A
A	G	C	G	3	9	C	C	G	1	G	8	G	1	A
A	G	C	G	3	9	C	C	G	1	G	8	G	1	A
A	G	C	T	0	9	G	G	C	0	A	8	C	2	A
A	G	C	G	3	9	C	C	G	1	G	8	G	1	A
A	G	N	N	3	9	G	G	C	0	A	8	C	2	A
A	G	C	T	0	9	G	G	C	0	A	8	C	2	A
A	G	C	G	3	9	C	C	G	1	G	8	G	0	A
A	G	N	N	3	9	G	G	C	0	A	8	C	2	A
A	G	C	T	0	9	G	G	C	0	A	8	G	2	T
A	G	N	N	N	N	N	N	N	N	N	N	N	N	N
A	G	C	G	3	9	C	C	G	1	G	8	G	0	A
A	G	C	G	3	9	C	C	G	1	G	8	G	1	A
A	G	C	G	3	9	C	C	G	1	G	8	G	1	A
A	G	N	N	N	9	G	G	C	0	A	8	C	2	A
A	G	C	T	0	0	G	G	C	0	A	8	C	2	A
G	A	C	T	0	9	G	G	C	0	A	8	C	2	A
A	G	N	N	N	N	N	N	N	N	N	N	N	N	N
G	A	T	T	3	9	C	C	G	1	G	0	G	2	T
A	G	C	G	3	9	C	C	G	1	G	8	G	1	A
A	G	C	T	0	0	G	G	C	0	A	8	C	1	A
A	G	C	G	3	9	C	C	G	1	G	8	G	0	A
A	G	N	N	3	9	C	C	G	1	G	8	G	1	A
A	G	C	G	3	9	C	C	G	1	G	8	G	0	A
A	G	C	T	0	0	G	G	C	0	A	8	C	2	A
N	N	C	T	0	0	G	G	C	0	A	8	C	2	A
A	G	N	N	N	9	G	G	C	0	A	8	C	2	A
A	G	N	N	N	N	N	N	N	N	N	N	N	N	N
A	G	C	G	3	9	C	C	G	1	G	8	G	1	A

A	G	C	G	3	9	C	C	G	1	G	8	G	1	A
A	G	C	T	0	0	G	G	C	0	A	8	C	2	A
A	G	C	G	0	9	G	G	C	0	A	8	C	2	A
A	G	C	G	3	9	C	C	G	1	G	8	G	1	A
A	G	C	G	3	9	C	C	G	1	G	8	G	1	A
A	G	N	N	N	N	N	N	N	N	N	N	N	N	N
A	G	C	T	0	0	G	G	C	0	A	8	C	1	A
G	A	C	T	0	0	G	G	C	0	A	8	C	2	A
A	G	C	G	3	9	C	C	G	1	G	8	G	1	A
A	G	T	T	3	9	C	C	G	1	G	0	G	2	T
G	A	C	T	0	0	G	G	C	0	A	8	C	2	A
G	A	C	T	0	9	G	G	C	0	A	8	C	2	A
A	G	C	T	0	9	G	G	C	0	A	8	C	2	A
A	G	C	T	0	9	G	G	C	0	A	8	C	2	A
A	G	C	G	3	9	C	C	G	1	G	8	G	1	A
G	A	N	N	N	9	G	G	C	0	A	8	C	2	A
A	G	T	T	3	9	C	C	G	1	G	0	G	2	T
A	G	T	T	3	9	C	C	G	1	G	0	G	2	T
A	G	T	T	3	9	C	C	G	1	G	0	G	2	T
A	G	C	T	0	9	G	G	C	0	A	8	G	1	A
A	G	C	T	0	9	G	G	C	0	A	8	C	2	A
G	A	C	T	0	9	G	G	C	0	A	8	G	1	A
N	N	C	T	0	9	G	G	C	0	A	8	C	2	A
A	G	C	G	3	9	C	C	G	1	G	8	G	1	A
A	G	T	T	0	0	G	G	C	0	A	8	G	1	A
A	G	T	T	0	0	G	G	C	0	A	8	G	1	A
A	G	T	T	3	9	C	C	G	1	G	8	G	1	A
A	G	C	G	3	9	C	C	G	1	G	8	G	1	A
A	G	C	T	0	9	G	G	C	0	A	8	C	2	A
A	G	C	G	3	9	C	C	G	1	G	8	G	1	A
A	G	C	G	3	9	C	C	G	1	G	8	G	1	A
A	G	C	G	3	9	C	C	G	1	G	8	G	1	A
A	G	C	G	3	9	C	C	G	1	G	8	G	1	A
G	A	C	T	0	0	G	G	C	0	A	8	C	2	A
G	A	C	T	0	9	G	G	C	0	A	8	C	2	A
A	G	C	G	3	9	C	C	G	1	G	8	G	1	A
N	N	N	N	N	N	N	N	N	N	N	N	N	N	N
A	G	N	N	N	N	N	N	N	N	N	N	N	N	N
G	A	C	T	0	9	G	G	C	0	A	8	C	2	A
A	G	C	G	3	9	C	C	G	1	G	8	G	1	A
A	G	C	G	3	9	C	C	G	1	G	8	G	1	A
A	G	C	G	3	9	C	C	G	1	G	8	G	1	A
A	G	N	N	N	N	N	N	N	N	N	N	G	1	A
A	G	N	N	3	9	C	C	G	1	G	0	G	2	T
A	G	T	T	0	0	G	G	C	0	A	8	G	1	A
G	A	T	T	3	9	C	C	G	1	G	8	G	1	A
A	G	C	G	3	9	C	C	G	1	G	8	G	0	A
A	G	C	G	3	9	C	C	G	1	G	8	G	1	A
A	G	C	G	3	9	C	C	G	1	G	8	G	1	A
A	G	C	T	0	9	G	G	C	0	A	8	C	2	A
A	G	C	T	0	9	G	G	C	0	A	8	C	1	A
G	A	T	T	3	9	C	C	G	1	G	0	G	2	T
G	A	C	T	0	9	G	G	C	0	A	8	C	2	A
G	A	C	T	0	9	G	G	C	0	A	8	C	2	A
A	G	C	T	0	9	G	G	C	0	A	8	C	2	A
A	G	C	G	3	9	C	C	G	1	G	8	G	1	A
G	A	C	G	3	9	C	C	G	1	G	8	G	1	A

A	G	C	G	3	9	C	C	G	1	G	8	G	1	A
A	G	C	G	3	9	C	C	G	1	G	8	G	0	A
A	G	C	G	3	9	C	C	G	1	G	8	G	1	A
A	G	C	G	3	9	C	C	G	1	G	8	G	1	A
N	N	C	T	0	0	G	G	C	0	A	8	C	1	A
G	A	N	N	0	0	G	G	C	0	A	8	C	2	A
A	G	C	G	3	9	C	C	G	1	G	8	G	0	A
A	G	C	G	3	9	C	C	G	1	G	8	G	1	A
A	G	C	T	0	9	G	G	C	0	A	8	C	2	A
A	G	C	T	0	0	G	G	C	0	A	8	C	2	A
A	G	C	T	0	0	G	G	C	0	A	8	C	2	A
A	G	C	T	0	9	G	G	C	0	A	8	C	2	A
A	G	C	T	0	9	G	G	C	0	A	8	G	1	A
G	A	T	T	3	9	C	C	G	1	G	0	G	2	T
A	G	C	T	0	0	G	G	C	0	A	8	C	2	A
A	G	C	G	3	9	C	C	G	1	G	8	G	1	A
G	A	C	G	3	9	C	C	G	1	G	8	G	1	A
G	A	C	T	0	9	G	G	C	0	A	8	C	2	A
A	G	C	T	0	9	G	G	C	0	A	8	C	2	A
A	G	C	T	0	9	G	G	C	0	A	8	G	1	A
G	A	N	T	0	9	G	G	C	0	A	8	G	1	A
A	G	C	G	3	9	C	C	G	1	G	8	G	1	A
A	G	C	G	3	9	C	C	G	1	G	8	G	1	A
G	A	C	T	0	9	G	G	C	0	A	8	G	1	A
A	G	C	T	0	0	G	G	C	0	A	8	C	2	A
A	G	T	T	3	9	C	C	G	1	G	0	G	2	T
A	G	C	G	3	9	C	C	G	1	G	8	G	1	A
A	G	C	T	0	9	G	G	C	0	A	8	C	2	A
A	G	T	T	3	9	C	C	G	1	G	0	G	2	T
A	G	C	G	3	9	C	C	G	1	G	8	G	1	A
A	G	C	G	3	0	G	G	C	0	A	8	C	2	A
A	G	C	G	3	9	C	C	G	1	G	8	G	1	A
A	G	T	T	3	9	C	C	G	1	G	0	G	2	T
A	G	T	T	0	0	G	G	C	0	A	8	G	1	A
A	G	C	G	3	9	C	C	G	1	G	8	G	1	A
N	N	C	G	3	9	C	C	G	1	G	8	G	1	A
A	G	N	N	0	0	G	G	C	0	A	8	C	2	A
A	G	C	G	3	9	C	C	G	1	G	8	G	1	A
A	G	N	N	3	9	C	C	G	1	G	8	G	1	A
A	G	C	G	3	9	C	C	G	1	G	8	G	1	A
A	G	C	T	0	0	G	G	C	0	A	8	C	2	A
N	N	N	N	N	N	N	N	N	N	N	N	N	N	N
A	G	C	G	3	9	C	C	G	1	G	8	G	1	A
A	G	C	G	3	9	C	C	G	1	G	8	G	1	A
A	G	C	G	3	9	C	C	G	1	G	8	G	1	A
A	G	C	T	0	0	G	G	C	0	A	8	C	2	A
A	G	C	T	0	0	G	G	C	0	A	8	C	2	A
A	G	C	T	0	9	G	G	C	0	A	N	C	2	A
A	G	N	N	N	N	N	N	N	N	N	N	N	N	N
A	G	C	T	0	9	G	G	C	0	A	N	C	2	A
A	G	C	T	0	9	G	G	C	0	A	N	C	2	A
N	N	N	N	N	N	N	N	N	N	N	N	N	N	N
A	G	C	G	3	9	C	C	G	1	G	8	G	1	A
A	G	C	G	3	9	C	C	G	1	G	8	G	1	A
A	G	C	G	3	9	C	C	G	1	G	8	G	1	A
N	N	N	N	N	N	N	N	N	N	N	N	N	N	N

ASL14	ASL15	ASL16	ASL17	ASL18	ASL19	ASL20	ASL21	ASL22	ASL23	ASL24	ASL25	ASL26
5	8	4	4	T	C	C	G	G	T	C	A	121
5	8	0	0	T	C	C	G	G	T	C	A	121
0	0	4	4	A	A	T	A	C	G	G	A	0
5	8	0	0	T	C	C	G	G	T	C	A	121
N	N	N	N	N	N	N	N	N	N	N	N	N
0	0	4	0	T	C	C	G	G	T	C	A	N
5	0	0	0	T	C	C	G	G	T	C	A	N
5	8	0	0	T	C	C	G	G	T	C	A	121
0	0	4	4	A	A	T	A	C	G	G	A	N
0	0	4	4	A	A	T	A	C	G	G	A	0
5	8	0	0	T	C	C	G	G	T	C	A	121
0	0	4	4	A	A	T	A	C	G	G	A	0
5	0	0	0	T	C	C	G	G	T	C	C	121
5	8	0	0	T	C	C	G	G	T	C	A	121
5	0	0	0	T	C	C	G	G	T	C	A	N
5	0	0	0	T	C	C	G	G	T	C	A	N
5	0	0	0	T	C	C	G	G	T	C	A	N
5	0	0	0	T	C	C	G	G	T	C	A	N
0	0	4	4	A	A	T	A	C	G	G	A	0
0	0	4	4	A	A	T	A	C	G	G	A	0
0	0	4	4	A	A	T	A	C	G	G	A	0
5	8	0	0	T	C	C	G	G	T	C	A	121
5	8	0	0	T	C	C	G	G	T	C	A	121
5	8	0	0	T	C	C	G	G	T	C	A	121
5	8	0	0	T	C	C	G	G	T	C	A	121
5	8	0	0	T	C	C	G	G	T	C	A	121
5	8	0	0	T	C	C	G	G	T	C	A	121
5	8	0	0	T	C	C	G	G	T	C	A	121
N	N	N	N	N	N	N	N	N	N	N	N	121
5	8	0	0	T	C	C	G	G	T	C	A	121
0	0	4	4	A	A	T	A	C	G	G	A	N
N	N	N	N	N	N	N	N	N	N	N	N	N
0	0	4	4	A	A	T	A	C	G	G	A	0
N	N	N	N	N	N	N	N	N	N	N	N	N
5	8	0	0	T	C	C	G	G	T	C	A	121
0	0	4	4	A	A	T	A	C	G	G	A	N
0	0	4	4	A	A	T	A	C	G	G	A	0
5	0	0	0	T	C	C	G	G	T	C	A	121
5	0	0	0	T	C	C	G	G	T	C	A	N
N	N	N	N	N	N	N	N	N	N	N	N	N
5	8	0	0	T	C	C	G	G	T	C	A	121
5	8	0	0	T	C	C	G	G	T	C	A	121
0	0	4	4	A	A	T	A	C	G	G	A	0
5	8	0	0	T	C	C	G	G	T	C	A	121
0	0	4	4	A	A	T	A	C	G	G	A	0
0	0	4	4	A	A	T	A	C	G	G	A	N
5	8	0	0	T	C	C	G	G	T	C	A	121
0	0	4	4	A	A	T	A	C	G	G	A	0
5	8	0	0	T	C	C	G	G	T	C	A	121
5	8	0	0	T	C	C	G	G	T	C	A	121
5	0	0	0	T	C	C	G	G	T	C	A	121
5	8	0	0	T	C	C	G	G	T	C	A	121
5	0	0	0	T	C	C	G	G	T	C	C	121
5	8	0	0	T	C	C	G	G	T	C	A	121
5	0	0	0	T	C	C	G	G	T	C	C	121
0	0	4	4	A	A	T	A	C	G	G	A	0

0	0	4	4	A	A	T	A	C	G	G	A	0
0	0	4	4	A	A	T	A	C	G	G	A	0
N	N	N	N	N	N	N	N	N	N	N	N	121
0	0	4	4	A	A	T	A	C	G	G	A	0
0	0	4	4	A	A	T	A	C	G	G	A	0
5	8	0	0	T	C	C	G	G	T	C	A	121
5	8	0	0	T	C	C	G	G	T	C	A	121
0	0	4	4	A	A	T	A	C	G	G	A	0
0	0	4	4	A	A	T	A	C	G	G	A	0
5	8	0	0	T	C	C	G	G	T	C	A	121
0	0	4	4	A	A	T	A	C	G	G	A	0
0	0	4	4	A	A	T	A	C	G	G	A	0
0	0	4	4	A	A	T	A	C	G	G	A	0
5	8	0	0	T	C	C	G	G	T	C	A	121
N	N	N	N	N	N	N	N	N	N	N	N	121
5	0	0	0	T	C	T	G	G	T	C	C	121
N	N	N	N	N	N	N	N	N	N	N	N	121
0	0	4	4	A	A	T	A	C	G	G	A	0
0	0	4	4	A	A	T	A	C	G	G	A	0
0	0	4	4	A	A	T	A	C	G	G	A	0
5	0	0	0	T	C	C	G	G	T	C	C	121

5	8	0	0	T	C	C	G	G	T	C	C	N
5	8	0	0	T	C	C	G	G	T	C	A	121
5	0	0	0	T	C	C	G	G	T	C	C	121
0	0	4	4	A	A	T	A	C	G	G	A	0
5	0	0	0	T	C	C	G	G	T	C	A	121
0	0	4	4	A	A	T	A	C	G	G	A	0
5	0	0	0	T	C	C	G	G	T	C	C	121
5	8	0	0	T	C	C	G	G	T	C	C	121
N	N	N	N	N	N	N	N	N	N	N	N	N
5	8	0	0	T	C	C	G	G	T	C	A	121
5	8	0	0	T	C	C	G	G	T	C	C	121
N	N	N	N	N	N	N	N	N	N	N	N	N
N	N	N	N	N	N	N	N	N	N	N	N	N
5	0	0	0	T	C	C	G	G	T	C	C	121
5	8	0	0	T	C	C	G	G	T	C	C	121
5	8	0	0	T	C	C	G	G	T	C	C	121
5	0	0	0	T	C	C	G	G	T	C	A	121
0	0	4	4	A	A	T	A	C	G	G	A	0
5	8	0	0	T	C	C	G	G	T	C	C	121
5	8	0	0	T	C	C	G	G	T	C	C	121
0	0	4	4	A	A	T	A	C	G	G	A	0
0	0	4	4	A	A	T	A	C	G	G	A	0
N	N	N	N	N	N	N	N	N	N	N	N	N
5	8	0	0	T	C	C	G	G	T	C	A	121
5	8	0	0	T	C	C	G	G	T	C	C	121
0	0	4	4	A	A	T	A	C	G	G	A	0
0	0	4	4	A	A	T	A	C	G	G	A	0
5	0	0	0	T	C	C	G	G	T	C	C	N
0	0	4	4	A	A	T	A	C	G	G	A	0
0	0	4	4	A	A	T	A	C	G	G	A	N
0	0	4	4	A	A	T	G	G	T	C	A	121
0	0	4	4	A	A	T	G	G	T	C	A	121
5	0	0	0	T	C	C	G	G	T	C	A	121
5	8	0	0	T	C	C	G	G	T	C	A	121
5	0	0	0	T	C	C	G	G	T	C	A	N

N	N	N	N	N	N	N	N	N	N	N	N	N
N	N	N	N	N	N	N	N	N	N	N	N	N
N	N	N	N	N	N	N	N	N	N	N	N	N
0	0	4	4	A	A	T	A	C	G	G	A	0
5	0	0	0	T	C	C	G	G	T	C	A	121
0	0	4	4	A	A	T	A	C	G	G	A	0
0	0	4	4	A	A	T	A	C	G	G	A	0
0	0	4	4	A	A	T	A	C	G	G	A	0
0	0	4	4	A	A	T	A	C	G	G	A	0
5	8	0	0	T	C	C	G	G	T	C	A	121
5	0	0	0	T	C	C	G	G	T	C	A	N
5	0	0	0	T	C	C	G	G	T	C	A	N
5	0	0	0	T	C	C	G	G	T	C	C	121
5	8	0	0	T	C	C	G	G	T	C	A	121
5	0	0	0	T	C	C	G	G	T	C	A	N
N	N	N	N	N	N	N	N	N	N	N	N	N
N	N	N	N	N	N	N	N	N	N	N	N	121
5	8	0	0	T	C	C	G	G	T	C	C	121
5	8	0	0	T	C	C	G	G	T	C	A	121
N	N	N	N	N	N	N	N	N	N	N	N	N
0	0	4	4	A	A	T	A	C	G	N	N	N
5	0	0	0	T	C	C	G	G	T	C	A	N
5	8	0	0	T	C	C	G	G	T	C	C	121
0	0	4	4	A	A	T	A	C	G	G	A	0
5	8	0	0	T	C	C	G	G	T	C	C	121
5	8	0	0	T	C	C	G	G	T	C	C	121
5	8	0	0	T	C	C	G	G	T	C	A	121
5	8	0	0	T	C	C	G	G	T	C	A	121
0	0	4	4	A	A	T	A	C	G	G	A	0
5	8	0	0	T	C	C	G	G	T	C	C	121
5	8	0	0	T	C	C	G	G	T	C	A	121
0	0	4	4	A	A	T	A	C	G	G	A	0
5	8	0	0	T	C	C	G	G	T	C	C	121
0	0	4	4	A	A	T	A	C	G	G	A	0
0	0	4	4	A	A	T	A	C	G	G	A	0
5	8	0	0	T	C	C	G	G	T	C	A	121
0	0	4	4	A	A	T	A	C	G	G	A	0
5	0	0	0	T	C	C	G	G	T	C	A	121
N	N	N	N	N	N	N	N	N	N	N	N	N
5	8	0	0	T	C	C	G	G	T	C	C	121
5	8	0	0	T	C	C	G	G	T	C	C	N
5	8	0	0	T	C	C	G	G	T	C	A	121
0	0	4	4	A	A	T	A	C	G	G	A	0
0	0	4	4	A	A	T	A	C	G	G	A	N
0	0	4	4	A	A	T	A	C	G	G	A	0
N	N	N	N	N	N	N	N	N	N	N	N	121
5	0	0	0	T	C	C	G	G	T	C	A	N
5	8	0	0	T	C	C	G	G	T	C	A	121
0	0	4	4	A	A	T	A	C	G	G	A	0
5	8	0	0	T	C	C	G	G	T	C	A	121
5	8	0	0	T	C	C	G	G	T	C	A	121
5	8	0	0	T	C	C	G	G	T	C	A	121
0	0	4	4	A	A	T	A	C	G	G	A	0
0	0	4	4	A	A	T	A	C	G	G	A	0
0	0	4	4	A	A	T	A	C	G	G	A	121
N	N	N	N	N	N	N	N	N	N	N	N	121
5	8	0	0	T	C	C	G	G	T	C	A	121

5	8	0	0	T	C	C	G	G	T	C	A	121
0	0	4	4	A	A	T	A	C	G	G	A	N
0	0	4	4	A	A	T	A	C	G	G	A	0
5	8	0	0	T	C	C	G	G	T	C	A	121
5	8	0	0	T	C	C	G	G	T	C	A	121
N	N	N	N	N	N	N	N	N	N	N	N	121
0	0	4	4	A	A	T	A	C	G	G	A	0
0	0	4	4	A	A	T	G	G	T	C	A	121
5	8	0	0	T	C	C	G	G	T	C	A	121
5	0	0	0	T	C	C	G	G	T	C	A	N
0	0	4	4	A	A	T	G	G	T	C	A	121
0	0	4	4	A	A	T	A	C	G	G	A	0
0	0	4	4	A	A	T	A	C	G	G	A	0
0	0	4	4	A	A	T	A	C	G	G	A	N
5	8	0	0	T	C	C	G	G	T	C	A	121
0	0	4	4	A	A	T	A	C	G	G	A	0
5	0	0	0	T	C	C	G	G	T	C	A	N
5	0	0	0	T	C	C	G	G	T	C	A	N
5	0	0	0	T	C	C	G	G	T	C	A	N
5	8	0	0	T	C	C	G	G	T	C	C	121
0	0	4	4	A	A	T	A	C	G	G	A	0
5	8	0	0	T	C	C	G	G	T	C	C	121
0	0	4	4	A	A	T	A	C	G	G	A	0
5	8	0	0	T	C	C	G	G	T	C	A	121
5	0	0	0	T	C	C	G	G	T	C	C	121
5	0	0	0	T	C	C	G	G	T	C	C	121
5	0	0	0	T	C	C	G	G	T	C	C	121
5	8	0	0	T	C	C	G	G	T	C	C	N
0	0	4	4	A	A	T	A	C	G	G	A	N
5	8	0	0	T	C	C	G	G	T	C	C	121
5	8	0	0	T	C	C	G	G	T	C	A	121
5	8	0	0	T	C	C	G	G	T	C	A	121
5	8	0	0	T	C	C	G	G	T	C	A	121
0	0	4	4	A	A	T	A	C	G	G	A	0
0	0	4	4	A	A	T	A	C	G	G	A	0
5	8	0	0	T	C	C	G	G	T	C	A	121
N	N	N	N	N	N	N	N	N	N	N	N	121
N	N	N	N	N	N	N	N	N	N	N	N	121
0	0	4	4	A	A	T	A	C	G	G	A	0
5	8	0	0	T	C	C	G	G	T	C	C	121
5	8	0	0	T	C	C	G	G	T	C	C	121
5	8	0	0	T	C	C	G	G	T	C	C	N
5	8	0	0	T	C	C	G	G	T	C	A	121
5	0	0	0	T	C	C	G	G	T	C	A	N
5	0	0	0	T	C	C	G	G	T	C	C	121
5	0	0	0	T	C	C	G	G	T	C	C	121
5	8	0	0	T	C	C	G	G	T	C	C	121
5	8	0	0	T	C	C	G	G	T	C	C	121
5	8	0	0	T	C	C	G	G	T	C	C	N
0	0	N	4	A	A	T	A	C	G	G	A	0
0	0	N	4	A	A	T	A	C	G	G	A	0
5	0	0	0	T	C	C	G	G	T	C	C	121
0	0	N	4	A	A	T	A	C	G	G	A	0
0	0	N	4	A	A	T	A	C	G	G	A	0
0	9	N	4	A	A	T	A	C	G	G	A	0
5	8	0	0	T	C	C	G	G	T	C	A	121
5	8	0	0	T	C	C	G	G	T	C	A	121

5	8	0	0	T	C	C	G	G	T	C	A	121
5	8	0	0	T	C	C	G	G	T	C	A	121
5	8	0	0	T	C	C	G	G	T	C	A	121
5	8	0	0	T	C	C	G	G	T	C	A	121
0	0	N	4	A	A	T	G	G	T	C	A	121
0	0	N	4	A	A	T	A	C	G	G	A	0
5	8	0	0	T	C	C	G	G	T	C	C	121
5	8	0	0	T	C	C	G	G	T	C	A	121
0	0	N	4	A	A	T	A	C	G	G	A	0
0	0	N	4	A	A	T	A	C	G	G	A	0
0	0	N	4	A	A	T	A	C	G	G	A	0
0	0	N	4	A	A	T	A	C	G	G	A	0
5	8	0	0	T	C	C	G	G	T	C	C	121
5	0	0	0	T	C	C	G	G	T	C	A	N
0	0	N	4	A	A	T	A	C	G	G	A	0
5	8	0	0	T	C	C	G	G	T	C	A	121
5	8	0	0	T	C	C	G	G	T	C	A	121
0	0	N	4	A	A	T	A	C	G	G	A	0
0	0	N	4	A	A	T	A	C	G	G	A	0
5	8	0	0	T	C	C	G	G	T	C	C	121
5	8	0	0	T	C	C	G	G	T	C	C	121
5	8	0	0	T	C	C	G	G	T	C	A	121
5	8	0	0	T	C	C	G	G	T	C	C	121
5	8	0	0	T	C	C	G	G	T	C	C	121
0	0	N	4	A	A	T	G	G	T	C	A	121
5	0	0	0	T	C	C	G	G	T	C	A	N
5	0	0	0	T	C	C	G	G	T	C	A	121
0	0	N	4	A	A	T	A	C	G	G	A	0
5	0	0	0	T	C	C	G	G	T	C	C	121
5	8	0	0	T	C	C	G	G	T	C	C	121
0	0	N	4	A	A	T	A	C	G	G	A	0
5	8	0	0	T	C	C	G	G	T	C	C	121
5	0	0	0	T	C	C	G	G	T	C	A	121
5	0	0	0	T	C	C	G	G	T	C	C	121
5	8	0	0	T	C	C	G	G	T	C	C	121
5	8	0	0	T	C	C	G	G	T	C	C	121
0	0	N	4	A	A	T	A	C	G	G	A	0
5	0	0	0	T	C	C	G	G	T	C	A	121
5	0	0	0	T	C	C	G	G	T	C	A	121
5	0	0	0	T	C	C	G	G	T	C	A	121
0	0	N	4	A	A	T	A	C	G	G	A	0
N	N	N	N	N	N	N	N	N	N	N	N	N
5	0	0	0	T	C	C	G	G	T	C	A	121
5	0	0	0	T	C	C	G	G	T	C	A	121
5	8	0	0	T	C	C	G	G	T	C	A	121
0	0	N	4	A	A	T	A	C	G	G	A	0
0	0	N	4	A	A	T	A	C	G	G	A	0
0	0	N	4	A	A	T	A	C	G	G	A	0
N	N	N	N	N	N	N	N	N	N	N	N	N
0	0	N	4	A	A	T	A	C	G	G	A	0
0	0	N	4	A	A	T	A	C	G	G	A	121
N	N	N	N	N	N	N	N	N	N	N	N	N
5	8	0	0	T	C	C	G	G	T	C	A	121
5	8	0	0	T	C	C	G	G	T	C	A	121
5	8	0	0	T	C	C	G	G	T	C	A	121
N	N	N	N	N	N	N	N	N	N	N	N	N

ASL27	ASL28	ASL29	ASL30	ASL31	ASL32	ASL33	ASL34	ASL35	ASL36	ASL37	ASL38	ASL39
T	C	C	4	C	C	3	N	N	8	N	1	1
T	C	C	4	C	C	N	N	N	N	N	N	N
C	A	T	0	N	N	N	N	N	N	N	N	N
T	C	C	4	C	C	3	C	G	8	G	0	0
N	N	C	4	C	C	3	C	G	8	G	0	0
N	N	N	N	N	N	3	C	G	8	A	0	0
N	N	N	N	N	N	3	C	G	8	G	0	1
T	C	C	4	C	C	3	C	G	8	G	0	0
C	A	C	4	C	C	3	C	G	8	G	0	1
C	A	T	4	T	G	N	N	N	N	N	N	N
T	C	C	4	C	C	3	C	G	8	G	0	0
C	A	T	0	T	G	0	T	G	8	G	0	1
T	C	C	4	C	C	3	C	G	8	G	0	1
T	C	C	4	C	C	3	C	A	11	-	N	0
N	N	N	N	N	N	3	C	G	8	G	0	1
N	N	N	N	N	N	3	C	G	8	G	0	1
N	N	N	N	N	N	N	N	N	N	N	N	N
N	N	N	N	N	N	3	C	G	8	G	0	1
C	A	T	4	T	G	3	C	G	8	G	0	1
C	A	T	0	N	N	3	C	G	8	T	1	3
C	A	T	4	T	G	3	C	G	8	G	0	1
T	C	C	4	C	C	3	C	G	8	G	0	0
T	C	C	4	C	C	3	C	G	8	G	0	0
T	C	C	4	C	C	3	C	G	8	G	0	0
T	C	C	4	C	C	3	C	G	8	G	0	0
T	C	C	4	C	C	3	C	G	8	G	0	0
T	C	C	4	C	C	3	C	G	8	G	0	0
T	C	C	4	C	C	3	C	G	8	G	0	0
T	C	C	4	C	C	N	N	N	N	N	N	N
T	C	C	4	C	C	3	C	G	8	G	0	0
N	N	N	N	N	N	0	T	G	8	G	0	1
N	N	N	N	N	N	3	C	G	8	G	0	1
C	A	T	0	T	G	0	T	G	8	G	0	1
N	N	N	N	N	N	N	N	N	N	N	N	N
T	C	C	4	C	C	3	C	G	8	G	N	N
N	N	N	N	N	N	3	C	G	8	G	0	0
C	A	T	0	T	G	0	T	G	8	G	0	1
T	C	C	4	C	C	3	C	G	8	G	0	0
N	N	N	N	N	N	3	C	G	8	G	0	1
N	N	N	N	N	N	3	C	G	8	G	0	1
T	C	C	4	C	C	3	C	G	8	G	0	0
T	C	C	4	C	C	N	N	N	N	N	N	N
C	A	T	0	T	G	0	T	G	8	G	0	1
T	C	C	4	C	C	3	C	G	8	G	0	0
C	A	T	0	N	N	3	C	G	8	T	1	3
N	N	N	N	N	N	3	C	G	8	T	1	3
T	C	C	4	C	C	3	C	G	8	G	0	0
C	A	T	0	T	G	0	T	A	11	-	N	0
T	C	C	4	C	C	3	C	G	8	G	0	0
T	C	C	4	C	C	3	C	G	8	G	0	N
T	C	C	4	C	C	N	N	N	N	N	N	N
T	C	C	4	C	C	N	N	N	N	N	N	N
T	C	C	4	C	C	3	C	G	8	G	0	1
T	C	C	4	C	C	N	N	N	N	N	N	N
T	C	C	4	C	C	N	N	N	N	N	N	N
C	A	T	N	N	N	N	N	N	N	N	N	N

C	A	T	0	T	G	3	T	G	8	G	0	1
C	A	T	0	T	G	0	T	A	11	-	N	0
T	C	C	4	C	C	3	C	A	11	-	N	0
C	A	T	0	T	G	3	C	G	8	G	0	1
C	A	T	4	T	G	3	C	G	8	G	0	1
T	C	C	4	C	C	3	C	A	11	-	N	0
T	C	C	4	C	C	3	C	G	8	G	0	0
C	A	N	N	N	N	3	C	G	8	G	0	1
C	A	T	0	N	N	3	C	G	8	G	0	1
T	C	C	4	C	C	3	C	G	8	G	0	0
C	A	T	4	T	C	N	N	A	8	A	0	1
C	A	T	0	T	G	0	T	A	11	-	N	0
C	A	T	0	T	G	3	C	G	8	G	0	1
T	C	C	4	C	C	3	C	G	8	T	10	0
T	C	C	4	C	C	3	T	G	8	T	1	3
C	A	T	0	T	G	3	T	G	8	T	1	3
T	C	C	4	C	C	3	C	G	8	G	0	1
C	A	T	N	N	N	0	T	A	8	A	0	1
C	A	T	4	N	N	N	N	N	N	N	N	N
C	A	T	4	T	C	3	T	G	8	T	1	3
T	C	T	4	C	C	3	C	G	8	G	0	1

-	-	T	3	C	C	3	C	G	8	G	0	1
T	C	C	4	C	C	3	C	G	8	G	0	1
T	C	C	4	C	C	3	T	G	8	T	1	3
C	A	T	0	T	G	N	N	N	N	N	N	N
T	C	C	4	C	C	3	T	G	8	T	1	3
C	A	T	0	T	G	3	C	G	8	G	0	1
T	C	T	4	C	C	3	T	G	8	T	1	3
T	C	T	4	C	G	0	T	G	8	G	0	1
N	N	N	N	N	N	N	T	A	8	A	0	1
T	C	C	4	C	C	0	T	G	8	G	0	0
T	C	C	4	C	C	3	C	G	8	G	0	1
N	N	N	N	N	N	N	N	N	N	N	N	N
N	N	N	N	N	N	0	C	G	0	G	0	3
T	C	C	4	C	C	0	T	G	8	G	0	1
T	C	C	4	C	C	3	C	G	8	G	0	1
T	C	T	4	C	C	3	C	G	8	G	0	1
T	C	T	4	C	C	3	C	G	8	G	0	1
C	A	T	4	N	N	0	T	G	11	-	N	0
T	C	T	4	C	C	3	C	G	8	G	0	1
T	C	C	4	C	C	N	N	G	8	G	0	1
C	A	T	4	T	C	N	N	N	N	N	N	N
C	A	T	0	T	G	3	T	G	8	T	1	3
N	N	N	N	N	N	0	T	G	8	G	0	1
T	C	C	4	C	C	3	C	G	8	G	0	1
T	C	C	4	C	C	3	T	G	8	T	1	3
C	A	T	0	T	G	3	C	G	8	G	0	1
C	A	T	0	T	G	0	T	A	8	A	0	1
N	N	N	N	N	N	0	T	G	N	N	N	N
C	A	T	0	T	G	0	T	G	8	G	0	1
N	N	N	N	N	N	N	N	N	N	N	N	N
T	C	C	4	C	C	0	T	G	8	G	0	1
T	C	C	4	C	C	0	T	G	8	G	0	1
T	C	C	4	C	C	3	C	A	11	-	N	0
T	C	C	4	C	C	3	C	G	8	G	0	0
N	N	N	N	N	N	3	C	G	N	N	N	N

N	N	N	N	N	N	N	N	N	N	N	N	N
N	N	N	N	N	N	N	N	N	N	N	N	N
N	N	N	N	N	N	0	T	A	11	-	N	0
C	A	T	4	T	G	3	C	G	8	G	0	1
T	C	C	4	C	C	3	C	A	11	-	N	0
C	A	T	4	C	C	0	T	G	8	G	0	1
C	A	T	4	T	G	3	T	G	8	G	0	1
C	A	T	4	T	G	3	C	G	8	G	0	1
C	A	T	0	T	G	0	T	G	8	G	0	1
T	C	C	4	C	C	3	C	G	8	G	0	0
N	N	N	N	N	N	3	C	G	8	G	0	1
N	N	N	N	N	N	3	C	G	8	G	0	1
T	C	C	4	C	C	3	C	G	8	G	0	1
T	C	C	4	C	C	3	C	G	8	G	0	0
N	N	N	N	N	N	3	C	G	8	G	0	1
T	C	C	4	C	C	3	C	A	11	-	N	0
T	C	C	4	C	C	3	C	A	11	-	N	0
T	C	C	4	C	G	3	C	G	8	G	0	1
T	C	C	4	C	C	3	C	G	8	G	0	0
N	N	N	N	N	N	0	T	G	8	G	0	1
C	A	T	4	T	C	0	T	A	11	-	N	0
N	N	N	N	N	N	3	C	G	8	G	0	0
T	C	C	4	C	C	3	C	G	8	G	0	0
C	A	T	4	T	G	N	N	N	N	N	N	N
T	C	C	4	C	C	3	C	G	8	G	0	0
T	C	C	4	C	C	3	C	G	8	G	0	0
T	C	C	4	C	C	3	C	G	8	G	0	0
T	C	C	4	C	C	3	C	G	8	G	0	0
C	A	T	4	N	N	3	C	G	8	G	0	1
T	C	C	4	C	C	N	N	N	N	N	N	N
T	C	C	4	C	C	3	C	G	8	G	0	0
C	A	T	4	T	C	N	N	A	8	A	0	1
T	C	C	4	C	C	3	C	G	8	G	0	1
C	A	T	4	T	G	3	C	G	8	G	0	1
C	A	T	4	T	C	0	T	A	8	A	0	1
T	C	C	4	C	C	3	C	G	8	G	0	1
C	A	T	4	T	G	0	T	G	8	G	0	1
T	C	C	4	C	C	3	C	G	8	G	0	1
N	N	T	4	C	C	N	N	N	N	N	N	N
T	C	C	4	C	C	3	C	G	8	G	0	1
-	-	T	3	C	C	3	C	G	8	G	0	1
T	C	C	4	C	C	0	C	G	8	G	0	1
C	A	T	0	T	G	0	T	A	11	-	N	0
N	N	N	N	N	N	0	T	G	11	-	N	0
C	A	T	4	T	G	3	C	G	8	G	0	1
T	C	T	4	C	C	3	C	G	8	G	0	1
N	N	N	N	N	N	N	N	N	N	N	N	N
T	C	C	4	C	C	3	C	A	11	-	N	0
C	A	T	0	T	G	3	C	G	8	G	0	1
T	C	C	4	C	C	3	C	G	8	G	0	1
T	C	C	4	C	C	N	N	N	N	N	N	N
T	C	C	4	C	C	3	C	G	8	G	0	0
C	A	T	0	T	G	3	C	G	8	G	0	1
C	A	T	0	T	G	3	C	G	8	G	0	1
T	C	C	4	C	C	0	T	A	11	-	N	0
T	C	T	4	C	C	3	C	G	8	G	0	1
T	C	C	4	C	C	3	C	G	8	G	0	0

T	C	C	4	C	C	3	C	G	8	G	0	0
N	N	N	N	N	N	3	C	G	8	T	1	3
C	A	T	4	T	G	3	C	N	N	N	N	N
T	C	C	4	C	C	3	C	G	8	G	0	0
T	C	C	4	C	C	3	C	G	8	G	0	0
T	C	C	4	C	C	N	N	N	N	N	N	N
C	A	T	0	T	G	0	T	G	8	G	0	1
T	C	C	4	C	C	0	T	G	8	G	0	1
T	C	C	4	C	C	3	C	G	8	G	0	0
N	N	N	N	N	N	3	C	G	8	G	0	1
T	C	C	4	C	C	0	T	G	8	G	0	1
C	A	T	4	T	G	N	N	N	N	N	N	N
C	A	T	0	T	G	N	N	N	N	N	N	N
N	N	N	N	N	N	3	C	G	8	G	0	1
T	C	C	4	C	C	3	C	G	8	G	0	0
C	A	T	0	T	G	0	T	A	11	-	N	0
N	N	N	N	N	N	3	C	G	8	G	0	1
N	N	N	N	N	N	3	C	G	8	G	0	1
N	N	N	N	N	N	3	C	G	8	G	0	1
T	C	T	4	C	C	3	C	G	8	G	0	1
C	A	T	0	T	G	0	T	A	11	-	N	0
T	C	T	4	C	C	N	N	N	N	N	N	N
C	A	T	0	T	G	0	T	A	11	-	N	0
T	C	C	4	C	C	3	C	G	8	G	0	0
T	C	C	4	C	C	N	N	N	N	N	N	N
T	C	C	4	C	C	3	C	G	8	G	0	1
T	C	C	4	C	C	0	T	G	8	T	1	3
-	-	T	3	C	C	3	C	G	8	G	0	1
N	N	N	N	N	N	N	N	N	N	N	N	N
T	C	T	4	C	C	3	C	G	8	G	0	1
T	C	C	4	C	C	3	C	G	8	G	0	0
T	C	C	4	C	C	3	C	G	8	G	0	0
T	C	C	4	C	C	N	N	N	N	N	N	N
C	A	T	4	T	G	N	T	G	8	G	0	1
C	A	T	0	T	G	3	T	G	8	T	1	3
T	C	C	4	C	C	3	C	G	8	G	0	0
T	C	C	4	C	C	3	C	G	8	G	0	0
T	C	C	4	C	C	3	C	G	8	G	0	0
C	A	T	0	T	G	3	C	G	8	G	0	N
T	C	T	4	C	C	3	C	G	8	G	0	1
T	C	T	4	C	C	3	C	G	8	G	0	1
-	-	T	3	C	C	3	C	G	8	G	0	1
T	C	C	4	C	C	N	N	N	N	N	N	N
N	N	N	N	N	N	3	C	G	8	G	0	1
T	C	C	4	C	C	N	N	N	N	N	N	N
T	C	C	4	C	C	0	T	G	8	T	1	3
T	C	T	4	C	C	3	C	G	8	G	0	1
T	C	T	4	C	C	3	C	G	8	G	0	1
N	N	N	N	N	N	3	C	G	8	G	0	1
C	A	T	0	T	G	0	T	A	11	-	N	0
C	A	T	0	T	G	N	N	N	N	N	N	N
T	C	C	4	C	C	0	T	A	8	G	0	1
C	A	T	0	T	G	0	T	A	11	-	N	0
C	A	T	0	T	G	0	T	A	11	-	N	0
C	A	T	4	T	C	N	N	N	N	N	N	N
T	C	C	4	C	C	3	C	G	8	G	0	0
T	C	C	4	C	C	3	C	G	8	G	0	0

T	C	C	4	C	C	3	C	A	11	-	N	0
T	C	C	4	C	C	3	C	A	11	-	N	0
T	C	C	4	C	C	3	C	A	11	-	N	0
T	C	C	4	C	C	3	C	G	8	G	0	1
T	C	C	4	C	C	0	T	G	8	G	0	1
C	A	T	0	T	G	0	T	G	8	G	0	1
T	C	C	4	C	C	3	C	G	8	G	0	0
T	C	C	4	C	C	3	C	A	11	-	N	0
C	A	T	0	T	G	N	N	N	N	N	N	N
C	A	T	0	T	G	3	C	G	8	G	0	1
C	A	T	0	N	N	3	C	G	8	T	1	3
C	A	T	0	T	G	0	T	A	11	-	N	0
T	C	T	4	C	C	3	C	G	8	G	0	1
N	N	N	N	N	N	3	C	G	8	G	0	0
C	A	T	4	N	N	0	T	G	8	G	0	1
T	C	C	4	C	C	3	C	G	N	N	N	N
T	C	C	4	C	C	3	C	G	18	-	N	0
C	A	T	4	T	G	3	C	G	8	G	0	1
C	A	T	0	T	G	0	T	A	11	-	N	0
T	C	T	4	C	C	3	C	G	8	G	0	1
T	C	T	4	C	C	N	N	N	N	N	N	N
T	C	C	4	C	C	3	C	A	11	-	N	0
T	C	C	4	C	C	3	C	G	8	G	0	0
T	C	T	4	C	C	3	C	G	8	G	0	1
T	C	C	4	C	C	0	T	G	8	G	0	1
N	N	N	N	N	N	3	C	G	8	G	0	1
T	C	C	4	C	C	N	N	N	N	N	N	N
C	A	T	0	T	G	0	T	A	11	-	N	0
T	C	C	4	C	C	N	N	N	N	N	N	N
T	C	C	4	C	C	3	T	G	8	T	1	3
C	A	T	4	T	C	3	T	G	8	T	1	3
T	C	C	4	C	C	0	T	G	8	G	0	0
T	C	C	4	C	C	N	N	N	N	N	N	N
T	C	T	4	C	C	3	C	G	8	G	0	1
T	C	C	4	C	C	3	C	G	8	G	0	1
T	C	C	4	C	C	3	C	G	8	G	0	1
C	A	T	4	T	C	0	T	G	8	G	0	1
T	C	C	4	C	C	3	C	G	8	G	0	0
T	C	C	4	C	C	3	C	G	8	G	0	0
T	C	C	4	C	C	3	C	G	8	G	0	0
C	A	T	0	T	G	0	T	G	8	G	0	1
N	N	C	4	C	C	N	N	N	N	N	N	N
T	C	C	4	C	C	3	C	G	8	G	0	0
T	C	C	4	C	C	3	C	G	8	G	0	0
T	C	C	4	C	C	0	T	G	8	T	11	0
C	A	T	0	N	N	3	C	G	8	T	1	3
C	A	T	4	T	C	3	T	G	11	-	N	0
C	A	T	4	T	G	3	T	G	8	G	0	1
N	N	N	N	N	N	3	C	G	8	G	0	0
C	A	T	4	N	N	3	C	G	8	G	0	0
T	C	C	4	C	C	3	C	G	8	G	0	0
N	N	N	N	N	N	N	N	N	N	N	N	N
T	C	C	4	C	C	3	C	G	8	G	0	0
T	C	C	4	C	C	0	T	G	8	G	0	0
T	C	C	4	C	C	3	C	G	8	G	0	0
N	N	N	N	N	N	N	N	N	N	N	N	N

ASL40	ASL41	ASL42	ASL43	ASL44	ASL45	ASL46	ASL47	ASL48	ASL49	ASL50	ASL51	ASL52
5	N	3	N	2	1	N	N	N	N	N	3	N
?	N	N	N	N	N	N	N	N	N	N	N	N
?	N	N	N	N	N	N	N	N	N	N	N	N
0	C	0	T	0	0	A	T	G	C	C	0	C
0	C	0	T	0	0	A	T	G	C	C	0	C
5	G	0	T	0	0	A	T	G	C	C	0	C
0	C	3	A	2	1	G	A	A	T	T	3	T
0	C	0	T	0	0	A	T	G	C	C	0	C
0	G	0	T	0	0	A	T	G	C	C	0	N
N	N	N	N	N	N	N	N	N	N	N	N	N
0	C	0	T	0	0	A	T	G	C	C	0	N
0	G	0	T	0	0	A	T	G	C	C	4	C
5	C	3	A	2	1	G	A	A	T	T	3	T
0	C	0	T	0	0	A	T	G	C	C	0	C
0	C	3	A	2	1	G	A	A	T	T	3	T
0	C	3	A	2	1	G	A	A	T	T	3	T
N	N	N	N	N	N	N	N	N	N	N	N	N
0	C	3	A	2	1	G	A	A	T	T	3	T
0	G	0	T	0	0	A	T	G	C	C	4	C
5	C	0	T	0	0	A	T	G	C	C	4	C
0	G	N	N	N	N	N	N	N	N	N	N	N
0	C	0	T	0	0	A	T	G	C	C	0	C
0	C	0	T	0	0	A	T	G	C	C	0	C
0	C	0	T	0	0	A	T	G	C	C	0	C
0	C	0	T	0	0	A	T	G	C	C	0	C
0	C	0	T	0	0	A	T	G	C	C	0	C
0	C	0	T	0	0	A	T	G	C	C	0	C
0	C	0	T	0	0	A	T	G	C	C	0	C
N	N	N	N	N	N	N	N	N	N	N	N	N
0	C	0	T	0	0	A	T	G	C	C	0	C
0	G	0	T	0	0	A	T	G	C	C	4	C
0	C	3	A	2	1	N	N	N	N	N	N	N
0	G	0	T	0	0	A	T	G	C	C	4	C
N	N	N	N	N	N	N	N	N	N	N	N	N
0	C	N	N	N	N	N	N	N	N	N	N	N
0	C	0	T	0	0	A	T	G	C	C	4	C
0	G	0	T	0	0	A	T	G	C	C	4	C
0	C	0	T	0	0	A	T	G	C	C	0	C
0	C	3	A	2	1	G	A	A	T	T	3	T
0	C	3	A	2	1	G	A	A	T	T	3	T
0	C	0	T	0	0	A	T	G	C	C	0	C
N	N	N	N	N	N	N	N	N	N	N	N	N
0	G	0	T	0	0	A	T	G	C	C	4	C
0	C	0	T	0	0	A	T	G	C	C	0	C
5	C	0	T	0	0	A	T	G	C	C	4	C
5	C	0	T	0	0	A	T	G	C	C	4	C
0	C	0	T	0	0	A	T	G	C	C	0	C
0	G	0	T	0	0	A	T	G	C	C	4	C
0	C	0	T	0	0	A	T	G	C	C	0	C
N	N	N	N	N	N	N	N	N	N	N	N	N
N	N	N	N	N	N	N	N	N	N	N	N	N
N	N	N	N	N	N	N	N	N	N	N	N	N
5	C	3	A	2	1	G	A	A	T	T	3	T
N	N	N	N	N	N	N	N	N	N	N	N	N
N	N	N	N	N	N	N	N	N	N	N	N	N
N	N	N	N	N	N	N	N	N	N	N	N	N

0	G	0	T	0	0	A	T	G	C	C	4	N
0	G	0	T	0	0	A	T	G	C	C	4	C
0	C	0	T	0	0	A	T	G	C	C	0	C
0	G	0	T	0	0	A	T	G	C	C	4	C
0	G	0	T	0	0	A	T	G	C	C	4	C
0	C	0	T	0	0	A	T	G	C	C	0	C
0	C	0	T	0	0	A	T	G	C	C	0	C
0	G	0	T	0	0	A	T	G	C	C	4	C
0	G	0	T	0	0	A	T	G	C	C	4	C
0	C	0	T	0	0	A	T	G	C	C	0	C
5	C	0	T	0	0	A	T	G	C	C	0	C
0	G	0	T	0	0	A	T	G	C	C	4	C
0	C	0	T	0	0	A	T	G	C	C	4	C
0	G	0	T	0	0	N	N	N	N	N	N	N
5	C	0	T	0	0	A	T	G	C	C	0	C
5	C	3	A	2	1	G	A	A	T	T	3	T
5	C	0	T	0	0	A	T	G	C	C	4	C
0	G	0	T	0	0	A	T	G	C	C	0	C
N	N	N	N	N	N	N	N	N	N	N	N	N
5	C	N	N	N	N	N	N	N	N	N	N	N
5	C	3	A	2	1	N	N	N	N	N	N	N

5	C	0	T	0	0	A	T	G	C	C	0	N
5	C	0	T	0	0	A	T	G	C	C	0	C
5	C	3	A	2	1	G	A	A	T	T	3	T
N	N	N	N	N	N	N	N	N	N	N	N	N
5	C	3	A	2	1	G	A	N	N	N	N	N
0	C	0	T	0	0	A	T	G	C	C	4	C
5	C	3	A	2	1	G	A	A	T	T	3	T
5	C	0	T	0	0	A	T	G	C	C	4	C
0	G	0	T	0	0	A	T	G	C	C	4	C
0	C	0	T	0	0	A	T	G	C	C	0	C
0	C	0	T	0	0	A	T	G	C	C	0	C
N	N	N	N	N	N	N	N	N	N	N	N	N
0	C	3	T	0	0	A	T	G	C	C	0	C
0	C	3	A	2	1	G	A	A	T	T	3	T
0	G	0	T	0	0	A	T	G	C	C	0	C
0	G	0	T	0	0	A	T	G	C	C	0	C
0	C	N	N	N	N	N	N	N	N	N	N	N
0	G	0	T	0	0	A	T	G	C	C	4	C
0	G	0	T	0	0	A	T	G	C	C	0	-
0	C	0	T	0	0	A	T	G	C	C	0	C
N	N	N	N	N	N	N	N	N	N	N	N	N
5	C	3	T	0	0	A	T	G	C	C	N	C
0	C	0	T	0	0	A	T	G	C	C	0	C
0	C	0	T	0	0	A	T	G	C	C	0	C
5	C	0	T	0	0	A	T	G	C	C	0	C
0	G	0	T	0	0	A	T	G	C	C	4	C
5	C	0	T	0	0	A	T	G	C	C	0	C
N	N	N	N	N	N	N	N	N	N	N	N	N
0	C	N	N	N	N	N	N	N	N	N	N	N
N	N	N	N	N	N	N	N	N	N	N	N	N
0	G	0	T	0	0	A	T	G	C	C	4	C
0	G	0	T	0	0	A	T	G	C	C	4	C
0	C	0	T	0	0	A	T	G	C	C	0	C
0	C	N	N	N	N	N	N	N	N	N	N	N
N	N	N	N	N	N	N	N	N	N	N	N	N

N	N	N	N	N	N	N	N	N	N	N	N	N
N	N	N	N	N	N	N	N	N	N	N	N	N
0	G	0	T	0	0	A	T	G	C	C	4	C
0	G	0	T	0	0	A	T	G	C	C	4	C
0	C	0	T	0	0	A	T	G	C	C	0	N
5	C	0	T	0	N	N	N	N	N	N	N	N
5	C	0	T	0	0	A	T	G	C	C	0	C
0	G	0	T	0	0	A	T	G	C	C	4	C
5	C	0	T	0	0	A	T	G	C	C	4	C
0	C	0	T	0	0	A	T	G	C	C	0	C
0	C	3	A	2	1	G	A	A	T	T	3	T
0	C	3	A	2	1	G	A	A	T	T	3	T
5	C	3	A	2	1	G	A	A	T	T	3	T
0	C	0	T	0	0	A	T	G	C	C	0	C
0	C	3	A	2	1	G	A	A	T	T	3	T
0	C	0	T	0	0	A	T	G	C	C	0	C
0	C	0	T	0	N	N	N	N	N	N	N	N
5	G	3	A	2	1	G	A	A	T	T	3	T
0	C	0	T	0	0	A	T	G	C	C	0	C
0	G	0	T	0	0	A	T	G	C	C	4	C
0	G	0	T	0	0	A	T	G	C	C	4	C
0	C	3	A	2	1	G	A	A	T	T	3	T
0	C	0	T	0	0	A	T	G	C	C	0	C
N	N	N	N	N	N	N	N	N	N	N	N	N
0	C	0	T	0	0	A	T	G	C	C	0	C
0	C	0	T	0	0	A	T	G	C	C	0	C
0	C	N	N	N	N	N	N	N	N	N	N	N
0	C	0	T	0	0	A	T	G	C	C	0	C
5	C	0	T	0	0	A	T	G	C	C	4	C
N	N	N	N	N	N	N	N	N	N	N	N	N
0	C	0	T	0	0	A	T	G	C	C	0	N
5	C	0	T	0	0	A	T	G	C	C	0	C
0	G	0	T	0	0	A	T	G	C	C	0	C
0	G	0	T	0	0	A	T	G	C	C	4	N
5	C	0	T	0	0	A	T	G	C	C	0	C
0	C	0	T	0	0	A	T	G	C	C	0	C
0	C	0	T	0	0	A	T	G	C	C	0	C
5	C	0	T	0	0	A	T	G	C	C	4	C
N	N	N	N	N	N	N	N	N	N	N	N	N
0	C	0	T	0	0	A	T	G	C	C	0	C
5	C	0	T	0	0	A	T	G	C	C	0	C
0	G	0	T	0	0	A	T	G	C	C	0	C
0	G	0	T	0	0	A	T	G	C	C	4	C
0	G	0	T	0	0	A	T	G	C	C	4	C
0	G	0	T	0	0	A	T	G	C	C	4	C
0	C	0	T	0	0	A	T	G	C	C	4	C
N	N	N	N	N	N	N	N	N	N	N	N	N
0	C	0	T	0	0	A	T	G	C	C	0	C
0	G	0	T	0	0	A	T	G	C	C	4	C
0	G	0	T	0	0	A	T	G	C	C	0	C
N	N	N	N	N	N	N	N	N	N	N	N	N
0	C	0	T	0	0	A	T	G	C	C	0	C
0	N	N	N	N	N	N	N	N	N	N	N	N
0	G	0	T	0	0	A	T	G	C	C	4	C
0	G	0	T	0	0	A	T	G	C	C	4	N
0	C	0	T	0	0	A	T	G	C	C	4	C
0	C	0	T	0	0	A	T	G	C	C	0	C

0	C	0	T	0	0	A	T	G	C	C	0	C
5	C	0	T	0	0	A	T	G	C	C	4	C
N	N	N	N	N	N	N	N	N	N	N	N	N
0	C	0	T	0	0	A	T	G	C	C	0	C
0	C	0	T	0	0	A	T	G	C	C	0	C
N	N	0	T	0	0	A	T	G	C	C	0	N
0	G	0	T	0	0	A	T	G	C	C	4	C
0	G	0	T	0	0	A	T	G	C	C	4	N
0	C	0	T	0	0	A	T	G	C	C	0	N
0	C	3	A	2	1	G	A	A	T	T	3	T
0	G	0	T	0	0	A	T	G	C	C	4	C
N	N	N	N	N	N	N	N	N	N	N	N	N
N	N	N	N	N	N	N	N	N	N	N	N	N
0	G	0	T	0	0	A	T	G	C	C	4	C
0	C	0	T	0	0	N	N	N	N	N	N	N
0	G	0	T	0	0	A	T	G	C	C	4	C
0	C	3	A	2	1	G	A	A	T	T	3	T
0	C	3	A	2	1	G	A	A	T	T	3	T
0	C	3	A	2	1	G	A	A	T	T	3	T
0	C	0	T	0	0	A	T	G	C	C	4	C
0	G	0	T	0	0	A	T	G	C	C	4	C
N	N	N	N	N	N	N	N	N	N	N	N	N
0	G	0	T	0	0	A	T	G	C	C	4	C
0	C	0	T	0	0	A	T	G	C	C	0	C
N	N	N	N	N	N	N	N	N	N	N	N	N
5	C	3	A	2	N	N	N	N	N	N	N	N
5	C	3	A	2	1	G	A	A	T	T	3	T
0	C	0	T	0	0	A	T	G	C	C	0	-
N	N	N	N	N	N	N	N	N	N	N	N	N
0	C	0	T	0	0	A	T	G	C	C	0	C
0	C	0	T	0	0	A	T	G	C	C	0	C
0	C	0	T	0	0	A	T	G	C	C	0	C
N	N	N	N	N	N	N	N	N	N	N	N	N
5	C	0	T	0	0	A	T	G	C	C	0	C
5	C	0	T	0	0	A	T	G	C	C	4	C
0	C	0	T	0	0	A	T	G	C	C	0	C
0	C	0	T	0	0	A	T	G	C	C	0	C
0	C	0	T	0	0	A	T	G	C	C	0	C
N	N	N	N	N	N	N	N	N	N	N	N	N
0	C	0	T	0	0	A	T	G	C	C	0	C
0	C	0	T	0	0	A	T	G	C	C	0	C
0	C	0	T	0	0	A	T	G	C	C	0	C
N	N	N	N	N	N	N	N	N	N	N	N	N
0	C	N	N	N	N	N	N	N	N	N	N	N
N	N	N	N	N	N	N	N	N	N	N	N	N
N	N	N	N	N	N	N	N	N	N	N	N	N
0	C	0	T	0	0	A	T	G	C	C	0	C
0	C	0	T	0	0	A	T	G	C	C	0	N
0	C	0	T	0	0	A	T	G	C	C	0	C
0	G	0	T	0	0	A	T	G	C	C	4	C
N	N	N	N	N	N	N	N	N	N	N	N	N
0	C	3	A	2	1	N	N	N	N	N	N	N
0	G	0	T	0	0	A	T	G	C	C	4	C
0	G	0	T	0	0	A	T	G	C	C	4	C
N	N	N	N	N	N	N	N	N	N	N	N	N
0	C	0	T	0	0	A	T	G	C	C	0	C
0	C	0	T	0	0	A	T	G	C	C	0	N

0	C	0	T	0	0	A	T	G	C	C	0	C
0	C	0	T	0	0	A	T	G	C	C	0	C
0	C	0	T	0	0	A	T	G	C	C	0	C
0	C	0	T	0	0	A	T	G	C	C	0	C
0	G	0	T	0	0	A	T	G	C	C	4	C
0	G	0	T	0	0	A	T	G	C	C	4	C
0	C	0	T	0	0	A	T	G	C	C	0	C
0	C	0	T	0	0	A	T	G	C	C	0	C
N	N	N	N	N	N	N	N	N	N	N	N	N
0	G	0	T	0	0	A	T	G	C	C	4	C
5	C	0	T	0	0	A	T	G	C	C	4	C
0	G	0	T	0	0	A	T	G	C	C	4	C
0	C	0	T	0	0	A	T	G	C	C	4	C
0	C	3	N	N	1	N	N	N	N	N	N	N
5	C	N	N	N	N	N	N	N	N	N	N	N
N	N	N	N	N	N	N	N	N	N	N	N	N
N	N	N	N	N	N	N	N	N	N	N	N	N
0	G	0	T	0	0	A	T	G	C	C	4	N
0	G	0	T	0	0	N	N	N	N	N	N	N
0	C	0	T	0	0	A	T	G	C	C	4	C
N	N	N	N	N	N	N	N	N	N	N	N	N
0	C	0	T	0	0	A	T	G	C	C	0	C
0	G	0	T	0	0	A	T	G	C	C	0	C
0	C	0	T	0	0	A	T	G	C	C	4	N
0	G	0	T	0	0	A	T	G	C	C	4	N
0	C	3	A	2	1	G	A	A	T	T	3	T
N	N	N	N	N	N	N	N	N	N	N	N	N
0	G	0	T	0	0	A	T	G	C	C	4	C
N	N	N	N	N	N	N	N	N	N	N	N	N
5	C	0	T	0	0	A	T	G	C	C	0	C
5	C	0	T	0	0	A	T	G	C	C	0	C
0	C	0	T	0	0	N	N	N	N	N	N	N
N	N	N	N	N	N	N	N	N	N	N	N	N
5	C	3	A	2	1	G	A	A	T	T	3	T
5	C	0	T	0	0	A	T	G	C	C	0	C
5	C	0	T	0	0	A	T	G	C	C	0	C
5	C	0	T	0	0	A	T	G	C	C	4	C
0	C	0	T	0	0	A	T	G	C	C	0	C
0	C	0	T	0	0	A	T	G	C	C	0	N
0	C	0	T	0	0	A	T	G	C	C	0	C
0	G	0	T	0	0	A	T	G	C	C	4	C
N	N	N	N	N	N	N	N	N	N	N	N	N
0	C	0	T	0	0	A	T	G	C	C	0	N
0	C	0	T	0	0	A	T	G	C	C	0	N
0	C	0	T	0	0	N	N	N	N	N	N	N
5	C	0	T	0	0	A	T	G	C	C	4	C
0	G	N	N	N	N	N	N	N	N	N	N	N
0	G	0	T	0	0	A	T	G	C	C	4	C
0	C	0	T	0	0	A	T	G	C	C	0	C
0	C	0	T	0	0	A	T	G	C	C	4	C
0	C	0	T	0	0	A	T	G	C	C	4	C
N	N	N	N	N	N	N	N	N	N	N	N	N
0	C	0	T	0	0	A	T	G	C	C	0	C
0	C	0	T	0	0	A	T	G	C	C	0	C
0	C	0	T	0	0	A	T	G	C	C	0	C
N	N	N	N	N	N	N	N	N	N	N	N	N

ASL53	ABC1	ABC2	ABC3	ABC4	ABC5	ABC6	ABC7	ABC8	ABC9	ABC10	ABC11	ABC12	ABC13
N	2	G	27	11	5	T	C	G	T	A	1	3	G
N	2	G	27	11	0	T	C	G	T	A	0	3	G
N	N	N	N	N	N	N	N	N	N	N	N	N	N
G	2	G	27	11	0	T	C	G	T	A	0	3	G
G	0	C	0	1	5	C	T	A	C	T	1	0	C
T	2	G	27	11	0	C	C	G	T	A	0	3	G
T	2	G	0	1	5	C	T	A	C	T	1	3	C
G	2	G	0	1	5	C	T	A	C	T	1	3	C
N	2	G	0	1	5	C	T	A	C	T	1	3	C
N	N	N	N	N	N	N	N	N	N	N	N	N	N
N	2	G	27	11	0	T	C	G	T	A	0	3	G
T	2	G	0	1	5	C	T	A	C	T	1	3	C
T	2	G	27	11	0	T	C	G	T	A	0	3	G
G	N	N	N	1	5	C	T	A	C	T	1	3	C
T	2	G	0	1	5	C	T	A	C	T	1	3	C
T	2	G	0	1	5	C	T	A	C	T	1	3	C
N	2	G	0	1	5	C	T	A	C	T	1	3	C
T	0	C	0	1	5	C	T	A	C	T	1	0	C
T	2	G	27	11	0	T	C	G	T	A	0	3	G
T	2	G	0	1	5	C	T	A	C	T	1	3	C
N	2	G	0	1	5	C	T	A	C	T	1	3	C
G	2	G	0	1	5	C	T	A	C	T	1	3	C
G	2	G	0	1	5	C	T	A	C	T	1	3	C
G	0	C	0	1	5	C	T	A	C	T	1	0	C
G	0	C	0	1	5	C	T	A	C	T	1	0	C
G	2	G	0	1	5	C	T	A	C	T	1	3	C
G	2	G	27	11	0	T	C	G	T	A	0	3	G
G	2	G	0	1	5	C	T	A	C	T	1	3	C
N	2	G	27	11	0	C	C	G	T	A	0	3	G
G	2	G	0	1	5	C	T	A	C	T	1	3	C
T	0	C	0	1	5	C	T	A	C	T	1	0	C
N	2	G	0	1	5	C	T	A	C	T	1	3	C
T	N	N	N	1	5	C	T	A	C	T	1	3	C
N	N	N	0	0	5	C	T	A	C	T	1	0	C
N	N	N	0	1	5	C	T	A	C	T	1	3	C
T	2	G	0	1	5	C	T	A	C	T	1	3	C
N	N	N	N	N	N	N	N	N	N	N	N	N	N
G	2	G	0	1	5	C	T	A	C	T	1	3	C
T	2	G	0	1	5	C	T	A	C	T	1	3	C
T	2	G	0	1	5	C	T	A	C	T	1	3	C
G	2	G	0	1	5	C	T	A	C	T	1	3	C
N	2	G	0	1	5	C	T	A	C	T	1	3	C
T	0	C	0	1	5	C	T	A	C	T	1	0	C
N	2	G	0	1	5	C	T	A	C	T	1	3	C
T	2	G	0	1	5	C	T	A	C	T	1	3	C
T	0	C	0	1	5	C	T	A	C	T	1	0	C
G	0	C	0	1	5	C	T	A	C	T	1	0	C
T	2	G	27	11	0	C	C	G	T	A	0	3	G
G	2	G	27	11	0	T	C	G	T	A	0	3	G
N	2	G	27	11	0	C	C	G	T	A	0	3	G
N	N	N	N	N	N	N	N	N	N	N	N	N	N
N	2	G	27	11	0	T	C	G	T	A	0	3	G
T	2	G	27	11	0	T	C	G	T	A	0	3	G
N	2	G	27	11	0	C	C	G	T	A	0	3	G
N	2	G	27	11	0	T	C	G	T	A	0	3	G
N	0	C	0	1	5	C	T	A	C	T	1	0	C

N	N	N	N	1	5	C	T	A	C	T	1	0	C
T	2	G	0	1	5	C	T	A	C	T	1	3	C
G	2	G	27	11	0	T	C	G	T	A	0	3	G
T	N	N	N	N	N	N	N	N	N	N	N	N	N
T	2	G	27	11	0	T	C	G	T	A	0	3	G
G	N	N	N	N	N	N	N	N	N	N	N	N	N
G	0	C	0	1	5	C	T	A	C	T	1	0	C
T	2	G	0	1	5	C	T	A	C	T	1	3	C
T	2	G	0	1	5	C	T	A	C	T	1	3	C
G	2	G	0	1	5	C	T	A	C	T	1	3	C
T	0	C	0	0	5	C	T	A	C	T	1	0	C
T	0	C	0	1	5	C	T	A	C	T	1	0	C
T	0	C	0	1	5	C	T	A	C	T	1	0	C
N	0	C	0	1	5	C	T	A	C	T	1	0	C
G	0	C	0	1	5	C	T	A	C	T	1	0	C
T	2	G	27	11	0	T	C	G	T	A	0	3	G
T	2	G	27	11	0	T	C	G	T	A	0	3	G
T	2	G	27	11	0	T	C	G	T	A	0	3	G
N	2	G	27	11	0	T	C	G	T	A	0	3	G
N	N	N	N	N	N	N	N	N	N	N	N	N	N
N	2	G	27	11	0	T	C	G	T	A	0	3	G

N	2	G	27	11	0	T	C	G	T	A	0	3	G
G	2	G	27	11	0	T	C	G	T	A	0	3	G
T	2	G	0	1	5	C	T	A	C	T	1	3	C
N	0	C	0	1	5	C	T	A	C	T	1	0	C
N	2	G	27	11	0	T	C	G	T	A	0	3	G
T	N	N	N	N	N	N	N	N	N	N	N	N	N
T	N	N	N	N	5	C	C	G	T	A	0	3	G
G	2	G	0	1	5	C	T	A	C	T	1	3	C
T	0	C	0	1	5	C	T	A	C	T	1	0	C
G	N	N	N	2	0	C	C	G	T	A	0	3	G
T	2	G	0	2	0	C	C	G	T	A	0	3	G
N	N	N	N	N	N	N	N	N	N	N	N	N	N
T	N	N	N	N	0	C	C	G	T	A	0	3	G
T	2	G	0	1	5	C	T	A	C	T	1	3	C
G	2	G	27	11	0	T	C	G	T	A	0	3	G
G	2	G	0	1	5	C	T	A	C	T	1	3	C
N	2	G	0	1	5	C	T	A	C	T	1	3	C
T	0	C	0	1	5	C	T	A	C	T	1	0	C
-	2	G	27	11	0	C	C	G	T	A	0	3	G
G	N	N	N	N	N	N	N	N	N	N	N	N	N
N	2	G	0	1	5	C	T	A	C	T	1	3	C
-	0	C	0	1	5	C	T	A	C	T	1	0	C
G	N	-	N	N	N	-	-	-	-	-	N	3	G
G	2	G	0	1	5	C	T	A	C	T	1	3	C
G	N	N	N	1	5	C	T	A	C	T	1	3	C
T	2	G	27	11	0	T	C	G	T	A	0	3	G
-	2	G	0	1	5	C	T	A	C	T	1	3	C
N	2	G	27	11	0	T	C	G	T	A	0	3	G
N	N	N	N	N	5	T	C	G	T	A	0	3	G
N	0	C	0	1	5	C	T	A	C	T	1	0	C
T	0	C	0	1	5	C	T	A	C	T	1	0	C
T	2	G	27	11	0	T	C	G	T	A	0	3	G
G	N	N	N	N	N	N	N	N	N	N	N	N	N
N	2	G	27	11	0	T	C	G	T	A	0	3	G
N	0	C	0	1	5	C	T	A	C	T	1	0	C

N	N	N	0	1	5	C	T	A	C	T	1	0	C
N	0	C	0	1	5	C	T	A	C	T	1	0	C
T	0	C	0	1	5	C	T	A	C	T	1	0	C
T	2	G	27	11	0	C	C	G	T	A	0	3	G
N	2	G	0	1	5	C	T	A	C	T	1	3	C
N	2	G	27	11	0	T	C	G	T	A	0	3	G
T	N	N	0	1	5	C	T	A	C	T	1	3	C
T	2	G	27	11	0	C	C	G	T	A	0	3	G
T	2	G	27	11	0	T	C	G	T	A	0	3	G
G	2	G	27	11	0	T	C	G	T	A	0	3	G
T	2	G	0	1	5	C	T	A	C	T	1	3	C
T	2	G	0	1	5	C	T	A	C	T	1	3	C
T	2	G	27	11	0	T	C	G	T	A	0	3	G
G	N	N	N	1	5	C	T	A	C	T	1	3	C
T	2	G	0	1	5	C	T	A	C	T	1	3	C
G	2	G	27	11	0	T	C	G	T	A	0	3	G
N	2	G	27	11	0	T	C	G	T	A	0	3	G
T	2	G	0	2	0	C	C	G	T	A	0	3	G
G	2	G	27	11	0	T	C	G	T	A	0	3	G
T	0	C	0	1	5	C	T	A	C	T	1	0	C
T	2	G	27	11	0	T	C	G	T	A	0	3	G
T	N	N	N	N	N	N	N	N	N	N	N	N	N
G	2	G	0	1	5	C	T	A	C	T	1	3	C
N	2	G	27	11	0	C	C	G	T	A	0	3	G
G	N	N	0	1	5	C	T	A	C	T	1	3	C
G	2	G	27	11	0	T	C	G	T	A	0	3	G
N	0	C	0	0	5	C	T	A	C	T	1	0	C
G	0	C	0	0	5	C	T	A	C	T	1	0	C
T	2	G	27	11	0	C	C	G	T	A	0	3	G
N	N	N	N	N	N	N	N	N	N	N	N	N	N
N	2	G	27	11	0	C	C	G	T	A	0	3	G
T	0	C	0	1	5	C	T	A	C	T	1	0	C
G	N	N	N	N	N	N	N	N	N	N	N	N	N
N	0	C	0	1	5	C	T	A	C	T	1	0	C
T	2	G	0	1	5	C	T	A	C	T	1	3	C
G	N	N	N	N	N	N	N	N	N	N	N	N	N
T	0	C	0	1	5	C	T	A	C	T	1	0	C
T	N	N	N	N	N	N	N	N	N	N	N	N	N
N	0	C	0	1	5	C	T	A	C	T	1	0	C
G	2	G	27	11	0	T	C	G	T	A	0	3	G
G	2	G	27	11	0	T	C	G	T	A	0	3	G
G	2	G	27	11	0	T	C	G	T	A	0	3	G
T	2	G	0	1	5	C	T	A	C	T	1	3	C
T	N	N	0	1	5	C	T	A	C	T	1	3	C
T	0	C	0	0	5	C	T	A	C	T	1	0	C
T	2	G	27	11	0	C	C	G	T	A	0	3	G
N	0	C	0	0	5	C	T	A	C	T	1	0	C
G	0	C	0	1	5	C	T	A	C	T	1	0	C
T	2	G	27	11	0	C	C	G	T	A	0	3	G
G	2	G	27	11	0	T	C	G	T	A	0	3	G
N	N	N	N	1	5	C	T	A	C	T	1	3	C
G	0	C	0	1	5	C	T	A	C	T	1	0	C
N	N	N	N	N	N	N	N	N	N	N	N	N	N
T	2	G	0	1	5	C	T	A	C	T	1	3	C
N	0	C	0	0	5	C	T	A	C	T	1	0	C
T	2	G	27	11	0	C	C	G	T	A	0	3	G
G	0	C	0	1	5	C	T	A	C	T	1	0	C

N	0	C	0	1	5	C	T	A	C	T	1	0	C
T	2	G	0	1	5	C	T	A	C	T	1	3	C
N	2	G	27	11	0	C	C	G	T	A	0	3	G
G	N	N	N	N	N	N	N	N	N	N	N	N	N
G	0	C	0	1	5	C	T	A	C	T	1	0	C
N	2	G	27	11	0	C	C	G	T	A	0	3	G
T	2	G	27	11	0	C	C	G	T	A	0	3	G
N	N	N	0	1	5	C	T	A	C	T	1	3	C
N	2	G	0	1	5	C	T	A	C	T	1	3	C
T	2	G	0	1	5	C	T	A	C	T	1	3	C
T	N	N	0	1	5	C	T	A	C	T	1	3	C
N	N	N	N	N	N	N	N	N	N	N	N	N	N
N	2	G	0	1	5	C	T	A	C	T	1	3	C
T	0	C	0	1	5	C	T	A	C	T	1	0	C
N	0	C	0	1	5	C	T	A	C	T	1	0	C
T	0	C	0	1	5	C	T	A	C	T	1	0	C
T	N	N	N	N	N	N	N	N	N	N	N	N	N
T	N	N	0	1	5	C	T	A	C	T	1	3	C
T	0	C	0	1	5	C	T	A	C	T	1	0	C
T	2	G	N	11	0	C	C	G	T	A	0	3	G
T	0	C	0	1	5	C	T	A	C	T	1	0	C
N	2	G	27	11	0	T	C	G	T	A	0	3	G
T	0	C	0	1	5	C	T	A	C	T	1	0	C
G	2	G	0	1	5	C	T	A	C	T	1	3	C
N	N	N	N	N	N	N	N	N	N	N	N	N	N
N	N	N	N	N	N	N	N	N	N	N	N	N	N
T	0	C	0	1	5	C	T	A	C	T	1	0	C
N	0	C	0	1	5	C	T	A	C	T	1	0	C
N	2	G	27	11	0	T	C	G	T	A	0	3	G
G	2	G	27	11	0	T	C	G	T	A	0	3	G
G	2	G	0	1	5	C	T	A	C	T	1	3	C
G	2	G	0	1	5	C	T	A	C	T	1	3	C
N	N	N	N	2	5	C	T	A	C	T	1	3	C
T	2	G	27	11	0	T	C	G	T	A	0	3	G
T	2	G	27	11	0	T	C	G	T	A	0	3	G
G	2	G	0	1	5	C	T	A	C	T	1	3	C
G	2	G	0	1	5	C	T	A	C	T	1	3	C
G	2	G	0	1	5	C	T	A	C	T	1	3	C
N	0	C	0	1	5	C	T	A	C	T	1	0	C
N	2	G	27	11	0	T	C	G	T	A	0	3	G
G	2	G	0	1	5	C	T	A	C	T	1	3	C
G	0	C	0	1	5	C	T	A	C	T	1	0	C
N	0	C	0	1	5	C	T	A	C	T	1	0	C
N	0	C	0	1	5	C	T	A	C	T	1	0	C
N	N	N	N	N	N	N	N	N	N	N	N	N	N
N	0	C	0	1	5	C	T	A	C	T	1	0	C
G	N	N	N	N	N	N	N	N	N	N	N	N	N
N	2	G	27	11	0	T	C	G	T	A	0	3	G
G	N	N	N	N	5	C	T	A	C	T	1	3	C
T	N	N	N	N	5	C	T	A	C	T	1	3	C
N	2	G	0	2	5	C	T	A	C	T	1	3	C
N	0	C	0	1	5	C	T	A	C	T	1	0	C
T	2	G	0	1	5	C	T	A	C	T	1	3	C
T	2	G	0	1	5	C	T	A	C	T	1	3	C
N	2	G	27	11	0	T	C	G	T	A	0	3	G
G	2	G	0	1	5	C	T	A	C	T	1	3	C
N	2	G	0	1	5	C	T	A	C	T	1	3	C

G	2	G	27	11	0	C	C	G	T	A	0	3	G
G	N	N	N	N	N	N	N	N	N	N	N	N	N
G	2	G	0	1	5	C	T	A	C	T	1	3	C
G	0	C	0	1	5	C	T	A	C	T	1	0	C
T	2	G	0	1	5	C	T	A	C	T	1	3	C
T	0	C	0	1	5	C	T	A	C	T	1	0	C
G	2	G	0	1	5	C	T	A	C	T	1	3	C
G	0	C	0	1	5	C	T	A	C	T	1	0	C
N	0	C	0	1	5	C	T	A	C	T	1	0	C
T	N	N	N	N	N	N	N	N	N	N	N	N	N
T	N	N	N	N	N	N	N	N	N	N	N	N	N
T	2	G	27	11	0	C	C	G	T	A	0	3	G
T	2	G	27	11	0	C	C	G	T	A	0	3	G
N	2	G	27	11	0	C	C	G	T	A	0	3	G
N	N	N	N	N	5	C	T	A	C	T	1	3	N
N	0	C	0	1	5	C	T	A	C	T	1	0	C
N	0	C	0	0	5	C	T	A	C	T	1	0	C
N	2	G	0	1	5	C	T	A	C	T	1	3	C
N	2	G	27	11	0	T	C	G	T	A	0	3	G
T	2	G	27	11	0	C	C	G	T	A	0	3	G
N	2	G	0	1	5	C	T	A	C	T	1	3	C
G	0	C	0	1	5	C	T	A	C	T	1	0	C
G	2	G	27	11	0	T	C	G	T	A	0	3	G
N	2	G	27	11	0	T	C	G	T	A	0	3	G
N	2	G	27	11	0	T	C	G	T	A	0	3	G
T	N	N	N	N	N	N	N	N	N	N	N	N	N
N	2	G	0	1	5	C	T	A	C	T	1	3	C
N	0	C	0	1	5	C	T	A	C	T	1	0	C
N	0	C	0	1	5	C	T	A	C	T	1	0	C
G	2	G	27	11	0	T	C	G	T	A	0	3	G
G	2	G	0	1	5	C	T	A	C	T	1	3	C
N	2	G	27	11	0	T	C	G	T	A	0	3	G
N	2	G	0	1	5	C	T	A	C	T	1	3	C
T	2	G	0	1	5	C	T	A	C	T	1	3	C
G	N	N	N	N	N	N	N	N	N	N	N	N	N
G	2	G	27	11	0	T	C	G	T	A	0	3	G
T	2	G	27	11	0	T	C	G	T	A	0	3	G
G	2	G	27	11	0	T	C	G	T	A	0	3	G
N	2	G	0	1	5	C	T	A	C	T	1	3	C
G	2	G	0	1	5	C	T	A	C	T	1	3	C
T	2	G	0	1	5	C	T	A	C	T	1	3	C
N	N	N	N	N	N	N	N	N	N	N	N	N	N
N	2	G	0	1	5	C	T	A	C	T	1	3	C
N	2	G	0	1	5	C	T	A	C	T	1	3	C
N	2	G	0	1	5	C	T	A	C	T	1	3	C
T	2	G	0	1	5	C	T	A	C	T	1	3	C
N	2	G	N	1	5	C	T	A	C	T	1	3	N
T	0	C	0	1	5	C	T	A	C	T	1	0	C
G	0	C	0	1	5	C	T	A	C	T	1	0	C
T	2	G	0	1	5	C	T	A	C	T	1	3	C
T	0	C	0	1	5	C	T	A	C	T	1	0	C
N	0	C	0	1	5	C	T	A	C	T	1	0	C
G	2	G	27	11	0	T	C	G	T	A	0	3	G
G	2	G	N	2	5	C	T	A	C	T	1	3	C
G	2	G	27	11	0	T	C	G	T	A	0	3	G
N	N	N	N	N	N	N	N	N	N	N	N	N	N

ABC14	ABC15	ABC16	ABC17	ABC18	ABC19	ABC20	ABC21	AL16_1	AL1_1	AL1_2	AL1_3	AL1_4
C	G	1	T	1	10	T	1	B	T	2	C	A
C	G	0	T	0	0	T	0	B	T	2	C	A
N	N	N	N	N	N	N	N	A	T	2	C	A
C	G	0	T	0	0	T	0	B	A	2	T	A
C	G	0	C	1	0	T	1	B	A	0	T	C
C	G	0	T	0	0	T	0	B	N	N	N	N
G	G	1	T	0	10	A	0	B	N	N	N	C
G	N	N	N	N	N	N	N	A	N	N	N	N
G	N	N	N	N	N	N	N	A	T	2	C	A
N	N	N	N	N	N	N	N	A	A	2	T	A
C	G	0	T	0	0	T	0	B	A	0	T	C
G	G	N	N	N	N	N	N	B	N	N	N	N
C	G	0	T	0	0	T	0	B	A	2	T	A
G	C	1	T	0	10	A	0	B	N	N	N	A
G	G	1	T	0	10	A	0	A	N	N	N	N
G	G	1	T	0	10	A	0	A	A	2	T	A
G	G	1	T	0	10	A	0	B	N	N	N	N
C	G	0	C	1	0	T	1	B	N	N	N	N
C	G	0	T	0	0	T	0	A	A	2	T	A
G	C	1	T	0	10	A	0	B	N	N	N	N
G	C	1	T	0	10	A	0	A	N	N	N	N
G	G	1	T	0	10	A	0	B	T	2	C	A
G	G	1	T	0	10	A	0	B	T	2	C	A
C	G	0	C	1	0	T	1	B	N	N	N	N
C	G	0	C	1	0	T	1	A	A	2	T	A
G	G	1	T	0	10	A	0	B	A	2	T	A
C	G	0	T	0	0	T	0	X	N	N	N	N
G	G	0	T	0	10	A	0	B	T	2	C	A
C	G	0	T	0	0	T	0	B	N	N	N	N
G	G	1	T	0	10	A	0	A	N	N	N	A
C	G	0	C	1	0	T	1	B	N	N	N	N
G	G	1	T	0	10	A	0	A	N	N	N	N
G	G	0	T	0	10	A	0	B	A	2	T	A
C	G	0	C	1	0	T	1	B	N	N	N	N
G	G	0	T	0	10	A	0	B	N	N	N	N
G	G	1	T	0	10	A	0	A	A	2	T	A
N	N	N	N	N	N	N	N	A	N	N	N	N
G	G	1	T	0	10	A	0	B	N	N	N	N
G	G	1	T	0	10	A	0	A	N	N	N	N
G	G	1	T	0	10	A	0	B	N	N	N	N
G	G	1	T	0	10	A	0	A	T	2	C	A
G	G	1	T	0	10	A	0	A	T	2	C	A
C	G	0	C	1	0	T	1	B	N	N	N	N
G	C	1	T	0	10	A	0	B	A	2	T	A
G	C	1	T	0	10	A	0	B	N	N	N	N
C	G	0	C	1	0	T	1	B	N	N	N	N
C	G	0	C	1	0	T	1	A	A	2	T	A
C	G	0	T	0	0	T	0	A	A	2	T	A
C	G	0	T	0	0	T	0	B	N	N	N	N
C	G	0	T	0	0	T	0	B	N	N	N	N
N	N	N	N	N	N	N	N	A	N	N	N	A
C	G	0	T	0	0	T	0	B	N	N	N	N
C	G	0	T	0	0	T	0	A	A	2	T	A
C	G	0	T	0	0	T	0	N	N	N	N	A
C	G	0	T	0	0	T	0	A	T	2	C	A
C	G	N	N	N	N	N	N	B	N	N	N	N

C	G	0	C	1	0	T	1	A	A	0	T	C
G	C	1	T	0	10	A	0	C	A	2	T	A
C	G	0	T	0	0	T	0	X	A	2	T	A
N	N	N	N	N	N	N	N	B	A	2	T	A
C	G	0	T	0	0	T	0	B	A	2	T	A
N	N	N	N	N	N	N	N	A	T	2	C	A
C	G	0	C	1	0	T	N	A	N	N	N	N
G	G	1	T	0	10	A	0	A	A	2	T	A
G	G	1	T	0	10	A	0	A	N	N	N	N
G	G	0	T	0	10	A	0	B	N	N	N	N
C	G	0	C	1	0	T	1	B	N	N	N	N
C	G	0	C	1	0	T	1	A	A	2	T	A
C	G	0	C	1	0	T	1	X	N	N	N	N
C	G	0	C	1	0	T	1	B	N	N	N	N
C	G	0	C	1	0	T	1	A	A	2	T	A
C	G	0	T	0	0	T	0	A	A	2	T	A
C	G	0	T	0	0	T	0	B	A	2	T	A
C	G	0	T	0	0	T	0	A	N	N	N	N
C	G	0	T	0	0	T	0	A	N	N	N	N
N	N	N	N	N	N	N	N	B	A	2	T	A
C	G	0	T	0	0	T	0	A	A	2	T	A

C	G	0	T	0	0	T	0	A	A	2	T	A
C	G	0	T	0	0	T	0	B	N	N	N	N
G	G	1	T	0	10	A	0	A	N	N	N	N
C	G	0	C	1	0	T	1	B	N	N	N	N
C	G	0	T	0	0	T	0	A	N	N	N	N
N	N	N	N	N	N	N	N	A	A	2	T	A
C	G	0	T	0	1	T	0	A	N	N	N	N
G	G	1	T	0	10	A	0	B	N	N	N	N
C	G	0	C	1	0	T	1	A	N	N	N	N
C	G	0	T	0	0	T	0	B	N	N	N	N
C	G	0	T	0	0	T	0	A	N	N	N	N
N	N	N	N	N	N	N	N	B	N	N	N	N
C	G	N	N	N	N	N	N	B	N	N	N	N
G	G	1	T	0	10	A	0	B	A	2	T	A
C	G	0	T	0	0	T	0	B	A	2	T	A
G	N	N	N	N	N	N	N	A	A	2	T	A
G	G	1	T	0	10	A	0	B	A	2	T	A
C	G	0	C	1	0	T	1	B	A	2	T	A
C	G	0	T	0	0	T	0	A	N	N	N	N
N	N	N	N	N	N	N	N	A	N	N	N	N
G	C	1	T	0	10	A	0	B	A	2	T	A
C	G	0	C	1	0	T	1	A	N	N	N	N
C	G	0	T	0	1	T	0	A	N	N	N	N
G	G	0	T	0	1	T	0	B	A	2	T	A
G	C	1	T	0	10	A	0	A	N	N	N	N
C	G	0	T	0	0	T	0	X	A	2	T	A
G	G	1	T	0	10	A	0	C	A	0	T	C
C	G	0	T	0	0	T	0	B	A	2	T	A
C	G	0	T	0	1	T	0	A	N	N	N	A
C	G	0	C	1	0	T	1	B	A	2	T	A
C	G	0	C	1	0	T	1	A	A	2	T	A
C	G	0	T	0	0	T	0	B	N	N	N	N
N	N	N	N	N	N	N	N	B	A	2	T	A
C	G	0	T	0	N	N	N	A	N	2	T	C
C	G	0	C	1	0	T	1	A	A	2	T	A

C	G	0	C	1	0	T	1	A	A	2	T	A
C	G	0	C	1	0	T	1	A	A	2	T	A
C	G	0	C	1	0	T	1	B	A	2	T	A
C	G	0	T	0	0	T	0	B	A	0	T	C
G	G	0	T	0	10	A	0	A	A	2	T	A
C	G	0	T	0	0	T	0	B	A	2	T	A
G	G	0	T	0	10	A	0	B	A	2	T	A
C	G	0	T	0	0	T	0	B	N	N	N	N
C	G	0	T	0	0	T	0	B	N	N	N	N
C	G	0	T	0	0	T	0	B	A	2	T	A
G	C	1	T	0	10	A	0	B	A	2	T	A
G	G	1	T	0	10	A	0	A	T	2	C	A
C	G	0	T	0	0	T	0	A	A	0	T	C
G	G	0	T	0	10	A	0	A	N	N	N	N
G	G	1	T	0	10	A	0	A	N	N	N	N
C	G	0	T	0	0	T	0	B	N	N	N	N
C	G	0	T	0	0	T	0	B	A	2	T	A
C	G	0	T	0	0	T	0	A	A	2	T	A
C	G	0	T	0	0	T	0	B	N	N	N	N
C	G	0	C	1	0	T	N	B	T	2	C	A
C	G	0	T	0	0	T	0	A	N	N	N	N
N	N	N	N	N	N	N	N	A	N	N	N	N
G	C	1	T	0	10	A	0	B	N	N	N	N
C	G	0	T	0	0	T	0	B	T	2	C	A
G	G	0	T	0	10	A	0	B	A	2	T	A
C	G	0	T	0	0	T	0	B	A	0	T	C
C	G	0	C	1	0	T	1	B	N	N	N	N
C	G	0	C	1	0	T	1	B	N	N	N	N
C	G	0	T	0	0	T	0	A	N	N	C	A
N	N	N	N	N	N	N	N	B	N	N	N	N
C	G	0	T	0	0	T	0	A	N	N	N	N
C	G	0	C	1	0	T	1	B	T	2	C	A
N	N	N	N	N	N	N	N	B	A	2	T	A
C	G	0	C	1	0	T	1	B	T	2	C	A
G	G	1	T	0	10	A	0	X	N	N	T	A
N	N	N	N	N	N	N	N	A	A	0	T	C
C	G	0	C	1	0	T	1	A	N	N	N	N
N	N	N	N	N	N	N	N	A	A	2	T	A
C	G	0	C	1	0	T	1	A	N	N	N	N
C	G	0	T	0	0	T	0	B	A	2	T	A
C	G	0	T	0	0	T	0	B	A	2	T	A
C	G	0	T	0	0	T	0	A	N	N	N	N
G	G	1	T	0	10	A	0	B	A	2	T	A
G	G	1	T	0	10	A	0	A	T	2	C	A
C	G	0	C	1	0	T	1	B	A	0	T	C
C	G	0	T	0	0	T	0	B	A	2	T	A
C	G	0	C	1	0	T	1	B	A	2	T	A
C	G	0	C	1	0	T	1	X	T	2	C	A
C	G	0	T	0	0	T	0	B	A	2	T	A
C	N	N	N	N	N	N	N	A	N	N	N	N
G	G	0	T	0	10	A	0	B	N	N	N	N
C	G	0	C	1	0	T	1	B	N	N	N	N
N	N	N	N	N	N	N	N	A	N	N	N	N
G	G	1	T	0	10	A	0	A	A	0	T	C
C	G	0	C	1	0	T	1	B	A	2	T	A
C	G	0	T	0	0	T	0	A	A	2	T	A
C	G	0	C	1	0	T	1	A	A	0	T	C

C	G	0	C	1	0	T	1	B	N	N	N	N
G	G	1	T	0	10	A	0	B	A	2	T	A
C	G	0	T	0	0	T	0	B	N	N	N	N
N	N	N	N	N	N	N	N	B	N	N	N	N
C	G	0	C	1	0	T	1	B	A	0	T	C
C	G	0	T	0	0	T	0	A	T	2	C	A
C	G	0	T	0	0	T	0	A	A	2	T	A
G	C	1	T	0	10	A	0	B	A	2	T	A
G	G	1	T	0	10	A	0	A	T	2	C	A
G	G	1	T	0	10	A	0	B	N	N	N	N
G	G	0	T	0	10	A	0	B	A	0	T	C
N	N	N	N	N	N	N	N	A	N	N	N	N
G	C	1	T	0	10	A	0	B	N	N	N	N
C	G	0	C	1	0	T	1	A	T	2	C	A
C	G	0	C	1	0	T	1	A	A	2	T	A
C	G	0	C	1	0	T	1	A	A	2	T	A
N	N	N	N	N	N	N	N	B	A	2	T	A
G	G	0	T	0	10	A	0	B	N	N	N	N
C	G	0	C	1	0	T	1	B	N	N	N	N
C	G	0	T	0	0	T	0	A	N	N	N	N
C	G	0	C	1	0	T	1	B	N	N	N	N
C	G	0	T	0	0	T	0	A	A	2	T	A
C	G	0	C	1	0	T	1	B	N	N	N	N
G	G	1	T	0	10	A	0	B	T	2	C	A
N	N	N	N	N	N	N	N	A	N	N	N	N
N	N	N	N	N	N	N	N	X	N	N	N	N
C	G	0	C	1	0	T	1	B	A	2	T	A
C	G	0	T	0	1	T	0	B	A	2	T	A
C	G	0	T	0	0	T	0	B	N	N	N	N
C	G	0	T	0	0	T	0	B	A	2	T	A
G	G	1	T	0	10	A	0	A	T	2	C	A
G	G	1	T	0	10	A	0	A	T	2	C	A
G	G	0	T	0	10	A	0	B	T	2	C	A
C	G	0	T	0	0	T	0	A	A	2	T	A
C	G	0	T	0	0	T	0	A	A	2	T	A
G	G	0	T	0	10	A	0	B	T	2	C	A
G	G	N	N	N	N	N	N	A	N	N	N	N
G	G	1	T	0	10	A	0	B	T	2	C	A
C	G	0	C	1	0	T	1	A	T	2	C	A
C	G	0	T	0	0	T	0	B	A	2	T	A
G	G	1	T	0	10	A	0	B	A	2	T	A
C	G	0	T	0	0	T	0	A	A	2	T	A
C	G	0	C	1	0	T	1	B	N	N	N	N
C	N	N	N	N	N	N	N	B	N	N	N	N
N	N	N	N	N	N	N	N	A	N	N	N	N
C	G	0	C	1	0	T	1	B	A	2	T	A
N	N	N	N	N	N	N	N	B	N	N	N	N
C	G	0	T	0	0	T	0	B	A	2	T	A
C	N	N	N	N	N	N	N	B	N	N	N	N
C	G	0	T	0	0	T	0	A	A	2	T	A
C	G	0	T	0	0	T	0	A	A	0	T	C
C	G	0	C	1	0	T	1	A	N	N	N	N
G	G	1	T	0	10	A	0	B	A	2	T	A
G	G	1	T	0	10	A	0	B	A	2	T	A
C	G	0	T	0	0	T	0	A	A	2	T	A
G	G	1	T	0	10	A	0	A	N	N	N	N
G	C	1	T	0	10	A	0	A	N	N	N	N

C	G	0	T	0	0	T	0	B	N	N	N	N
N	N	N	N	N	N	N	N	B	N	N	N	N
G	C	1	T	0	10	A	0	B	N	N	N	N
C	G	0	C	1	0	T	1	B	N	N	N	N
G	C	1	T	0	10	A	0	A	T	2	C	A
C	G	0	C	1	0	T	1	A	N	N	N	A
G	G	0	T	0	10	A	0	B	N	N	N	A
C	G	0	C	1	0	T	1	B	N	N	N	N
C	G	0	C	1	0	T	1	B	A	0	T	C
N	N	N	N	N	N	N	N	A	T	2	C	A
N	N	N	N	N	N	N	N	A	A	0	T	C
C	G	0	T	0	0	T	0	B	N	N	N	N
C	G	0	T	0	0	T	0	A	A	2	T	A
C	G	0	T	0	0	T	0	A	N	2	T	C
G	G	0	T	0	10	A	0	A	T	2	C	A
C	G	0	C	1	0	T	1	B	N	N	N	N
C	G	0	C	1	0	T	1	B	N	N	N	N
G	C	1	T	0	10	A	0	B	A	2	T	A
C	G	0	T	0	0	T	0	X	N	N	N	N
C	G	0	T	0	0	T	0	B	A	0	T	C
G	C	1	T	0	10	A	0	X	A	2	T	A
C	G	0	C	1	0	T	1	B	A	0	T	C
C	G	0	T	0	0	T	0	B	A	2	T	A
C	G	0	T	0	0	T	0	B	N	N	N	N
C	G	0	T	0	0	T	0	A	A	2	T	A
N	N	N	N	N	N	N	N	B	N	N	T	A
G	C	1	T	0	10	A	0	B	A	2	T	A
C	G	0	C	1	0	T	1	A	A	2	T	A
C	G	0	C	1	0	T	1	A	A	2	T	A
C	G	0	T	0	0	T	0	A	N	N	N	N
G	G	1	T	0	10	A	0	A	T	2	C	A
C	G	0	T	0	0	T	0	B	A	2	T	A
G	G	1	T	0	10	A	0	A	A	2	T	A
G	G	1	T	0	10	A	0	A	T	2	C	A
N	N	N	N	N	N	N	N	A	N	N	N	N
C	G	0	T	0	0	T	0	A	N	N	N	N
C	G	0	T	0	0	T	0	B	A	2	T	A
C	G	0	T	0	0	T	0	B	A	2	T	A
G	C	1	T	0	10	A	0	A	A	2	T	A
G	C	1	T	0	10	A	0	A	A	2	T	A
G	C	1	T	0	10	A	0	A	A	2	T	A
N	N	N	N	N	N	N	N	B	N	N	N	N
G	C	1	T	0	10	A	0	B	A	2	T	A
G	C	1	T	0	10	A	0	B	A	2	T	A
G	G	1	T	0	10	A	0	A	N	N	N	N
G	C	1	T	0	10	A	0	A	N	N	N	N
G	C	1	T	0	10	A	0	A	A	2	T	A
C	G	0	C	1	0	T	1	B	N	N	N	N
C	G	0	C	1	0	T	1	B	A	2	T	A
G	G	1	T	0	10	A	0	B	N	N	N	N
C	G	0	C	1	0	T	1	B	N	N	N	N
C	G	0	C	1	0	T	N	N	N	N	N	N
C	G	0	T	0	0	T	0	A	N	N	N	N
C	G	0	T	0	0	T	0	B	A	2	T	A
C	G	0	T	0	0	T	N	A	A	0	T	C
N	N	N	N	N	N	N	N	A	N	N	N	N

AL1_5	AL1_6	AL1_7	AL1_8	AL1_9	AL1_10	AL1_11	AL1_12	AL1_13	AL1_14	AL1_15	AL1_16
G	72	17	N	N	N	N	G	C	A	T	G
G	72	N	N	N	N	N	G	C	A	T	G
G	72	N	N	N	N	N	G	C	A	T	G
A	0	0	A	G	G	C	A	T	G	G	A
G	6	17	C	A	T	C	A	T	G	T	A
N	N	N	N	N	N	N	N	N	N	N	N
G	6	17	C	G	T	T	G	T	G	T	A
N	N	N	N	N	N	N	N	N	N	N	N
G	72	N	-	-	-	-	G	C	A	T	G
A	0	0	A	G	G	C	A	T	G	G	A
A	0	0	A	G	G	C	A	T	G	G	A
N	N	N	N	N	N	N	N	N	N	N	N
A	0	0	A	G	G	C	A	T	G	G	A
G	6	17	C	A	T	C	A	T	G	T	A
N	N	N	N	N	N	N	N	N	N	N	N
-	N	17	C	A	T	C	A	T	G	G	A
N	N	N	N	N	N	N	N	N	N	N	N
N	N	N	N	N	N	N	N	N	N	N	N
A	0	0	A	G	G	C	A	T	G	G	A
N	N	N	N	N	N	N	N	N	N	N	N
N	N	N	N	N	N	N	N	N	N	N	N
G	72	N	-	-	-	-	G	C	A	T	G
G	72	N	-	-	-	-	G	C	A	T	G
N	N	N	N	N	N	N	N	N	N	N	N
A	0	0	A	G	G	C	A	T	G	G	A
A	0	0	A	G	G	C	A	T	G	G	A
N	N	N	N	N	N	N	N	N	N	N	N
G	72	N	-	-	-	-	G	C	A	T	G
N	N	N	N	N	N	N	N	N	N	N	N
G	72	N	-	-	-	-	G	C	A	T	G
N	N	N	N	N	N	N	N	N	N	N	N
N	N	N	N	N	N	N	N	N	N	N	N
A	0	0	A	G	G	C	A	T	G	G	A
G	6	17	C	G	T	T	G	T	G	T	A
N	N	N	N	N	N	N	N	N	N	N	N
A	0	0	A	G	G	C	A	T	G	G	A
N	N	N	N	N	N	N	N	N	N	N	N
N	N	N	N	N	N	N	N	N	N	N	N
N	N	N	N	N	N	N	N	N	N	N	N
N	N	N	N	N	N	N	N	N	N	N	N
G	72	N	-	-	-	-	G	C	A	T	G
G	72	N	-	-	-	-	G	C	A	T	G
N	N	N	N	N	N	N	N	N	N	N	N
A	0	0	A	G	G	C	A	T	G	G	A
N	N	N	N	N	N	N	N	N	N	N	N
N	N	N	N	N	N	N	N	N	N	N	N
A	0	0	A	G	G	C	A	T	G	G	A
A	0	0	A	G	G	C	A	T	G	G	A
N	N	N	N	N	N	N	N	N	N	N	N
N	N	N	N	N	N	N	N	N	N	N	N
A	0	0	A	G	G	C	A	T	G	G	A
N	N	N	N	N	N	N	N	N	N	N	N
A	0	0	A	G	G	C	A	T	G	G	A
G	72	N	-	-	-	-	G	C	A	T	G
G	72	N	-	-	-	-	G	C	A	T	G
N	N	N	N	N	N	N	N	N	N	N	N

G	6	17	C	A	T	C	A	T	G	T	A
A	0	0	A	G	G	C	A	T	G	G	A
A	0	0	A	G	G	C	A	T	G	G	A
A	0	0	A	G	G	C	A	T	G	G	A
A	0	0	A	G	G	C	A	T	G	G	A
G	72	N	-	-	-	-	G	C	A	T	G
N	N	N	N	N	N	N	N	N	N	N	N
A	0	0	A	G	G	C	A	T	G	G	A
N	N	N	N	N	N	N	N	N	N	N	N
N	N	N	N	N	N	N	N	N	N	N	N
N	N	N	N	N	N	N	N	N	N	N	N
A	0	0	A	G	G	C	A	T	G	G	A
N	N	N	N	N	N	N	N	N	N	N	N
N	N	N	N	N	N	N	N	N	N	N	N
A	0	0	A	G	G	C	A	T	G	G	A
A	0	0	A	G	G	C	A	T	G	G	A
A	0	0	A	G	G	C	A	T	G	G	A
N	N	N	N	N	N	N	N	N	N	N	N
G	6	17	C	G	T	T	G	T	G	T	A
A	0	0	A	G	G	C	A	T	G	G	A
A	0	0	A	G	G	C	A	T	G	G	A

A	0	0	A	G	G	C	A	T	G	G	A
N	N	N	N	N	N	N	N	N	N	N	N
N	N	N	N	N	N	N	N	N	N	N	N
G	6	17	C	G	T	T	G	T	G	T	A
N	N	N	N	N	N	N	N	N	N	N	N
A	0	0	A	G	G	C	A	T	G	G	A
N	N	N	N	N	N	N	N	N	N	N	N
N	N	N	N	N	N	N	N	N	N	N	N
N	N	N	N	N	N	N	N	N	N	N	N
N	N	N	N	N	N	N	N	N	N	N	N
N	N	N	C	G	T	T	G	T	G	T	A
N	N	N	N	N	N	N	N	N	N	N	N
N	N	N	N	N	N	N	N	N	N	N	N
A	0	0	A	G	G	C	A	T	G	G	A
A	0	0	A	G	G	C	A	T	G	G	A
G	6	17	C	A	T	C	A	T	G	T	A
A	0	0	A	G	G	C	A	T	G	G	A
A	0	0	A	G	G	C	A	T	G	G	A
N	N	N	N	N	N	N	N	N	N	N	N
N	N	N	N	N	N	N	N	N	N	N	N
A	0	0	A	G	G	C	A	T	G	G	A
N	N	N	N	N	N	N	N	N	N	N	N
G	6	17	C	G	T	T	G	T	G	T	A
A	0	0	A	G	G	C	A	T	G	G	A
N	N	N	N	N	N	N	N	N	N	N	N
A	0	0	A	G	G	C	A	T	G	G	A
G	6	17	C	G	T	T	G	T	G	T	A
A	0	0	A	G	G	C	A	T	G	G	A
A	0	0	A	G	G	C	A	T	G	G	A
A	0	0	A	G	G	C	A	T	G	G	A
A	0	0	A	G	G	C	A	T	G	G	A
N	N	N	N	N	N	N	N	N	N	N	N
A	0	0	A	G	G	C	A	T	G	G	A
G	6	17	C	A	T	N	N	N	N	N	N
A	0	0	A	G	G	C	A	T	G	G	A

A	0	0	A	G	G	C	A	T	G	G	A
A	0	0	A	G	G	C	A	T	G	G	A
A	0	0	A	G	G	C	A	T	G	G	A
G	6	17	C	A	T	C	A	T	G	T	A
G	6	17	C	A	T	C	A	T	G	G	A
A	0	0	A	G	G	C	A	T	G	G	A
A	0	0	A	G	G	C	A	T	G	G	A
N	N	N	N	N	N	N	N	N	N	N	N
G	6	17	C	G	T	T	G	T	G	T	A
A	0	0	A	G	G	C	A	T	G	G	A
A	0	0	A	G	G	C	A	T	G	G	A
G	72	N	-	-	-	-	G	C	A	T	G
A	0	0	A	G	G	C	A	T	G	G	A
N	N	N	N	N	N	N	N	N	N	N	N
N	N	N	N	N	N	N	N	N	N	N	N
N	N	N	N	N	N	N	N	N	N	N	N
A	0	0	A	G	G	C	A	T	G	G	A
A	0	0	A	G	G	C	A	T	G	G	A
N	N	N	N	N	N	N	N	N	N	N	N
G	72	N	-	-	-	-	G	C	A	T	G
N	N	N	N	N	N	N	N	N	N	N	N
N	N	N	N	N	N	N	N	N	N	N	N
N	N	N	N	N	N	N	N	N	N	N	N
G	72	N	-	-	-	-	G	C	A	T	G
A	0	0	A	G	G	C	A	T	G	G	A
G	6	17	C	A	T	C	A	T	G	T	A
N	N	N	N	N	N	N	N	N	N	N	N
N	N	N	N	N	N	N	N	N	N	N	N
G	72	N	-	-	-	-	G	C	A	T	G
N	N	N	N	N	N	N	N	N	N	N	N
N	N	N	N	N	N	N	N	N	N	N	N
G	72	N	-	-	-	-	G	C	A	T	G
A	0	0	A	G	G	C	A	T	G	G	A
G	72	N	-	-	-	-	G	C	A	T	G
A	0	0	A	G	G	C	A	T	G	G	A
G	6	17	C	A	T	C	A	T	G	T	A
N	N	N	N	N	N	N	N	N	N	N	N
A	N	N	N	N	N	N	N	N	N	N	N
N	N	N	N	N	N	N	N	N	N	N	N
A	0	0	A	G	G	C	A	T	G	G	A
A	0	0	A	G	G	C	A	T	G	G	A
N	N	N	N	N	N	N	N	N	N	N	N
A	0	0	A	G	G	C	A	T	G	G	A
G	72	N	-	-	-	-	G	C	A	T	G
G	6	17	C	G	T	T	G	T	G	T	A
A	0	0	A	G	G	C	A	T	G	G	A
A	0	0	A	G	G	C	A	T	G	G	A
G	72	N	-	-	-	-	G	C	A	T	G
A	0	0	A	G	G	C	A	T	G	G	A
G	6	18	C	G	T	T	G	T	G	T	A
N	N	N	N	N	N	N	N	N	N	N	N
N	N	N	N	N	N	N	N	N	N	N	N
N	N	N	N	N	N	N	N	N	N	N	N
A	0	0	A	G	G	C	A	T	G	G	A
A	0	0	A	G	G	C	A	T	G	G	A
A	0	0	A	G	G	C	A	T	G	G	A
A	0	0	A	G	G	C	A	T	G	G	A

N	N	N	N	N	N	N	N	N	N	N	N
A	0	0	A	G	G	C	A	T	G	G	A
N	N	N	N	N	N	N	N	N	N	N	N
G	6	17	C	A	T	C	A	T	G	T	A
G	6	17	C	A	T	C	A	T	G	T	A
G	72	N	-	-	-	-	G	C	A	T	G
A	0	0	A	G	G	C	A	T	G	G	A
A	0	0	A	G	G	C	A	T	G	G	A
G	72	N	-	-	-	-	G	C	A	T	G
G	6	17	C	G	T	T	G	T	G	T	A
G	6	17	C	A	T	C	A	T	G	T	A
N	N	N	N	N	N	N	N	N	N	N	N
N	N	N	N	N	N	N	N	N	N	N	N
G	72	N	-	-	-	-	G	C	A	T	G
A	0	0	A	G	G	C	A	T	G	G	A
A	0	0	A	G	G	C	A	T	G	G	A
A	0	0	A	G	G	C	A	T	G	G	A
-	N	17	C	G	T	T	G	T	G	T	A
N	N	N	N	N	N	N	N	N	N	N	N
N	N	N	N	N	N	N	N	N	N	N	N
G	72	N	-	-	-	-	G	C	A	T	G
A	0	0	A	G	G	C	A	T	G	G	A
N	N	N	N	N	N	N	N	N	N	N	N
G	72	N	-	-	-	-	G	C	A	T	G
N	N	N	N	N	N	N	N	N	N	N	N
N	N	N	N	N	N	N	N	N	N	N	N
A	0	0	A	G	G	C	A	T	G	G	A
A	0	0	A	G	G	C	A	T	G	G	A
N	N	N	N	N	N	N	N	N	N	N	N
A	0	0	A	G	G	C	A	T	G	G	A
G	72	N	-	-	-	-	G	C	A	T	G
G	72	N	-	-	-	-	G	C	A	T	G
G	72	N	-	-	-	-	G	C	A	T	G
A	0	0	A	G	G	C	A	T	G	G	A
A	0	0	A	G	G	C	A	T	G	G	A
G	72	N	-	-	-	-	G	C	A	T	G
N	N	N	N	N	N	N	N	N	N	N	N
G	72	N	-	-	-	-	G	C	A	T	G
G	72	N	-	-	-	-	G	C	A	T	G
A	0	0	A	G	G	C	A	T	G	G	A
A	0	0	A	G	G	C	A	T	G	G	A
A	0	0	A	G	G	C	A	T	G	G	A
N	N	N	N	N	N	N	N	N	N	N	N
N	N	N	N	N	N	N	N	N	N	N	N
N	N	N	N	N	N	N	N	N	N	N	N
A	0	0	A	G	G	C	A	T	G	G	A
N	N	N	N	N	N	N	N	N	N	N	N
A	0	0	A	G	G	C	A	T	G	G	A
G	6	17	C	G	T	T	G	T	G	T	A
A	0	0	A	G	G	C	A	T	G	G	A
G	6	17	C	G	T	T	G	T	G	T	A
N	N	N	N	N	N	N	N	N	N	N	N
A	0	0	A	G	G	C	A	T	G	G	A
A	0	0	A	G	G	C	A	T	G	G	A
A	0	0	A	G	G	C	A	T	G	G	A
N	N	N	N	N	N	N	N	N	N	N	N
N	N	N	N	N	N	N	N	N	N	N	N

N	N	N	N	N	N	N	N	N	N	N	N
N	N	N	N	N	N	N	N	N	N	N	N
N	N	N	N	N	N	N	N	N	N	N	N
N	N	N	N	N	N	N	N	N	N	N	N
G	72	N	-	-	-	-	G	C	A	T	G
A	0	0	A	G	G	C	A	T	G	G	A
G	6	17	C	A	T	C	A	T	G	T	A
N	N	N	N	N	N	N	N	N	N	N	N
A	0	0	A	G	G	C	A	T	G	G	A
G	72	N	-	-	-	-	G	C	A	T	G
A	0	0	A	G	G	C	A	T	G	G	A
N	N	N	N	N	N	N	N	N	N	N	N
A	0	0	A	G	G	C	A	T	G	G	A
G	6	17	C	G	T	T	G	T	G	T	A
G	72	N	-	-	-	-	G	C	A	T	G
N	N	N	N	N	N	N	N	N	N	N	N
N	N	N	N	N	N	N	N	N	N	N	N
A	0	0	A	G	G	C	A	T	G	G	A
N	N	N	N	N	N	N	N	N	N	N	N
G	6	17	C	A	T	C	A	T	G	T	A
A	0	0	A	G	G	C	A	T	G	G	A
A	0	0	A	G	G	C	A	T	G	G	A
A	0	0	A	G	G	C	A	T	G	G	A
N	N	N	N	N	N	N	N	N	N	N	N
A	0	0	A	G	G	C	A	T	G	G	A
A	0	0	A	G	G	C	A	T	G	G	A
A	0	0	A	G	G	C	A	T	G	G	A
A	0	0	A	G	G	C	A	T	G	G	A
A	0	0	A	G	G	C	A	T	G	G	A
N	N	N	N	N	N	N	N	N	N	N	N
G	72	N	-	-	-	-	G	C	A	T	G
A	0	0	A	G	G	C	A	T	G	G	A
G	6	17	C	G	C	C	A	T	G	G	A
G	72	N	-	-	-	-	G	C	A	T	G
G	6	17	C	A	T	C	A	T	G	G	A
N	N	N	N	N	N	N	N	N	N	N	N
A	0	0	A	G	G	C	A	T	G	G	A
A	0	0	A	G	G	C	A	T	G	G	A
A	0	0	A	G	G	C	A	T	G	G	A
A	0	0	A	G	G	C	A	T	G	G	A
A	0	0	A	G	G	C	A	T	G	G	A
N	N	N	N	N	N	N	N	N	N	N	N
A	0	0	A	G	G	C	A	T	G	G	A
A	0	0	A	G	G	C	A	T	G	G	A
N	N	N	N	N	N	N	N	N	N	N	N
G	6	17	C	G	T	T	G	T	G	T	A
A	0	0	A	G	G	C	A	T	G	G	A
N	N	N	N	N	N	N	N	N	N	N	N
A	0	0	A	G	G	C	A	T	G	G	A
N	N	N	N	N	N	N	N	N	N	N	N
A	0	0	A	G	G	C	A	T	G	G	A
G	6	17	C	G	T	T	G	T	G	T	A
N	N	N	N	N	N	N	N	N	N	N	N
A	0	0	A	G	G	C	A	T	G	G	A
A	0	0	A	G	G	C	A	T	G	G	A
N	N	N	N	N	N	N	N	N	N	N	N

AL1_17	AL1_18	AL1_19	AL1_20	AL1_21	AL1_22	AL1_23	AL1_24	AL1_25	AL1_26	AL1_27
A	C	G	T	T	2	A	G	T	4	A
A	C	G	T	T	0	A	G	T	4	A
A	C	G	T	T	0	A	G	T	4	A
G	T	A	A	A	2	C	C	C	0	G
G	T	G	T	T	0	A	G	T	4	A
N	N	N	N	N	N	N	N	N	N	N
G	T	G	T	T	0	A	G	C	0	A
N	N	N	N	N	N	N	N	N	N	N
A	C	G	T	T	0	A	G	T	N	N
G	T	A	A	A	2	C	C	C	0	G
G	T	A	A	A	2	C	C	C	0	G
N	N	N	N	N	N	N	N	N	N	N
G	T	A	A	A	2	C	C	C	0	G
G	T	G	T	T	0	A	G	T	4	A
N	N	N	N	N	N	N	N	N	N	N
G	T	G	T	T	0	A	G	C	0	G
N	N	N	N	N	N	N	N	N	N	N
N	N	N	N	N	N	N	N	N	N	N
G	T	A	A	A	2	C	C	C	0	G
N	N	N	N	N	N	N	N	N	N	N
N	N	N	N	N	N	N	N	N	N	N
A	C	G	T	T	0	A	G	T	4	A
A	C	G	T	T	0	A	G	T	4	A
N	N	N	N	N	N	N	N	N	N	N
G	T	A	A	A	2	C	C	C	0	G
G	T	A	A	A	2	C	C	C	0	G
N	N	N	N	N	N	N	N	N	N	N
A	C	G	T	T	0	A	G	T	4	A
N	N	N	N	N	N	N	N	N	N	N
A	C	G	T	T	0	A	G	T	4	A
N	N	N	N	N	N	N	N	N	N	N
N	N	N	N	N	N	N	N	N	N	N
G	T	A	A	A	2	C	C	C	0	G
G	T	G	T	T	0	A	G	C	0	A
N	N	N	N	N	N	N	N	N	N	N
G	T	A	A	A	2	C	C	C	0	G
N	N	N	N	N	N	N	N	N	N	N
N	N	N	N	N	N	N	N	N	N	N
N	N	N	N	N	N	N	N	N	N	N
N	N	N	N	N	N	N	N	N	N	N
A	C	G	T	T	0	A	G	T	4	A
A	C	G	T	T	0	A	G	T	4	A
N	N	N	N	N	N	N	N	N	N	N
G	T	A	A	A	2	C	C	C	0	G
N	N	N	N	N	N	N	N	N	N	N
N	N	N	N	N	N	N	N	N	N	N
G	T	A	A	A	2	C	C	C	0	G
G	T	A	A	A	2	C	C	C	0	G
N	N	N	N	N	N	N	N	N	N	N
N	N	N	N	N	N	N	N	N	N	N
G	T	A	A	A	2	C	C	C	0	G
N	N	N	N	N	N	N	N	N	N	N
G	T	A	A	A	2	C	C	C	0	G
A	C	G	T	T	0	A	G	T	4	A
A	C	G	T	T	0	A	G	T	4	A
N	N	N	N	N	N	N	N	N	N	N

G	T	G	T	T	0	A	G	T	4	A
G	T	A	A	A	2	C	C	C	0	N
G	T	A	A	A	2	C	C	C	0	G
G	T	A	N	N	N	N	N	N	N	N
G	T	A	A	A	2	C	C	C	0	G
A	C	G	T	T	0	A	G	T	4	A
N	N	N	N	N	N	N	N	N	N	N
G	T	A	A	A	2	C	C	C	0	G
N	N	N	N	N	N	N	N	N	N	N
N	N	N	N	N	N	N	N	N	N	N
N	N	N	N	N	N	N	N	N	N	N
G	T	A	A	A	2	C	C	C	0	G
N	N	N	N	N	N	N	N	N	N	N
N	N	N	N	N	N	N	N	N	N	N
G	T	A	A	A	2	C	C	C	0	G
G	T	A	A	A	2	C	C	C	0	G
G	T	A	A	A	2	C	C	C	0	G
N	N	N	N	N	N	N	N	N	N	N
G	T	G	T	T	0	A	G	C	0	A
G	T	A	A	A	2	C	C	C	0	G
G	T	A	A	A	2	C	C	C	0	G

G	T	A	T	T	0	A	G	T	4	A
N	N	N	N	N	N	N	N	N	N	N
N	N	N	N	N	N	N	N	N	N	N
G	T	G	T	T	0	A	G	C	0	A
N	N	N	N	N	N	N	N	N	N	N
G	T	A	A	A	2	C	C	C	0	G
N	N	N	N	N	N	N	N	N	N	N
N	N	N	N	N	N	N	N	N	N	N
N	N	N	N	N	N	N	N	N	N	N
N	N	N	N	N	N	N	N	N	N	N
G	T	G	T	T	0	A	G	C	0	A
N	N	N	N	N	N	N	N	N	N	N
N	N	N	N	N	N	N	N	N	N	N
G	T	A	A	A	2	C	C	C	0	G
G	T	A	A	A	2	C	C	C	0	G
G	T	G	T	T	0	A	G	T	4	A
G	T	A	A	A	2	C	C	C	0	G
G	T	A	A	A	2	C	C	C	0	G
N	N	N	N	N	N	N	N	N	N	N
N	N	N	N	N	N	N	N	N	N	N
G	T	A	A	A	2	C	C	C	0	G
N	N	N	N	N	N	N	N	N	N	N
G	T	G	T	T	0	A	G	C	0	A
G	T	A	A	A	2	C	C	C	0	G
N	N	N	N	N	N	N	N	N	N	N
G	T	A	A	A	2	C	C	C	0	G
G	T	G	T	T	0	A	G	C	0	A
G	T	A	A	A	2	C	C	C	0	G
G	T	A	A	A	2	C	C	C	0	G
G	T	A	A	A	2	C	C	C	0	G
G	T	A	A	A	2	C	C	C	0	G
N	N	N	N	N	N	N	N	N	N	N
G	T	A	A	A	2	C	C	C	0	G
N	N	N	N	N	N	N	N	N	N	N
G	T	A	A	A	2	C	C	C	0	G

G	T	A	A	A	2	C	C	C	0	G
G	T	A	A	A	2	C	C	C	0	G
G	T	A	A	A	2	C	C	C	0	G
G	T	G	T	T	0	A	G	T	4	A
G	T	G	T	T	0	A	G	C	0	G
G	T	A	A	A	2	C	C	C	0	G
G	T	A	A	A	2	C	C	C	0	G
N	N	N	N	N	N	N	N	N	N	N
G	T	G	T	T	0	A	G	C	0	A
G	T	A	A	A	2	C	C	C	0	G
G	T	A	A	A	2	C	C	C	0	G
A	C	G	T	T	0	A	G	T	4	A
G	T	A	A	A	2	C	C	C	0	G
N	N	N	N	N	N	N	N	N	N	N
N	N	N	N	N	N	N	N	N	N	N
N	N	N	N	N	N	N	N	N	N	N
G	T	A	A	A	2	C	C	C	0	G
G	T	A	A	A	2	C	C	C	0	G
N	N	N	N	N	N	N	N	N	N	N
A	C	G	T	T	0	A	G	T	4	A
N	N	N	N	N	N	N	N	N	N	N
N	N	N	N	N	N	N	N	N	N	N
N	N	N	N	N	N	N	N	N	N	N
A	C	G	T	T	0	A	G	T	4	A
G	T	A	A	A	2	C	C	C	0	G
G	T	G	T	T	0	A	G	T	4	A
N	N	N	N	N	N	N	N	N	N	N
N	N	N	N	N	N	N	N	N	N	N
A	C	G	T	T	0	A	G	T	4	A
N	N	N	N	N	N	N	N	N	N	N
N	N	N	N	N	N	N	N	N	N	N
A	C	G	T	T	0	A	G	T	4	A
G	T	A	A	A	2	C	C	C	0	G
A	C	G	T	T	0	A	G	T	4	A
G	T	N	N	N	N	N	N	N	N	N
G	T	G	T	T	0	A	G	T	4	A
N	N	N	N	N	N	N	N	N	N	N
N	N	N	N	N	N	N	N	N	N	N
N	N	N	N	N	N	N	N	N	N	N
G	T	A	A	A	2	C	C	C	0	G
G	T	A	A	A	2	C	C	C	0	G
N	N	N	N	N	N	N	N	N	N	N
G	T	A	A	A	2	C	C	C	0	G
A	C	G	T	T	0	A	G	T	4	A
G	T	G	T	T	0	A	G	T	4	A
G	T	A	A	A	2	C	C	C	0	G
G	T	A	A	A	2	C	C	C	0	G
A	C	G	T	T	0	A	G	T	4	A
G	T	A	A	A	2	N	N	N	N	N
G	T	G	T	T	0	A	G	C	0	A
N	N	N	N	N	N	N	N	N	N	N
N	N	N	N	N	N	N	N	N	N	N
N	N	N	N	N	N	N	N	N	N	N
G	T	A	A	A	2	C	C	C	0	G
G	T	A	A	A	2	C	C	C	0	G
G	T	A	A	A	2	C	C	C	0	G
G	T	A	A	A	2	C	C	C	0	G

N	N	N	N	N	N	N	N	N	N	N
G	T	A	A	A	2	C	C	C	0	G
N	N	N	N	N	N	N	N	N	N	N
G	T	G	T	T	0	A	G	T	4	A
G	T	G	T	T	0	A	G	T	4	A
A	C	G	T	T	0	A	G	T	4	A
G	T	A	A	A	2	C	C	C	0	G
G	T	A	A	A	2	C	C	C	0	G
A	C	G	T	T	0	A	G	T	4	A
G	T	G	T	T	0	A	G	C	0	A
G	T	G	T	T	0	A	G	T	4	A
N	N	N	N	N	N	N	N	N	N	N
N	N	N	N	N	N	N	N	N	N	N
A	C	G	T	T	0	A	G	T	4	A
G	T	A	A	A	2	C	C	C	0	G
G	T	A	A	A	2	C	C	C	0	G
G	T	A	A	A	2	C	C	C	0	G
G	T	G	T	T	0	A	G	C	0	A
N	N	N	N	N	N	N	N	N	N	N
N	N	N	N	N	N	N	N	N	N	N
A	C	G	T	T	0	A	G	T	4	A
G	T	A	A	A	2	C	C	C	0	G
N	N	N	N	N	N	N	N	N	N	N
A	C	G	T	T	0	A	G	T	4	A
N	N	N	N	N	N	N	N	N	N	N
N	N	N	N	N	N	N	N	N	N	N
G	T	A	A	A	2	C	C	C	0	G
G	T	A	A	A	2	C	C	C	0	G
N	N	N	N	N	N	N	N	N	N	N
G	T	A	A	A	2	C	C	C	0	G
A	C	G	T	T	0	A	G	T	4	A
A	C	G	T	T	0	A	G	T	4	A
A	C	G	T	T	0	A	G	T	4	A
G	T	A	A	A	2	C	C	C	0	G
G	T	A	A	A	2	C	C	C	0	G
A	C	G	T	T	0	A	G	T	4	A
N	N	N	N	N	N	N	N	N	N	N
A	C	G	T	T	0	A	G	T	4	A
A	C	G	T	T	0	A	G	T	4	A
G	T	A	A	A	2	C	C	C	0	G
G	T	A	A	A	2	C	C	C	0	G
G	T	A	A	A	2	C	C	C	0	G
N	N	N	N	N	N	N	N	N	N	N
N	N	N	N	N	N	N	N	N	N	N
N	N	N	N	N	N	N	N	N	N	N
G	T	A	A	A	2	C	C	C	0	G
N	N	N	N	N	N	N	N	N	N	N
G	T	A	A	A	2	C	C	C	0	G
G	T	G	T	T	0	A	G	C	0	A
G	T	A	A	A	2	C	C	C	0	G
G	T	G	T	T	0	A	G	C	0	A
N	N	N	N	N	N	N	N	N	N	N
G	T	A	A	A	2	C	C	C	0	G
G	T	A	A	A	2	C	C	C	0	G
G	T	A	A	A	2	C	C	C	0	G
N	N	N	N	N	N	N	N	N	N	N
N	N	N	N	N	N	N	N	N	N	N

N	N	N	N	N	N	N	N	N	N	N
N	N	N	N	N	N	N	N	N	N	N
N	N	N	N	N	N	N	N	N	N	N
N	N	N	N	N	N	N	N	N	N	N
A	C	G	T	T	0	A	G	T	4	A
G	T	A	A	A	2	C	C	C	0	G
G	T	G	T	T	0	A	G	T	4	A
N	N	N	N	N	N	N	N	N	N	N
G	T	A	A	A	2	C	C	C	0	G
A	C	G	T	T	0	A	G	T	4	A
G	T	A	A	A	2	C	C	C	0	G
N	N	N	N	N	N	N	N	N	N	N
G	T	A	A	A	2	C	C	C	0	G
G	T	G	T	T	0	A	G	C	0	A
A	C	G	T	T	0	A	G	T	4	A
N	N	N	N	N	N	N	N	N	N	N
N	N	N	N	N	N	N	N	N	N	N
G	T	A	A	A	2	C	C	C	0	G
N	N	N	N	N	N	N	N	N	N	N
G	T	G	T	T	0	A	G	T	4	A
G	T	A	A	A	2	C	C	C	0	G
G	T	A	A	A	2	C	C	C	0	G
G	T	A	A	A	2	C	C	C	0	G
N	N	N	N	N	N	N	N	N	N	N
G	T	A	A	A	2	C	C	C	0	G
G	T	A	A	A	2	C	C	C	0	G
G	T	A	A	A	2	C	C	C	0	G
G	T	A	A	A	2	C	C	C	0	G
G	T	A	A	A	2	C	C	C	0	G
N	N	N	N	N	N	N	N	N	N	N
A	C	G	T	T	0	A	G	T	4	A
G	T	A	A	A	2	C	C	C	0	G
G	T	G	T	T	0	A	G	T	4	A
A	C	G	T	T	0	A	G	T	4	A
G	T	A	A	A	2	C	C	C	0	G
N	N	N	N	N	N	N	N	N	N	N
G	T	A	A	A	2	C	C	C	0	G
G	T	A	A	A	2	C	C	C	0	G
G	T	A	A	A	2	C	C	C	0	G
G	T	A	A	A	2	C	C	C	0	G
G	T	A	A	A	2	C	C	C	0	G
N	N	N	N	N	N	N	N	N	N	N
G	T	A	A	A	2	C	C	C	0	G
G	T	A	A	A	2	C	C	C	0	G
N	N	N	N	N	N	N	N	N	N	N
G	T	G	T	T	0	A	G	C	0	A
G	T	A	A	A	2	C	C	C	0	G
N	N	N	N	N	N	N	N	N	N	N
G	T	A	A	A	2	C	C	C	0	G
N	N	N	N	N	N	N	N	N	N	N
G	T	A	A	A	2	C	C	C	0	G
G	T	G	T	T	0	A	G	C	0	A
N	N	N	N	N	N	N	N	N	N	N
G	T	A	A	A	2	C	C	C	0	G
G	T	A	A	A	2	C	C	C	0	N
N	N	N	N	N	N	N	N	N	N	N

AL1_28	AL1_29	AL1_30	AL1_31	AL2_1	AL2_2	AL2_3	AL2_4	AL2_5	AL2_6	AL2_7	AL2_8	AL2_9
4	7	G	1	C	G	3	A	G	3	C	T	C
4	7	G	0	C	G	0	A	G	0	C	T	C
4	7	G	0	N	N	N	N	N	N	N	N	N
0	0	A	1	C	G	0	A	G	0	C	T	C
4	7	G	0	N	N	N	N	N	N	N	N	N
N	N	N	N	T	G	0	G	G	3	T	T	G
0	0	G	1	C	G	3	G	A	3	T	C	T
N	N	N	N	C	G	0	A	G	0	C	T	C
N	N	N	N	N	N	N	N	N	N	N	N	N
0	0	A	1	C	G	0	A	G	0	C	T	C
0	0	A	1	C	G	3	G	A	3	T	C	T
N	N	N	N	C	T	0	A	G	3	T	T	G
0	0	A	1	T	G	0	G	G	3	T	T	G
4	7	G	0	N	N	N	N	N	N	N	N	N
N	N	N	N	C	G	3	G	A	3	T	C	T
0	0	A	1	C	G	3	G	A	3	T	C	T
N	N	N	N	N	N	N	N	N	N	N	N	N
N	N	N	N	C	G	0	A	G	0	C	T	C
0	0	A	1	C	G	3	G	A	3	T	C	T
N	N	N	N	N	N	N	N	N	N	N	N	N
N	N	N	N	N	N	N	N	N	N	N	N	N
4	7	G	0	N	N	N	N	N	N	N	N	N
4	7	G	0	C	G	3	G	A	3	T	C	T
N	N	N	N	N	N	N	N	N	N	N	N	N
0	0	A	1	C	G	0	G	G	3	T	T	G
0	0	A	1	C	G	0	A	G	0	C	T	C
N	N	N	N	C	G	0	A	G	0	C	T	C
4	0	G	1	C	G	3	G	A	3	T	C	T
N	N	N	N	C	G	0	A	G	0	C	T	C
4	7	G	0	C	G	3	G	A	3	T	C	T
N	N	N	N	N	G	3	G	A	3	T	C	T
N	N	N	N	C	G	3	G	A	3	T	C	T
0	0	A	1	C	G	3	G	A	3	T	C	T
0	0	G	N	N	N	N	N	N	N	N	N	N
N	N	N	N	C	G	0	A	G	0	C	T	C
0	0	A	1	N	N	N	N	N	N	N	N	N
N	N	N	N	N	G	3	G	A	3	T	C	T
N	N	N	N	N	N	N	N	N	N	N	N	N
N	N	N	N	C	G	3	G	A	3	T	C	T
N	N	N	N	N	N	N	N	N	N	N	N	N
4	7	G	0	C	G	3	G	A	3	T	C	T
4	7	G	0	C	G	3	G	A	3	T	C	T
N	N	N	N	N	N	N	N	N	N	N	N	N
0	0	A	1	C	G	0	A	G	0	C	T	C
N	N	N	N	C	G	3	G	A	3	T	C	T
N	N	N	N	N	N	N	N	N	N	N	N	N
0	0	A	1	C	G	0	A	G	0	C	T	C
0	0	A	1	C	G	0	A	G	0	C	T	C
N	N	N	N	N	N	N	N	N	N	N	N	N
N	N	N	N	N	N	N	N	N	N	N	N	N
0	0	A	1	C	G	0	A	G	0	C	T	C
N	N	N	N	C	G	3	G	A	3	T	C	T
0	0	A	1	C	G	0	A	G	0	C	T	C
4	7	G	0	C	G	0	A	G	0	C	T	C
4	7	G	0	C	T	0	A	G	3	T	T	G
N	N	N	N	C	G	3	G	A	3	T	C	T

4	7	G	0	C	G	0	A	G	0	C	T	C
N	N	N	N	C	T	0	A	G	3	T	T	G
0	0	A	1	C	G	0	A	G	0	C	T	C
N	N	N	N	T	G	0	G	G	3	T	T	G
0	0	A	1	T	G	0	G	G	3	T	T	G
4	7	G	0	N	N	N	N	N	N	N	N	N
N	N	N	N	C	G	0	A	G	0	C	T	C
0	0	A	1	C	G	0	G	G	3	T	T	G
N	N	N	N	C	G	0	G	G	3	T	T	G
N	N	N	N	N	N	N	N	N	N	N	N	N
N	N	N	N	C	G	3	G	A	3	T	C	T
0	0	A	1	N	N	N	N	N	N	N	N	N
N	N	N	N	T	G	0	G	G	3	T	T	G
N	N	N	N	C	G	0	A	G	0	C	T	C
0	0	A	1	T	G	0	G	G	3	T	T	G
0	0	A	1	N	N	N	N	N	N	N	N	N
0	0	A	1	T	G	0	G	G	3	T	T	G
N	N	N	N	N	N	N	N	N	N	N	N	N
0	0	G	1	T	G	0	G	G	3	T	T	G
0	0	A	1	N	N	N	G	G	3	T	T	G
0	0	A	1	C	T	0	A	G	3	T	T	G

N	N	N	N	T	G	0	G	G	3	T	T	G
N	N	N	N	C	G	3	G	A	3	T	C	T
N	N	N	N	T	G	0	G	G	3	T	T	G
0	0	G	1	T	G	0	G	G	3	T	T	G
N	N	N	N	C	T	0	A	G	3	T	T	G
0	0	A	1	T	G	0	G	G	3	T	T	G
N	N	N	N	T	G	0	G	G	3	T	T	G
N	N	N	N	T	G	0	G	G	3	T	T	G
N	N	N	N	T	G	0	G	G	3	T	T	G
N	N	N	N	T	G	0	G	G	3	T	T	G
0	0	G	1	N	N	N	N	N	N	N	N	N
N	N	N	N	T	G	0	G	G	3	T	T	G
N	N	N	N	T	G	0	G	G	3	T	T	G
0	0	A	1	C	T	0	A	G	3	T	T	G
0	0	A	1	C	G	3	G	A	3	T	C	T
4	7	G	0	T	G	0	G	G	3	T	T	G
0	0	A	1	T	G	0	G	G	3	T	T	G
0	0	A	1	T	G	0	G	G	3	T	T	G
N	N	N	N	C	T	0	A	G	3	T	T	G
N	N	N	N	N	N	N	N	N	N	N	N	N
0	0	A	1	T	G	0	G	G	3	T	T	G
N	N	N	N	N	N	N	N	N	N	N	N	N
0	0	G	1	T	G	0	G	G	3	T	T	G
0	0	A	1	T	G	0	G	G	3	T	T	G
N	N	N	N	T	G	0	G	G	3	T	T	G
0	0	A	1	T	G	0	G	G	3	T	T	G
0	0	G	1	C	G	0	A	G	3	T	T	G
0	0	A	1	T	G	0	G	G	3	T	T	G
0	0	A	1	N	N	N	N	N	N	N	N	N
0	0	A	1	C	G	0	G	G	3	T	T	G
0	0	A	1	N	N	N	N	N	N	N	N	N
N	N	N	N	C	G	3	G	A	3	T	C	T
0	0	A	1	N	N	N	N	N	N	N	N	N
N	N	N	N	C	G	3	A	G	3	C	T	G
0	0	A	1	C	G	0	A	G	0	C	T	C

0	0	A	1	N	N	N	N	N	N	N	N	N
0	0	A	1	N	N	N	N	N	N	N	N	N
0	0	A	1	C	G	3	G	A	3	T	C	T
4	7	G	0	N	N	N	N	N	N	N	N	N
0	0	A	1	N	N	N	N	N	N	N	N	N
0	0	A	1	C	G	3	A	G	3	C	T	G
0	0	A	1	C	G	3	G	A	3	T	C	T
N	N	N	N	N	N	N	N	N	N	N	N	N
0	0	G	1	N	N	N	N	N	N	N	N	N
0	0	A	1	N	N	N	N	N	N	N	N	N
0	0	A	1	C	G	3	G	A	3	T	C	T
4	7	G	0	C	G	3	G	A	3	T	C	T
0	0	A	1	C	G	3	G	A	3	T	C	T
N	N	N	N	N	G	3	G	A	3	T	C	T
N	N	N	N	C	G	3	G	A	3	T	C	T
N	N	N	N	C	T	0	A	G	3	T	T	G
0	0	A	1	N	N	N	N	N	N	N	N	N
0	0	A	1	N	N	3	G	A	3	T	C	T
N	N	N	N	N	N	N	N	N	N	N	N	N
4	7	G	0	C	G	3	G	A	3	T	C	T
N	N	N	N	C	G	0	G	G	3	T	T	G
N	N	N	N	C	G	3	G	A	3	T	C	T
N	N	N	N	N	N	N	N	N	N	N	N	N
4	7	G	0	N	N	N	N	N	N	N	N	N
0	0	A	1	N	N	N	N	N	N	N	N	N
4	7	G	0	N	G	0	G	G	3	T	T	G
N	N	N	N	N	N	N	N	N	N	N	N	N
N	N	N	N	C	G	3	G	A	3	T	C	T
4	7	G	0	N	N	N	N	N	N	N	N	N
N	N	N	N	N	N	N	N	N	N	N	N	N
N	N	N	N	N	N	N	N	N	N	N	N	N
4	7	G	0	N	N	N	N	N	N	N	N	N
0	0	A	1	N	N	N	N	N	N	N	N	N
4	7	G	0	T	G	0	G	G	3	T	T	G
N	N	N	N	N	N	N	N	N	N	N	N	N
4	7	G	0	T	G	0	G	G	3	T	T	G
N	N	N	N	C	G	0	A	G	0	C	T	C
N	N	N	N	N	N	N	N	N	N	N	N	N
N	N	N	N	C	G	3	G	A	3	T	C	T
0	0	A	1	T	G	0	G	G	3	T	T	G
0	0	A	1	N	N	N	N	N	N	N	N	N
N	N	N	N	C	G	0	A	G	0	C	T	C
0	0	A	1	N	G	3	G	A	3	T	C	T
4	7	G	0	C	T	0	A	G	3	T	T	G
4	7	G	0	C	T	0	A	G	3	T	T	G
0	0	A	1	C	G	0	A	G	0	C	T	C
0	0	A	1	C	G	3	G	A	3	T	C	T
4	7	G	0	N	N	N	N	N	N	N	N	N
N	N	N	N	N	N	N	N	N	N	N	N	N
0	0	G	1	N	N	N	N	N	N	N	N	N
N	N	N	N	N	N	N	N	N	N	N	N	N
N	N	N	N	N	N	N	N	N	N	N	N	N
N	N	N	N	C	G	3	G	A	3	T	C	T
0	0	A	1	C	G	3	G	A	3	T	C	T
0	0	A	1	N	G	0	A	G	0	C	T	N
0	0	A	1	N	N	N	N	N	N	N	N	N
0	0	A	1	N	N	N	N	N	N	N	N	N

N	N	N	N	N	N	N	N	N	N	N	N	N
0	0	A	1	C	G	3	G	A	3	T	C	T
N	N	N	N	C	G	0	A	G	0	C	T	C
4	7	G	0	C	G	3	G	A	3	T	C	T
4	7	G	0	N	N	N	N	N	N	N	N	N
4	7	G	0	C	G	0	A	G	0	C	T	C
0	0	A	1	N	N	N	N	N	N	N	N	N
0	0	A	1	C	G	0	G	G	3	T	T	G
4	7	G	0	C	G	3	G	A	3	T	C	T
0	0	G	1	N	N	N	N	N	N	N	N	N
4	7	G	0	C	G	0	A	G	0	C	T	C
N	N	N	N	C	G	3	G	A	3	T	C	T
N	N	N	N	N	N	N	N	N	N	N	N	N
4	7	G	0	T	G	0	G	G	3	T	T	G
0	0	A	1	N	G	3	G	A	3	T	C	T
0	0	A	1	N	N	N	N	N	N	N	N	N
0	0	A	1	C	G	3	G	A	3	T	C	T
0	0	G	1	C	G	0	A	G	0	C	T	C
N	N	N	N	N	N	N	N	N	N	N	N	N
N	N	N	N	N	G	3	G	A	3	T	C	T
4	7	G	0	N	N	N	N	N	N	N	N	N
0	0	A	1	C	G	3	A	G	3	C	T	G
N	N	N	N	N	N	N	N	N	N	N	N	N
4	7	G	0	C	G	0	A	G	0	C	T	C
N	N	N	N	T	G	0	G	G	3	T	T	G
N	N	N	N	T	G	0	G	G	3	T	T	G
0	0	A	N	T	G	0	G	G	3	T	T	G
0	0	A	1	T	G	0	G	G	3	T	T	G
N	N	N	N	N	N	N	G	G	3	T	T	G
N	N	N	N	T	G	0	G	G	3	T	T	G
4	7	G	0	C	G	0	A	G	0	C	T	C
4	7	G	0	C	G	3	G	A	3	T	C	T
4	7	G	0	C	G	3	G	A	3	T	C	T
0	0	A	1	C	G	3	G	A	3	T	C	T
0	0	A	1	C	G	3	G	A	3	T	C	T
4	7	G	0	C	G	3	G	A	3	T	C	T
N	N	N	N	N	N	N	N	N	N	N	N	N
4	7	G	0	C	G	0	A	G	0	C	T	C
4	7	G	0	C	G	0	A	G	0	C	T	C
0	0	A	1	T	G	0	G	G	3	T	T	G
0	0	A	1	T	G	0	G	G	3	T	T	G
0	0	A	1	T	G	0	G	G	3	T	T	G
N	N	N	N	C	G	0	A	G	0	C	T	C
N	N	N	N	N	G	3	G	A	3	T	C	T
N	N	N	N	T	G	0	G	G	3	T	T	G
0	0	A	1	T	G	0	G	G	3	T	T	G
N	N	N	N	C	T	0	A	G	3	T	T	G
0	0	A	1	N	G	0	G	G	3	T	T	G
0	0	G	1	T	G	0	G	G	3	T	T	G
0	0	A	1	N	N	N	N	N	N	N	N	N
0	0	G	1	C	G	3	G	A	3	T	C	T
N	N	N	N	N	N	N	N	N	N	N	N	N
0	0	A	1	N	N	N	N	N	N	N	N	N
0	0	A	1	N	N	N	N	N	N	N	N	N
0	0	A	1	N	N	N	N	N	N	N	N	N
N	N	N	N	C	G	0	A	G	0	C	T	C
N	N	N	N	C	G	3	G	A	3	T	C	G

N	N	N	N	N	N	N	N	N	N	N	N	N
N	N	N	N	N	N	N	N	N	N	N	N	N
N	N	N	N	N	N	N	N	N	N	N	N	N
N	N	N	N	N	N	N	N	N	N	N	N	N
4	7	G	0	N	N	N	N	N	N	N	N	N
0	0	A	1	C	G	0	A	G	0	C	T	C
4	7	G	0	T	G	0	G	G	3	T	T	G
N	N	N	N	N	N	N	N	N	N	N	N	N
0	0	A	1	N	N	N	N	N	N	N	N	N
4	7	G	0	N	N	N	N	N	N	N	N	N
0	0	A	1	N	N	N	N	N	N	N	N	N
N	N	N	N	C	G	0	A	G	0	C	T	C
0	0	A	1	N	N	N	N	N	N	N	N	N
0	0	G	1	N	N	N	N	N	N	N	N	N
4	7	G	0	C	G	3	G	A	3	T	C	T
N	N	N	N	C	G	0	A	G	0	C	T	C
N	N	N	N	C	G	3	G	A	3	T	C	T
0	0	A	1	C	G	3	G	A	3	T	C	T
N	N	N	N	N	N	N	N	N	N	N	N	N
4	7	G	0	T	G	0	G	G	3	T	T	G
0	0	A	1	C	G	3	G	A	3	T	C	T
0	0	A	1	N	G	3	G	A	3	T	C	T
0	0	A	1	N	N	N	N	N	N	N	N	N
N	N	N	N	N	N	N	N	N	N	N	N	N
0	0	A	1	T	G	0	G	G	3	T	T	G
0	0	A	1	N	G	3	G	A	3	T	C	T
0	0	A	1	N	N	N	N	N	N	N	N	N
0	0	A	1	T	G	0	G	G	3	T	T	G
0	0	A	1	N	G	3	G	A	3	T	C	T
N	N	N	N	N	N	N	N	N	N	N	N	N
4	7	G	0	C	T	0	A	G	3	T	T	G
0	0	A	1	N	N	N	N	N	N	N	N	N
4	7	G	0	C	G	3	G	A	3	T	C	T
4	7	G	0	C	G	3	G	A	3	T	C	T
0	0	A	1	N	N	N	N	N	N	N	N	N
N	N	N	N	C	G	3	G	A	3	T	C	T
0	0	A	1	N	N	N	N	N	N	N	N	N
0	0	A	1	N	N	N	N	N	N	N	N	N
0	0	A	1	C	G	3	G	A	3	T	C	T
0	0	A	1	C	G	3	G	A	3	T	C	T
0	0	A	1	C	G	3	G	A	3	T	C	T
N	N	N	N	N	N	N	N	N	N	N	N	N
0	0	A	1	C	G	0	A	G	0	C	T	C
0	0	A	1	N	N	N	N	N	N	N	N	N
N	N	N	N	N	N	N	N	N	N	N	N	N
0	0	G	1	N	N	N	N	N	N	N	N	N
0	0	A	1	N	N	N	N	N	N	N	N	N
N	N	N	N	T	G	0	G	G	3	T	T	G
0	0	A	1	C	G	0	G	G	3	T	T	G
N	N	N	N	N	N	N	N	N	N	N	N	N
0	0	A	1	C	G	3	G	A	3	T	C	T
0	0	G	1	N	N	N	N	N	N	N	N	N
N	N	N	N	N	N	N	N	N	N	N	N	N
0	0	A	1	C	G	0	A	G	0	C	T	C
N	N	N	N	N	N	N	N	N	N	N	N	N
N	N	N	N	N	N	N	N	N	N	N	N	N

AL2_10	AL2_11	AL2_12	AL2_13	AL2_14	AL2_15	AL2_16	AL2_17	AL3_1	AL3_2	AL3_3	AL3_4
A	6	G	C	A	G	8	C	1	1	4	N
A	6	G	C	A	G	0	C	N	N	N	N
G	0	G	C	T	A	8	G	1	1	4	C
A	6	G	C	A	G	0	C	1	1	4	G
N	N	N	N	N	N	N	N	N	N	N	N
G	6	T	T	T	G	8	C	1	1	4	G
A	0	G	C	T	A	8	G	N	N	N	N
A	6	G	C	A	G	0	C	1	0	0	G
N	N	N	N	N	N	N	N	1	1	4	G
A	6	G	C	A	G	0	C	0	0	4	G
A	0	G	C	T	A	8	G	0	1	4	G
A	6	C	C	T	G	8	G	N	N	N	N
G	6	T	T	T	G	8	C	N	N	N	N
N	N	N	N	N	N	N	N	1	1	4	G
A	0	G	C	T	A	8	G	N	N	N	N
A	0	G	C	T	A	8	G	1	1	4	G
N	N	N	N	N	N	N	N	N	N	N	N
A	6	G	C	A	G	0	C	N	N	N	N
A	0	G	C	T	A	8	G	N	N	N	N
N	N	N	N	N	N	N	N	1	1	4	C
N	N	N	N	N	N	N	N	N	N	N	N
N	N	N	N	N	N	N	N	N	N	N	N
A	0	G	C	T	A	8	G	N	N	N	N
N	N	N	N	N	N	N	N	1	1	4	G
G	6	C	C	T	G	8	C	1	1	4	G
A	6	G	C	A	G	0	C	N	N	N	N
A	6	G	C	A	G	0	C	1	1	4	G
A	0	G	C	T	A	8	G	1	1	4	G
A	6	G	C	A	G	0	C	N	N	N	N
A	0	G	C	T	A	8	G	N	N	N	N
A	0	G	C	T	A	8	N	1	0	0	G
A	0	G	C	T	A	8	G	N	N	N	N
A	0	G	C	T	A	8	G	N	N	N	N
N	N	N	N	N	N	N	N	N	N	N	N
A	6	G	C	A	G	0	C	N	N	N	N
N	N	N	N	N	N	N	N	1	1	4	G
A	0	G	C	T	A	8	N	1	1	4	G
N	N	N	N	N	N	N	N	N	N	N	N
A	0	G	C	T	A	8	G	1	1	4	G
N	N	N	N	N	N	N	N	N	N	N	N
A	0	G	C	T	A	8	G	1	1	4	G
A	0	G	C	T	A	8	G	N	N	N	N
N	N	N	N	N	N	N	N	1	0	0	G
A	6	G	C	A	G	0	C	1	1	4	C
A	0	G	C	T	A	8	G	N	N	N	N
N	N	N	N	N	N	N	N	1	0	0	G
A	6	G	C	A	G	0	C	1	1	4	G
A	6	G	C	A	G	0	C	1	1	4	G
N	N	N	N	N	N	N	N	1	1	4	G
N	0	G	C	T	A	8	G	1	1	4	G
A	6	G	C	A	G	0	C	N	N	N	N
A	0	G	C	T	A	8	G	N	N	N	N
A	6	G	C	A	G	0	C	N	N	N	N
A	6	G	C	A	G	0	C	1	1	4	G
A	6	C	C	T	G	8	G	N	N	N	N
A	0	G	C	T	A	8	G	1	1	4	G

A	6	G	C	A	G	0	C	0	0	4	G
A	6	C	C	T	G	8	G	N	N	N	N
A	6	G	C	A	G	0	C	N	N	N	N
G	6	T	T	T	G	8	C	0	1	4	G
G	6	T	T	T	G	8	C	N	N	N	N
N	N	N	N	N	N	N	N	1	1	4	G
A	6	G	C	A	G	0	C	N	N	N	N
G	6	T	T	T	G	8	C	1	1	4	G
G	6	T	T	T	G	8	C	N	N	N	N
N	N	N	N	N	N	N	N	1	1	4	G
A	0	G	C	T	A	8	G	N	N	N	N
N	N	N	N	N	N	N	N	0	0	4	G
G	6	T	T	T	G	8	C	0	1	4	C
A	6	G	C	A	G	0	C	0	0	4	G
G	6	T	T	T	G	8	C	0	0	4	G
N	N	N	N	N	N	N	N	N	N	N	N
G	6	T	T	T	N	N	N	N	N	N	N
N	N	N	N	N	N	N	N	N	N	N	N
G	6	T	T	T	G	8	C	1	1	4	G
A	6	C	C	T	G	8	C	0	0	4	G
A	6	C	C	T	G	8	G	N	N	N	N

G	6	T	T	T	G	8	C	0	0	4	G
A	0	G	C	T	A	8	G	1	1	4	G
G	6	T	T	T	G	8	C	1	1	4	G
G	6	T	T	T	G	8	C	0	0	4	G
A	6	C	C	T	G	8	G	1	1	4	G
G	6	T	T	T	G	8	C	0	0	4	G
G	6	T	T	T	G	8	C	1	1	4	G
G	6	T	T	T	G	8	C	1	1	4	G
G	6	T	T	T	G	8	C	N	N	N	N
G	6	T	T	T	G	8	C	0	1	4	C
N	N	N	N	N	N	N	N	0	0	4	G
G	6	T	T	T	G	8	C	1	1	4	G
G	6	T	T	T	G	8	C	1	1	4	G
G	6	T	T	T	G	8	C	1	1	4	G
A	0	G	C	T	A	8	G	0	0	4	G
G	6	T	T	T	G	8	C	1	1	4	G
G	6	T	T	T	G	8	C	0	0	4	G
G	6	T	T	T	G	8	C	0	0	4	G
A	6	C	C	T	G	8	G	0	1	4	G
N	0	G	C	T	A	8	N	N	N	N	N
G	6	T	T	T	G	8	C	N	N	N	N
N	N	N	N	N	N	N	N	0	0	4	G
G	6	T	T	T	G	8	C	N	N	N	N
G	6	T	T	T	G	8	C	0	1	4	C
G	6	T	T	T	G	8	C	N	N	N	N
G	6	T	T	T	G	8	C	1	1	4	G
G	6	T	T	T	G	8	C	N	N	N	N
G	6	T	T	T	G	8	C	N	N	N	N
N	N	N	N	N	N	N	N	N	N	N	N
G	6	T	T	T	G	8	C	1	1	4	G
N	N	N	N	N	N	N	N	1	1	4	G
A	0	G	C	T	A	8	G	N	N	N	N
N	N	N	N	N	N	N	N	1	0	0	G
G	6	G	C	T	G	8	C	1	0	0	G
A	6	G	C	A	G	0	C	N	N	N	N

N	N	N	N	N	N	N	N	N	N	N	N
N	N	N	N	N	N	N	N	N	N	N	N
A	0	G	C	T	A	8	G	1	1	4	C
N	N	N	N	N	N	N	N	N	N	N	N
N	N	N	N	N	N	N	N	1	0	0	G
G	6	G	C	T	G	8	C	1	1	4	G
A	0	G	C	T	A	8	G	1	1	4	G
N	N	N	N	N	N	N	N	1	1	4	G
N	N	N	N	N	N	N	N	1	0	0	G
N	N	N	N	N	N	N	N	N	N	N	N
A	0	G	C	T	A	8	G	N	N	N	N
A	0	G	C	T	A	8	G	1	1	4	G
A	0	G	C	T	A	8	G	N	N	N	N
A	0	G	C	T	A	8	G	N	N	N	N
A	0	G	C	T	A	8	G	N	N	N	N
A	6	C	C	T	G	8	G	1	0	0	G
N	0	G	C	T	A	8	G	N	N	N	N
A	0	G	C	T	A	8	G	N	N	N	N
N	N	N	N	N	N	N	N	1	1	4	G
A	0	G	C	T	A	8	G	1	1	4	G
G	0	G	C	T	A	8	G	0	0	4	G
A	0	G	C	T	A	8	G	N	N	N	N
N	N	N	N	N	N	N	N	1	1	4	G
N	N	N	N	N	N	N	N	1	0	0	G
N	N	N	N	N	N	N	N	1	1	4	G
G	6	N	N	N	N	N	N	N	N	N	N
N	N	N	N	N	N	N	N	1	1	4	G
A	0	G	C	T	A	8	G	N	N	N	N
G	0	G	C	T	A	8	G	1	0	0	G
N	N	N	N	N	N	N	N	1	1	4	G
N	0	G	C	T	A	8	G	1	0	0	G
N	N	N	N	N	N	N	N	1	0	0	G
N	N	N	N	N	N	N	N	1	1	4	C
G	6	T	T	T	G	8	C	N	N	N	N
N	N	N	N	N	N	N	N	0	1	4	G
G	6	T	T	T	G	8	C	1	1	4	G
A	0	G	C	T	A	8	G	0	0	4	G
N	N	N	N	N	N	N	N	0	0	4	G
A	0	G	C	T	A	8	G	1	1	4	G
G	6	T	T	T	G	8	C	N	N	N	N
N	N	N	N	N	N	N	N	N	N	N	N
A	6	G	C	A	G	0	C	N	N	N	N
A	0	G	C	T	A	8	G	1	1	4	G
A	6	C	C	T	G	8	G	N	N	N	N
A	6	C	C	T	G	8	G	1	1	4	G
A	6	G	C	A	G	0	C	1	1	4	G
A	0	G	C	T	A	8	G	1	0	0	G
N	N	N	N	N	N	N	N	N	N	N	N
N	N	N	N	N	N	N	N	0	1	4	G
N	N	N	N	N	N	N	N	0	0	4	G
N	N	N	N	N	N	N	N	1	1	4	G
N	N	N	N	N	N	N	N	1	1	4	G
A	0	G	C	T	A	8	G	1	1	4	G
A	0	G	C	T	A	8	G	N	N	N	N
A	6	G	C	A	G	0	C	N	N	N	N
G	0	G	C	T	A	8	G	1	1	4	C
N	N	N	N	N	N	N	N	1	1	4	G

N	0	G	C	T	A	8	G	N	N	N	N
A	0	G	C	T	A	8	G	N	N	N	N
A	6	G	C	A	G	0	C	N	N	N	N
A	0	G	C	T	A	8	G	1	1	4	G
N	0	G	C	T	A	8	G	1	0	N	G
A	6	G	C	A	G	0	C	1	0	0	G
N	N	N	N	N	N	N	N	1	0	0	G
G	6	T	T	T	G	8	C	0	1	4	G
A	0	G	C	T	A	8	G	1	1	4	G
N	N	N	N	N	N	N	N	1	1	4	G
A	6	G	C	A	G	0	C	0	0	4	G
A	0	G	C	T	A	8	G	N	N	N	N
N	N	N	N	N	N	N	N	N	N	N	N
G	6	T	T	T	G	8	C	1	1	4	G
A	0	G	C	T	A	8	G	1	1	4	G
N	N	N	N	N	N	N	N	N	N	N	N
A	0	G	C	T	A	8	G	1	1	4	G
A	6	G	C	A	G	0	C	N	N	N	N
N	N	N	N	N	N	N	N	1	1	4	G
A	0	G	C	T	A	8	G	N	N	N	N
N	N	N	N	N	N	N	N	1	1	4	G
G	6	G	C	T	G	8	C	1	1	4	C
N	N	N	N	N	N	N	N	N	N	N	N
A	6	G	C	A	G	0	C	N	N	N	N
G	6	T	T	T	G	8	C	1	1	4	G
G	6	T	T	T	G	8	C	1	1	4	G
G	6	T	T	T	G	8	G	1	1	4	G
G	6	T	T	T	G	8	C	N	N	N	N
G	6	T	T	T	G	8	C	N	N	N	N
G	6	T	T	T	G	8	C	N	N	N	N
A	6	G	C	A	G	0	C	1	1	4	G
A	0	G	C	T	A	8	G	1	1	4	G
A	0	G	C	T	A	8	G	0	0	4	G
A	0	G	C	T	A	8	G	1	1	4	C
A	0	G	C	T	A	8	G	1	1	4	C
A	0	G	C	T	A	8	G	0	0	4	G
N	N	N	N	N	N	N	N	N	N	N	N
A	6	G	C	A	G	0	C	N	N	N	N
A	6	G	C	A	G	0	C	1	1	4	G
G	6	T	T	T	G	8	C	N	N	N	N
G	6	T	T	T	G	8	C	N	N	N	N
G	6	T	T	T	G	8	C	1	0	4	G
A	6	G	C	A	G	0	C	N	N	N	N
A	0	G	C	T	A	8	G	N	N	N	N
G	6	T	T	T	G	8	C	1	1	4	G
G	6	T	T	T	G	8	C	N	N	N	N
A	6	C	C	T	G	8	G	1	1	4	G
G	6	T	T	T	G	8	C	N	N	N	N
G	6	T	T	T	G	8	C	0	0	4	G
N	N	N	N	N	N	N	N	1	1	4	G
A	0	G	C	T	A	8	G	1	1	4	G
N	N	N	N	N	N	N	N	1	1	4	G
N	N	N	N	N	N	N	N	N	N	N	N
N	N	N	N	N	N	N	N	1	1	4	G
N	N	N	N	N	N	N	N	1	1	4	G
A	6	G	C	A	G	0	C	0	0	4	G
A	0	G	C	T	A	8	G	0	0	4	G

N	N	N	N	N	N	N	N	1	1	4	G
N	N	N	N	N	N	N	N	1	0	0	G
N	N	N	N	N	N	N	N	1	0	0	G
N	N	N	N	N	N	N	N	1	1	4	C
N	N	N	N	N	N	N	N	1	1	4	G
A	6	G	C	A	G	0	C	1	1	4	G
G	6	T	T	T	G	8	C	1	0	0	G
N	N	N	N	N	N	N	N	1	0	0	G
N	N	N	N	N	N	N	N	N	N	N	N
N	N	N	N	N	N	N	N	1	1	4	G
N	N	N	N	N	N	N	N	1	1	4	C
A	6	G	C	A	G	0	C	N	N	N	N
N	N	N	N	N	N	N	N	N	N	N	N
N	0	G	C	T	A	8	G	N	N	N	N
A	0	G	C	T	A	8	G	1	1	4	G
A	6	G	C	A	G	0	C	1	1	4	G
A	0	G	C	T	A	8	G	N	N	N	N
A	0	G	C	T	A	8	G	N	N	N	N
N	0	G	C	T	A	8	N	1	1	4	G
G	6	T	T	T	G	8	C	1	0	0	G
A	0	G	C	T	A	8	G	N	N	N	N
A	0	G	C	T	A	8	N	N	N	N	N
N	N	N	N	N	N	N	N	1	1	4	G
N	N	N	N	N	N	N	N	1	1	4	G
G	6	T	T	T	G	8	C	1	1	4	G
A	0	G	C	T	A	8	N	N	N	N	N
N	N	N	N	N	N	N	N	1	0	0	G
G	6	T	T	T	G	8	C	1	1	4	G
A	0	G	C	T	A	8	G	1	1	4	G
N	N	N	N	N	N	N	N	N	N	N	N
G	6	T	T	T	G	8	C	0	0	4	G
N	N	N	N	N	N	N	N	1	1	4	G
A	0	G	C	T	A	8	G	N	N	N	N
A	0	G	C	T	A	8	G	1	1	4	G
N	N	N	N	N	N	N	N	0	1	4	C
A	0	G	C	T	A	8	G	N	N	N	N
N	N	N	N	N	N	N	N	1	0	0	G
N	N	N	N	N	N	N	N	N	N	N	N
A	0	G	C	T	A	8	G	1	0	0	G
A	0	G	C	T	A	8	G	1	0	0	G
A	0	G	C	T	A	8	G	N	N	N	N
N	N	N	N	N	N	N	N	N	N	N	N
A	6	G	C	A	G	0	C	N	N	N	N
N	N	N	N	N	N	N	N	1	0	0	G
N	N	N	N	N	N	N	N	1	1	4	G
N	N	N	N	N	N	N	N	1	1	4	C
N	N	N	N	N	N	N	N	N	N	N	N
G	6	T	T	T	G	8	C	1	0	0	G
G	6	T	T	T	G	8	C	N	N	N	N
N	N	N	N	N	N	N	N	0	0	4	G
A	0	G	C	T	A	8	G	N	N	N	N
N	N	N	N	N	N	N	N	N	N	N	N
N	N	N	N	N	N	N	N	N	N	N	N
A	6	G	C	A	G	0	C	0	0	4	G
N	N	N	N	N	N	N	N	N	N	N	N
N	N	N	N	N	N	N	N	N	N	N	N

AL3_5	AL3_6	AL3_7	AL3_8	AL3_9	AL5_1	AL5_2	AL5_3	AL5_4	AL5_5	AL5_6	AL5_7	AL5_8
N	C	C	T	1	T	A	G	8	T	A	A	T
N	C	C	T	1	T	A	G	8	T	A	A	T
A	C	C	T	1	C	G	G	8	T	G	A	A
C	C	C	T	1	T	A	G	8	T	A	A	T
N	N	N	N	N	N	N	N	N	N	N	N	N
C	C	C	T	1	N	N	N	N	N	N	N	N
N	N	N	N	N	N	N	N	N	N	N	N	N
C	C	T	T	1	T	A	G	8	T	A	A	T
C	C	C	T	1	C	G	G	8	T	G	A	A
A	T	C	A	2	T	A	G	8	T	A	A	T
C	C	C	T	0	C	G	T	8	G	G	G	A
N	N	N	N	N	C	G	G	8	T	A	A	A
N	N	N	N	N	C	G	G	8	T	A	A	A
C	C	C	T	1	C	G	G	0	G	A	A	A
N	N	N	N	N	C	G	T	8	G	G	G	A
C	C	C	T	1	N	N	N	N	N	N	N	N
N	N	N	N	N	T	A	G	8	T	A	A	T
N	N	N	N	N	T	A	G	8	T	A	A	T
N	N	N	N	N	C	G	G	8	T	A	A	A
A	C	C	T	1	C	G	G	0	G	A	A	A
C	C	C	T	1	N	N	N	N	N	N	N	N
N	N	N	N	N	N	N	N	N	N	N	N	N
N	C	C	T	1	C	G	T	8	G	G	G	A
C	C	C	T	1	C	G	G	0	G	A	A	A
C	C	C	T	1	C	G	T	8	G	G	G	A
N	N	N	N	N	N	N	N	0	G	A	A	A
C	C	C	T	1	T	A	G	8	T	A	A	T
C	C	C	T	1	C	G	T	8	G	G	G	A
N	N	N	N	N	T	A	G	8	T	A	A	T
N	N	N	N	N	N	N	N	N	N	N	N	N
C	C	T	T	1	C	G	G	8	T	A	A	A
N	N	N	N	N	C	G	T	8	G	G	G	A
N	N	N	N	N	C	G	T	8	G	G	G	A
N	N	N	N	N	N	N	N	N	N	N	N	N
N	N	N	N	N	N	N	N	N	N	N	N	N
C	C	C	T	1	N	N	N	N	N	N	N	N
C	N	N	N	N	C	G	G	8	T	G	A	A
N	T	C	A	2	N	N	N	N	N	N	N	N
C	C	C	T	1	N	N	N	N	N	N	N	N
N	N	N	N	N	N	G	T	8	G	G	G	A
C	C	C	T	1	C	G	T	8	G	G	G	A
N	C	C	T	1	N	N	N	N	N	N	N	N
C	C	T	T	1	N	N	N	0	G	A	A	A
A	C	C	T	1	C	G	G	0	G	A	A	A
N	N	N	N	N	C	G	T	8	G	G	G	A
C	C	T	T	1	C	G	G	0	G	A	A	A
C	C	C	T	1	T	A	G	8	T	A	A	T
C	C	C	T	1	T	A	G	8	T	A	A	T
C	C	C	T	1	N	N	N	N	N	N	N	N
C	C	C	T	1	N	N	N	N	N	N	N	N
N	N	N	N	N	N	N	N	N	N	N	N	N
N	C	C	T	1	C	G	T	8	G	G	G	A
N	C	C	T	1	T	A	G	8	T	A	A	T
C	C	C	T	1	T	A	G	8	T	A	A	T
N	N	N	N	N	C	G	T	8	G	G	A	A
C	C	C	T	1	N	N	N	N	N	N	N	N

A	C	C	T	1	T	A	G	8	T	A	A	T
N	N	N	N	N	N	N	T	8	G	G	A	A
N	N	N	N	N	T	A	G	8	T	A	A	T
C	N	N	N	N	N	N	N	8	T	A	A	A
N	N	N	N	N	C	G	G	0	G	A	A	A
C	C	C	T	1	N	N	N	N	N	N	N	N
N	C	C	T	1	T	A	G	8	T	A	A	T
C	C	C	T	1	C	G	T	8	G	G	A	A
N	N	N	N	N	N	N	N	N	N	N	N	N
C	C	C	T	1	N	N	N	0	G	A	A	A
N	N	N	N	N	C	G	G	8	T	A	A	A
A	T	C	A	2	C	G	T	8	G	G	A	A
A	C	C	A	2	C	G	G	0	G	A	A	A
A	T	C	A	2	N	A	G	8	T	A	A	T
A	T	C	A	2	N	N	N	N	N	N	N	N
N	C	C	A	2	N	N	N	8	T	N	N	A
N	N	N	N	N	N	N	G	8	T	A	A	A
N	N	N	N	N	N	N	N	N	N	N	N	N
C	C	C	T	1	N	N	N	N	N	N	N	N
A	T	C	A	2	N	N	T	8	G	G	G	A
N	T	C	A	2	C	G	T	8	G	G	A	A

A	T	C	A	2	C	G	G	8	G	G	G	A
C	C	C	T	1	C	G	T	8	G	G	G	A
C	C	C	T	1	C	G	G	0	G	A	A	A
A	T	C	A	2	N	N	N	N	N	N	N	N
C	C	C	T	1	C	G	T	8	G	G	A	A
A	T	C	A	2	C	G	T	8	G	G	G	A
C	C	C	T	1	N	N	N	N	N	N	N	N
C	C	C	T	1	C	G	G	8	T	G	A	A
N	N	N	N	N	N	N	N	N	N	N	N	N
A	C	C	A	2	C	G	G	8	T	A	A	A
A	T	C	A	2	N	N	N	N	N	N	N	N
C	C	C	T	1	C	G	G	8	T	A	A	A
C	C	C	A	2	C	G	T	8	G	G	G	A
C	C	C	T	1	C	G	G	0	G	A	A	A
A	T	C	A	2	C	G	T	8	G	G	G	A
C	C	C	T	1	C	G	G	8	T	A	A	A
A	T	C	A	2	N	N	N	0	G	A	A	A
A	T	C	A	2	C	G	G	0	G	A	A	A
G	C	C	T	0	C	G	G	0	G	A	A	A
N	N	N	N	N	N	N	N	N	N	N	N	N
N	C	C	T	1	C	G	T	8	G	G	G	A
A	T	C	A	2	C	G	G	8	T	G	A	A
N	N	N	N	N	N	N	N	N	N	N	N	N
A	C	C	A	2	C	G	T	8	G	G	G	A
N	N	N	N	N	C	G	T	8	G	G	G	A
C	C	C	T	1	C	G	T	8	G	G	G	A
N	N	N	N	N	N	N	N	N	N	N	N	A
N	C	C	A	2	N	N	N	N	N	N	N	N
N	N	N	N	N	C	G	G	0	G	A	A	A
C	C	C	T	1	N	N	N	N	N	N	N	N
C	C	C	T	1	C	G	G	0	G	A	A	A
N	N	N	N	N	C	G	T	8	G	G	G	A
C	C	T	T	1	N	N	N	N	N	N	N	N
C	C	T	T	1	C	G	G	8	T	G	A	A
N	N	N	N	N	N	N	G	8	T	A	A	T

N	N	N	N	N	N	N	N	N	N	N	N	N
N	N	N	N	N	N	N	N	N	N	N	N	N
A	C	C	T	1	N	N	N	N	N	N	N	N
N	N	N	N	N	N	N	G	0	G	A	A	A
C	C	T	T	1	N	N	N	N	N	N	N	N
C	C	C	T	1	C	G	G	8	T	G	A	A
C	C	C	T	1	C	G	T	8	G	G	G	A
C	C	C	T	1	C	G	G	0	G	A	A	A
C	C	T	T	1	N	N	N	N	N	N	N	N
N	N	N	N	N	N	N	N	N	N	N	N	N
N	N	N	N	N	N	N	N	N	N	N	N	N
C	C	C	T	1	C	G	T	8	G	G	G	A
N	N	N	N	N	N	N	N	N	N	N	N	N
N	N	N	N	N	C	G	T	8	G	G	G	A
N	N	N	N	N	C	G	T	8	G	G	G	A
C	C	T	T	1	N	N	N	N	N	N	N	N
N	N	N	N	N	C	G	G	0	G	A	A	A
N	N	N	N	N	N	N	N	N	N	N	N	N
C	C	C	T	1	C	G	G	0	G	A	A	A
C	C	C	T	1	N	N	T	8	G	G	G	A
A	T	C	A	2	N	N	N	N	N	N	N	N
N	N	N	N	N	C	G	G	8	T	A	A	A
C	C	C	T	1	N	N	N	N	N	N	N	N
C	C	T	T	1	C	G	G	0	G	A	A	A
C	C	C	T	1	C	G	G	0	G	A	A	A
N	C	T	T	1	C	G	G	0	G	A	A	A
C	C	C	T	1	N	N	N	0	G	A	A	A
N	N	N	N	N	N	N	N	N	N	N	N	N
C	C	T	T	1	C	G	G	8	T	G	A	A
C	C	C	T	1	C	G	G	0	G	A	A	A
C	C	T	T	1	N	N	G	8	G	G	A	A
C	C	T	T	1	C	G	G	0	G	A	A	A
A	C	C	T	1	C	G	G	0	G	A	A	A
N	C	C	T	1	C	G	G	0	G	A	A	A
C	C	C	T	1	N	N	N	N	N	N	N	N
C	C	C	T	1	C	G	G	8	G	G	G	A
A	T	C	A	2	N	N	N	8	G	G	A	A
A	T	C	A	2	N	N	N	N	N	N	N	N
C	C	C	T	1	N	N	N	N	N	N	N	N
N	N	N	N	N	C	G	T	8	G	G	G	A
N	N	N	N	N	T	A	G	8	T	A	A	T
N	N	N	N	N	N	N	G	8	T	A	A	A
C	C	C	T	1	C	G	G	0	G	A	A	A
N	N	N	N	N	C	G	T	8	G	G	A	A
C	C	C	T	1	N	N	N	8	T	A	A	T
C	C	C	T	1	T	A	G	8	T	A	A	T
C	C	T	T	1	C	G	G	8	T	A	A	A
N	N	N	N	N	C	G	G	0	G	A	A	A
C	C	C	T	1	N	N	-	N	-	A	A	A
A	T	C	A	2	C	G	G	0	G	A	A	A
C	C	C	T	1	N	N	N	N	N	N	N	N
C	C	C	T	1	C	G	G	0	G	A	A	A
C	C	C	T	1	C	G	G	8	T	A	A	A
N	N	N	N	N	C	G	G	8	T	A	A	A
N	N	N	N	N	T	A	G	8	T	A	A	T
A	C	C	T	1	C	G	G	8	T	G	A	A
C	C	C	T	1	C	G	G	8	T	A	A	A

N	N	N	N	N	C	G	G	8	T	G	A	A
N	N	N	N	N	N	N	T	8	G	G	G	A
N	N	N	N	N	N	N	G	8	T	A	A	T
C	C	C	T	1	C	G	G	8	T	A	A	A
C	C	T	T	1	C	G	G	8	T	G	A	A
C	C	T	T	1	T	A	G	8	T	A	A	T
C	C	T	T	1	C	G	G	0	G	A	A	A
N	C	C	T	0	C	G	T	8	G	G	A	A
C	C	C	T	1	N	N	N	N	N	N	N	N
C	C	C	T	1	N	N	N	N	N	N	N	N
A	T	C	T	1	T	A	G	8	T	A	A	T
N	N	N	N	N	N	N	N	N	N	N	N	N
N	N	N	N	N	C	G	G	8	G	G	A	A
C	C	C	T	1	C	G	G	0	G	A	A	A
C	C	C	T	1	N	N	N	8	G	A	A	A
N	N	N	N	N	C	G	G	8	T	G	A	A
C	C	C	T	1	N	N	N	N	N	N	N	N
N	N	N	N	N	N	N	N	N	N	N	N	N
C	C	C	T	1	N	N	N	N	N	N	N	N
N	N	N	N	N	N	N	N	N	N	N	N	N
C	C	C	T	1	N	N	N	N	N	N	N	N
A	C	C	T	1	C	G	G	8	T	G	A	A
N	N	N	N	N	N	N	N	N	N	N	N	N
N	N	N	N	N	N	N	G	0	G	A	A	A
C	C	C	T	1	N	N	N	N	N	N	N	N
C	C	C	T	1	N	N	N	N	N	N	N	N
C	C	C	T	1	C	G	T	8	G	G	G	A
N	N	N	N	N	C	G	G	0	G	A	A	A
N	C	C	T	1	N	N	T	8	G	G	G	A
N	T	C	A	2	C	G	T	8	G	G	G	A
A	T	C	A	2	N	N	N	N	N	N	N	N
C	C	C	T	1	N	N	N	N	N	N	N	N
A	T	C	A	2	N	N	N	N	N	N	G	A
A	C	C	T	1	C	G	T	8	G	G	G	A
A	C	C	T	1	C	G	T	8	G	G	G	A
A	T	C	A	2	N	N	N	N	N	N	N	N
N	N	N	N	N	N	N	N	8	G	G	G	A
N	N	N	N	N	N	N	N	N	N	N	N	N
C	C	C	T	1	T	A	G	8	T	A	A	T
N	N	N	N	N	C	G	T	8	G	G	G	A
N	T	C	A	2	C	G	T	8	G	G	G	A
A	T	C	A	2	C	G	G	0	G	A	A	A
N	N	N	N	N	T	A	G	8	T	A	A	T
N	N	N	N	N	N	N	N	N	N	N	N	N
C	C	C	T	1	N	N	T	8	G	G	G	A
N	N	N	N	N	N	N	N	N	N	N	N	N
C	C	C	T	1	C	G	G	0	G	A	A	A
N	N	N	N	N	C	G	T	8	G	G	G	A
A	T	C	A	2	N	N	N	N	N	N	N	N
C	C	C	T	1	C	G	G	8	T	A	A	A
C	C	C	T	1	C	G	G	8	G	G	A	A
C	C	C	T	1	C	G	G	0	G	A	A	A
N	N	N	N	N	N	N	N	N	N	N	N	N
C	C	C	T	1	C	G	G	0	G	A	A	A
C	C	C	T	1	N	N	N	0	G	A	A	A
A	T	C	A	2	C	G	G	0	G	A	A	A
A	T	C	A	2	N	N	N	N	N	N	N	N

C	C	C	T	1	C	G	G	0	G	A	A	A
C	C	T	T	1	C	G	G	0	G	A	A	A
C	C	N	T	1	C	G	G	0	G	A	A	A
A	C	C	T	1	C	G	G	0	G	A	A	A
C	C	C	T	1	C	G	G	0	G	A	A	A
C	C	C	T	1	N	N	G	8	T	A	A	T
C	C	T	T	1	C	G	G	0	G	A	A	A
C	C	T	T	1	N	N	-	N	-	-	-	A
N	C	C	T	1	C	G	G	0	G	A	A	A
C	C	C	T	1	N	N	N	0	G	A	A	A
A	C	C	T	1	C	G	G	8	T	G	A	A
N	N	N	N	N	T	A	G	8	T	A	A	T
N	N	N	N	N	N	N	N	N	N	N	N	N
N	N	N	N	N	C	G	G	8	T	G	A	A
C	C	C	T	1	C	G	T	8	G	G	G	A
C	C	C	T	1	T	A	G	8	T	A	A	T
N	N	N	N	N	N	N	G	0	G	A	A	A
N	C	C	T	1	C	G	T	8	G	G	G	A
C	C	C	T	1	N	N	N	N	N	N	N	N
C	C	T	T	1	N	N	N	N	N	N	N	N
N	N	N	N	N	N	N	N	N	N	N	N	N
N	N	N	N	N	C	G	G	0	G	A	A	A
C	C	C	T	1	N	N	G	0	G	A	A	A
C	C	C	T	1	C	G	G	0	G	A	A	A
C	C	C	T	1	N	N	G	0	G	A	A	A
N	N	N	N	N	N	N	N	N	N	N	N	N
C	C	T	T	1	T	A	G	8	T	A	A	T
C	C	C	T	1	N	N	N	N	N	N	N	N
C	C	C	T	1	N	N	G	8	T	A	A	A
N	N	N	N	N	C	G	G	8	T	A	A	A
A	T	C	A	2	N	N	N	N	N	N	N	N
C	C	C	T	1	N	N	N	N	N	N	N	N
N	N	N	N	N	C	G	G	8	T	A	A	A
C	C	C	T	1	C	G	G	0	G	A	A	A
A	C	C	A	2	N	G	T	8	G	G	A	A
N	N	N	N	N	C	G	T	8	G	G	G	A
C	C	T	T	1	N	N	G	8	T	A	A	A
N	N	N	N	N	T	A	G	8	T	A	A	T
C	C	T	T	1	C	G	G	8	T	A	A	A
C	C	T	T	1	C	G	G	8	T	A	A	A
N	N	N	N	N	C	G	G	8	T	A	A	A
N	N	N	N	N	N	N	N	N	N	N	N	N
N	N	N	N	N	T	A	G	8	T	A	A	T
C	C	T	T	1	C	G	G	0	G	A	A	A
C	C	C	T	1	N	N	N	N	N	N	N	N
A	C	C	T	1	C	G	G	8	T	G	A	A
N	N	N	N	N	N	N	N	N	N	N	N	N
C	C	T	T	1	N	N	N	N	N	N	N	N
N	N	N	N	N	N	N	N	N	N	N	N	N
A	T	C	A	2	N	N	N	N	N	N	N	N
N	N	N	N	N	C	G	G	8	T	A	A	A
N	N	N	N	N	N	N	N	N	N	N	N	N
N	N	N	N	N	T	A	G	8	T	A	A	T
A	T	C	A	2	T	A	G	8	T	A	A	T
N	N	N	N	N	C	G	G	8	T	A	A	A
N	N	N	N	N	N	N	N	N	N	N	N	N

AL5_9	AL5_10	AL5_11	AL5_12	AL5_13	AL5_14	AL5_15	AL5_16	AL5_17	AL5_18	AL5_19	AL5_20
7	6	A	C	2	G	3	T	T	3	7	1
0	6	A	C	2	G	3	T	T	0	7	0
0	6	G	T	5	G	6	T	T	3	2	1
0	6	A	C	2	G	3	T	T	0	7	0
N	N	N	N	N	N	N	N	N	N	N	N
N	N	N	N	N	N	N	N	N	N	N	N
N	N	N	N	N	N	N	N	N	N	N	N
0	6	A	C	2	G	3	T	T	0	7	0
0	6	G	T	5	G	6	T	T	3	2	1
0	6	A	C	2	G	0	T	T	0	7	0
7	0	G	T	4	C	0	T	T	3	2	1
0	6	G	T	5	C	0	C	T	0	0	1
0	6	G	T	5	C	0	C	T	0	0	1
0	6	G	T	5	C	6	T	G	3	2	1
7	0	G	T	4	C	0	T	T	3	2	1
N	N	N	N	N	N	N	N	N	N	N	N
0	6	A	C	2	G	3	T	T	0	7	0
0	6	A	C	2	G	3	T	T	0	7	0
0	6	G	T	5	C	0	C	T	0	0	1
0	6	G	T	5	C	6	T	G	3	2	1
N	N	N	N	N	N	N	N	N	N	N	N
N	N	N	N	N	N	N	N	N	N	N	N
7	0	G	T	4	C	0	T	T	3	2	1
0	6	G	T	5	C	6	T	G	3	2	1
7	0	G	T	4	C	0	T	T	3	2	1
0	6	G	T	5	C	6	T	G	3	2	1
0	6	A	C	2	G	3	T	T	0	7	0
7	0	G	T	4	C	0	T	T	3	2	1
0	6	A	C	2	G	3	T	T	0	7	0
N	N	N	N	N	N	N	N	N	N	N	N
0	6	G	T	5	C	0	C	T	0	0	1
7	0	G	T	4	C	0	T	T	3	2	1
7	0	G	T	4	C	0	T	T	3	2	1
N	N	N	N	N	N	N	N	N	N	N	N
N	N	N	N	N	N	N	N	N	N	N	N
N	N	N	N	N	N	N	N	N	N	N	N
0	6	G	T	5	G	6	T	T	3	2	1
N	N	N	N	N	N	N	N	N	N	N	N
N	N	N	N	N	N	N	N	N	N	N	N
7	0	G	T	4	C	0	T	T	3	2	1
7	0	G	T	4	C	0	T	T	3	2	1
N	N	N	N	N	N	N	N	N	N	N	N
0	6	G	T	5	C	6	T	G	3	2	1
0	6	G	T	5	C	6	T	G	3	2	1
7	0	G	T	4	C	0	T	T	3	2	1
0	6	G	T	5	C	6	T	G	3	2	1
0	6	A	C	2	G	3	T	T	0	7	0
0	6	A	C	2	G	3	T	T	0	7	0
N	N	N	N	N	N	N	N	N	N	N	N
N	N	N	N	N	N	N	N	N	N	N	N
N	N	N	N	N	N	N	N	N	N	N	N
7	0	G	T	N	C	0	T	T	3	2	1
0	6	A	C	2	G	3	T	T	0	7	0
0	6	A	C	2	G	3	T	T	0	7	0
7	0	G	T	4	C	0	T	T	0	7	1
N	N	N	N	N	N	N	N	N	N	N	N

0	6	A	C	2	G	3	T	T	0	7	0
7	0	G	T	4	C	0	T	T	0	7	1
0	6	A	C	2	G	3	T	T	0	7	0
0	6	G	T	5	C	0	C	T	0	0	1
0	6	G	T	5	C	6	T	G	3	2	1
N	N	G	T	4	C	0	T	T	0	7	1
0	6	A	C	2	G	3	T	T	0	7	0
7	0	G	T	4	C	0	T	T	3	2	1
N	N	N	N	N	N	N	N	N	N	N	N
0	6	G	T	5	C	6	T	G	3	2	1
0	6	G	T	5	C	0	C	T	0	0	1
7	0	G	T	4	C	0	T	T	0	7	1
0	6	G	T	5	C	6	T	G	3	2	1
0	6	A	C	2	G	3	T	T	0	7	0
N	N	N	N	N	N	N	N	N	N	N	N
0	6	G	T	5	C	0	C	T	0	0	1
0	6	G	T	5	C	0	C	T	0	0	1
N	N	N	N	N	N	N	N	N	N	N	N
N	N	N	N	N	N	N	N	N	N	N	N
7	0	G	T	4	C	0	T	T	3	2	1
7	0	G	T	4	C	0	T	T	0	7	1

7	0	G	T	4	C	0	T	T	3	2	1
7	0	G	T	4	C	0	T	T	3	2	1
0	6	G	T	5	C	6	T	G	3	2	1
N	N	N	N	N	N	N	N	N	N	N	N
7	0	G	T	4	C	0	T	T	0	7	1
7	0	G	T	4	C	0	T	T	3	2	1
N	N	N	N	N	N	N	N	N	N	N	N
0	0	G	T	5	C	3	T	T	3	2	1
N	N	N	N	N	N	N	N	N	N	N	N
0	6	G	T	5	C	0	C	T	0	0	1
N	N	N	N	N	N	N	N	N	N	N	N
0	6	G	T	5	C	0	C	T	0	0	1
7	0	G	T	4	C	0	T	T	3	2	1
0	6	G	T	5	C	6	T	G	3	2	1
7	0	G	T	4	C	0	T	T	3	2	1
0	6	G	T	5	C	0	C	T	0	0	1
0	6	G	T	5	C	6	T	G	3	2	1
0	6	G	T	5	C	6	T	G	3	2	1
0	6	G	T	5	C	6	T	G	3	2	1
N	N	N	N	N	N	0	C	T	0	0	1
7	0	G	T	4	C	0	T	T	3	2	1
0	0	G	T	5	C	3	T	T	3	2	1
N	N	N	N	N	N	N	N	N	N	N	N
7	0	G	T	4	C	0	T	T	3	2	1
7	0	G	T	4	C	0	T	T	3	2	1
7	0	G	T	4	C	0	T	T	3	2	1
0	6	G	T	5	G	6	T	T	3	2	1
N	N	N	N	N	N	N	N	N	N	N	N
0	6	G	T	5	C	6	T	G	3	2	1
N	N	N	N	N	N	N	N	N	N	N	N
0	6	G	T	5	C	6	T	G	3	2	1
7	0	G	T	4	C	0	T	T	3	2	1
N	N	N	N	N	N	3	T	T	0	7	0
0	6	G	T	5	G	6	T	T	3	2	1
0	6	A	C	2	G	3	T	T	0	7	0

N	N	N	N	N	N	N	N	N	N	N	N
N	N	N	N	N	N	N	N	N	N	N	N
N	N	N	N	N	N	N	N	N	N	N	N
0	6	G	T	5	C	6	T	G	3	2	1
N	N	N	N	N	N	3	T	T	0	7	0
0	6	G	T	5	G	6	T	T	3	2	1
7	0	G	T	4	C	0	T	T	3	2	1
0	6	G	T	5	C	6	T	G	3	2	1
N	N	N	N	N	N	3	T	T	0	7	0
N	N	N	N	N	N	N	N	N	N	N	N
N	N	N	N	N	N	N	N	N	N	N	N
7	0	G	T	4	C	0	T	T	3	2	1
N	N	N	N	N	N	N	N	N	N	N	N
7	0	G	T	4	C	0	T	T	3	2	1
7	0	G	T	4	C	0	T	T	3	2	1
N	N	N	N	N	N	N	N	N	N	N	N
0	6	G	T	5	C	6	T	G	3	2	1
N	N	N	N	N	N	N	N	N	N	N	N
0	6	G	T	5	C	6	T	G	3	2	1
7	0	G	T	4	C	0	T	T	3	2	1
N	N	G	T	4	C	0	T	T	3	2	1
0	6	G	T	5	C	0	C	T	0	0	1
N	N	N	N	N	N	N	N	N	N	N	N
0	6	G	T	5	C	6	T	G	3	2	1
0	6	G	T	5	C	6	T	G	3	2	1
0	6	G	T	5	C	6	T	G	3	2	1
0	6	G	T	5	C	6	T	G	3	2	1
N	N	N	N	N	N	N	N	N	N	N	N
0	6	G	T	5	G	6	T	T	3	2	1
0	6	G	T	5	C	6	T	G	3	2	1
7	0	G	T	0	C	0	T	T	0	7	1
0	6	G	T	5	C	6	T	G	3	2	1
0	6	G	T	5	C	6	T	G	3	2	1
0	6	G	T	5	C	6	T	G	3	2	1
N	N	N	N	N	N	N	N	N	N	N	N
7	0	G	T	4	C	0	T	T	3	2	1
7	0	G	T	4	C	0	T	T	3	2	1
N	N	N	N	N	N	N	N	N	N	N	N
N	N	G	T	5	C	0	C	T	0	0	1
7	0	G	T	4	C	0	T	T	3	2	1
0	6	A	C	2	G	3	T	T	0	7	0
0	6	G	T	5	C	0	C	T	0	0	1
0	6	G	T	5	C	6	T	G	3	2	1
7	0	G	T	4	C	0	T	T	3	2	1
0	6	A	C	2	G	3	T	T	0	7	0
0	6	A	C	2	G	3	T	T	0	7	0
0	6	G	T	5	C	0	C	T	0	0	1
0	6	G	T	5	C	6	T	G	3	2	1
0	6	G	T	5	C	6	T	G	3	2	1
0	6	G	T	5	C	6	T	G	3	2	1
N	N	N	N	N	N	N	N	N	N	N	N
0	6	G	T	5	C	6	T	G	3	2	1
0	6	G	T	5	C	0	C	T	0	0	1
0	6	G	T	5	C	0	C	T	0	0	1
0	6	A	C	2	G	3	T	T	0	7	0
0	6	G	T	5	G	6	T	T	3	2	1
0	6	G	T	5	C	0	C	T	0	0	1

0	6	G	T	5	G	6	T	T	3	2	1
7	0	G	T	4	C	0	T	T	3	2	1
0	6	A	C	2	G	3	T	T	0	7	0
0	6	G	T	5	C	0	C	T	0	0	1
0	6	G	T	5	G	6	T	T	3	2	1
0	6	A	C	2	G	3	T	T	0	7	0
0	6	G	T	5	C	6	T	G	3	2	1
7	0	G	T	4	C	0	T	T	3	2	1
N	N	N	N	N	N	N	N	N	N	N	N
N	N	N	N	N	N	N	N	N	N	N	N
0	6	A	C	2	G	3	T	T	0	7	0
N	N	N	N	N	N	N	N	N	N	N	N
7	0	G	T	0	C	0	T	T	0	7	1
0	6	G	T	5	C	6	T	G	3	2	1
0	6	G	T	5	C	6	T	G	3	2	1
0	6	G	T	5	G	6	T	T	3	2	1
N	N	N	N	N	N	N	N	N	N	N	N
N	N	N	N	N	N	N	N	N	N	N	N
N	N	N	N	N	N	N	N	N	N	N	N
N	N	N	N	N	N	N	N	N	N	N	N
N	N	N	N	N	N	N	N	N	N	N	N
0	6	G	T	5	G	6	T	T	3	2	1
N	N	N	N	N	N	N	N	N	N	N	N
0	6	G	T	5	C	6	T	G	3	2	1
N	N	N	N	N	N	N	N	N	N	N	N
N	N	N	N	N	N	N	N	N	N	N	N
7	0	G	T	4	C	0	T	T	3	2	1
0	6	G	T	5	C	6	T	G	3	2	1
7	0	G	T	4	C	0	T	T	3	2	1
7	0	G	T	4	C	0	T	T	3	2	1
N	N	N	N	N	N	N	N	N	N	N	N
N	N	N	N	N	N	N	T	T	3	2	1
7	0	G	T	4	C	0	T	T	3	2	1
7	0	G	T	4	C	0	T	T	3	2	1
7	0	G	T	4	C	0	T	T	3	2	1
N	N	N	N	N	N	N	N	N	N	N	N
7	0	G	T	4	C	0	T	T	3	2	1
N	N	G	T	5	C	6	T	G	3	2	1
0	6	A	C	2	G	3	T	T	0	7	0
7	0	G	T	4	C	0	T	T	3	2	1
7	0	G	T	4	C	0	T	T	3	2	1
0	6	G	T	5	C	6	T	G	3	2	1
0	6	A	C	2	G	3	T	T	0	7	0
N	N	N	N	N	N	N	N	N	N	N	N
7	0	G	T	4	C	0	T	T	3	2	1
N	N	N	N	N	N	N	N	N	N	N	N
0	6	G	T	5	C	6	T	G	3	2	1
7	0	G	T	4	C	0	T	T	3	2	1
N	N	N	N	N	N	N	N	N	N	N	N
0	6	G	T	5	C	0	C	T	0	0	1
7	0	G	T	0	C	0	T	T	0	7	1
0	6	G	T	5	C	6	T	G	3	2	1
N	N	N	N	N	N	N	N	N	N	N	N
0	6	G	T	5	C	6	T	G	3	2	1
0	6	G	T	5	C	6	T	G	3	2	1
0	6	G	T	5	C	6	T	G	3	2	1
N	N	N	N	N	-	3	T	T	0	7	0

0	6	G	T	5	C	6	T	G	3	2	1
0	6	G	T	5	C	6	T	G	3	2	1
0	6	G	T	5	C	6	T	G	3	2	1
0	6	G	T	5	C	6	T	G	3	2	1
0	6	G	T	5	C	6	T	G	3	2	1
0	6	A	C	2	G	3	T	T	0	7	0
0	6	G	T	5	C	6	T	G	3	2	1
7	6	G	T	5	C	6	T	G	3	2	1
0	6	G	T	5	C	6	T	G	3	2	1
0	6	G	T	5	C	6	T	G	3	2	1
0	6	G	T	5	G	6	T	T	3	2	1
0	6	A	C	2	G	3	T	T	0	7	0
N	N	N	N	N	N	N	N	N	N	N	N
0	6	G	T	5	G	6	T	T	3	2	1
7	0	G	T	4	C	0	T	T	3	2	1
0	6	A	C	2	G	3	T	T	0	7	0
0	6	G	T	5	C	5	T	G	3	2	1
7	0	G	T	4	C	0	T	T	3	2	1
N	N	N	N	N	N	N	N	N	N	N	N
N	N	N	N	N	N	N	N	N	N	N	N
N	N	N	N	N	N	0	T	T	3	2	1
0	6	G	T	5	C	6	T	G	3	2	1
0	6	G	T	5	C	6	T	G	3	2	1
0	6	G	T	5	C	6	T	G	3	2	1
0	6	G	T	5	C	6	T	G	3	2	1
N	N	N	N	N	N	N	N	N	N	N	N
0	6	A	C	2	G	3	T	T	0	7	0
N	N	N	N	N	N	N	N	N	N	N	N
0	6	G	T	5	C	0	C	T	0	0	1
0	6	G	T	5	C	0	C	T	0	0	1
N	N	N	N	N	N	N	N	N	N	N	N
N	N	N	N	N	N	N	N	N	N	N	N
0	6	G	T	5	C	0	C	T	0	0	1
0	6	G	T	5	C	6	T	G	3	2	1
7	0	G	T	4	C	0	T	T	3	2	1
7	0	G	T	4	C	0	T	T	3	2	1
0	6	G	T	5	C	0	C	T	0	0	1
0	6	A	C	2	G	3	T	T	0	7	0
0	6	G	T	5	C	0	C	T	0	0	1
0	6	G	T	5	C	0	C	T	0	0	1
0	6	G	T	5	C	0	C	T	0	0	1
N	N	N	N	N	N	N	N	N	N	N	N
0	6	A	C	2	G	3	T	T	0	7	0
0	6	G	T	5	C	6	T	G	3	2	1
N	N	N	N	N	N	N	N	N	N	N	N
0	6	G	T	5	G	6	T	T	3	2	1
N	N	N	N	N	N	N	N	N	N	N	N
N	N	N	N	N	N	N	N	N	N	N	N
N	N	N	N	N	N	N	N	N	N	N	N
N	N	N	N	N	N	N	N	N	N	N	N
0	6	G	T	5	C	0	C	T	0	0	1
N	N	N	N	N	N	N	N	N	N	N	N
0	6	A	C	2	G	3	T	T	0	7	0
0	6	A	C	2	G	3	T	T	0	7	0
0	6	G	T	5	C	0	C	T	0	0	1
N	N	N	N	N	N	N	N	N	N	N	N

AL5_21	AL5_22	AL5_23	AL5_24	AL8_1	AL8_2	AL8_3	AL8_4	AL8_5	AL8_6	AL8_7	AL8_8	AL8_9
T	A	A	G	3	18	A	2	G	T	G	3	A
T	A	A	G	3	18	A	0	G	T	G	3	A
T	G	G	C	3	18	A	0	G	T	G	3	A
T	A	A	G	N	N	N	N	N	N	N	N	N
N	N	N	N	N	N	N	N	N	N	N	N	N
N	N	N	N	3	18	A	0	G	T	G	3	A
N	N	N	N	3	18	A	0	G	T	G	3	A
T	A	A	G	N	N	N	N	N	N	N	N	N
T	G	G	C	3	0	A	0	G	T	G	3	A
T	A	A	G	3	18	A	0	G	T	G	3	A
T	G	G	C	0	N	G	2	A	C	A	0	G
C	G	G	C	0	N	G	2	A	C	A	0	G
C	G	G	C	3	18	A	0	G	T	G	3	A
T	G	G	N	N	N	N	N	N	N	N	N	N
T	G	G	C	0	N	G	2	A	C	A	0	G
N	N	N	N	3	18	A	0	G	T	G	3	A
T	A	A	G	N	0	A	0	G	T	G	3	A
T	A	A	G	N	N	N	N	N	N	N	N	N
C	G	G	C	3	18	A	0	G	T	G	3	A
T	G	G	N	3	0	A	0	G	T	G	3	A
N	N	N	N	N	N	N	N	N	N	N	N	N
N	N	N	N	N	N	N	N	N	N	N	N	N
T	G	G	N	N	N	N	N	N	N	N	N	N
T	G	G	C	3	18	A	0	G	T	G	3	A
T	G	G	C	3	0	A	0	G	T	G	3	A
T	G	G	C	N	N	N	N	N	N	N	N	N
T	A	A	G	0	N	G	2	A	C	A	0	G
T	G	G	N	3	18	A	0	G	T	G	3	A
T	A	A	G	3	18	A	0	G	T	G	3	A
N	N	N	N	3	18	A	0	G	T	G	3	A
C	G	G	C	N	N	N	N	N	N	N	N	N
T	G	G	C	3	18	A	0	G	T	G	3	A
T	G	G	C	3	18	A	0	G	T	G	3	A
N	N	N	N	N	N	N	N	N	N	N	N	N
N	N	N	N	3	0	A	0	G	T	G	3	A
N	N	N	N	N	N	N	N	N	N	N	N	N
T	G	G	C	N	N	N	N	N	N	N	N	N
N	N	N	N	N	N	N	N	N	N	N	N	N
N	N	N	N	3	18	A	0	G	T	G	3	A
T	G	G	C	3	18	A	0	G	T	G	3	A
T	G	G	C	3	18	A	0	G	T	G	3	A
N	N	N	N	3	18	A	0	G	T	G	3	A
T	G	G	N	3	18	A	0	G	T	G	3	A
T	G	G	C	3	0	A	0	G	T	G	3	A
T	G	G	N	3	18	A	0	G	T	G	3	A
T	G	G	C	3	18	A	0	G	T	G	3	A
T	A	A	G	N	N	N	N	N	N	N	N	N
T	A	A	G	N	N	N	N	N	N	G	3	A
N	N	N	N	3	18	A	0	G	T	G	3	A
N	N	N	N	3	18	A	0	G	T	G	3	A
N	N	N	N	N	N	N	N	N	N	N	N	N
T	G	G	N	3	0	A	0	G	T	G	3	A
T	A	A	G	0	N	G	2	A	C	A	0	G
T	A	A	G	3	18	A	0	G	T	G	3	A
T	G	G	C	3	0	A	0	G	T	G	3	A
N	N	N	N	3	0	A	0	G	T	G	3	A

T	A	A	G	N	N	N	N	N	N	N	N	N
T	G	G	C	N	N	N	N	N	N	N	N	N
T	A	A	G	3	0	A	0	G	T	G	3	A
C	G	G	C	N	N	N	N	N	N	N	N	N
T	G	G	C	3	0	A	0	G	T	G	3	A
T	G	G	C	3	0	A	0	G	T	G	3	A
T	A	A	G	3	18	A	0	G	T	G	3	A
T	G	G	C	0	N	G	2	A	C	A	0	G
N	N	N	N	0	N	G	2	A	C	A	0	G
T	G	G	C	N	N	N	N	N	N	N	N	N
C	G	G	C	N	N	N	N	N	N	N	N	N
T	G	G	C	3	18	A	0	G	T	G	3	A
T	G	G	C	3	18	A	0	G	T	G	3	A
T	A	A	G	0	N	G	2	A	C	A	0	G
N	N	N	N	N	N	N	N	N	N	N	N	N
C	G	G	C	3	18	A	0	G	T	G	3	A
C	G	G	C	3	0	A	0	G	T	G	3	A
N	N	N	N	3	18	A	0	N	T	G	3	A
N	N	N	N	3	0	A	0	G	T	G	3	A
T	G	G	C	3	0	A	0	G	T	G	3	A
T	G	G	C	N	N	N	N	N	N	N	N	N

T	G	G	C	3	0	A	0	G	T	G	3	A
T	G	G	C	3	18	A	0	G	T	G	3	A
T	G	G	C	3	0	A	0	G	T	G	3	A
N	N	N	N	3	18	A	0	G	T	G	3	A
T	G	G	C	N	0	A	0	G	T	G	3	A
T	G	G	C	3	18	A	0	G	T	G	3	A
N	N	N	N	N	N	N	N	N	N	N	N	N
T	G	G	C	N	N	N	N	N	N	G	3	A
N	N	N	N	3	18	A	0	G	T	G	3	A
C	G	G	C	N	N	N	N	N	N	N	N	A
N	N	N	N	N	N	N	N	N	N	N	N	N
C	G	G	N	N	N	N	N	N	N	N	N	N
T	G	G	C	3	18	A	0	G	T	G	3	A
T	G	G	C	3	18	A	0	G	T	G	3	A
T	G	G	C	N	N	N	N	N	N	N	N	N
C	G	G	C	N	N	N	N	N	N	N	N	N
T	G	G	N	N	N	N	N	N	N	N	N	N
T	G	G	N	3	0	A	0	G	T	G	3	A
T	G	G	C	3	18	A	0	G	T	G	3	A
C	G	G	C	3	18	A	0	G	T	G	3	A
T	G	G	N	3	18	A	0	G	T	G	3	A
T	G	G	C	3	18	A	0	G	T	G	3	A
N	N	N	N	3	18	A	0	G	T	G	3	A
T	G	G	C	3	0	A	0	G	T	G	3	A
T	G	G	C	3	18	A	0	G	T	G	3	A
T	G	G	C	N	N	A	0	G	T	G	3	A
T	G	N	N	3	0	A	0	G	T	G	3	A
N	N	N	N	N	N	N	N	N	N	N	N	N
T	G	G	N	N	N	N	N	N	N	N	N	A
N	N	N	N	0	N	G	2	A	C	A	0	G
T	G	G	C	3	18	A	0	G	T	G	3	A
T	G	G	C	3	18	A	0	G	T	G	3	A
T	G	G	C	N	N	N	N	N	N	N	N	N
T	G	G	C	N	N	N	N	N	N	N	N	N
T	A	A	N	N	N	N	N	N	N	N	N	N

N	N	N	N	N	N	N	N	N	N	N	N	N
N	N	N	N	N	N	N	N	N	N	N	N	N
N	N	N	N	3	0	A	0	G	T	G	3	A
T	G	G	N	N	N	N	N	N	N	N	N	N
T	G	G	C	N	N	N	N	N	N	N	N	N
T	G	G	C	N	N	N	N	N	N	N	N	N
T	G	G	C	3	18	A	0	G	T	G	3	A
T	G	G	C	3	0	A	0	G	T	G	3	A
T	G	G	C	N	N	N	N	N	N	N	N	N
N	N	N	N	3	18	A	0	G	T	G	3	A
N	N	N	N	3	18	A	0	G	T	G	3	A
T	G	G	C	0	N	G	2	A	C	A	0	G
N	N	N	N	0	N	G	2	A	C	A	0	G
T	G	G	C	N	N	N	N	N	N	N	N	N
T	G	G	C	3	18	A	0	G	T	G	3	A
N	N	N	N	3	0	A	0	G	T	G	3	A
T	G	G	C	3	0	A	0	G	T	G	3	A
N	N	N	N	3	0	A	0	G	T	G	3	A
T	G	G	N	N	N	N	N	N	N	G	3	A
T	G	G	N	N	N	N	N	N	N	N	N	N
T	G	G	N	N	N	N	N	N	N	N	N	N
C	G	G	C	0	N	G	2	A	C	A	0	G
N	N	N	N	0	N	G	2	A	C	A	0	G
T	G	G	C	N	N	N	N	N	N	N	N	N
T	G	G	C	3	18	A	0	G	T	G	3	A
T	G	G	C	N	N	N	N	N	N	N	N	N
T	G	G	N	N	N	N	N	N	N	N	N	N
N	N	N	N	3	18	A	0	G	T	G	3	A
T	G	G	C	N	N	N	N	N	N	N	N	N
T	G	G	C	N	N	N	N	N	N	G	3	A
T	G	G	N	0	N	G	2	A	C	A	0	G
T	G	G	C	N	N	N	N	N	N	N	N	N
T	G	G	C	3	0	A	0	G	T	G	3	A
T	G	G	C	3	0	A	0	G	T	G	3	A
N	N	N	N	N	N	N	N	N	N	N	N	N
T	G	G	N	N	N	N	N	N	N	G	3	A
T	G	G	C	3	18	A	0	G	T	G	3	A
N	N	N	N	3	0	A	0	G	T	G	3	A
C	G	G	C	3	18	A	0	G	T	G	3	A
T	G	G	C	3	18	A	0	G	T	G	3	A
T	A	A	G	3	18	A	0	G	T	G	3	A
C	G	G	C	3	18	A	0	G	T	G	3	A
T	G	G	C	3	18	A	0	G	T	G	3	A
T	G	G	C	3	18	A	0	G	T	G	3	A
T	A	A	N	N	N	N	N	N	N	N	N	N
T	A	A	G	3	0	A	0	G	T	G	3	A
C	G	G	C	N	N	N	N	N	N	N	N	N
T	G	G	C	N	N	N	N	N	N	N	N	N
T	G	G	N	N	N	N	N	N	N	G	3	A
T	G	G	C	N	N	N	N	N	N	G	3	A
N	N	N	N	3	18	A	0	G	T	G	3	A
T	G	G	C	3	18	A	0	G	T	G	3	A
C	G	G	N	0	18	A	0	G	T	G	3	A
C	G	G	C	0	N	G	2	A	C	A	0	G
T	A	A	G	3	18	A	0	G	T	G	3	A
T	G	G	C	N	N	N	N	N	N	N	N	N
C	G	G	C	N	N	N	N	N	N	N	N	N

T	G	G	C	3	18	A	0	G	T	G	3	A
T	G	G	C	3	18	A	0	G	T	G	3	A
T	A	A	N	3	0	A	0	G	T	G	3	A
C	G	G	C	3	18	A	0	G	T	G	3	A
T	G	G	C	N	N	N	N	N	N	N	N	N
T	A	A	G	3	18	A	0	G	T	G	3	A
T	G	G	C	N	N	N	N	N	N	N	N	N
T	G	G	N	N	N	N	N	N	N	N	N	N
N	N	N	N	3	18	A	0	G	T	G	3	A
N	N	N	N	3	18	A	0	G	T	G	3	A
T	A	A	G	3	18	A	0	G	T	G	3	A
N	N	N	N	3	18	A	0	G	T	G	3	A
T	G	G	C	3	0	A	0	G	T	G	3	A
T	G	G	C	3	0	A	0	G	T	G	3	A
T	G	G	C	N	N	N	N	N	N	N	N	N
T	G	G	C	3	18	A	0	G	T	G	3	A
N	N	N	N	3	0	A	0	G	T	G	3	A
N	N	N	N	N	N	N	N	N	N	N	N	N
N	N	N	N	N	N	N	N	N	N	N	N	N
N	N	N	N	N	N	N	N	N	N	N	N	N
N	N	N	N	N	18	A	0	G	T	G	3	A
T	G	G	C	N	N	N	N	N	N	N	N	N
N	N	N	N	3	18	A	0	G	T	G	3	A
T	G	G	N	3	0	A	0	G	T	G	3	A
N	N	N	N	N	N	N	N	N	N	N	N	N
N	N	N	N	3	18	A	0	G	T	G	3	A
T	G	G	C	3	0	A	0	G	T	G	3	A
T	G	G	C	N	N	N	N	N	N	N	N	N
T	G	G	C	3	18	A	0	G	T	G	3	A
T	G	G	C	3	18	A	0	G	T	G	3	A
N	N	N	N	3	0	A	0	G	T	G	3	A
T	G	G	C	3	18	A	0	G	T	G	3	A
T	G	G	N	3	0	A	0	G	T	G	3	A
T	G	G	C	N	N	N	N	N	N	N	N	N
T	G	G	C	N	N	N	N	N	N	N	N	N
N	N	N	N	N	N	N	N	N	N	N	N	N
T	G	G	C	3	18	A	0	G	T	G	3	A
T	G	G	N	3	18	A	0	G	T	G	3	A
T	A	A	G	3	18	A	0	G	T	G	3	A
T	G	G	C	3	0	A	0	G	T	G	3	A
T	G	G	C	3	18	A	0	G	T	G	3	A
T	G	G	C	N	N	N	N	N	N	N	N	N
T	A	A	G	3	18	A	0	G	T	G	3	A
N	N	N	N	N	N	N	N	N	N	N	N	N
T	G	G	C	3	18	A	0	G	T	G	3	A
N	N	N	N	3	0	A	0	G	T	G	3	A
T	G	G	N	3	18	A	0	G	T	G	3	A
T	G	G	C	3	0	A	0	G	T	G	3	A
N	N	N	N	3	0	A	0	G	T	G	3	A
C	G	G	C	3	N	A	0	G	T	G	3	A
T	G	G	C	3	18	A	0	G	T	G	3	A
T	G	G	C	3	18	A	0	G	T	G	3	A
N	N	N	N	3	18	A	0	G	T	G	3	A
T	G	G	C	3	18	A	0	G	T	G	3	A
T	G	G	N	N	N	N	N	N	N	N	N	N
T	G	G	N	3	0	A	0	G	T	G	3	A
T	G	G	C	N	N	N	N	N	N	N	N	N

T	G	G	C	0	N	G	2	A	C	A	0	G
T	G	G	C	3	18	A	0	G	T	G	3	A
T	G	G	C	3	18	A	0	G	T	G	3	A
T	G	G	C	3	18	A	0	G	T	G	3	A
T	G	G	C	N	N	N	N	N	N	N	N	N
T	A	A	N	N	N	N	N	N	N	N	N	N
T	G	G	N	3	18	A	0	G	T	G	3	A
T	G	G	N	3	18	A	0	G	T	G	3	A
T	G	G	C	3	18	A	0	G	T	G	3	A
T	G	G	N	0	N	G	2	A	C	A	0	G
T	G	G	C	3	18	A	0	G	T	G	3	A
T	A	A	G	N	N	N	N	N	N	N	N	N
N	N	N	N	N	N	N	N	N	N	N	N	N
T	G	G	N	N	N	N	N	N	N	N	N	N
T	G	G	C	3	18	A	0	G	T	G	3	A
T	A	A	G	N	N	N	N	N	N	N	N	N
T	G	G	C	3	18	A	0	G	T	G	3	A
T	G	G	C	3	0	A	0	G	T	G	3	A
N	N	N	N	N	N	N	N	N	N	N	N	N
N	N	N	N	3	0	A	0	G	T	G	3	A
T	G	G	C	3	0	A	0	G	T	G	3	A
T	G	G	N	0	N	G	2	A	C	A	0	G
T	G	G	C	3	0	A	0	G	T	G	3	A
T	G	G	C	0	N	G	2	A	C	A	0	G
T	G	G	C	3	0	A	0	G	T	G	3	A
N	N	N	N	N	N	N	N	N	N	N	N	N
T	A	A	G	3	18	A	0	G	T	G	3	A
N	N	N	N	3	0	A	0	G	T	G	3	A
C	G	G	C	3	0	A	0	G	T	G	3	A
C	G	G	C	3	0	A	0	G	T	G	3	A
N	N	N	N	3	18	A	0	G	T	G	3	A
N	N	N	N	3	18	A	0	G	T	G	3	A
C	G	G	C	3	18	A	0	G	T	G	3	A
T	G	G	N	3	18	A	0	G	T	G	3	A
T	G	G	C	3	0	A	0	G	T	G	3	A
T	G	G	C	3	0	A	0	G	T	G	3	A
C	G	G	C	3	0	A	0	G	T	G	3	A
T	A	A	G	3	18	A	0	G	T	G	3	A
C	G	G	C	0	N	G	2	A	C	A	0	G
C	G	G	C	N	N	N	N	N	N	N	N	N
C	G	G	C	3	18	A	0	G	T	G	3	A
N	N	N	N	3	18	A	0	G	T	G	3	A
T	A	A	G	3	18	A	0	G	T	G	3	A
T	G	G	C	3	18	A	0	G	T	G	3	A
N	N	N	N	N	N	N	N	N	N	N	N	N
T	G	G	C	3	18	A	0	G	T	G	3	A
N	N	N	N	3	18	A	0	G	T	G	3	A
N	N	N	N	0	N	G	2	A	C	A	0	G
N	N	N	N	3	18	A	0	G	T	G	3	A
N	N	N	N	3	18	A	0	G	T	G	3	A
C	G	G	C	N	N	N	N	N	N	N	N	N
N	N	N	N	N	N	N	N	N	N	N	N	N
T	A	A	G	0	N	G	2	A	C	A	0	G
T	A	A	G	3	18	A	0	G	T	G	3	A
C	G	G	C	N	N	N	N	N	N	N	N	N
N	N	N	N	N	N	N	N	N	N	N	N	N

AL8_10	AL8_11	AL8_12	AL8_13	AL8_14	AL8_15	AL8_16	AL8_17	AL8_18	AL8_19	AL8_20
C	C	A	G	T	T	G	A	A	A	T
C	C	A	G	T	T	G	A	A	A	T
C	C	A	G	T	T	G	A	A	A	T
N	N	N	N	N	N	N	N	N	N	N
N	N	N	N	N	N	N	N	N	N	N
C	C	A	G	T	T	G	A	A	A	T
C	C	A	G	T	T	G	A	A	A	T
N	N	N	N	N	N	N	N	N	N	N
G	C	A	G	T	T	A	A	G	A	T
C	C	A	G	T	T	G	A	A	A	T
C	G	G	C	C	A	G	G	G	G	C
C	G	G	C	C	A	G	G	G	G	C
C	C	A	G	T	T	G	A	A	A	T
N	N	N	N	N	N	N	N	N	N	N
C	G	G	C	C	A	G	G	G	G	C
C	C	A	G	T	T	G	A	A	A	T
G	C	A	G	T	T	A	A	G	A	T
N	N	N	N	N	N	N	N	N	N	N
C	C	A	G	T	T	G	A	A	A	T
G	C	A	G	T	T	A	A	G	A	T
N	N	N	N	N	N	N	N	N	N	N
N	N	N	N	N	N	N	N	N	N	N
N	N	N	N	N	N	N	N	N	N	N
C	C	A	G	T	T	G	A	A	A	T
G	C	A	G	T	T	A	A	G	A	T
N	N	N	N	N	N	N	N	N	N	N
C	C	G	C	N	A	G	G	G	G	C
C	C	A	G	T	T	G	A	A	A	T
C	C	A	G	T	T	G	A	A	A	T
C	C	A	G	T	T	G	A	A	A	T
N	N	N	N	N	N	N	N	N	N	N
C	C	A	G	T	T	G	A	A	A	T
G	C	A	G	T	T	G	A	A	A	T
N	N	N	N	N	N	N	N	N	N	N
G	C	A	G	T	T	A	A	G	A	T
N	N	N	N	N	N	N	N	N	N	N
N	N	N	N	N	N	N	N	N	N	N
N	N	N	N	N	N	N	N	N	N	N
C	C	A	G	T	T	G	A	A	A	T
C	C	A	G	T	T	G	A	A	A	T
C	C	A	G	T	T	G	A	A	A	T
C	C	A	G	T	T	G	A	A	A	T
C	C	A	G	T	T	G	A	A	A	T
G	C	A	G	T	T	A	A	G	A	T
C	C	A	G	T	T	G	A	A	A	T
C	C	A	G	T	T	G	A	A	A	T
N	N	N	N	N	N	N	N	N	N	N
C	C	A	G	T	T	G	A	A	A	T
C	C	A	G	T	T	G	A	A	A	T
C	C	A	G	T	T	G	A	A	A	T
N	N	N	N	N	N	N	N	N	N	N
G	C	A	G	T	T	A	A	G	A	T
C	N	G	C	C	A	G	G	G	G	C
C	C	A	G	T	T	G	A	A	A	T
G	C	A	G	T	T	A	A	G	A	T
G	N	A	G	T	T	A	A	G	A	T

N	N	N	N	N	N	N	N	N	N	N
N	N	N	N	N	N	N	N	N	N	N
G	C	A	G	T	T	A	A	G	A	T
N	N	N	N	N	N	N	N	N	N	N
G	C	A	G	T	T	A	A	C	A	T
G	C	A	G	T	T	A	A	G	A	T
C	C	A	G	T	T	G	A	A	A	T
C	N	G	C	C	A	G	G	G	G	C
C	G	G	C	C	A	G	G	G	G	C
N	N	N	N	N	N	N	N	N	N	N
N	N	N	N	N	N	N	N	N	N	N
C	C	A	G	T	T	G	A	A	A	T
C	C	A	G	T	T	G	A	A	A	T
C	G	G	C	C	A	G	G	G	G	C
N	N	N	N	N	N	N	N	N	N	N
C	C	A	G	T	T	G	A	A	A	T
G	C	A	G	T	T	A	A	G	A	T
C	C	A	G	T	T	G	A	A	A	T
G	C	A	G	T	T	A	A	G	A	T
G	N	A	G	T	T	A	A	G	A	T
N	N	N	N	N	N	N	N	N	N	N

G	C	A	G	T	T	A	A	G	A	T
C	C	A	G	T	T	G	A	A	A	T
G	C	A	G	T	T	A	A	G	A	T
C	C	A	G	T	T	G	A	A	A	T
G	C	A	G	T	T	A	A	G	A	T
C	C	A	G	T	T	G	A	A	A	T
N	N	N	N	N	N	N	N	N	N	N
G	C	A	G	T	T	A	A	G	A	T
C	C	A	G	T	T	G	A	A	A	T
G	C	A	G	T	T	A	A	G	A	T
N	N	N	N	N	N	N	N	N	N	N
N	N	N	N	N	N	N	N	N	N	N
C	C	A	G	T	T	G	A	A	A	T
C	C	A	G	T	T	G	A	A	A	T
N	N	N	N	N	N	N	N	N	N	N
N	N	N	N	N	N	N	N	N	N	N
N	N	N	N	N	N	N	N	N	N	N
G	C	A	G	T	T	A	A	G	A	T
C	C	A	G	T	T	G	A	A	A	T
C	C	A	G	T	T	G	A	A	A	T
C	C	A	G	T	T	G	A	A	A	T
C	C	A	G	T	T	G	A	A	A	T
C	C	A	G	T	T	G	A	A	A	T
G	C	A	G	T	T	A	A	G	A	T
C	C	A	G	T	T	G	A	A	A	T
C	C	A	G	T	T	G	A	A	A	T
G	C	A	G	T	T	A	A	G	A	T
N	N	N	N	N	N	N	N	N	N	N
G	C	A	G	T	T	A	A	G	A	T
C	G	G	C	C	A	G	G	G	G	C
C	C	A	G	T	T	G	A	A	A	T
C	C	A	G	T	T	G	A	A	A	T
N	N	N	N	N	N	N	N	N	N	N
N	N	N	N	N	N	N	N	N	N	N
N	N	N	N	N	N	N	N	N	N	N

N	N	N	N	N	N	N	N	N	N	N
N	N	N	N	N	N	N	N	N	N	N
G	C	A	G	T	T	A	A	G	A	T
N	N	N	N	N	N	N	N	N	N	N
N	N	N	N	N	N	N	N	N	N	N
N	N	N	N	N	N	N	N	N	N	N
C	C	A	G	T	T	G	A	A	A	T
G	C	A	G	T	T	A	A	G	A	T
N	N	N	N	N	N	N	N	N	N	N
C	C	A	G	T	T	G	A	A	A	T
C	C	A	G	T	T	G	A	A	A	T
C	G	G	C	C	A	G	G	G	G	C
C	C	G	C	C	A	G	G	G	G	C
N	N	N	N	N	N	N	N	N	N	N
C	C	A	G	T	T	G	A	A	A	T
G	C	A	G	T	T	A	A	G	A	T
G	C	A	G	T	T	A	A	G	A	T
G	C	A	G	T	T	A	A	G	A	T
G	C	A	G	T	T	G	A	A	A	T
N	N	N	N	N	N	N	N	N	N	N
N	N	N	N	N	N	N	N	N	N	N
C	G	G	C	C	A	G	G	G	G	C
C	N	G	C	C	A	G	G	G	G	C
N	N	N	N	N	N	N	N	N	N	N
C	C	A	G	T	T	G	A	A	A	T
N	N	N	N	N	N	N	N	N	N	N
N	N	N	N	N	N	N	N	N	N	N
C	C	A	G	T	T	G	A	A	A	T
N	N	N	N	N	N	N	N	N	N	N
G	C	A	G	T	T	G	A	A	A	T
C	G	G	C	C	A	G	G	G	G	C
N	N	N	N	N	N	N	N	N	N	N
G	C	A	G	T	T	A	A	G	A	T
G	C	A	G	T	T	A	A	G	A	T
N	N	N	N	N	N	N	N	N	N	N
G	C	A	G	T	T	A	A	G	A	T
C	C	A	G	T	T	G	A	A	A	T
G	C	A	G	T	T	A	A	G	A	T
C	C	A	G	T	T	G	A	A	A	T
C	C	A	G	T	T	G	A	A	A	T
C	C	A	G	T	T	G	A	A	A	T
C	C	A	G	T	T	G	A	A	A	T
C	C	A	G	T	T	G	A	A	A	T
C	C	A	G	T	T	G	A	A	A	T
N	N	N	N	N	N	N	N	N	N	N
G	C	A	G	T	T	A	A	G	A	T
N	N	N	N	N	N	N	N	N	N	N
N	N	N	N	N	N	N	N	N	N	N
C	C	A	G	T	T	G	A	A	A	T
G	C	A	G	T	T	G	A	A	A	T
C	C	A	G	T	T	G	A	A	A	T
C	C	A	G	T	T	G	A	A	A	T
C	C	A	G	T	T	G	A	A	A	T
C	G	G	C	C	A	G	G	G	G	C
C	C	A	G	T	T	G	A	A	A	T
N	N	N	N	N	N	N	N	N	N	N
N	N	N	N	N	N	N	N	N	N	N

C	C	A	G	T	T	G	A	A	A	T
C	C	A	G	T	T	G	A	A	A	T
G	C	A	G	T	T	A	A	G	A	T
C	C	A	G	T	T	G	A	A	A	T
N	N	N	N	N	N	N	N	N	N	N
C	C	A	G	T	T	G	A	A	A	T
N	N	N	N	T	T	G	A	A	A	T
N	N	N	N	N	N	N	N	N	N	N
C	C	A	G	T	T	G	A	A	A	T
C	C	A	G	T	T	G	A	A	A	T
C	C	A	G	T	T	G	A	A	A	T
C	C	A	G	T	T	G	A	A	A	T
G	C	A	G	T	T	A	A	G	A	T
G	C	A	G	T	T	A	A	G	A	T
N	N	N	N	N	N	N	N	N	N	N
C	C	A	G	T	T	G	A	A	A	T
G	C	A	G	T	T	A	A	G	A	T
N	N	N	N	N	N	N	N	N	N	N
N	N	N	N	N	N	N	N	N	N	N
N	N	N	N	N	N	N	N	N	N	N
C	C	A	G	T	T	G	A	A	A	T
N	N	N	N	N	N	N	N	N	N	N
C	C	A	G	T	T	G	A	A	A	T
G	C	A	G	T	T	A	A	G	A	T
N	N	N	N	N	N	N	N	N	N	N
C	C	A	G	T	T	G	A	A	A	T
G	C	A	G	T	T	A	A	G	A	T
N	N	N	N	N	N	N	N	N	N	N
C	C	A	G	T	T	G	A	A	A	T
C	C	A	G	T	T	G	A	A	A	T
G	C	A	G	T	T	A	A	G	A	T
C	C	A	G	T	T	G	A	A	A	T
G	C	A	G	T	T	A	A	G	A	T
N	N	N	N	N	N	N	N	N	N	N
N	N	N	N	N	N	N	N	N	N	N
N	N	N	N	N	N	N	N	N	N	N
C	C	A	G	T	T	G	A	A	A	T
C	C	A	G	T	T	G	A	A	A	T
C	C	A	G	T	T	G	A	A	A	T
G	C	A	G	T	T	A	A	G	A	T
C	C	A	G	T	T	G	A	A	A	T
N	N	N	N	N	N	N	N	N	N	N
C	C	A	G	T	T	G	A	A	A	T
N	N	N	N	N	N	N	N	N	N	N
C	C	A	G	T	T	G	A	A	A	T
G	C	A	G	T	T	A	A	G	A	T
C	C	A	G	T	T	G	A	A	A	T
G	C	A	G	T	T	A	A	G	A	T
G	C	A	G	T	T	A	A	G	A	T
C	C	A	G	T	T	G	A	A	A	T
C	C	A	G	T	T	G	A	A	A	T
C	C	A	G	T	T	G	A	A	A	T
C	C	A	G	T	T	G	A	A	A	T
C	C	A	G	T	T	G	A	A	A	T
N	N	N	N	N	N	N	N	N	N	N
G	C	A	G	T	T	A	A	G	A	T
N	N	N	N	N	N	N	N	N	N	N

C	G	G	C	C	A	G	G	G	G	C
C	C	A	G	T	T	G	A	A	A	T
C	C	A	G	T	T	G	A	A	A	T
C	C	A	G	T	T	G	A	A	A	T
N	C	A	G	T	T	G	A	A	A	T
N	N	N	N	N	N	N	N	N	N	N
C	C	A	G	T	T	G	A	A	A	T
C	C	A	G	T	T	G	A	A	A	T
C	C	A	G	T	T	G	A	A	A	T
C	G	G	C	C	A	G	G	G	G	C
C	C	A	G	T	T	G	A	A	A	T
N	N	N	N	N	N	N	N	N	N	N
N	N	N	N	N	N	N	N	N	N	N
N	N	N	N	N	N	N	N	N	N	N
C	C	A	G	T	T	G	A	A	A	T
N	N	N	N	N	N	N	N	N	N	N
C	C	A	G	T	T	G	A	A	A	T
G	C	A	G	T	T	A	A	G	A	T
N	N	N	N	N	N	N	N	N	N	N
G	C	A	G	T	T	A	A	G	A	T
G	C	A	G	T	T	A	A	G	A	T
C	G	G	C	N	A	G	G	G	G	C
G	C	A	G	T	T	A	A	G	A	T
C	G	G	C	C	C	G	G	G	G	C
G	C	A	G	T	T	A	A	G	A	T
N	N	N	N	N	N	N	N	N	N	N
C	C	A	G	T	T	G	A	A	A	T
G	C	A	G	T	T	A	A	G	A	T
G	C	A	G	T	T	A	A	G	A	T
G	C	A	G	T	T	A	A	G	A	T
C	C	A	G	T	T	G	A	A	A	T
C	C	A	G	T	T	G	A	A	A	T
C	C	A	G	T	T	G	A	A	A	T
C	C	A	G	T	T	G	A	A	A	T
G	C	A	G	T	T	A	A	G	A	T
G	C	A	G	T	T	A	A	G	A	T
G	C	A	G	T	T	A	A	G	A	T
C	C	A	G	T	T	G	A	A	A	T
C	G	G	C	C	A	G	G	G	G	C
N	N	N	N	N	N	N	N	N	N	N
C	C	A	G	T	T	G	A	A	A	T
C	C	A	G	T	T	G	A	A	A	T
C	C	A	G	T	T	G	A	A	A	T
C	C	A	G	T	T	G	A	A	A	T
N	N	N	N	N	N	N	N	N	N	N
C	C	A	G	T	T	G	A	A	A	T
C	C	A	G	T	T	G	A	A	A	T
C	G	G	C	C	A	G	G	G	G	C
C	C	A	G	T	T	G	A	A	A	T
C	C	A	G	T	T	G	A	A	A	T
N	N	N	N	N	N	N	N	N	N	N
N	N	N	N	N	N	N	N	N	N	N
C	N	G	C	C	A	G	G	G	G	C
C	C	A	G	T	T	G	A	A	A	T
N	N	N	N	N	N	N	N	N	N	N
N	N	N	N	N	N	N	N	N	N	N

AL8_21	AL8_22	AL8_23	AL8_24	AL8_25	AL8_26	anti1	anti2	anti3	anti4	anti5	anti6	anti7
G	T	G	T	T	T	G	T	15	G	4	T	1
G	T	G	T	T	T	G	T	0	G	0	T	1
G	T	G	T	T	C	G	A	0	G	N	N	N
N	N	N	N	N	N	G	A	0	G	0	T	1
N	N	N	N	N	N	G	A	0	G	0	T	1
G	T	G	T	T	C	G	T	0	N	4	C	N
G	T	G	T	T	C	G	T	0	A	0	T	1
N	N	N	N	N	N	G	A	15	A	0	T	1
G	T	T	T	T	C	G	A	0	G	0	T	1
G	T	G	T	T	C	G	A	0	G	0	T	1
C	C	G	C	G	C	G	A	15	A	0	T	1
C	C	G	C	G	C	G	A	0	G	N	N	N
G	T	G	T	T	C	C	T	0	A	0	C	0
N	N	N	N	N	N	G	A	0	G	0	T	1
C	C	G	C	G	C	G	A	0	G	0	T	1
G	T	G	T	T	N	G	A	0	G	0	T	1
G	T	T	T	T	N	G	A	0	G	0	T	1
N	N	N	N	N	N	G	T	0	G	0	T	1
G	T	G	T	T	C	G	A	0	G	0	T	1
G	T	T	T	T	C	G	T	0	G	0	T	1
N	N	N	N	N	N	C	T	0	A	0	C	0
N	N	N	N	N	N	N	N	N	N	N	N	N
N	N	N	N	N	N	G	A	0	G	0	T	1
G	T	G	T	T	T	G	T	0	G	0	T	1
G	T	T	T	T	C	C	T	0	A	0	C	0
N	N	N	N	N	N	G	A	0	G	0	T	1
C	C	G	C	G	C	C	T	0	A	0	C	0
G	T	G	T	T	C	G	A	0	G	0	T	1
G	T	G	T	T	C	N	N	N	N	N	N	N
G	T	G	T	T	C	G	A	0	G	0	T	1
N	N	N	N	N	N	N	N	N	N	N	N	N
G	T	G	T	T	C	G	A	0	G	N	N	N
G	T	N	N	N	N	N	N	N	N	N	N	N
N	N	N	N	N	N	C	T	0	A	0	C	0
G	T	T	T	T	C	G	A	0	G	0	T	1
N	N	N	N	N	N	G	T	0	G	0	T	1
N	N	N	N	N	N	N	N	N	N	N	N	N
N	N	N	N	N	N	N	N	N	N	N	N	N
G	T	G	T	T	C	C	T	0	A	0	T	1
G	T	G	T	T	N	G	A	0	G	0	T	1
G	T	G	T	T	C	N	N	N	N	N	N	N
G	T	G	T	T	C	G	A	0	G	0	T	1
G	T	G	T	T	T	G	A	0	G	0	T	1
G	T	T	T	T	C	G	A	0	G	0	T	1
G	T	G	T	T	C	G	T	0	G	0	T	1
G	T	G	T	T	T	G	A	0	G	N	N	N
N	N	N	N	N	N	G	A	0	G	0	T	1
G	T	G	T	T	C	N	T	0	G	0	T	1
G	T	G	T	T	T	G	T	0	G	0	T	1
G	T	G	T	T	T	G	T	0	G	0	T	1
N	N	N	N	N	N	G	A	0	G	0	T	N
G	T	T	T	T	C	G	A	0	G	0	T	1
C	C	G	C	G	C	G	A	0	G	0	T	1
G	T	G	T	T	T	G	T	0	G	0	T	1
G	T	T	T	T	C	G	A	0	G	0	T	1
N	T	T	T	T	C	N	N	N	N	N	N	N

N	N	N	N	N	N	C	T	0	A	0	C	0
N	N	N	N	N	N	N	N	N	N	N	N	N
G	T	T	T	T	C	G	A	0	G	0	T	1
N	N	N	N	N	N	N	N	N	N	N	N	N
G	T	T	T	T	C	G	A	0	G	0	T	1
G	T	T	T	T	C	G	A	15	A	0	T	1
G	T	G	T	T	T	G	A	0	G	0	T	1
C	C	G	C	G	C	G	A	0	G	0	T	1
C	C	G	C	G	C	G	A	0	G	0	T	1
N	N	N	N	N	N	G	A	0	G	0	T	1
N	N	N	N	N	N	G	A	0	G	0	T	1
G	T	G	T	T	C	C	T	0	A	0	C	0
G	T	G	T	T	C	N	N	N	N	N	N	N
C	C	G	C	G	C	G	A	0	A	4	T	2
N	N	N	N	N	N	N	N	N	N	N	N	N
G	T	G	T	T	C	G	A	15	A	0	C	1
G	T	T	T	T	C	C	T	0	A	0	C	N
G	T	G	T	T	C	G	A	0	A	4	C	1
G	T	T	T	T	C	G	A	0	A	4	C	1
G	T	T	T	N	N	C	T	0	A	0	C	0
N	N	N	N	N	N	G	A	0	A	4	T	2

G	T	T	T	T	C	N	N	N	N	N	N	N
G	T	G	T	T	C	C	T	0	A	0	C	0
G	T	T	T	T	C	C	T	0	A	0	C	0
G	T	G	T	T	C	C	T	0	A	0	C	0
G	T	T	T	T	C	G	A	0	A	4	T	2
G	T	G	T	T	N	C	T	0	A	0	C	0
N	N	N	N	N	N	G	A	0	A	N	N	N
G	T	T	T	T	C	G	T	15	A	4	C	1
G	T	G	T	T	C	C	T	0	A	0	C	0
G	T	T	T	T	C	G	A	15	A	0	C	1
N	N	N	N	N	N	G	T	0	N	4	C	1
N	N	N	N	N	N	G	A	0	G	N	N	N
G	T	G	T	T	C	G	T	15	A	4	C	1
G	T	G	T	T	C	G	A	0	G	0	T	1
N	N	N	N	N	N	G	A	0	G	0	T	1
N	N	N	N	N	N	C	T	0	A	0	C	0
N	N	N	N	N	N	C	T	0	A	0	C	0
G	T	T	T	T	C	C	T	0	A	0	C	0
G	T	G	T	T	N	G	A	0	A	4	C	1
G	T	G	T	T	C	G	A	15	A	N	N	N
G	T	G	T	T	C	G	T	0	N	4	C	1
G	T	G	T	T	C	G	A	15	A	0	C	1
G	T	G	T	T	C	G	A	0	N	N	N	N
G	T	T	T	T	C	G	A	0	A	4	C	1
G	T	G	T	T	C	G	A	0	A	4	T	2
G	T	G	T	T	C	C	T	0	A	0	C	1
G	T	T	T	T	C	G	A	15	A	0	C	1
N	N	N	N	N	N	G	T	0	N	4	C	1
G	T	T	T	T	C	N	N	N	N	N	N	N
C	C	G	C	G	C	G	A	0	G	0	T	1
G	T	G	T	T	C	G	T	0	G	0	T	1
G	T	G	T	T	C	G	A	0	G	0	T	1
N	N	N	N	N	N	G	A	0	G	0	T	1
N	N	N	N	N	N	G	T	0	G	N	N	N
N	N	N	N	N	N	G	A	15	A	0	C	N

N	N	N	N	N	N	G	A	15	A	0	C	1
N	N	N	N	N	N	G	T	0	G	0	T	1
G	T	T	T	T	C	G	A	0	G	N	N	N
N	N	N	N	N	N	G	A	0	G	0	T	1
N	N	N	N	N	N	G	A	15	A	4	C	1
N	N	N	N	N	N	G	A	0	G	0	T	1
G	T	G	T	T	C	G	A	15	A	0	T	1
G	T	T	T	T	C	C	A	0	A	0	T	1
N	N	N	N	N	N	G	A	0	G	0	T	1
G	T	G	T	T	T	G	A	0	G	0	T	N
G	T	G	T	T	C	C	T	0	A	0	C	0
C	C	G	C	G	C	G	T	0	G	0	T	1
C	C	G	C	G	C	N	N	N	N	N	N	N
N	N	N	N	N	N	G	A	0	G	0	T	1
G	T	G	T	T	C	G	A	0	G	0	T	1
G	T	T	T	T	C	G	A	0	G	0	T	1
G	T	T	T	T	C	N	N	N	N	N	N	N
G	T	T	T	T	C	G	A	0	G	0	T	N
G	T	G	T	T	C	G	T	0	G	0	T	1
N	N	N	N	N	N	N	N	N	N	N	N	N
N	N	N	N	N	N	G	A	0	G	0	T	1
C	C	G	C	G	C	G	A	0	G	0	T	1
C	C	G	C	G	C	G	A	0	G	0	T	1
N	N	N	N	N	N	N	T	0	A	0	C	0
G	T	G	T	T	C	G	A	0	G	0	T	1
N	N	N	N	N	N	C	T	0	A	0	C	0
N	N	N	N	N	N	G	T	0	G	0	T	1
G	T	G	T	T	C	G	T	0	G	0	T	1
N	N	N	N	N	N	G	A	0	G	0	T	1
G	T	G	T	T	C	G	A	0	G	0	T	1
C	C	G	C	G	C	G	T	0	G	0	T	1
N	N	N	N	N	N	G	T	0	N	4	C	1
G	T	T	T	T	N	G	T	0	G	0	T	1
G	T	T	T	T	C	C	T	0	A	4	C	1
N	N	N	N	N	N	G	A	0	G	N	N	N
G	T	T	T	T	C	G	T	0	A	0	C	0
G	T	G	T	T	N	C	T	0	A	4	C	1
G	T	T	T	T	N	C	T	0	A	0	C	0
G	T	G	T	T	T	N	N	N	N	N	N	N
G	T	G	T	T	C	C	T	0	A	0	C	0
G	T	G	T	T	N	C	T	0	A	0	C	0
G	T	G	T	T	N	G	T	0	N	4	C	1
G	T	G	T	T	C	N	N	N	N	N	N	N
G	T	G	T	T	N	G	A	0	G	0	T	1
N	N	N	N	N	N	C	A	0	A	0	T	1
G	T	T	T	T	C	G	A	0	G	0	T	1
N	N	N	N	N	N	C	T	0	A	0	C	0
N	N	N	N	N	N	G	A	15	A	0	T	1
G	T	G	T	T	C	G	A	0	G	0	T	1
G	T	G	T	T	T	G	A	0	G	0	T	1
G	T	G	T	T	N	G	A	0	G	0	T	1
G	T	G	T	T	T	N	N	N	N	N	N	N
G	T	G	T	T	C	N	N	N	N	N	N	N
C	C	G	C	N	N	G	A	0	G	0	T	1
G	T	G	T	T	C	G	A	0	G	0	T	1
N	N	N	N	N	N	G	A	0	G	0	T	1
N	N	N	N	N	N	G	T	0	G	0	T	1

G	T	G	T	T	C	N	N	N	N	N	N	N
G	T	G	T	T	N	N	N	N	N	N	N	N
G	T	T	T	T	C	N	N	N	N	N	N	N
G	T	G	T	T	N	G	A	0	G	0	T	1
N	N	N	N	N	N	N	N	N	N	N	N	N
G	T	G	T	T	N	G	T	0	G	0	T	1
G	T	G	T	T	C	N	N	N	N	N	N	N
N	N	N	N	N	N	G	T	0	G	0	T	1
G	T	G	T	T	N	G	A	0	G	N	N	N
G	T	G	T	T	N	G	A	15	A	0	T	1
G	T	G	T	T	N	N	N	N	N	N	N	N
G	T	G	T	T	C	N	N	N	N	N	N	N
G	T	T	T	T	N	G	A	0	G	0	T	1
G	T	T	T	T	C	G	A	0	G	0	T	1
N	N	N	N	N	N	G	A	0	G	0	T	1
G	T	G	T	T	C	G	A	0	G	0	T	1
G	T	T	T	N	N	C	T	0	A	N	N	N
N	N	N	N	N	N	G	A	15	A	4	C	1
N	N	N	N	N	N	G	T	0	G	0	T	1
N	N	N	N	N	N	N	N	N	N	N	N	N
G	T	G	T	T	C	G	T	0	G	0	T	1
N	N	N	N	N	N	G	A	0	G	0	T	1
G	T	G	T	T	C	G	T	0	G	0	T	1
G	T	T	T	T	C	G	A	0	G	N	N	N
N	N	N	N	N	N	G	A	15	A	0	C	1
G	T	G	T	T	C	G	A	15	A	0	C	1
G	T	T	T	T	C	G	A	15	A	0	C	1
N	N	N	N	N	N	C	T	0	A	4	C	1
G	T	G	T	T	N	G	A	15	A	0	C	1
G	T	G	T	T	N	C	T	0	A	4	C	N
G	T	T	T	T	C	G	A	0	G	0	T	1
G	T	G	T	T	C	G	A	0	G	0	T	1
G	T	T	T	T	N	G	A	0	G	0	T	1
N	N	N	N	N	N	G	A	15	A	0	T	1
N	N	N	N	N	N	G	A	0	G	0	T	1
N	N	N	N	N	N	G	A	0	G	0	T	1
G	T	G	T	T	N	G	A	0	G	0	T	1
G	T	G	T	T	C	G	A	0	G	0	T	1
G	T	G	T	T	C	G	A	0	A	4	C	1
G	T	T	T	T	C	G	A	15	A	0	C	N
G	T	G	T	T	C	G	A	0	G	0	C	N
N	N	N	N	N	N	C	T	0	A	4	C	1
G	T	G	T	T	C	N	N	N	N	N	N	N
N	N	N	N	N	N	N	N	N	N	N	N	N
G	T	G	T	T	N	G	A	15	A	0	C	1
G	T	T	T	T	C	G	A	15	A	0	C	1
G	T	G	T	T	C	G	A	15	A	0	C	1
G	T	T	T	T	C	G	A	15	A	0	C	1
G	T	T	T	T	C	G	A	15	A	0	C	1
G	T	G	T	T	C	G	T	0	N	4	C	1
G	T	G	T	T	C	G	A	0	A	4	C	1
G	T	G	T	T	N	G	T	0	G	0	T	1
G	T	G	T	T	N	C	T	0	A	0	C	0
G	T	G	N	N	N	C	T	0	A	0	C	0
N	N	N	N	N	N	C	T	0	A	0	C	0
G	T	T	T	T	C	G	A	0	G	0	T	1
N	N	N	N	N	N	G	A	0	G	0	T	N

C	C	G	C	N	N	G	A	0	G	0	T	1
G	T	G	T	T	T	G	A	0	G	0	T	1
G	T	G	T	T	T	G	A	0	G	0	T	1
G	T	G	T	T	T	C	T	0	A	0	C	0
G	T	G	T	T	C	G	A	0	G	0	T	1
N	N	N	N	N	N	G	T	0	G	0	T	1
G	T	G	T	T	C	N	N	N	N	N	N	N
G	T	G	T	T	N	G	A	0	G	0	T	1
G	T	G	T	T	T	G	A	0	G	0	T	1
C	C	G	C	G	C	G	A	0	G	0	T	1
G	T	G	T	T	N	G	A	0	G	0	T	1
N	N	N	N	N	N	G	A	0	G	0	T	1
N	N	N	N	N	N	G	A	15	A	4	C	1
N	N	N	N	N	N	G	T	0	G	0	T	1
G	T	G	T	T	C	G	A	0	G	0	T	1
N	N	N	N	N	N	G	A	0	G	0	T	1
G	T	G	T	T	C	G	A	0	G	0	T	1
G	T	T	T	T	C	C	T	0	A	0	C	0
N	N	N	N	N	N	C	T	0	A	4	C	1
G	T	T	T	T	C	G	A	0	G	0	T	1
G	T	T	T	T	C	G	A	0	G	0	T	1
C	C	G	C	G	C	N	N	N	N	N	N	N
G	T	T	N	N	N	G	T	0	G	0	T	1
C	C	G	C	G	C	G	T	0	G	0	T	1
G	T	T	T	T	N	G	A	0	G	0	T	1
N	N	N	N	N	N	N	N	N	N	N	N	N
G	T	G	T	T	C	G	A	0	G	0	T	N
G	T	T	T	T	C	N	N	N	N	N	N	N
G	T	T	T	T	C	N	N	N	N	N	N	N
G	T	T	T	T	C	C	T	0	A	0	C	0
G	T	G	T	T	C	G	A	15	A	0	T	1
G	T	G	T	T	T	G	A	0	G	0	C	0
G	T	G	T	T	C	G	A	0	G	0	C	0
G	T	G	T	T	C	C	T	0	A	0	C	0
G	T	T	T	T	N	G	A	0	G	0	T	1
G	T	T	T	T	C	G	A	0	G	N	N	N
G	T	T	T	T	N	G	A	0	G	0	T	1
G	T	G	T	T	N	G	A	0	G	0	T	1
C	C	G	C	G	C	G	A	15	A	0	T	N
N	N	N	N	N	N	G	A	0	G	0	T	1
G	T	G	T	T	C	C	T	0	A	0	C	0
G	T	G	T	T	N	C	T	0	A	0	C	0
G	T	G	T	T	C	G	A	0	G	0	T	1
G	T	G	T	T	C	G	A	0	G	0	T	1
N	N	N	N	N	N	G	A	0	G	0	T	1
G	T	G	T	T	C	G	A	0	G	0	T	1
G	T	G	T	T	T	G	A	0	G	0	T	N
C	C	G	C	G	C	C	T	0	A	0	C	0
G	T	G	T	T	T	G	A	0	G	0	T	1
G	T	G	T	T	C	G	T	0	G	0	T	1
N	N	N	N	N	N	G	A	0	G	0	T	1
N	N	N	N	N	N	G	T	0	G	0	T	1
C	C	G	C	G	C	N	T	0	A	4	C	1
G	T	G	T	T	C	G	A	15	A	0	T	1
N	N	N	N	N	N	G	A	0	G	0	T	1
N	N	N	N	N	N	G	A	0	G	0	T	1

anti8	anti9	anti10	anti11	anti12	anti13	anti14	cp1	cp2	cp3	cp4	cp5	cp6	cp7	cp8	cp9	cp10
G	C	G	G	G	C	G	N	N	8	4	10	12	N	39	N	N
G	C	G	G	G	C	G	N	N	N	N	N	N	N	N	N	N
N	N	N	N	N	N	N	N	N	N	N	N	N	N	N	N	N
G	C	G	G	G	C	C	A	C	8	4	10	12	C	39	G	G
G	C	G	N	N	N	N	N	N	N	N	N	N	N	N	N	N
N	N	N	N	N	N	N	G	C	8	4	0	12	C	39	G	G
G	C	G	G	G	C	C	G	C	0	4	0	12	C	39	A	T
G	C	G	G	G	C	C	G	C	0	4	0	12	C	39	A	T
G	C	G	G	G	C	C	G	C	8	4	0	12	C	39	G	G
G	C	G	G	G	C	C	N	N	N	N	N	N	N	N	N	N
G	C	G	G	G	C	C	A	T	0	4	0	12	T	39	G	G
N	N	N	N	N	N	N	G	C	0	4	0	12	C	39	A	T
A	A	T	G	G	T	G	A	T	8	0	47	0	T	0	G	G
G	C	G	G	G	C	C	N	N	N	N	N	N	N	N	N	N
G	C	G	G	G	C	C	G	C	0	4	0	12	C	39	A	T
G	N	N	N	N	N	N	G	C	0	4	0	12	C	39	A	T
G	C	G	G	G	C	C	G	C	0	4	0	12	C	39	A	T
G	C	G	G	G	C	N	G	C	0	4	0	12	C	39	A	T
G	C	G	G	G	C	C	A	T	0	4	0	12	T	39	G	G
G	C	C	A	C	C	G	G	C	0	4	0	12	C	39	A	T
A	A	T	G	G	T	G	N	N	8	0	47	0	T	0	G	G
N	N	N	N	N	N	N	N	N	N	N	N	N	N	N	N	N
G	C	G	G	G	C	C	G	C	0	4	0	12	C	39	A	T
G	C	C	A	C	C	G	G	C	0	4	0	12	C	39	A	T
A	A	T	G	G	T	G	A	C	8	4	10	12	C	39	G	G
G	C	G	G	G	C	C	G	C	0	4	0	12	C	39	A	T
A	A	T	G	G	T	G	G	C	0	4	0	12	C	39	A	T
G	C	G	G	G	C	C	G	C	0	4	0	12	C	39	A	T
N	N	N	N	N	N	N	G	C	8	4	0	12	C	39	G	G
G	C	G	G	G	C	C	G	C	0	4	0	12	C	39	A	T
N	N	N	N	N	N	N	G	C	8	4	0	12	C	39	G	G
N	N	N	N	N	N	N	G	C	0	4	0	12	C	39	A	T
N	N	N	N	N	N	N	G	C	0	4	0	12	C	39	A	T
A	A	T	G	G	T	G	N	N	N	N	N	N	N	N	N	N
G	C	G	G	G	C	C	G	C	0	4	0	12	C	39	A	T
G	C	C	A	C	C	G	A	T	8	0	47	0	T	0	G	G
N	N	N	N	N	N	N	A	T	8	0	0	12	T	39	G	G
N	N	N	N	N	N	N	A	T	0	4	0	12	T	39	G	G
G	N	N	N	N	N	N	G	C	0	4	0	12	C	39	A	T
G	C	G	G	G	C	C	G	C	0	4	0	12	C	39	A	T
N	N	N	N	N	N	N	G	C	0	4	0	12	C	39	A	T
G	C	G	G	G	C	C	G	C	0	4	0	12	C	39	A	T
G	C	G	G	G	C	G	A	T	8	0	47	0	T	0	G	G
G	C	G	G	G	C	C	G	C	0	4	0	12	C	39	A	T
G	C	C	A	C	C	G	G	C	0	4	0	12	C	39	A	T
N	N	N	N	N	N	N	G	C	0	4	0	12	C	39	A	T
G	C	G	G	G	C	C	G	C	0	4	0	12	C	39	A	T
G	C	G	G	G	C	C	G	C	0	4	0	12	C	39	A	T
G	C	C	A	C	C	G	A	T	8	0	47	0	T	0	G	G
G	C	C	A	C	C	G	A	T	8	0	47	0	T	0	G	G
N	N	N	N	N	N	N	G	C	8	4	0	12	C	39	G	G
G	C	G	G	G	C	C	N	N	N	N	N	N	N	N	N	N
G	C	G	G	G	C	C	A	T	0	4	0	12	T	39	G	G
G	C	G	G	G	C	G	N	N	N	N	N	N	N	N	N	N
G	C	G	G	G	C	C	N	N	N	N	N	N	N	N	N	N
N	N	N	N	N	N	N	G	C	0	4	0	12	C	39	A	T

A	A	T	G	G	T	G	A	T	0	4	0	12	T	39	G	G
N	N	N	N	N	N	N	G	C	0	4	0	12	C	39	A	T
G	C	G	G	G	C	C	N	N	N	N	N	N	N	N	N	N
N	N	N	N	N	N	N	G	C	8	4	0	12	C	39	G	G
G	C	G	G	G	C	C	A	T	8	0	47	0	T	0	G	G
G	C	G	G	G	C	C	A	T	8	0	47	0	T	0	G	G
G	C	G	G	G	C	C	G	C	0	4	0	12	C	39	A	T
G	C	G	G	G	C	C	G	C	0	4	0	12	C	39	A	T
G	C	G	G	G	C	C	G	C	0	4	0	12	C	39	A	T
G	C	G	G	G	C	C	G	C	8	4	0	12	C	39	G	G
G	C	G	G	G	C	C	A	T	0	4	0	12	T	39	G	G
A	A	T	G	G	T	G	G	C	0	4	0	12	C	39	A	T
N	N	N	N	N	N	N	A	C	8	4	0	12	T	39	G	G
G	A	G	G	G	C	G	A	T	8	0	47	0	T	0	G	G
N	N	N	N	N	N	N	G	C	0	4	0	12	C	39	A	T
A	C	T	G	G	T	G	N	N	N	N	N	N	N	N	N	N
N	N	N	N	N	N	N	G	C	0	4	0	12	C	39	A	T
G	C	G	G	G	C	G	G	T	8	0	47	0	T	0	G	G
G	C	G	G	G	C	G	G	C	8	4	0	12	C	39	A	T
A	A	T	G	G	T	G	A	C	8	4	10	12	C	39	G	G
G	A	G	G	G	C	G	N	N	N	N	N	N	N	N	N	N

N	N	N	N	N	N	N	G	C	0	4	0	12	C	39	A	T
A	A	T	G	G	T	G	A	C	8	4	10	12	C	39	G	G
A	A	T	G	G	T	G	A	T	8	0	47	0	T	0	G	G
A	A	N	N	N	N	N	G	C	8	4	0	12	C	39	A	T
G	A	G	G	G	C	G	G	C	0	4	0	12	C	39	A	T
A	A	T	G	G	T	G	A	T	8	0	47	0	T	0	G	G
N	N	N	N	N	N	N	G	C	0	4	0	12	C	39	A	T
G	C	G	G	G	C	C	G	C	0	4	0	12	C	39	A	T
A	A	T	G	G	T	G	G	C	8	4	0	12	C	39	A	T
A	C	T	G	G	T	G	A	C	8	4	0	12	T	39	G	G
A	N	C	A	C	C	G	A	T	8	0	47	0	T	0	G	G
N	N	N	N	N	N	N	A	T	8	0	47	0	T	0	G	G
G	C	G	G	G	C	C	A	T	8	0	47	0	T	0	G	G
G	C	G	G	G	C	C	A	T	8	0	47	0	T	0	G	G
G	C	G	G	G	C	C	A	T	0	4	0	12	T	39	G	G
A	A	T	G	G	T	G	A	C	8	4	10	12	C	39	G	G
A	A	N	N	N	N	N	A	T	8	0	47	0	T	0	G	G
A	A	T	G	G	T	C	A	T	8	0	47	0	T	0	G	G
G	C	G	G	G	C	G	G	C	0	4	0	12	C	39	A	T
N	N	N	N	N	N	N	A	T	8	0	47	0	T	0	G	G
A	N	C	A	C	C	G	A	C	8	4	0	12	T	39	G	G
A	C	T	G	G	T	G	A	C	8	4	0	12	T	39	G	G
N	N	N	N	N	N	N	G	C	0	4	0	12	C	39	A	T
G	C	G	G	G	C	G	A	T	8	0	47	0	T	0	G	G
G	A	G	G	G	C	G	N	N	N	N	N	N	N	N	N	N
A	N	N	N	N	N	N	G	C	0	4	0	12	C	39	A	T
A	C	T	G	G	T	G	A	C	8	4	0	12	T	39	G	G
A	N	C	A	C	C	G	A	C	8	4	10	12	C	39	G	G
N	N	N	N	N	N	N	N	N	N	N	N	N	N	N	N	N
G	C	G	G	G	C	C	G	C	0	4	0	12	C	39	A	T
G	C	G	G	G	C	C	N	N	N	N	N	N	N	N	N	N
G	C	G	G	G	C	C	A	T	0	4	0	12	T	39	G	G
G	C	G	G	G	C	C	A	T	8	0	47	0	T	0	G	G
N	N	N	N	N	N	N	A	T	0	4	0	12	T	39	G	G
N	N	N	N	N	N	N	G	C	0	4	0	12	C	39	A	T

A	C	T	G	G	T	G	N	N	N	N	N	N	N	N	N	N
G	C	C	A	C	C	G	N	N	N	N	N	N	N	N	N	N
N	N	N	N	N	N	N	G	C	0	4	0	12	C	39	A	T
G	C	G	G	G	C	C	G	C	8	4	0	12	C	39	G	G
G	C	C	A	C	C	G	A	T	8	0	47	0	T	0	G	G
G	C	G	G	G	C	C	N	N	N	N	N	N	N	N	N	N
G	C	G	G	G	C	C	G	C	0	4	0	12	C	39	A	T
G	C	G	G	G	C	C	G	C	0	4	0	12	C	39	A	T
G	C	G	G	G	C	C	A	C	8	4	0	12	T	39	G	G
N	N	N	N	N	N	N	A	T	8	0	47	0	T	0	G	G
A	A	T	G	G	T	G	A	C	8	4	10	12	C	39	G	G
G	C	G	G	G	C	N	G	C	0	4	0	12	C	39	A	T
N	N	N	N	N	N	N	A	T	0	4	0	12	T	39	G	G
G	C	G	G	G	C	C	G	C	0	4	0	12	C	39	A	T
G	C	G	G	G	C	C	G	C	0	4	0	12	C	39	A	T
G	C	G	G	G	C	C	A	T	8	0	47	0	T	0	G	G
N	N	N	N	N	N	N	G	C	8	4	0	12	C	39	A	T
N	N	N	N	N	N	N	G	C	0	4	0	12	C	39	A	T
G	C	G	G	G	C	C	A	T	8	0	47	0	T	0	G	G
N	N	N	N	N	N	N	G	C	8	4	0	12	C	39	G	G
G	C	G	G	G	C	C	N	N	N	N	N	N	N	N	N	N
G	C	G	G	G	C	C	G	C	0	4	0	12	C	39	G	G
G	C	G	G	G	C	C	A	T	8	0	47	0	T	0	G	G
A	A	T	G	G	T	G	G	C	0	4	0	12	C	39	A	T
G	C	G	G	G	C	C	G	C	8	4	0	12	C	39	G	G
A	A	T	G	G	T	G	G	C	0	4	0	12	C	39	A	T
G	C	G	G	G	C	C	G	C	0	4	0	12	C	39	A	T
G	C	G	G	G	C	C	A	T	8	0	47	0	T	0	G	G
G	C	G	G	G	C	C	G	C	8	4	0	12	C	39	G	G
G	C	G	G	G	C	N	A	T	8	0	0	12	T	39	G	G
G	C	G	G	G	C	C	A	T	8	0	0	12	T	39	G	G
A	N	C	A	C	C	G	G	C	8	4	0	12	C	39	A	T
G	C	G	G	G	C	G	A	T	8	0	47	0	T	0	G	G
G	C	G	G	G	C	G	A	T	8	0	47	0	T	0	G	G
N	N	N	N	N	N	N	G	C	8	4	0	12	C	39	G	G
A	A	T	G	G	C	G	G	C	0	4	0	12	C	39	A	T
G	C	G	G	G	C	G	G	C	0	4	0	12	C	39	A	T
A	A	T	G	G	T	G	G	C	0	4	0	12	C	39	A	T
N	N	N	N	N	N	N	A	T	8	0	47	0	T	0	G	G
A	A	T	G	G	T	G	G	C	0	4	0	12	C	39	A	T
A	A	T	G	G	T	G	G	C	0	4	0	12	C	39	A	T
A	N	C	A	C	C	G	G	C	8	4	0	12	C	39	G	G
N	N	N	N	N	N	N	N	N	N	N	N	N	N	N	N	N
G	C	G	G	G	C	C	A	T	0	4	0	12	T	39	G	G
G	C	G	G	G	C	C	G	C	8	4	0	12	C	39	A	T
G	C	G	G	G	C	C	A	T	8	0	47	0	T	0	G	G
A	A	T	G	G	T	G	N	N	N	N	N	N	N	N	N	N
G	C	G	G	G	C	C	N	N	N	N	N	N	N	N	N	N
G	C	G	G	G	C	C	G	C	8	4	0	12	C	39	G	G
G	C	G	G	G	C	N	A	T	0	4	0	12	T	39	G	G
G	C	G	G	G	C	C	N	N	N	N	N	N	N	N	N	N
N	N	N	N	N	N	N	G	C	0	4	0	12	C	39	A	T
N	N	N	N	N	N	N	G	C	0	4	0	12	C	39	A	T
G	C	G	G	G	C	C	A	T	8	0	0	12	T	39	G	G
G	C	G	G	G	C	C	A	T	8	0	0	12	T	39	G	G
G	C	G	G	G	C	C	G	C	0	4	0	12	C	39	A	T
G	C	C	A	C	C	G	N	N	N	N	N	N	N	N	N	N

N	N	N	N	N	N	N	A	C	8	4	0	12	T	39	G	G
N	N	N	N	N	N	N	A	T	8	0	47	0	T	0	G	G
N	N	N	N	N	N	N	A	T	8	0	47	0	T	0	G	G
G	C	G	G	G	C	C	N	T	8	0	47	0	T	0	G	G
N	N	N	N	N	N	N	G	C	8	4	0	12	C	39	G	G
G	C	G	G	G	C	C	N	T	8	0	47	0	T	0	G	G
N	N	N	N	N	N	N	G	C	0	4	0	12	C	39	A	T
G	C	G	G	G	C	C	A	T	8	0	0	12	T	39	G	G
N	N	N	N	N	N	N	N	N	0	4	0	12	C	39	A	T
G	C	G	G	G	C	C	G	C	0	4	0	12	C	39	A	T
N	N	N	N	N	N	N	G	C	0	4	0	12	C	39	A	T
N	N	N	N	N	N	N	A	T	0	0	0	12	C	39	A	T
G	C	G	G	G	C	C	N	C	0	4	0	12	C	39	G	G
G	C	G	G	G	C	C	A	T	8	0	47	0	T	0	G	G
G	C	N	N	N	N	N	G	C	0	4	0	12	C	39	A	T
G	C	G	G	G	C	C	A	T	8	0	47	0	T	0	G	G
N	N	N	N	N	N	N	N	N	N	N	N	N	N	N	N	N
G	C	C	A	C	C	G	G	C	0	4	0	12	C	39	A	T
G	C	C	A	C	C	G	N	T	8	0	47	0	T	0	G	G
N	N	N	N	N	N	N	G	C	0	4	0	12	C	39	A	T
G	C	C	A	C	C	G	A	T	8	0	47	0	T	0	G	G
G	C	G	G	G	C	C	A	T	8	0	47	0	T	0	G	G
G	C	C	A	C	C	G	A	T	8	0	47	0	T	0	G	G
N	N	N	N	N	N	N	G	C	0	4	0	12	C	39	A	T
A	C	N	N	N	N	N	A	T	8	0	47	0	T	0	G	G
A	C	T	G	G	T	G	N	N	N	N	N	N	N	N	N	N
A	C	T	G	G	T	G	G	C	0	4	0	12	C	39	A	T
G	C	G	G	G	C	G	A	T	8	0	47	0	T	0	G	G
A	C	T	G	G	T	G	N	T	8	0	47	0	T	0	G	G
N	N	N	N	N	N	N	G	C	0	4	0	12	C	39	A	T
G	C	G	G	G	C	C	A	C	8	4	0	12	T	39	G	G
G	C	G	G	G	C	C	G	C	0	4	0	12	C	39	A	T
G	C	G	G	G	C	C	A	C	8	4	0	12	T	39	G	G
G	C	G	G	G	C	C	A	T	8	0	47	0	T	0	G	G
G	C	G	G	G	C	C	A	T	8	0	47	0	T	0	G	G
G	N	N	N	N	N	N	A	C	8	4	0	12	T	39	G	G
G	C	G	G	G	C	N	G	C	0	4	0	12	C	39	A	T
G	C	G	G	G	C	C	G	C	0	4	0	12	C	39	A	T
G	C	G	G	G	C	G	A	T	8	0	0	12	T	39	G	G
N	N	N	N	N	N	N	A	T	8	0	47	0	T	0	G	G
N	N	N	N	N	N	N	G	C	0	4	0	12	C	39	A	T
G	C	G	G	G	C	G	A	T	8	0	47	0	T	0	G	G
N	N	N	N	N	N	N	A	T	8	0	47	0	T	0	G	G
N	N	N	N	N	N	N	N	N	N	N	N	N	N	N	N	N
A	C	T	G	G	T	G	A	T	8	0	47	0	T	0	G	G
A	C	T	G	G	T	G	G	C	0	4	0	12	C	39	A	T
A	C	T	G	G	T	G	N	C	0	4	0	12	C	39	A	T
A	C	T	G	G	T	G	A	T	8	0	47	0	T	0	G	G
A	C	T	G	G	T	G	G	C	0	4	0	12	C	39	A	T
A	N	C	A	C	C	G	A	T	8	0	47	0	T	0	G	G
G	C	G	G	G	C	G	A	T	8	0	47	0	T	0	G	G
G	C	C	A	C	C	G	A	T	8	0	47	0	T	0	G	G
A	A	T	G	G	T	G	N	N	N	N	N	N	N	N	N	N
A	A	T	G	G	T	G	N	C	8	0	47	0	T	N	G	G
A	A	T	G	G	T	G	A	T	8	0	47	0	T	0	G	G
G	C	G	G	G	C	C	A	C	8	4	0	12	T	39	G	G
N	N	N	N	N	N	N	G	C	8	4	0	12	C	39	G	G

G	C	G	G	G	C	G	G	C	8	4	0	12	C	39	G	G
G	C	N	N	N	N	N	G	C	0	4	0	12	C	39	G	G
G	C	G	G	G	C	C	G	C	0	4	0	12	C	39	A	T
A	A	T	G	G	T	G	G	C	0	4	0	12	C	39	A	T
G	C	G	G	G	C	C	G	C	0	4	0	12	C	39	A	T
G	C	G	G	G	C	G	G	C	0	4	0	12	C	39	A	T
N	N	N	N	N	N	N	G	C	8	4	0	12	C	39	G	G
G	C	G	G	G	C	C	A	T	8	0	0	12	T	39	G	G
G	C	G	G	G	C	C	G	C	8	4	0	12	C	39	A	T
G	C	G	G	G	C	C	N	C	0	4	0	12	C	39	A	T
G	C	G	G	G	C	C	A	T	0	4	0	12	T	39	G	G
G	C	G	G	G	C	C	G	C	0	4	0	12	C	39	A	T
G	C	C	A	C	C	G	G	C	0	4	0	12	C	39	A	T
G	C	C	A	C	C	G	G	C	0	4	0	12	C	39	A	T
G	C	G	G	G	C	C	G	C	0	4	0	12	C	39	A	T
G	C	G	G	G	C	C	G	C	0	4	0	12	C	39	A	T
G	C	G	G	G	C	C	A	C	8	4	0	12	T	39	G	G
A	A	T	G	G	T	G	A	C	8	4	10	12	C	39	G	G
G	C	G	G	G	C	G	A	C	8	4	10	12	C	39	G	G
G	C	G	G	G	C	C	G	C	0	4	0	12	C	39	A	T
G	C	G	G	G	C	C	N	N	N	N	N	N	N	N	N	N
N	N	N	N	N	N	N	A	T	0	4	0	12	T	39	G	G
G	C	G	G	G	C	C	A	T	8	0	47	0	T	0	G	G
G	C	G	G	G	C	C	A	T	8	0	47	0	T	0	G	G
G	C	G	G	G	C	C	G	C	0	4	0	12	C	39	A	T
N	N	N	N	N	N	N	A	T	8	0	47	0	T	0	G	G
N	N	N	N	N	N	N	A	T	8	0	47	0	T	0	G	G
N	N	N	N	N	N	N	A	C	8	4	10	12	C	39	G	G
N	N	N	N	N	N	N	A	C	8	4	10	12	C	39	G	G
A	A	T	G	G	T	G	G	C	0	4	0	12	C	39	A	T
G	C	G	G	G	C	C	A	T	8	0	47	0	T	0	G	G
A	A	T	G	G	T	N	G	C	0	4	0	12	C	39	A	T
A	A	T	G	G	T	N	G	C	0	4	0	12	C	39	A	T
A	A	T	G	G	T	G	G	C	0	4	0	12	C	39	A	T
G	C	G	G	G	C	N	G	C	8	4	0	12	C	39	G	G
N	N	N	N	N	N	N	A	T	8	0	47	0	T	0	G	G
G	C	G	G	G	C	C	N	N	N	N	N	N	N	N	N	N
G	C	G	G	G	C	C	N	T	8	0	47	0	T	0	G	G
N	N	N	N	N	N	N	A	T	8	0	0	12	T	39	G	G
G	C	G	G	G	C	C	A	T	8	0	0	12	T	39	G	G
A	A	T	G	G	T	G	A	T	8	0	0	12	T	39	G	G
A	A	T	G	G	T	G	N	N	N	N	N	N	N	N	N	N
G	C	G	G	G	C	C	A	T	8	0	47	0	T	0	G	G
G	C	G	G	G	C	C	A	T	8	0	47	0	T	0	G	G
G	C	G	G	G	C	C	A	T	8	0	0	12	T	39	G	G
G	C	G	G	G	C	C	A	T	0	0	0	12	C	39	A	T
N	N	N	N	N	N	N	A	T	8	0	47	0	T	0	G	G
A	A	T	G	G	T	G	A	T	8	0	0	12	T	39	G	G
G	C	G	G	G	C	C	N	N	N	N	N	N	N	N	N	N
G	C	C	A	C	C	G	A	T	8	0	47	0	T	0	G	G
G	C	G	G	G	C	C	N	N	N	N	N	N	N	N	N	N
G	C	C	A	C	C	G	N	N	N	N	N	N	N	N	N	N
G	C	G	G	G	C	G	A	T	8	0	0	12	T	39	G	G
G	C	G	G	G	C	C	G	C	8	4	0	12	C	39	G	G
G	C	G	G	G	C	C	A	T	8	0	0	12	T	39	G	G
G	C	G	G	G	C	C	N	N	N	N	N	N	N	N	N	N

cp11	cp12	cp13	cp14	Fe1	Fe2	Fe3	Fe4	Fe5	Fe6	Fe7	Fe8	Fe9	Fe10	Fe11	Fum1	Fum2
N	N	N	N	5	4	2	4	C	G	C	C	C	3	A	N	1
N	N	N	N	5	0	0	4	C	G	C	C	C	3	A	N	N
N	N	N	N	0	4	2	0	G	A	G	G	C	0	G	N	N
C	G	G	C	5	0	0	4	C	G	C	C	C	3	A	T	1
N	N	N	N	0	4	2	0	G	A	G	G	C	0	G	T	1
C	G	G	C	N	N	N	N	N	N	N	N	N	N	N	C	2
C	A	G	C	N	4	2	0	C	G	G	C	C	3	A	C	1
T	G	N	A	5	0	0	4	C	G	C	C	C	3	A	T	1
C	G	G	C	5	0	0	4	C	G	C	C	C	3	A	C	2
N	N	N	N	N	N	N	N	N	N	N	N	N	N	N	N	N
C	A	G	N	0	4	2	0	G	A	G	G	C	0	G	C	2
T	G	A	N	0	4	2	0	G	A	G	G	C	0	G	N	2
C	A	G	C	5	0	0	4	C	G	C	N	N	N	N	C	2
N	N	N	N	5	4	0	4	C	G	C	G	C	0	G	T	1
C	A	N	C	5	0	0	4	C	G	C	C	C	3	A	T	1
C	A	G	C	5	0	0	4	C	G	C	C	C	N	N	T	1
C	A	G	C	5	0	0	4	C	G	C	C	C	3	A	T	1
C	A	N	C	5	0	0	4	C	G	C	N	N	N	N	C	2
C	A	G	C	5	0	0	4	C	G	C	C	C	3	A	C	2
C	A	N	C	5	0	0	4	C	G	C	C	C	3	A	C	2
C	A	G	C	0	4	2	0	G	A	G	C	A	0	G	T	1
N	N	N	N	N	N	N	N	N	N	N	N	N	N	N	T	1
T	G	A	A	0	4	2	0	G	A	G	G	C	0	G	T	1
C	A	A	C	0	4	2	0	G	A	G	C	A	0	G	T	1
C	G	G	C	0	4	2	0	G	A	G	G	C	0	G	C	2
T	G	A	A	0	4	2	0	G	A	G	C	A	0	G	T	1
C	A	A	C	0	4	2	0	G	A	G	C	A	0	G	C	2
C	A	A	C	0	4	2	0	G	A	G	C	A	0	G	T	1
C	G	G	C	0	4	2	0	G	A	G	N	N	N	N	T	1
T	G	G	A	0	4	2	0	G	A	G	G	C	0	G	N	1
C	G	G	C	N	N	N	N	N	N	N	N	N	N	N	T	1
C	A	G	C	5	0	0	4	C	G	N	N	N	N	N	T	1
C	A	A	C	0	4	2	0	G	A	G	G	C	0	G	N	1
N	N	N	N	0	4	2	0	G	A	G	C	A	0	G	T	0
T	G	G	A	5	4	0	4	C	G	C	G	C	0	G	C	2
C	A	G	N	5	0	0	4	C	G	C	C	C	N	N	C	2
C	A	G	C	N	N	N	N	N	N	N	N	N	N	N	T	1
C	A	G	C	0	4	2	0	G	A	N	N	N	N	N	T	1
C	A	G	C	N	N	N	N	N	N	N	N	N	N	N	T	1
C	N	N	C	5	0	0	4	C	G	N	N	N	N	N	T	1
T	G	G	A	N	N	N	N	N	N	N	N	N	N	N	N	1
T	G	G	A	0	4	2	0	G	A	G	G	C	0	G	T	1
C	A	G	C	5	0	0	4	C	G	C	C	C	3	A	T	0
T	G	G	A	5	4	0	4	C	G	C	G	C	0	G	C	2
C	A	N	C	5	0	0	4	C	G	C	C	C	3	A	C	2
C	A	N	C	5	0	0	4	C	G	C	C	C	3	A	N	1
T	G	N	A	5	0	0	4	C	G	C	C	C	3	A	T	1
T	G	N	A	0	4	2	0	G	A	G	C	A	0	G	T	1
C	A	G	C	0	4	2	0	G	A	G	C	A	0	G	T	1
C	A	G	C	5	0	0	4	C	G	C	C	C	3	A	T	1
C	G	G	C	0	4	2	0	G	A	G	C	A	0	G	N	2
N	N	N	N	5	0	0	4	C	G	C	C	C	3	A	N	N
C	A	G	N	N	4	2	0	C	G	G	C	C	3	A	C	2
N	N	N	N	0	4	2	0	G	A	G	G	C	0	G	N	N
N	N	N	N	5	0	0	4	C	G	C	C	C	3	A	N	N
C	A	G	C	N	N	N	N	N	N	N	N	N	N	N	T	1

C	A	G	C	0	4	2	0	G	A	G	C	A	0	G	T	0
T	G	A	A	5	4	0	4	C	G	C	N	N	N	N	N	N
N	N	N	N	5	0	0	4	C	G	C	C	C	3	A	C	2
C	G	N	C	N	4	2	0	C	G	G	C	C	N	N	C	2
C	A	G	C	5	0	0	4	C	G	C	C	C	3	A	C	2
C	A	G	C	5	0	0	4	C	G	C	C	C	3	A	C	2
T	G	G	A	0	4	2	0	G	A	G	N	N	N	N	T	1
C	A	G	C	5	0	0	4	C	G	C	C	C	3	A	T	1
C	A	G	C	5	0	0	4	C	G	C	C	C	3	A	N	1
C	G	G	C	5	0	0	4	C	G	C	C	C	3	A	C	2
C	A	G	C	5	0	0	4	C	G	C	C	C	3	A	T	1
T	G	A	N	5	0	0	4	C	G	N	N	N	N	N	C	2
C	G	G	C	5	0	0	4	C	G	C	C	C	3	A	C	2
C	A	G	C	5	0	0	4	C	G	C	C	C	3	A	C	2
T	G	G	A	N	N	N	N	N	N	N	N	N	N	N	C	2
N	N	N	N	5	0	0	4	C	G	C	C	C	3	A	C	2
T	G	G	C	5	0	0	4	C	G	C	C	C	3	A	T	1
C	A	G	C	N	0	0	4	C	G	C	C	C	3	A	C	2
C	G	G	C	N	0	0	4	C	G	C	G	C	N	N	T	1
C	G	G	C	5	0	0	0	C	G	N	N	N	N	N	T	1
N	N	N	N	5	0	0	4	C	G	C	C	C	3	A	C	2

T	G	G	A	5	0	0	4	C	G	C	C	C	3	A	C	2
C	G	G	C	0	4	2	0	G	A	G	C	A	0	G	T	1
C	N	N	N	N	4	2	0	C	G	G	C	C	3	A	T	1
C	G	G	C	5	0	0	4	C	G	C	C	C	3	A	C	2
T	G	A	A	5	0	0	4	C	G	C	C	C	3	A	C	2
C	A	G	C	5	0	0	4	C	G	C	N	N	N	N	C	2
T	G	A	A	N	4	2	0	C	G	N	N	N	N	N	C	2
T	G	A	A	5	0	0	4	C	G	C	C	C	3	A	C	2
C	G	G	C	5	0	0	4	C	G	C	C	C	3	A	N	N
C	G	G	C	5	0	0	4	C	G	C	C	C	3	A	C	2
C	A	G	C	N	0	0	4	C	G	C	C	C	3	A	T	1
C	A	G	C	N	4	2	0	N	N	N	N	N	N	N	C	2
C	A	G	C	N	0	0	4	C	G	C	G	C	0	G	T	1
C	A	G	C	5	0	0	4	C	G	C	C	C	3	A	C	2
C	A	G	C	5	0	0	4	C	G	C	C	C	3	A	C	2
C	G	G	C	5	0	0	4	C	G	C	C	C	3	A	N	1
C	A	G	C	5	0	0	4	C	G	C	C	C	N	N	C	2
C	A	G	C	5	0	0	4	C	G	C	C	C	3	A	C	2
T	G	N	A	5	0	0	4	C	G	C	C	C	3	A	C	2
C	A	N	N	N	N	N	N	N	N	N	N	N	N	N	C	2
C	G	G	C	5	0	0	4	C	G	C	C	C	3	A	C	2
C	G	G	C	5	0	0	4	C	G	C	C	C	3	A	N	N
T	G	G	A	N	N	N	N	N	N	N	N	N	N	N	C	2
C	A	G	C	5	0	0	4	C	G	C	C	C	3	A	C	N
N	N	N	N	N	4	2	0	C	G	G	C	C	3	A	N	N
T	G	G	A	5	0	0	4	C	G	C	N	N	N	N	C	2
C	G	G	C	N	N	N	N	N	N	N	N	N	N	N	C	2
C	G	G	C	5	0	0	4	C	G	C	C	C	3	A	C	2
N	N	N	N	N	N	N	N	N	N	N	N	N	N	N	C	2
T	G	A	A	5	0	0	4	C	G	C	C	C	3	A	T	1
N	N	N	N	0	4	2	0	G	A	G	C	A	0	G	C	2
C	A	G	C	0	4	2	0	G	A	G	C	A	0	G	T	1
C	A	G	C	5	4	0	4	C	G	C	G	C	0	G	T	1
C	A	G	C	N	N	N	N	N	N	N	N	N	N	N	T	1
C	A	N	C	N	N	N	N	N	N	N	N	N	N	N	T	1

N	N	N	N	0	4	2	0	G	A	G	C	A	0	G	N	N
N	N	N	N	5	4	0	4	C	G	C	G	C	0	G	N	N
C	A	G	C	5	0	0	4	C	G	C	N	N	N	N	T	1
C	G	G	C	0	4	2	0	G	A	G	C	A	0	G	T	1
C	A	G	C	5	4	0	4	C	G	C	G	C	0	G	T	1
N	N	N	N	0	4	2	0	G	A	G	C	A	0	G	T	1
C	A	G	C	5	0	0	4	C	G	C	C	C	0	A	C	1
T	G	A	A	0	4	2	0	G	A	G	C	A	0	G	C	2
C	G	G	C	5	4	0	4	C	G	C	G	C	0	G	T	1
C	A	G	C	N	N	N	N	N	N	N	N	N	N	N	T	1
C	G	G	C	5	0	0	4	C	G	C	C	C	3	A	C	2
C	A	N	C	5	0	0	4	N	N	N	N	N	N	N	T	1
C	A	G	C	N	N	N	N	N	N	N	N	N	N	N	T	1
T	G	N	A	5	4	0	4	C	G	C	G	C	0	G	C	2
C	A	G	C	5	0	0	4	C	G	C	C	C	3	A	T	1
C	A	G	C	5	4	0	4	C	G	C	G	C	0	G	C	2
C	G	G	C	N	N	N	N	N	N	N	N	N	N	N	T	1
C	A	G	C	0	4	2	0	G	N	N	N	N	N	N	C	2
C	A	G	C	0	4	2	0	G	A	G	G	C	0	G	C	2
C	G	G	C	N	N	N	N	N	N	N	N	N	N	N	T	1
N	N	N	N	N	4	2	0	C	G	G	C	C	3	A	T	1
C	G	G	C	0	4	2	0	G	A	G	C	A	0	G	T	1
C	A	G	C	5	4	0	4	C	G	C	G	C	0	G	T	1
T	G	N	A	0	4	2	0	G	A	G	C	A	0	G	T	1
C	G	G	C	0	4	2	0	G	A	G	C	A	0	G	T	1
C	A	G	C	0	4	2	0	G	A	G	C	A	0	G	C	1
C	A	G	C	0	4	2	0	G	A	G	G	C	0	G	C	2
C	A	G	C	0	4	2	0	G	A	G	G	C	0	G	C	2
C	G	G	C	0	4	2	0	G	A	G	C	A	0	G	T	1
C	A	G	C	0	4	2	0	G	A	G	N	N	N	N	T	1
C	A	G	C	0	4	2	0	G	A	G	C	A	0	G	T	1
C	G	G	C	0	4	2	0	G	A	G	C	A	0	G	N	1
C	A	G	C	5	0	0	4	C	G	C	C	C	3	A	T	1
C	A	G	C	5	0	0	4	C	G	C	C	C	3	A	C	2
C	G	G	C	N	4	2	0	C	G	G	C	C	3	A	T	1
T	G	A	A	5	0	0	4	C	G	C	C	C	3	A	T	1
T	G	A	A	5	0	0	4	C	G	C	C	C	3	A	C	2
T	G	G	A	5	0	0	4	C	G	C	C	N	N	N	C	2
C	A	G	C	N	N	N	N	N	N	N	N	N	N	N	C	2
T	G	A	A	5	0	0	4	C	G	C	C	C	3	A	C	2
T	G	A	A	5	0	0	4	C	G	C	C	C	3	A	T	1
C	G	G	C	5	4	0	4	C	G	C	G	C	0	G	N	2
N	N	N	N	N	N	N	N	N	N	N	N	N	N	N	T	1
C	A	G	C	5	0	0	4	C	G	C	C	C	3	A	N	2
C	G	G	C	0	4	2	0	G	A	G	G	C	0	G	C	2
C	A	G	C	5	0	0	4	C	G	C	C	C	3	A	T	1
N	N	N	N	0	4	2	0	G	A	G	G	C	0	G	T	1
N	N	N	N	5	4	0	4	C	G	C	G	C	0	G	T	1
C	G	G	C	5	0	0	4	C	G	C	C	C	N	N	C	2
C	A	G	C	5	0	0	4	C	G	C	C	C	3	A	T	1
N	N	N	N	0	4	2	0	G	A	G	C	A	0	G	N	N
C	A	G	C	N	N	N	N	N	N	N	N	N	N	N	T	1
T	G	G	A	N	N	N	N	N	N	N	N	N	N	N	C	2
C	A	G	C	N	4	2	0	C	G	G	C	C	N	N	C	2
C	A	G	C	5	0	0	4	N	N	N	N	N	N	N	T	1
T	G	G	A	5	0	0	4	C	G	C	N	N	N	N	T	1
N	N	N	N	5	0	0	4	C	G	C	C	C	3	A	T	1

C	G	G	C	N	N	N	N	N	N	N	N	N	N	N	T	1
C	A	G	C	5	0	0	4	C	G	C	C	C	3	A	T	1
C	A	G	C	5	0	0	4	C	G	C	C	C	3	A	T	1
C	A	G	C	5	0	0	4	C	G	C	C	C	3	A	T	1
C	G	G	C	0	4	2	0	G	A	G	G	C	0	G	T	1
C	N	N	N	0	4	2	0	G	A	G	C	A	0	G	T	1
T	G	N	A	N	N	N	N	N	N	N	N	N	N	N	T	1
C	A	G	C	0	4	2	0	G	A	G	C	A	0	G	C	2
T	G	A	A	5	0	0	4	C	G	C	C	C	N	N	T	1
C	A	A	C	5	0	0	4	C	G	C	C	C	3	A	C	2
C	A	N	C	N	N	N	N	N	N	N	N	N	N	N	T	1
C	A	G	C	N	N	N	N	N	N	N	N	N	N	N	C	2
C	G	G	C	5	0	0	4	C	G	C	C	C	3	A	N	1
C	A	G	C	N	N	N	0	C	A	C	C	N	0	G	T	1
C	A	G	C	N	N	N	N	N	N	N	N	N	N	N	C	2
C	A	G	C	0	4	2	0	G	A	G	C	A	0	G	N	1
N	N	N	N	5	0	0	4	C	G	C	C	C	3	A	C	2
T	G	G	A	5	4	0	4	C	G	C	G	C	0	G	N	2
C	A	G	C	0	4	2	0	G	A	G	C	A	0	G	N	1
T	G	G	A	N	N	N	N	N	N	N	N	N	N	N	T	1
C	A	G	C	0	4	2	0	G	A	G	C	A	0	G	T	1
C	A	G	C	0	4	2	0	G	A	G	G	C	0	G	C	2
C	A	G	C	0	4	2	0	G	A	G	C	A	0	G	T	1
T	G	G	A	N	N	N	N	N	N	N	N	N	N	N	C	2
C	A	G	C	5	0	0	4	C	G	C	C	C	N	N	T	1
N	N	N	N	5	0	0	4	C	G	C	C	C	3	A	T	1
T	G	A	A	5	0	0	4	C	G	C	C	C	3	A	C	2
C	A	G	C	5	0	0	4	C	G	C	C	C	3	A	C	2
C	A	G	C	5	0	0	4	C	G	C	C	C	3	A	C	2
T	G	G	C	5	0	0	4	C	G	C	C	C	3	A	C	2
C	G	G	C	0	4	2	0	G	A	N	N	N	N	N	T	1
T	G	N	A	5	4	0	4	C	G	C	G	C	0	G	T	1
C	G	G	C	0	4	2	0	G	A	G	G	C	0	G	C	2
C	A	G	C	0	4	2	0	G	A	G	G	C	0	G	C	2
C	A	G	C	0	4	2	0	G	A	G	G	C	0	G	C	2
C	G	G	C	0	4	2	0	G	A	G	G	C	0	G	C	2
T	G	G	A	0	4	2	0	G	A	N	N	N	N	N	T	1
T	G	G	A	5	4	0	4	C	G	C	G	C	0	G	T	1
C	A	G	C	5	0	0	4	C	G	C	C	C	3	A	N	1
C	A	G	C	N	N	N	N	N	N	N	N	N	N	N	T	1
T	G	A	A	5	0	0	4	C	G	N	N	N	N	N	T	1
C	A	G	C	5	0	0	4	C	G	C	C	C	3	A	C	2
C	A	G	C	N	N	N	N	N	N	N	N	N	N	N	T	1
N	N	N	N	N	N	N	N	N	N	N	N	N	N	N	T	1
C	A	G	C	5	0	0	4	C	G	C	C	C	3	A	T	1
T	G	N	A	0	4	2	0	G	A	G	G	C	0	G	C	2
T	G	G	A	N	N	N	N	N	N	N	N	N	N	N	N	1
C	A	G	C	5	0	0	4	C	G	C	C	C	3	A	T	1
T	G	G	A	5	0	0	4	C	G	C	N	N	N	N	T	1
C	A	G	C	5	0	0	4	C	G	C	C	C	3	A	T	1
C	A	G	C	5	0	0	4	C	G	C	C	C	3	A	T	1
C	A	G	C	5	0	0	4	C	G	C	C	C	3	A	T	1
N	N	N	N	N	N	N	N	N	N	N	N	N	N	N	T	1
C	A	G	C	5	0	0	4	C	G	C	C	C	N	N	T	1
C	A	G	C	5	0	0	4	C	G	C	C	C	3	A	T	1
C	G	G	C	5	4	0	4	C	G	C	G	C	0	G	T	1
C	G	G	C	5	0	0	4	C	G	C	C	C	3	A	T	1

C	G	G	C	5	4	0	4	C	G	C	G	C	0	G	T	1
C	G	G	C	5	4	0	4	C	G	N	N	N	N	N	T	1
C	A	G	C	5	4	0	4	C	G	C	G	C	0	G	T	1
C	A	G	C	0	4	2	0	G	A	G	G	C	0	G	T	1
C	A	N	C	0	4	2	0	G	A	G	G	C	0	G	C	2
C	A	G	C	0	4	2	0	G	A	G	C	A	0	G	T	1
C	G	G	C	0	4	2	0	G	A	G	C	A	N	N	T	1
C	A	G	C	5	4	0	4	C	G	C	G	C	0	G	T	1
C	G	G	C	0	4	2	0	G	A	G	C	A	0	G	C	2
T	G	G	A	0	4	2	0	G	A	G	C	A	0	G	C	2
C	A	G	C	5	4	0	4	C	G	C	G	C	0	G	T	1
C	A	G	C	5	0	0	4	C	G	C	C	C	3	A	N	1
T	G	G	A	0	4	2	0	G	A	G	C	A	0	G	T	0
C	A	G	C	0	4	2	0	G	A	G	C	A	0	G	T	1
T	G	A	A	0	4	2	0	G	A	G	G	C	0	G	C	2
T	G	G	A	0	4	2	0	G	A	G	C	A	0	G	T	1
C	G	G	C	0	4	2	0	G	A	G	G	C	N	N	T	1
C	G	G	C	5	0	0	4	C	G	C	C	C	3	A	C	2
C	G	G	C	N	0	0	4	C	G	C	G	C	0	G	T	1
C	A	G	C	0	4	2	0	G	A	G	C	A	0	G	N	1
N	N	N	N	5	0	0	4	C	G	C	C	C	3	A	C	2
C	A	G	C	N	N	N	N	N	N	N	N	N	N	N	T	1
C	A	G	C	0	4	2	0	G	A	G	G	C	0	G	N	1
C	A	G	C	0	4	2	0	G	A	G	G	C	0	G	T	1
C	A	A	C	5	0	0	4	C	G	C	C	C	3	A	C	2
C	A	G	C	N	N	N	N	N	N	N	N	N	N	N	T	1
C	A	G	C	0	4	2	0	G	A	G	G	C	N	N	T	1
C	G	G	C	N	N	N	N	N	N	N	N	N	N	N	T	1
C	G	G	C	N	N	N	N	N	N	N	N	N	N	N	C	2
C	A	G	C	N	N	N	N	N	N	N	N	N	N	N	T	1
C	A	G	C	N	0	0	4	C	G	C	G	C	0	G	C	2
T	G	N	A	N	0	0	4	C	G	C	N	N	N	N	C	2
C	A	G	C	5	0	0	4	C	G	C	C	C	3	A	C	2
C	A	A	C	5	0	0	4	C	G	C	C	C	3	A	C	2
C	G	G	C	5	0	0	4	C	G	C	C	C	3	A	C	2
C	A	G	C	N	N	N	N	N	N	N	N	N	N	N	C	2
N	N	N	N	5	0	0	4	C	G	C	C	C	3	A	T	1
C	A	G	C	0	4	2	0	G	A	G	G	C	0	G	T	1
C	A	G	C	N	N	N	N	N	N	N	N	N	N	N	T	1
C	A	G	C	0	4	2	0	G	A	G	G	C	0	G	T	1
C	A	G	C	0	4	2	0	G	A	G	G	C	0	G	T	1
N	N	N	N	5	4	0	4	C	G	C	G	C	0	G	T	1
C	A	G	C	0	4	2	0	G	A	G	G	C	0	G	T	1
C	A	G	C	0	4	2	0	G	A	G	G	C	0	G	T	1
C	A	G	C	0	4	2	0	G	A	G	C	A	0	G	T	1
C	A	G	C	0	4	2	0	G	A	G	G	C	0	G	T	1
C	A	G	C	5	4	0	4	C	G	C	G	C	0	G	T	1
C	A	G	C	0	4	2	0	G	A	G	G	C	0	G	C	2
N	N	N	N	0	4	2	0	G	A	G	C	A	0	G	T	1
C	A	G	C	0	4	2	0	G	A	G	G	C	0	G	T	1
N	N	N	N	5	0	0	4	C	G	C	C	C	3	A	C	2
N	N	N	N	5	0	0	4	C	G	C	C	C	N	N	N	N
C	A	G	C	0	4	2	0	G	A	G	C	A	0	G	T	1
C	G	G	C	0	4	2	0	G	A	G	C	A	0	G	T	1
C	A	G	C	0	4	2	0	G	A	G	C	A	N	N	T	1
N	N	N	N	N	N	N	N	N	N	N	N	N	N	N	N	N

Fum3	Fum4	Fum5	Fum6	Fum7	Fum8	Ger1	Ger2	Ger3	Ger4	Ger5	Ger6	Ger7	Ger8	Ger9
N	N	N	N	1	1	N	N	N	N	N	N	N	N	N
N	N	N	N	N	N	N	N	N	N	N	N	N	N	N
N	N	N	N	N	N	N	N	N	N	N	N	N	N	N
A	T	A	G	0	0	C	A	C	G	T	G	C	C	A
A	T	A	G	0	0	N	N	N	N	N	N	N	N	N
T	C	T	A	1	1	N	N	N	N	N	N	N	N	N
A	T	T	A	0	1	C	A	C	G	T	G	C	C	A
A	T	A	G	0	0	C	A	C	G	T	G	C	C	A
A	C	T	A	1	1	T	C	T	G	C	A	T	T	C
N	N	N	N	N	N	N	N	N	N	N	N	N	N	N
A	C	T	A	1	1	C	A	C	G	T	G	C	C	A
T	C	T	A	1	1	C	A	C	G	T	G	C	C	A
A	C	T	A	0	0	N	N	N	N	N	N	N	N	N
A	T	A	G	0	0	N	N	N	N	N	N	N	N	N
A	T	A	G	0	0	C	C	C	A	C	A	C	C	C
A	T	A	G	0	0	N	N	N	N	N	N	N	N	N
A	T	A	G	0	0	C	C	C	A	C	A	C	C	C
A	C	T	A	1	1	N	N	N	N	N	N	N	N	N
A	C	T	A	1	1	C	A	C	G	T	G	C	C	A
A	C	T	A	1	1	C	A	C	G	T	G	C	C	A
A	T	A	G	0	0	N	N	N	N	N	N	N	N	N
A	T	A	G	0	0	C	A	C	G	T	G	C	C	A
A	T	A	G	0	0	C	C	C	A	C	A	C	C	C
A	T	A	G	0	0	C	C	C	A	C	A	C	C	C
A	C	T	A	0	0	T	C	T	G	C	A	T	T	C
A	T	A	G	0	0	C	C	C	A	C	A	C	C	C
A	C	N	N	N	N	C	C	C	A	C	A	C	C	C
A	T	A	G	0	0	C	C	C	A	C	A	C	C	C
A	T	A	G	0	0	C	A	C	G	T	G	C	C	A
A	T	A	G	0	0	C	C	C	A	C	A	C	C	C
A	T	A	G	0	0	N	N	N	N	N	N	N	N	N
A	T	A	G	0	0	N	N	N	N	N	N	N	N	N
A	T	A	G	0	0	C	A	C	G	T	G	C	C	A
A	T	A	G	0	0	N	N	N	N	N	N	N	N	N
A	C	T	A	1	1	C	A	C	G	T	G	C	C	A
T	C	T	A	1	1	C	A	C	G	T	G	C	C	A
A	T	A	G	0	0	C	A	C	G	T	G	C	C	A
A	T	T	G	0	0	N	N	N	N	N	N	N	N	N
A	T	A	G	0	0	C	A	C	G	T	G	C	C	A
A	T	A	G	0	0	C	C	C	A	C	A	C	C	C
A	T	A	G	0	0	C	C	C	A	C	A	C	C	C
A	T	A	G	0	0	C	C	C	A	C	A	C	C	C
A	T	A	G	0	0	C	A	C	G	T	G	C	C	A
A	C	T	A	1	1	C	A	C	G	T	G	C	C	A
T	C	T	A	1	1	C	C	C	A	C	A	C	C	C
A	T	A	G	0	0	N	C	C	A	C	A	C	C	C
A	T	A	G	0	0	C	C	C	A	C	A	C	C	C
A	T	A	G	0	0	N	N	N	N	N	N	N	N	N
A	T	A	G	0	0	C	C	C	A	C	A	C	C	C
A	T	A	G	0	0	C	A	C	G	T	G	C	C	A
A	C	T	A	1	1	N	N	N	N	N	N	N	N	N
N	N	N	N	N	N	N	N	N	N	N	N	N	N	N
A	C	T	A	1	1	C	C	C	G	T	A	C	C	C
N	N	N	N	N	N	N	N	N	N	N	N	N	N	N
N	N	N	N	N	N	N	N	N	N	N	N	N	N	N
A	T	A	G	0	0	N	N	N	N	N	N	N	N	N

A	T	T	G	0	0	C	A	C	G	T	G	C	C	A
A	T	T	G	0	0	T	C	T	G	C	A	T	T	C
A	C	T	A	1	1	N	N	N	N	N	N	N	N	N
T	C	T	A	1	1	C	C	C	A	C	A	C	C	C
T	C	T	A	1	1	C	C	C	A	C	A	C	C	C
T	C	T	A	1	1	T	C	T	G	C	A	T	T	C
A	T	A	G	0	0	N	N	N	N	N	N	N	N	N
A	T	A	G	0	0	N	N	N	N	N	N	N	N	N
A	T	A	G	0	0	N	N	N	N	N	N	N	N	N
A	C	T	A	1	1	N	N	N	N	N	N	N	N	N
A	T	A	G	0	0	C	C	C	A	C	A	C	C	C
T	C	T	A	1	1	C	C	C	A	C	A	C	C	C
T	C	T	A	1	1	N	N	N	N	N	N	N	N	N
T	C	T	A	1	1	C	C	C	A	C	A	C	C	C
T	C	T	A	1	1	C	C	C	A	C	A	C	C	C
T	C	T	A	1	1	C	C	C	A	C	A	C	C	C
A	T	A	G	0	0	C	C	C	A	C	A	C	C	C
A	C	T	A	1	1	C	C	C	A	C	A	C	C	C
A	C	T	A	1	1	C	A	C	G	T	G	C	C	A
A	T	A	G	0	0	C	C	C	A	C	A	C	C	C
T	C	T	A	1	1	C	A	C	G	T	G	C	C	A

T	C	T	A	1	1	C	A	C	G	T	G	C	C	A
A	T	A	G	0	0	C	A	C	G	T	G	C	C	A
A	T	A	G	0	0	C	C	C	A	C	A	C	C	C
A	C	T	A	1	1	C	C	C	A	C	A	C	C	C
T	C	T	A	1	1	C	C	C	A	C	A	C	C	C
A	C	T	A	1	1	T	C	T	G	C	A	T	T	C
A	C	T	A	1	1	C	A	C	G	T	G	C	C	A
A	C	T	A	0	0	C	C	C	A	C	A	C	C	C
A	N	N	N	N	N	N	N	N	N	N	N	N	N	N
T	C	T	A	1	1	T	C	T	G	C	A	T	T	C
A	T	A	G	0	0	C	C	C	A	C	A	C	C	C
T	C	T	A	1	1	C	C	C	A	C	A	C	C	C
A	T	A	G	0	0	C	C	C	A	C	A	C	C	C
A	C	T	A	0	0	T	C	T	G	C	A	T	T	C
T	C	T	A	1	1	N	N	N	N	N	N	N	N	N
A	T	A	G	0	0	C	C	C	A	C	A	C	C	C
T	C	T	A	1	1	T	C	T	G	C	A	T	T	C
A	C	T	A	0	0	T	C	T	G	C	A	T	T	C
T	C	T	A	1	1	C	A	C	G	T	G	C	C	A
A	C	T	A	1	1	C	C	C	A	C	A	C	C	C
T	C	T	A	1	1	C	C	C	A	C	A	C	C	C
N	N	N	N	N	N	C	C	C	A	C	A	C	C	C
T	C	T	A	1	1	C	C	C	A	C	A	C	C	C
N	N	N	N	N	N	C	A	C	G	T	G	C	C	A
N	N	N	N	N	N	C	C	C	A	C	A	C	C	C
T	C	T	A	1	1	N	N	N	N	N	N	N	N	N
T	C	T	A	1	1	C	C	C	A	C	A	C	C	C
T	C	T	A	1	1	T	C	T	G	C	A	T	T	C
T	C	T	A	1	1	C	A	C	G	T	G	C	C	A
A	T	A	G	0	0	N	N	N	N	N	N	N	N	N
A	C	T	A	1	1	N	N	N	N	N	N	N	N	N
A	T	A	G	0	0	C	A	C	G	T	G	C	C	A
A	T	A	G	0	0	N	N	N	N	N	N	N	N	N
A	T	A	G	0	0	C	A	C	G	T	G	C	C	A
A	T	A	G	0	0	N	N	N	N	N	N	N	N	N

N	N	N	N	N	N	N	N	N	N	N	N	N	N	N
N	N	N	N	N	N	N	N	N	N	N	N	N	N	N
A	T	A	G	0	0	C	C	C	A	C	A	C	C	C
A	T	A	G	0	0	N	N	N	N	N	N	N	N	N
A	T	T	G	0	0	N	N	N	N	N	N	N	N	N
A	T	A	G	0	0	C	A	C	G	T	G	C	C	A
A	T	A	G	0	0	C	A	C	G	T	G	C	C	A
T	C	T	A	1	1	C	A	C	G	T	G	C	C	A
A	T	A	G	0	0	N	N	N	N	N	N	N	N	N
A	T	A	G	0	0	T	C	T	G	C	A	T	T	C
A	C	T	A	0	0	T	C	T	G	C	A	T	T	C
A	T	A	G	0	0	N	N	N	N	N	N	N	N	N
A	C	T	A	1	1	N	N	N	N	N	N	N	N	N
A	C	T	A	1	1	N	N	N	N	N	N	N	N	N
A	T	A	G	0	0	C	C	C	A	C	A	C	C	C
T	C	T	A	1	1	T	C	T	G	C	A	T	T	C
A	T	A	G	0	0	C	A	C	G	T	G	C	C	A
T	C	T	A	1	1	C	C	C	A	C	A	C	C	C
T	C	T	A	1	1	N	N	N	N	N	N	N	N	N
A	C	T	A	1	1	N	N	N	N	N	N	N	N	N
A	T	A	G	0	0	N	N	N	N	N	N	N	N	N
A	T	A	G	0	0	N	N	N	N	N	N	N	N	N
A	T	T	G	0	0	N	N	N	N	N	N	N	N	N
A	T	A	G	0	0	N	N	N	N	N	N	N	N	N
A	T	A	G	0	0	N	N	N	N	N	N	N	N	N
A	T	T	A	0	1	N	N	N	N	N	N	N	N	N
T	C	T	A	1	1	N	N	N	N	N	N	N	N	N
T	C	T	A	1	1	N	N	N	N	N	N	N	N	N
A	T	A	G	0	0	N	N	N	N	N	N	N	N	N
A	T	A	G	0	0	N	N	N	N	N	N	N	N	N
A	T	A	G	0	0	C	C	C	A	C	A	C	C	C
A	T	A	G	0	0	N	N	N	N	N	N	N	N	N
A	C	T	A	1	1	C	C	C	A	C	A	C	C	C
T	C	T	A	1	1	C	C	C	A	C	A	C	C	C
A	T	A	G	0	0	C	C	C	A	C	A	C	C	C
A	T	A	G	0	0	C	A	C	G	T	G	C	C	A
T	C	T	A	1	1	C	A	C	G	T	G	C	C	A
T	C	T	A	1	1	C	A	C	G	T	G	C	C	A
T	C	T	A	1	1	C	C	C	A	C	A	C	C	C
T	C	T	A	1	1	C	A	C	G	T	G	C	C	A
A	T	A	G	0	0	T	C	T	G	C	A	T	T	C
T	C	T	A	1	1	T	C	T	G	C	A	T	T	C
A	T	A	G	0	0	C	C	C	A	C	A	C	C	C
T	C	T	A	1	1	N	N	N	N	N	N	N	N	N
T	C	T	A	1	1	C	C	C	A	C	A	C	C	C
A	T	A	G	0	0	N	N	N	N	N	N	N	N	N
A	C	T	A	1	1	C	C	C	A	C	A	C	C	C
A	T	A	G	0	0	N	N	N	N	N	N	N	N	N
T	C	T	A	1	1	T	C	T	G	C	A	T	T	C
A	T	A	G	0	0	N	N	N	N	N	N	N	N	N
N	N	N	N	N	N	N	N	N	N	N	N	N	N	N
A	T	A	G	0	0	N	N	N	N	C	A	C	C	C
A	C	T	A	1	1	N	N	N	N	N	N	N	N	N
A	C	T	A	1	1	N	N	N	N	N	N	N	N	N
A	T	A	G	0	0	C	C	C	A	C	A	C	C	C
A	T	A	G	0	0	N	N	N	N	N	N	N	N	A
A	T	A	G	0	0	C	C	C	A	C	A	C	C	C

A	T	A	G	0	0	C	C	C	A	C	A	C	C	C
A	T	T	G	0	0	C	C	C	A	C	A	C	C	C
A	T	A	G	0	0	C	C	C	A	C	A	C	C	C
A	T	A	G	0	0	C	C	C	A	C	A	C	C	C
A	T	A	G	0	0	C	A	C	G	T	G	C	C	A
A	T	A	G	0	0	N	N	N	N	N	N	N	N	N
A	T	A	G	0	0	C	A	C	G	T	G	C	C	A
A	C	T	A	1	1	C	C	C	A	C	A	C	C	C
A	T	A	G	0	0	C	C	C	A	C	A	C	C	C
T	C	T	A	1	1	C	C	C	A	C	A	C	C	C
A	C	T	G	1	1	N	N	N	N	N	N	N	N	N
A	C	T	A	1	1	C	C	C	A	C	A	C	C	C
A	T	A	G	0	0	N	N	N	N	N	N	N	N	N
A	T	T	G	0	0	C	C	C	A	C	A	C	C	C
A	C	T	A	1	1	C	C	C	A	C	A	C	C	C
A	T	A	G	0	0	C	C	C	A	C	A	C	C	C
A	C	T	A	0	0	T	C	T	G	C	A	T	T	C
A	C	T	A	1	1	C	A	C	G	T	G	C	C	A
A	T	A	G	0	0	N	N	N	N	N	N	N	N	N
A	T	A	G	0	0	C	C	C	A	C	A	C	C	C
A	T	A	G	0	0	C	C	C	A	C	A	C	C	C
T	C	T	A	1	1	C	C	C	A	C	A	C	C	C
A	T	A	G	0	0	C	C	C	A	C	A	C	C	C
A	C	T	A	1	1	C	A	C	G	T	G	C	C	A
A	T	A	G	0	0	C	A	C	G	T	G	C	C	A
A	T	A	G	0	0	N	N	N	N	N	N	N	N	N
T	C	T	A	1	1	C	A	C	G	T	G	C	C	A
A	C	T	A	0	0	C	A	C	G	T	G	C	C	A
T	C	T	A	1	1	N	N	N	N	N	N	N	N	N
T	C	T	A	1	1	C	A	C	G	T	G	C	C	A
A	T	A	G	0	0	C	A	C	G	T	G	C	C	A
A	T	A	G	0	0	C	A	C	G	T	G	C	C	A
A	C	T	A	1	1	C	C	C	A	C	A	C	C	C
A	C	T	A	0	0	N	N	N	N	N	N	N	N	N
T	C	T	A	1	1	C	C	C	A	C	A	C	C	C
A	C	T	A	1	1	C	A	C	G	T	G	C	C	A
A	T	A	G	0	0	C	C	C	A	C	A	C	C	C
A	T	A	G	0	0	C	A	C	G	T	G	C	C	A
A	T	A	G	0	0	T	C	T	G	C	A	T	T	C
A	T	A	G	0	0	C	A	C	G	T	G	C	C	A
A	T	A	G	0	0	C	A	C	G	T	G	C	C	A
A	C	T	A	0	0	T	C	T	G	C	A	T	T	C
A	T	A	G	0	0	N	N	N	N	N	N	N	N	N
A	T	A	G	0	0	N	N	N	N	N	N	N	N	N
A	T	A	G	0	0	C	A	C	G	T	G	C	C	A
T	C	T	A	1	1	C	A	C	G	T	G	C	C	A
A	T	A	G	0	0	N	N	N	N	N	N	N	N	N
A	T	A	G	0	0	C	A	C	G	T	G	C	C	A
A	T	A	G	0	0	C	A	C	G	T	G	C	C	A
A	T	A	G	0	0	C	A	C	G	T	G	C	C	A
A	T	A	G	0	0	C	C	C	A	C	A	C	C	C
A	T	A	G	0	0	C	C	C	A	C	A	C	C	C
A	T	A	G	0	0	C	C	C	A	C	A	C	C	C
A	T	A	G	0	0	N	N	N	N	N	N	N	N	N
A	T	A	G	0	0	C	A	C	G	T	G	C	C	A
A	T	A	G	0	0	C	A	C	G	T	G	C	C	A
A	T	A	G	0	0	N	N	N	N	N	N	N	N	N

A	T	A	G	0	0	N	N	N	N	N	N	N	N	N
A	T	A	G	0	0	N	N	N	N	N	N	N	N	N
A	T	A	G	0	0	N	N	N	N	N	N	N	N	N
A	T	A	G	0	0	C	C	C	A	C	A	C	C	C
A	C	T	A	1	1	C	C	C	A	C	A	C	C	C
A	T	A	G	0	0	N	N	N	N	N	N	N	N	N
A	T	A	G	0	0	N	N	N	N	N	N	N	N	N
A	T	A	G	0	0	N	N	N	N	N	N	N	N	N
T	C	T	A	1	1	N	N	N	N	N	N	N	N	N
T	C	T	A	1	1	N	N	N	N	N	N	N	N	N
A	C	T	A	1	1	N	N	N	N	N	N	N	N	N
A	T	A	G	0	0	C	C	C	A	C	A	C	C	C
A	T	A	G	0	0	N	N	N	N	N	N	N	N	N
A	T	A	G	0	0	C	C	C	A	C	A	C	C	C
A	C	T	A	1	1	C	C	C	A	C	A	C	C	C
A	T	A	G	0	0	C	A	C	G	T	G	C	C	A
A	T	A	G	0	0	N	N	N	N	N	N	N	N	N
A	C	T	A	0	0	T	C	T	G	C	A	T	T	C
A	T	A	G	0	0	C	C	C	A	C	A	C	C	C
A	T	A	G	0	0	C	C	C	A	C	A	C	C	C
A	C	N	N	N	N	T	C	C	A	C	A	C	C	C
A	T	A	G	0	0	N	N	N	N	N	N	N	N	N
A	T	A	G	0	0	N	N	N	N	N	N	N	N	N
A	T	A	G	0	0	N	N	N	N	N	N	N	N	N
A	C	T	A	1	1	C	C	C	A	C	A	C	C	C
A	T	T	G	0	0	N	N	N	N	N	N	N	N	N
A	T	A	G	0	0	N	N	N	N	N	N	N	N	N
A	T	A	G	0	0	C	C	C	A	C	A	C	C	C
T	C	T	A	1	1	T	C	T	G	C	A	T	T	C
A	T	A	G	0	0	C	C	C	A	C	A	C	C	C
T	C	T	A	1	1	C	A	C	G	T	G	C	C	A
T	C	T	A	1	1	C	C	C	A	C	A	C	C	C
A	C	T	A	0	0	C	C	C	A	C	A	C	C	C
T	C	T	A	1	1	C	C	C	A	C	A	C	C	C
T	C	T	A	1	1	C	A	C	G	T	G	C	C	A
T	C	T	A	1	1	T	C	T	G	C	A	T	T	C
A	T	A	G	0	0	T	C	T	G	C	A	T	T	C
A	T	A	G	0	0	N	N	N	N	N	N	N	N	N
A	T	A	G	0	0	N	N	N	N	N	N	N	N	N
A	T	A	G	0	0	N	N	N	N	N	N	N	N	N
A	T	A	G	0	0	C	A	C	G	T	G	C	C	A
A	T	A	G	0	0	N	N	N	N	N	N	N	N	N
A	T	A	G	0	0	N	N	N	N	N	N	N	N	N
A	T	A	G	0	0	C	C	C	A	C	A	C	C	C
A	T	A	G	0	0	N	N	N	N	N	N	N	N	N
A	C	T	G	1	1	N	N	N	N	N	N	N	N	N
A	T	A	G	0	0	N	N	N	N	N	N	N	N	N
T	C	T	A	1	1	C	C	C	A	C	A	C	C	C
A	T	A	G	0	0	C	A	C	G	T	G	C	C	A
A	T	A	G	0	0	C	C	C	A	C	A	C	C	C
T	C	T	A	1	1	C	A	C	G	T	G	C	C	A
N	N	N	N	N	N	N	N	N	N	N	N	N	N	N
A	T	A	G	0	0	N	N	N	N	N	N	N	N	N
A	T	A	G	0	0	C	C	C	A	C	A	C	C	C
A	C	T	A	1	1	N	N	N	N	N	N	N	N	N
N	N	N	N	N	N	N	N	N	N	N	N	N	N	N

Ger10	Ger11	Ger12	Ger13	Ger14	Ger15	Ger16	Ger17	Ger18	Ger19	Ger20	Ger21	Ger22
N	N	N	N	N	N	N	N	N	N	N	N	N
N	N	N	N	N	N	N	N	N	N	N	N	N
N	N	N	N	N	N	N	N	N	N	N	N	N
T	C	C	A	C	C	C	C	A	T	A	G	T
N	N	N	N	N	N	N	N	N	N	N	N	N
N	N	N	N	N	N	N	N	N	N	N	N	N
T	C	C	A	C	C	C	C	A	T	A	G	T
T	C	C	A	C	C	C	C	A	T	A	G	T
C	T	A	T	G	T	C	C	C	C	G	T	C
N	N	N	N	N	N	N	N	N	N	N	N	N
T	C	C	A	C	C	C	C	A	T	A	G	T
T	C	C	A	C	C	C	C	A	T	A	G	T
N	N	N	N	N	N	N	N	N	N	N	N	N
N	N	N	N	N	N	N	N	N	N	N	N	N
T	C	C	A	C	C	T	T	A	T	G	G	T
N	N	N	N	N	N	N	N	N	N	N	N	N
T	C	C	A	C	C	T	T	A	T	G	G	T
N	N	N	N	N	N	N	N	N	N	N	N	N
T	C	C	A	C	C	C	C	A	T	A	G	T
T	C	C	A	C	C	C	C	A	T	A	G	T
N	N	N	N	N	N	N	N	N	N	N	N	N
T	C	C	A	C	C	C	C	A	T	A	G	T
T	C	C	A	C	C	T	T	A	T	G	G	T
T	C	C	A	C	C	T	T	A	T	G	G	T
C	T	A	T	G	T	C	C	C	C	G	T	C
T	C	C	A	C	C	T	T	A	T	G	G	T
T	C	C	A	C	C	T	T	A	T	G	G	T
T	C	C	A	C	C	T	T	A	T	G	G	T
T	C	C	A	C	C	C	C	A	T	A	G	T
T	C	C	A	C	C	T	T	A	T	G	G	T
N	N	N	N	N	N	N	N	N	N	N	N	N
N	N	N	N	N	N	N	N	N	N	N	N	N
T	C	C	A	C	C	C	C	A	T	A	G	T
N	N	N	N	N	N	N	N	N	N	N	N	N
T	C	C	A	C	C	C	C	A	T	A	G	T
T	C	C	A	C	C	C	C	A	T	A	G	T
T	C	C	A	C	C	C	C	N	N	N	N	N
N	N	N	N	N	N	N	N	N	N	N	N	N
T	C	C	A	C	C	C	C	A	T	A	G	T
T	C	C	A	C	C	T	T	A	T	G	G	T
T	C	C	A	C	C	T	T	A	T	G	G	T
T	C	C	A	C	C	T	T	A	T	G	G	T
T	C	C	A	C	C	C	C	A	T	A	G	T
T	C	C	A	C	C	C	C	A	T	A	G	T
T	C	C	A	C	C	T	T	A	T	G	G	T
T	C	C	A	C	C	T	T	A	T	G	G	T
T	C	C	A	C	C	T	T	A	T	G	G	T
N	N	N	N	N	N	N	N	N	N	N	N	N
T	C	C	A	C	C	T	T	A	T	G	G	T
T	C	C	A	C	C	C	C	A	T	A	G	T
N	N	N	N	N	N	N	N	N	N	N	N	N
N	N	N	N	N	N	N	N	N	N	N	N	N
T	C	C	A	C	C	C	T	A	N	N	G	T
N	N	N	N	N	N	N	N	N	N	N	N	N
N	N	N	N	N	N	N	N	N	N	N	N	N
N	N	N	N	N	N	N	N	N	N	N	N	N

T	C	C	A	C	C	C	C	A	T	A	G	T
C	T	A	T	G	T	C	C	C	C	G	T	C
N	N	N	N	N	N	N	N	N	N	N	N	N
T	C	C	A	C	C	T	T	A	T	G	G	T
T	C	C	A	C	C	T	T	A	T	G	G	T
C	T	A	T	G	T	C	C	C	C	G	T	C
N	N	N	N	N	N	N	N	N	N	N	N	N
N	N	N	N	N	N	N	N	N	N	N	N	N
N	N	N	N	N	N	N	N	N	N	N	N	N
N	N	N	N	N	N	N	N	N	N	N	N	N
T	C	C	A	C	C	T	T	A	T	G	G	T
T	C	C	A	C	C	T	T	A	T	G	G	T
N	N	N	N	N	N	N	N	N	N	N	N	N
T	C	C	A	C	C	T	T	A	T	G	G	T
T	C	C	A	C	C	T	T	A	T	G	G	T
T	C	C	A	C	C	T	T	A	T	G	G	T
T	C	C	A	C	C	T	T	A	T	G	G	T
T	C	C	A	C	C	T	T	A	T	G	G	T
T	C	C	A	C	C	C	C	A	T	A	G	T
T	C	C	A	C	C	T	T	A	T	G	G	T
T	C	C	A	C	C	C	C	A	T	A	G	T

T	C	C	A	C	C	C	C	A	T	A	G	T
T	C	C	A	C	C	C	C	A	T	A	G	T
T	C	C	A	C	C	T	T	A	T	G	G	T
T	C	C	A	C	C	T	T	A	T	G	G	T
T	C	C	A	C	C	T	T	A	T	G	G	T
C	T	A	T	G	T	C	C	C	C	G	T	C
T	C	C	A	C	C	C	C	A	T	A	G	T
T	C	C	A	C	C	T	T	A	T	G	G	T
N	N	N	N	N	N	N	N	N	N	N	N	N
C	T	A	T	G	T	C	C	C	C	G	T	C
T	C	C	A	C	C	T	T	A	T	G	G	T
T	C	C	A	C	C	T	T	A	T	G	G	T
T	C	C	A	C	C	T	T	A	T	G	G	T
C	T	A	T	G	T	C	C	C	C	G	T	C
N	N	N	N	N	N	N	N	N	N	N	N	N
T	C	C	A	C	C	T	T	A	T	G	G	T
C	T	A	T	G	T	C	C	C	C	G	T	C
C	T	A	T	G	T	C	C	C	C	G	T	C
T	C	C	A	C	C	C	C	A	T	A	G	T
T	C	C	A	C	C	T	T	A	T	G	G	T
T	C	C	A	C	C	T	T	A	T	G	G	T
T	C	C	A	C	C	T	T	A	T	G	G	T
T	C	C	A	C	C	T	T	A	T	G	G	T
T	C	C	A	C	C	C	C	A	T	A	G	T
T	C	C	A	C	C	T	T	A	T	G	G	T
N	N	N	N	N	N	N	N	N	N	N	N	N
T	C	C	A	C	C	T	T	A	T	G	G	T
C	T	A	T	G	T	C	C	C	C	G	T	C
T	C	C	A	C	C	C	C	A	T	A	G	T
N	N	N	N	N	N	N	N	N	N	N	N	N
N	N	N	N	N	N	N	N	N	N	N	N	N
T	C	C	A	C	C	C	C	A	T	A	G	T
N	N	N	N	N	N	N	N	N	N	N	N	N
T	C	C	A	C	C	C	C	A	T	A	G	T
N	N	N	N	N	N	N	N	N	N	N	N	N

N	N	N	N	N	N	N	N	N	N	N	N	N
N	N	N	N	N	N	N	N	N	N	N	N	N
T	C	C	A	C	C	T	T	A	T	G	G	T
N	N	N	N	N	N	N	N	N	N	N	N	N
N	N	N	N	N	N	N	N	N	N	N	N	N
T	C	C	A	C	C	C	C	A	T	A	G	T
T	C	C	A	C	C	C	C	A	T	A	G	T
T	C	C	A	C	C	C	C	A	T	A	G	T
N	N	N	N	N	N	N	N	N	N	N	N	N
C	T	A	T	G	T	C	C	C	C	G	T	C
C	T	A	T	G	T	C	C	C	C	G	T	C
N	N	N	N	N	N	N	N	N	N	N	N	N
N	N	N	N	N	N	N	N	N	N	N	N	N
N	N	N	N	N	N	N	N	N	N	N	N	N
T	C	C	A	C	C	T	T	A	T	G	G	T
C	T	A	T	G	T	C	C	C	C	G	T	C
T	C	C	A	C	C	C	C	A	T	A	G	T
T	C	C	A	C	C	T	T	A	T	G	G	T
N	N	N	N	N	N	N	N	N	N	N	N	N
N	N	N	N	N	N	N	N	N	N	N	N	N
N	N	N	N	N	N	N	N	N	N	N	N	N
N	N	N	N	N	N	N	N	N	N	N	N	N
N	N	N	N	N	N	N	N	N	N	N	N	N
N	N	N	N	N	N	N	N	N	N	N	N	N
N	N	N	N	N	N	N	N	N	N	N	N	N
N	N	N	N	N	N	N	N	N	N	N	N	N
N	N	N	N	N	N	N	N	N	N	N	N	N
N	N	N	N	N	N	N	N	N	N	N	N	N
N	N	N	N	N	N	N	N	N	N	N	N	N
N	N	N	N	N	N	N	N	N	N	N	N	N
T	C	C	A	C	C	T	T	A	T	G	G	T
N	N	N	N	N	N	N	N	N	N	N	N	N
T	C	C	A	C	C	T	T	A	T	G	G	T
T	C	C	A	C	C	T	T	A	T	G	G	T
T	C	C	A	C	C	T	T	A	T	G	G	T
T	C	C	A	C	C	C	C	A	T	A	G	T
T	C	C	A	C	C	C	C	A	T	A	G	T
T	C	C	A	C	C	C	C	A	T	A	G	T
T	C	C	A	C	C	T	T	A	T	G	G	T
T	C	C	A	C	C	C	C	A	T	A	G	T
C	T	A	T	G	T	C	C	C	C	G	T	C
C	T	A	T	G	T	C	C	C	C	G	T	C
T	C	C	A	C	C	T	T	A	T	G	G	T
N	N	N	N	N	N	N	N	N	N	N	N	N
T	C	C	A	C	C	T	T	A	T	G	G	T
N	N	N	N	N	N	N	N	N	N	N	N	N
T	C	C	A	C	C	T	T	A	T	G	G	T
N	N	N	N	N	N	N	N	N	N	N	N	N
C	T	A	T	G	T	C	C	C	C	G	T	C
N	N	N	N	N	N	N	N	N	N	N	N	N
N	N	N	N	N	N	N	N	N	N	N	N	N
T	C	C	A	C	C	T	T	A	T	G	G	T
N	N	N	N	N	N	N	N	N	N	N	N	N
N	N	N	N	N	N	N	N	N	N	N	N	N
T	C	C	A	C	C	T	T	A	T	G	G	T
T	C	C	A	C	C	C	C	A	T	A	G	T
T	C	C	A	C	C	T	T	A	T	G	G	T

T	C	C	A	C	C	T	T	A	T	G	G	T
T	C	C	A	C	C	T	T	A	T	G	G	T
T	C	C	A	C	C	T	T	A	T	G	G	T
T	C	C	A	C	C	T	T	A	T	G	G	T
T	C	C	A	C	C	C	C	A	T	A	G	T
N	N	N	N	N	N	N	N	N	N	N	N	N
T	C	C	A	C	C	C	C	A	T	A	G	T
T	C	C	A	C	C	T	T	A	T	G	G	T
T	C	C	A	C	C	T	T	A	T	G	G	T
T	C	C	A	C	C	T	T	A	T	G	G	T
N	N	N	N	N	N	N	N	N	N	N	N	N
T	C	C	A	C	C	T	T	A	T	G	G	T
N	N	N	N	N	N	N	N	N	N	N	N	N
T	C	C	A	C	C	T	T	A	T	G	G	T
T	C	C	A	C	C	T	T	A	T	G	G	T
T	C	C	A	C	C	T	T	A	T	G	G	T
C	T	A	T	G	T	C	C	C	C	G	T	C
T	C	C	A	C	C	C	C	A	T	A	G	T
N	N	N	N	N	N	N	N	N	N	N	N	N
T	C	C	A	C	C	T	T	A	T	G	G	T
T	C	C	A	C	C	T	T	A	T	G	G	T
T	C	C	A	C	C	T	T	A	T	G	G	T
T	C	C	A	C	C	T	T	A	T	G	G	T
T	C	C	A	C	C	C	C	A	T	A	G	T
T	C	C	A	C	C	C	C	A	T	A	G	T
N	N	N	N	N	N	N	N	N	N	N	N	N
T	C	C	A	C	C	C	C	A	T	A	G	T
T	C	C	A	C	C	C	C	A	T	A	G	T
N	N	N	N	N	N	N	N	N	N	N	N	N
T	C	C	A	C	C	C	C	A	T	A	G	T
T	C	C	A	C	C	C	C	A	T	A	G	T
T	C	C	A	C	C	C	C	A	T	A	G	T
T	C	C	A	C	C	T	T	A	T	G	G	T
N	N	N	N	N	N	N	N	N	N	N	N	N
T	C	C	A	C	C	T	T	A	T	G	G	T
T	C	C	A	C	C	C	C	A	T	A	G	T
T	C	C	A	C	C	T	T	A	T	G	G	T
T	C	C	A	C	C	C	C	A	T	A	G	T
C	T	A	T	G	T	C	C	C	C	G	T	C
T	C	C	A	C	C	C	C	A	T	A	G	T
T	C	C	A	C	C	C	C	A	T	A	G	T
C	T	A	T	G	T	C	C	C	C	G	T	C
N	N	N	N	N	N	N	N	N	N	N	N	N
N	N	N	N	N	N	N	N	N	N	N	N	N
T	C	C	A	C	C	C	C	A	T	A	G	T
T	C	C	A	C	C	C	C	A	T	A	G	T
N	N	N	N	C	C	C	C	A	T	A	G	T
T	C	C	A	C	C	C	C	A	T	A	G	T
T	C	C	A	C	C	C	C	A	T	A	G	T
T	C	C	A	C	C	C	C	A	T	A	G	T
T	C	C	A	C	C	T	T	A	T	G	G	T
T	C	C	A	C	C	T	T	A	T	G	G	T
T	C	C	A	C	C	T	T	A	T	G	G	T
N	N	N	N	N	N	N	N	N	N	N	N	N
T	C	C	A	C	C	C	C	A	T	A	G	T
T	C	C	A	C	C	C	C	A	T	A	G	T
N	N	N	N	N	N	N	N	N	N	N	N	N

N	N	N	N	N	N	N	N	N	N	N	N	N
N	N	N	N	N	N	N	N	N	N	N	N	N
N	N	N	N	N	N	N	N	N	N	N	N	N
T	C	C	A	C	C	T	T	A	T	G	G	T
T	C	C	A	C	C	T	T	A	T	G	G	T
N	N	N	N	N	N	N	N	N	N	N	N	N
N	N	N	N	N	N	N	N	N	N	N	N	N
N	N	N	N	N	N	N	N	N	N	N	N	N
N	N	N	N	N	N	N	N	N	N	N	N	N
N	N	N	N	N	N	N	N	N	N	N	N	N
N	N	N	N	N	N	N	N	N	N	N	N	N
T	C	C	A	C	C	T	T	A	T	G	G	T
N	N	N	N	N	N	N	N	N	N	N	N	N
T	C	C	A	C	C	T	T	A	T	G	G	T
T	C	C	A	C	C	T	T	A	T	G	G	T
T	C	C	A	C	C	C	C	A	T	A	G	T
N	N	N	N	N	N	N	N	N	N	N	N	N
C	T	A	T	G	T	C	C	C	C	G	T	C
T	C	C	A	C	C	T	T	A	T	G	G	T
T	C	C	A	C	C	T	T	A	T	G	G	T
T	C	C	A	C	C	T	T	A	T	G	G	T
N	N	N	N	N	N	N	N	N	N	N	N	N
N	N	N	N	N	N	N	N	N	N	N	N	N
N	N	N	N	N	N	N	N	N	N	N	N	N
T	C	C	A	C	C	T	T	A	T	G	G	T
N	N	N	N	N	N	N	N	N	N	N	N	N
N	N	N	N	N	N	N	N	N	N	N	N	N
T	C	C	A	C	C	T	T	A	T	G	G	T
C	T	A	T	G	T	C	C	C	C	G	T	C
T	C	C	A	C	C	T	T	A	T	G	G	T
T	C	C	A	C	C	C	C	A	T	A	G	T
T	C	C	A	C	C	T	T	A	T	G	G	T
T	C	C	A	C	C	T	T	A	T	G	G	T
T	C	C	A	C	C	T	T	A	T	G	G	T
T	C	C	A	C	C	C	C	A	T	A	G	T
C	T	A	T	G	T	C	C	C	C	G	T	C
C	T	A	T	G	T	C	C	C	C	G	T	C
N	N	N	N	N	N	N	N	N	N	N	N	N
N	N	N	N	N	N	N	N	N	N	N	N	N
N	N	N	N	N	N	N	N	N	N	N	N	N
T	C	C	A	C	C	C	C	A	T	A	G	T
N	N	N	N	N	N	N	N	N	N	N	N	N
N	N	N	N	N	N	N	N	N	N	N	N	N
T	C	C	A	C	C	T	T	A	T	G	G	T
N	N	N	N	N	N	N	N	N	N	N	N	N
N	N	N	N	N	N	N	N	N	N	N	N	N
N	N	N	N	N	N	N	N	N	N	N	N	N
T	C	C	A	C	C	T	T	A	T	G	G	T
T	C	C	A	C	C	C	C	A	T	A	G	T
T	C	C	A	C	C	T	T	A	T	G	G	T
T	C	C	A	C	C	C	C	A	T	A	G	T
N	N	N	N	N	N	N	N	N	N	N	N	N
N	N	N	N	N	N	N	N	N	N	N	N	N
T	C	C	A	C	C	T	T	A	T	G	G	T
N	N	N	N	N	N	N	N	N	N	N	N	N
N	N	N	N	N	N	N	N	N	N	N	N	N

Ger23	Ger24	Ger25	Ger26	ICD1	ICD2	ICD3	ICD4	ICD5	ICD6	ICD7	ICD8	ICD9	ICD10	ICD11
N	N	N	N	N	1	N	N	N	N	7	N	N	N	N
N	N	N	N	N	N	N	N	N	N	N	N	N	N	N
N	N	N	N	N	N	N	N	N	N	N	N	N	N	N
A	A	G	G	C	1	T	T	T	T	7	T	A	A	C
N	N	N	N	G	1	G	G	T	A	0	T	A	T	T
N	N	N	N	G	1	T	T	T	T	7	T	A	T	T
A	A	G	G	G	1	T	T	T	T	7	T	A	T	T
A	A	G	G	G	1	T	T	T	T	7	T	A	T	T
G	A	C	A	G	1	T	T	T	T	7	T	A	T	T
N	N	N	N	N	N	N	N	N	N	N	N	N	N	N
A	A	G	G	G	0	G	T	T	A	7	T	A	A	C
A	A	G	G	C	1	T	T	T	T	7	T	A	T	T
N	N	N	N	G	2	T	T	A	T	7	C	G	A	C
N	N	N	N	G	1	T	G	T	T	0	T	A	T	T
A	G	G	G	G	1	T	T	T	T	7	T	A	T	T
N	N	N	N	G	1	T	T	T	T	7	T	A	T	T
A	G	G	G	C	1	G	T	T	T	7	N	N	N	N
N	N	N	N	G	1	T	T	T	T	7	T	A	T	T
A	A	G	G	G	1	T	T	T	T	7	T	A	T	T
A	A	G	G	G	1	T	G	T	T	0	T	A	T	T
N	N	N	N	G	0	G	T	T	T	7	T	A	A	C
A	A	G	G	N	N	N	N	N	N	N	T	A	T	T
A	G	G	G	G	1	T	T	T	T	7	T	A	T	T
A	G	G	G	N	1	T	T	T	T	7	T	A	T	T
G	A	C	A	C	1	T	T	T	T	7	T	A	T	T
A	G	G	G	G	0	G	T	T	A	7	T	A	T	T
A	G	G	G	G	2	T	T	A	T	7	C	G	A	C
A	G	G	G	G	1	T	T	T	T	7	T	A	T	T
A	A	G	G	G	2	G	T	A	T	7	C	G	A	C
A	G	G	G	G	1	T	T	T	T	7	T	A	T	T
N	N	N	N	G	1	T	T	T	T	7	T	A	T	T
N	N	N	N	G	1	T	T	T	T	7	T	A	T	T
A	A	G	G	G	1	T	T	T	T	7	T	A	T	T
N	N	N	N	G	1	T	T	T	T	7	T	A	T	T
A	A	G	N	G	2	T	T	A	A	7	C	G	A	C
A	A	G	G	G	1	T	T	T	T	7	T	A	T	T
N	N	N	N	G	1	A	T	T	A	7	T	A	T	T
N	N	N	N	G	1	T	T	T	T	7	T	A	T	T
A	A	G	G	C	1	T	T	T	T	7	T	A	T	T
A	G	G	G	N	N	N	N	N	N	N	T	A	T	T
A	G	G	G	N	N	N	N	N	N	N	T	A	T	T
A	G	G	G	G	1	T	T	T	T	7	T	A	T	T
A	A	G	G	G	1	T	G	T	T	0	T	A	T	T
A	A	G	G	G	2	T	T	A	T	7	C	G	A	C
A	G	G	G	G	1	T	G	T	T	0	T	A	T	T
A	G	G	G	N	N	N	N	N	N	N	C	G	A	C
A	G	G	G	G	1	T	T	T	T	7	T	A	T	T
N	N	N	N	C	1	T	T	T	T	7	T	A	T	T
A	G	G	G	G	1	T	T	T	T	7	T	A	T	T
A	A	G	G	G	1	T	T	T	T	7	T	A	T	T
N	N	N	N	G	1	T	T	T	T	7	T	A	T	T
N	N	N	N	N	N	N	N	N	N	N	N	N	N	N
A	A	C	C	N	2	T	T	A	T	7	N	N	N	N
N	N	N	N	N	N	N	N	N	N	N	N	N	N	N
N	N	N	N	N	N	N	N	N	N	N	N	N	N	N
N	N	N	N	G	1	T	T	T	T	7	T	A	T	T

A	A	G	G	G	1	T	T	T	N	N	T	A	T	T
G	A	C	A	G	1	T	T	T	T	7	T	A	A	C
N	N	N	N	G	1	T	G	T	T	0	N	N	N	N
A	G	G	G	C	1	T	T	T	T	7	T	A	T	T
A	G	G	G	G	2	T	T	A	T	7	C	G	A	C
G	A	C	A	G	2	T	T	A	T	7	C	G	A	C
N	N	N	N	G	1	G	T	T	A	7	T	A	T	T
N	N	N	N	G	1	T	G	T	T	0	T	A	T	T
N	N	N	N	G	1	T	G	T	T	0	T	A	T	T
N	N	N	N	G	1	G	T	T	T	7	T	A	T	T
A	G	G	G	C	1	G	T	T	T	7	T	A	T	T
A	G	G	G	C	2	T	T	A	T	7	C	G	A	C
N	N	N	N	G	1	T	T	T	T	7	T	A	T	T
A	G	G	G	G	1	T	T	T	T	7	T	A	T	T
A	G	G	G	N	N	N	N	N	N	N	C	G	A	C
A	G	G	G	G	2	T	T	T	T	7	T	A	T	T
A	G	G	N	G	1	T	T	T	T	7	T	A	T	T
A	G	G	G	N	N	N	N	N	N	N	T	A	T	T
A	A	G	G	G	1	T	T	T	T	7	T	A	T	C
A	G	G	G	G	2	T	T	A	T	7	C	G	A	C
A	A	G	N	N	N	N	N	N	N	N	C	G	A	C

A	A	G	G	G	2	T	T	A	T	7	C	G	A	C
A	A	G	G	G	1	T	T	T	T	7	T	A	T	T
A	G	G	G	C	1	T	T	T	T	7	T	A	T	T
A	G	G	G	G	1	T	T	T	N	N	T	A	T	T
A	G	G	G	G	2	T	T	A	T	7	C	G	A	C
G	A	C	A	C	2	T	T	A	T	7	C	G	A	C
A	A	G	G	G	1	T	T	T	T	7	T	A	T	T
A	G	G	G	G	1	G	T	T	T	7	T	A	T	T
N	N	N	N	G	2	T	T	A	T	7	C	G	A	C
G	A	C	A	G	1	T	T	T	T	7	T	A	T	T
A	G	G	G	G	1	T	T	T	T	7	T	A	T	T
A	G	G	G	G	2	T	T	A	T	7	C	G	A	C
A	G	G	G	N	1	T	N	N	N	N	T	A	T	T
G	A	C	A	C	1	T	T	T	T	7	T	A	T	T
N	N	N	N	N	2	T	T	A	T	7	C	G	A	C
A	G	G	G	G	1	T	G	T	T	0	T	A	T	T
G	A	C	A	G	1	T	T	T	T	7	T	A	T	T
G	A	C	A	G	1	T	T	T	T	7	T	A	T	T
A	A	G	G	G	1	T	T	T	N	N	T	A	T	T
A	G	G	G	G	2	T	T	A	T	7	C	G	A	C
A	G	G	G	C	1	T	T	T	T	7	T	A	T	T
A	G	G	G	G	1	T	T	T	N	N	T	A	T	T
A	G	G	G	G	2	G	T	A	T	7	C	G	A	C
A	A	G	G	G	1	T	T	T	T	7	T	A	T	T
A	G	G	G	N	N	N	N	N	N	N	T	A	A	C
N	N	N	N	G	1	T	T	T	T	7	T	A	A	C
A	G	G	G	G	2	T	T	A	T	7	C	G	A	C
G	A	C	A	G	1	T	T	T	T	7	T	A	A	C
A	A	G	G	G	1	T	T	T	N	N	T	A	T	T
N	N	N	N	G	1	G	G	T	T	7	T	A	T	T
N	N	N	N	N	1	T	T	T	T	7	T	A	T	T
A	A	G	G	C	1	T	G	T	T	0	T	A	T	T
N	N	N	N	N	1	G	T	T	T	0	T	A	T	T
A	A	G	N	G	0	G	T	T	A	7	T	A	A	C
N	N	N	N	G	1	T	T	T	T	7	T	A	T	T

N	N	N	N	N	N	N	N	N	N	N	N	N	N	N
N	N	N	N	N	N	N	N	N	N	N	N	N	N	N
A	G	G	G	G	1	T	T	T	T	7	T	A	T	T
N	N	N	N	C	1	G	T	T	T	7	T	A	T	T
N	N	N	N	G	1	T	T	T	T	0	T	A	T	T
A	A	G	G	G	1	G	T	T	A	7	T	A	T	T
A	A	G	G	C	1	T	T	T	T	7	T	A	T	T
A	A	G	G	C	1	T	T	T	T	7	T	A	T	T
N	N	N	N	G	1	T	G	T	T	0	T	A	T	T
G	A	C	A	G	2	T	T	A	T	7	C	G	A	C
G	A	C	A	G	1	T	T	T	T	7	T	A	T	T
N	N	N	N	G	1	T	T	T	T	7	T	A	T	T
N	N	N	N	N	N	N	N	N	N	N	C	G	A	C
N	N	N	N	C	1	T	T	T	T	7	T	A	T	T
A	G	G	G	G	1	T	T	T	T	7	T	A	T	T
G	A	C	A	G	2	T	T	A	T	7	C	G	A	C
A	A	G	G	N	N	N	N	N	N	N	C	G	A	C
A	G	G	G	G	1	T	T	T	T	7	T	A	T	T
N	N	N	N	C	1	T	T	T	T	7	T	A	A	C
N	N	N	N	G	1	T	T	T	T	7	T	A	T	T
N	N	N	N	G	0	G	T	T	A	7	T	A	A	C
N	N	N	N	G	1	T	T	T	T	7	T	A	T	T
N	N	N	N	G	1	T	T	T	A	7	T	A	A	C
N	N	N	N	G	2	T	T	A	T	7	C	G	A	C
N	N	N	N	G	1	T	T	T	T	7	T	A	T	T
N	N	N	N	G	1	T	G	T	T	0	T	A	T	T
N	N	N	N	G	1	T	T	T	T	7	T	A	A	C
N	N	N	N	G	1	T	G	T	T	0	T	A	T	T
N	N	N	N	G	1	T	T	T	T	7	T	A	T	T
N	N	N	N	G	2	T	T	A	T	7	C	G	A	C
A	G	G	G	G	1	T	T	T	T	7	T	A	T	T
N	N	N	N	C	0	G	T	T	A	7	T	A	A	C
A	G	G	G	C	2	T	T	A	A	7	C	G	A	C
A	G	G	G	C	2	T	T	A	T	7	C	G	A	C
A	G	G	G	C	1	T	T	T	T	7	T	A	T	T
A	A	G	G	G	1	T	T	T	A	7	T	A	T	T
A	A	G	G	G	0	G	T	T	A	7	T	A	A	C
A	A	G	G	C	1	T	T	T	T	7	T	A	T	T
A	G	G	G	G	1	T	T	T	T	7	T	A	T	T
A	A	G	G	G	2	T	T	A	T	7	C	G	A	C
G	A	C	A	C	1	T	T	T	T	7	T	A	T	T
G	A	C	A	C	2	T	T	A	T	7	C	G	A	C
A	G	G	G	N	N	N	N	N	N	N	T	A	T	T
N	N	N	N	G	0	G	T	T	A	7	T	A	A	C
A	G	G	G	G	1	T	G	T	T	0	T	A	T	T
N	N	N	N	C	1	T	G	T	T	0	T	A	T	T
A	G	G	G	N	N	N	N	N	N	N	T	A	T	T
N	N	N	N	N	N	N	N	N	N	N	T	A	T	T
G	A	C	A	C	1	T	T	T	T	7	T	A	T	T
N	N	N	N	N	0	G	T	T	T	7	T	A	A	C
N	N	N	N	N	N	N	N	N	N	N	N	N	N	N
A	G	G	G	G	1	T	T	T	T	7	N	N	N	N
N	N	N	N	G	2	T	T	A	T	7	C	G	A	C
N	N	N	N	G	2	T	T	A	T	7	C	G	A	C
A	G	G	G	G	2	T	T	A	T	7	C	G	A	C
A	A	N	N	G	1	T	T	T	T	7	T	A	T	T
A	G	G	G	G	1	T	T	T	T	7	T	A	T	T

A	G	G	G	G	1	T	T	T	T	7	T	A	T	T
A	G	G	G	G	1	T	T	T	T	7	T	A	T	T
A	G	G	G	G	1	T	G	T	T	0	T	A	T	T
A	G	G	G	G	1	T	G	T	T	0	T	A	T	T
A	A	G	N	G	2	T	T	A	T	7	C	G	A	C
N	N	N	N	G	2	T	T	A	T	7	C	G	A	C
A	A	G	G	G	1	T	T	T	T	7	T	A	T	T
A	G	G	G	G	1	T	T	T	T	7	T	A	T	T
A	G	G	G	C	1	T	T	T	A	7	T	A	T	T
A	G	G	G	G	2	T	T	A	T	7	C	G	A	C
N	N	N	N	N	N	N	N	N	N	N	T	A	T	T
A	G	G	N	G	1	T	T	T	T	7	T	A	T	T
N	N	N	N	N	N	N	N	N	N	N	T	A	A	C
A	G	G	G	G	2	T	T	A	T	7	C	G	A	C
A	G	N	N	G	1	T	T	T	T	7	T	A	A	C
A	G	G	G	G	0	G	T	T	A	0	T	A	T	T
G	A	C	A	G	1	T	T	T	T	7	T	A	T	T
A	A	G	G	G	2	T	T	A	T	7	C	G	A	C
N	N	N	N	G	1	T	T	T	T	7	T	A	T	T
A	G	G	G	G	1	G	T	T	A	7	T	A	T	T
A	G	G	G	C	1	T	T	T	T	7	T	A	T	T
A	G	G	G	G	1	G	T	T	N	N	T	A	T	T
A	G	G	G	G	1	G	T	T	T	7	T	A	T	T
A	A	G	G	G	2	T	T	A	T	7	C	G	A	C
A	A	G	G	G	1	T	T	T	T	7	T	A	T	T
N	N	N	N	N	N	N	N	N	N	N	T	A	T	T
A	A	G	G	C	2	T	T	A	T	7	C	G	A	C
A	A	G	G	C	1	T	T	T	T	7	T	A	T	T
N	N	N	N	C	0	G	T	T	A	0	T	A	T	T
A	A	G	G	G	0	G	T	T	A	0	T	A	T	T
A	A	G	G	G	2	T	T	A	N	N	C	G	A	C
A	A	G	G	G	1	T	T	T	T	7	T	A	T	T
A	G	G	G	G	2	T	T	A	T	7	C	G	A	C
N	N	N	N	G	1	T	T	T	N	N	T	A	T	T
A	G	G	G	C	1	T	T	T	T	7	T	A	T	T
A	A	G	G	G	2	T	T	A	T	7	C	G	A	C
A	G	G	G	G	1	T	T	T	T	7	T	A	T	T
A	A	G	G	G	1	T	T	T	T	7	T	A	T	T
G	A	C	A	G	1	T	G	T	N	N	T	A	T	T
A	A	G	G	G	1	T	T	T	T	7	T	A	T	T
A	A	G	G	N	2	G	T	A	T	7	T	A	A	C
G	A	C	A	G	1	T	T	T	T	7	T	A	T	T
N	N	N	N	G	1	T	T	T	T	7	T	A	T	T
N	N	N	N	N	N	N	N	N	N	N	T	A	T	T
A	A	G	G	G	1	G	T	T	A	7	T	A	T	T
A	A	G	G	N	N	N	N	N	N	N	C	G	A	C
A	A	G	N	C	1	G	T	T	A	7	T	A	T	T
A	A	G	N	G	1	T	T	T	T	7	T	A	T	T
A	A	G	G	G	1	G	T	T	G	7	T	A	T	T
A	A	G	G	C	2	T	T	A	T	7	T	A	A	C
A	G	G	G	G	1	T	T	T	T	7	T	A	T	T
A	G	G	G	C	2	T	T	A	T	7	C	G	A	C
A	G	G	G	G	1	T	T	T	T	7	T	A	T	T
N	N	N	N	G	1	T	T	T	T	7	T	A	T	T
A	A	G	G	G	1	G	T	T	T	7	T	A	T	T
A	A	G	G	G	2	T	T	A	T	7	C	G	A	C
N	N	N	N	G	0	G	T	T	A	0	T	A	T	T

N	N	N	N	C	1	T	G	T	T	0	T	A	T	T
N	N	N	N	G	1	T	G	T	T	0	T	A	T	T
N	N	N	N	C	1	G	T	T	T	7	T	A	T	T
A	G	G	G	C	1	T	T	T	T	7	T	A	T	T
A	G	G	G	C	1	T	T	T	T	7	T	A	T	T
N	N	N	N	N	N	N	N	N	N	N	N	N	N	N
N	N	N	N	G	1	T	T	T	T	7	T	A	T	T
N	N	N	N	C	2	T	T	A	T	7	C	G	A	C
N	N	N	N	C	2	T	T	A	N	N	C	G	A	C
N	N	N	N	C	1	T	T	T	T	7	T	A	A	C
N	N	N	N	G	1	T	G	T	T	0	T	A	T	T
A	G	G	G	G	1	T	T	T	T	7	T	A	T	T
N	N	N	N	G	1	G	T	T	T	7	T	A	T	T
A	G	G	G	N	N	N	G	T	T	0	T	A	T	T
A	G	G	G	C	1	T	T	T	T	7	T	A	T	T
A	A	G	N	G	1	T	T	T	T	7	T	A	T	T
N	N	N	N	G	1	T	T	T	T	7	T	A	T	T
G	A	C	A	G	1	G	T	T	T	7	T	A	T	T
A	G	G	G	G	1	T	T	T	T	7	T	A	T	T
A	G	G	G	G	2	T	T	A	T	7	C	G	A	C
A	G	G	G	C	1	T	T	T	T	7	T	A	T	T
N	N	N	N	C	1	T	T	T	T	7	T	A	T	T
N	N	N	N	C	1	T	T	T	T	7	T	A	A	C
N	N	N	N	N	1	G	T	T	T	7	T	A	A	C
A	G	G	G	C	2	T	T	A	T	7	C	G	A	C
N	N	N	N	G	1	T	T	T	T	7	T	A	A	C
N	N	N	N	G	1	T	G	T	T	0	T	A	T	T
A	G	G	G	N	N	N	N	N	N	N	C	G	A	C
G	A	C	N	G	1	T	T	T	T	7	T	A	T	T
A	G	G	N	G	0	G	T	T	T	0	T	A	T	T
A	A	G	N	G	1	T	T	T	T	7	T	A	T	T
A	G	G	N	G	2	T	T	A	T	7	C	G	A	C
A	G	G	N	G	0	G	T	T	A	7	T	A	A	C
A	G	G	G	C	2	T	T	A	T	7	C	G	A	C
A	A	G	G	G	2	T	T	A	T	7	C	G	A	C
G	A	C	A	G	1	T	T	T	T	7	T	A	T	T
G	A	C	A	G	2	T	T	A	T	7	C	G	A	C
N	N	N	N	G	1	T	G	T	T	0	T	A	T	T
N	N	N	N	G	1	T	G	T	T	0	T	A	T	T
N	N	N	N	G	1	T	G	T	T	0	T	A	T	T
A	A	G	G	G	1	T	T	T	N	N	T	A	T	T
N	N	N	N	G	1	G	G	T	T	0	N	N	N	N
N	N	N	N	C	1	T	G	T	T	0	T	A	T	T
A	G	G	G	G	1	T	G	T	T	0	T	A	T	T
N	N	N	N	G	1	T	T	T	T	7	T	A	T	T
N	N	N	N	G	1	T	G	T	T	0	T	A	T	T
N	N	N	N	G	2	T	T	A	A	7	C	G	A	C
A	G	G	G	C	1	T	T	T	T	7	T	A	A	C
A	A	G	G	G	2	T	T	A	T	7	C	G	A	C
A	G	G	G	G	0	G	T	T	A	7	T	A	A	C
A	A	G	G	N	N	N	N	N	N	N	T	A	T	T
N	N	N	N	N	N	N	N	N	N	N	N	N	N	N
N	N	N	N	G	1	G	T	T	T	7	T	A	T	T
A	G	G	G	G	0	G	T	T	A	7	T	A	A	C
N	N	N	N	C	2	T	T	A	T	7	C	G	A	C
N	N	N	N	N	N	N	N	N	N	N	N	N	N	N

ICD12	ICD13	ICD14	ISL1	ISL2	ISL3	ISL4	ISL5	ISL6	ME1	ME2	ME3	ME4	ME5	ME6	ME7	ME8
N	N	1	T	G	C	C	G	C	8	T	C	G	2	6	T	T
N	N	N	T	G	C	C	G	C	0	T	C	G	2	6	T	T
N	N	N	T	A	C	C	C	C	N	N	N	N	N	N	N	N
T	C	0	C	G	C	C	G	C	0	T	C	G	2	6	T	T
T	A	0	T	G	C	C	C	C	0	T	G	G	2	4	C	T
G	A	1	N	N	N	N	N	N	N	N	N	N	N	N	N	N
G	A	1	T	G	C	G	G	C	8	T	G	G	2	4	C	T
G	A	1	T	G	C	C	C	C	0	C	G	G	2	6	T	T
G	A	1	T	G	C	C	C	C	0	T	G	C	2	6	T	C
N	N	N	T	A	C	G	G	N	0	T	G	G	0	4	T	C
T	C	0	C	G	C	C	G	C	0	T	G	G	0	4	T	T
G	A	1	T	A	C	G	G	T	0	T	G	G	0	4	T	T
T	A	0	T	A	C	C	G	T	0	C	G	G	2	6	T	T
T	A	0	T	G	C	G	G	C	0	C	G	G	2	6	T	T
G	A	1	C	G	C	C	G	C	8	T	G	G	2	4	C	T
G	A	1	T	G	C	C	G	C	0	T	G	G	0	4	T	T
N	N	N	T	G	C	C	G	C	8	T	G	G	2	4	C	T
G	A	1	T	G	C	C	G	C	8	T	G	G	2	4	C	T
G	A	1	T	A	C	C	G	T	0	T	C	G	0	4	T	C
T	A	0	T	G	C	C	C	C	0	T	C	G	0	4	T	C
T	C	0	T	G	C	C	G	C	0	T	C	G	2	6	T	T
G	A	0	N	N	N	N	N	N	N	N	N	N	N	N	N	N
G	A	1	T	G	A	G	C	C	0	T	G	C	2	6	T	C
G	A	1	T	A	C	G	G	T	0	T	C	G	0	4	T	C
G	A	1	T	A	C	C	C	C	0	T	C	C	2	4	C	T
G	A	1	T	G	A	G	C	C	8	T	G	G	2	4	C	T
T	A	0	T	A	C	C	G	T	0	T	G	G	0	4	T	C
G	A	N	T	G	C	C	C	C	8	T	G	G	2	4	C	T
T	A	0	N	N	N	N	N	N	0	T	G	G	2	4	C	T
G	A	0	T	G	C	C	G	C	0	T	G	G	2	4	C	T
G	A	N	N	N	N	N	N	N	0	T	G	G	0	4	T	T
G	A	0	N	N	N	N	N	N	8	T	G	G	2	4	C	T
G	A	1	T	A	C	G	G	C	0	C	G	G	2	6	T	T
G	A	1	T	A	C	C	C	C	0	T	C	G	2	6	T	T
T	A	1	C	G	C	C	G	C	0	T	G	G	2	4	C	T
G	A	0	T	G	C	C	C	C	8	T	G	G	2	4	C	T
G	A	0	N	N	N	N	N	N	N	N	N	N	N	N	N	N
N	N	N	N	N	N	N	N	N	0	T	G	G	2	4	T	C
G	A	0	N	N	N	N	N	N	0	T	G	G	2	4	C	T
G	A	0	T	G	C	C	G	C	8	T	G	G	2	4	C	T
G	A	1	T	G	A	G	C	C	0	T	G	G	2	4	C	T
G	A	1	T	G	A	G	C	C	0	T	G	G	2	4	C	T
T	A	0	C	G	C	C	G	C	0	T	G	G	0	4	T	C
T	A	0	C	G	C	C	G	C	0	T	G	G	2	4	C	T
T	A	0	T	A	C	G	G	T	0	T	C	G	2	6	T	T
T	A	0	T	G	C	C	C	C	8	T	G	G	2	4	C	T
G	A	1	T	G	A	G	C	C	0	C	G	G	2	6	T	T
G	A	0	T	G	C	C	C	C	0	T	G	G	2	4	C	T
G	A	1	T	A	C	C	G	T	0	T	C	G	2	6	T	T
G	A	0	T	A	C	C	G	T	0	T	G	G	0	4	T	C
G	A	1	T	G	C	C	G	C	0	C	G	G	2	6	T	T
N	N	N	C	G	C	C	G	C	0	C	G	G	2	6	T	T
N	N	N	C	G	C	C	G	C	0	T	C	G	0	4	T	C
N	N	N	C	G	C	G	C	C	0	T	C	G	2	6	T	T
N	N	N	T	G	A	G	C	C	0	T	G	G	0	4	T	C
G	A	1	N	N	N	N	N	N	N	N	N	N	N	N	N	N

G	A	1	T	A	C	C	C	C	0	T	C	G	2	6	T	T
T	C	0	T	G	A	G	C	C	0	T	G	G	0	4	T	C
N	N	N	C	G	C	G	C	C	0	T	C	G	2	6	T	T
G	A	1	T	G	A	G	C	C	N	N	N	N	N	N	N	N
T	A	0	T	G	C	C	C	C	0	C	G	G	2	6	T	T
T	A	0	N	N	N	N	N	N	0	T	C	G	0	4	T	C
G	A	1	T	G	C	C	C	C	0	T	C	G	0	4	T	C
T	A	0	T	G	C	C	G	C	8	T	G	G	2	4	C	T
G	A	0	T	G	C	C	G	C	8	T	G	G	2	4	C	T
G	A	0	N	N	N	N	N	N	0	T	C	G	2	6	T	T
G	A	1	T	A	C	G	G	T	N	N	N	N	N	N	N	N
T	A	0	T	A	C	C	G	T	0	T	G	G	0	2	T	C
G	A	1	C	G	C	C	G	C	N	N	N	N	N	N	N	N
G	A	1	T	A	C	C	G	T	0	T	C	G	2	4	C	T
T	A	0	N	N	N	N	N	N	N	N	N	N	N	N	N	N
G	A	1	T	A	C	C	G	T	0	T	C	C	2	4	C	T
G	A	0	T	A	C	C	G	T	0	T	G	C	2	6	T	T
G	A	1	T	A	C	G	C	C	0	T	G	C	0	4	T	C
T	A	1	N	N	N	N	N	N	0	T	G	G	0	4	T	C
T	A	0	T	A	C	C	G	T	0	T	G	C	0	4	T	C
T	A	0	C	G	C	C	C	C	0	T	C	G	0	4	T	C

T	A	0	C	G	C	C	C	C	8	T	G	C	2	4	C	T
G	A	0	T	G	C	G	G	C	0	T	G	G	2	4	T	C
G	A	0	C	G	C	C	G	C	0	T	G	C	2	6	T	T
G	A	1	T	G	A	C	N	N	0	T	G	C	2	4	T	C
T	A	0	T	A	C	G	C	C	0	T	G	G	2	4	T	C
T	A	0	T	A	C	G	C	C	0	T	G	G	2	4	C	T
G	A	1	N	N	N	N	N	N	N	N	N	N	N	N	N	N
G	A	1	T	G	C	C	G	C	0	T	G	C	2	6	T	C
T	A	0	C	G	C	C	G	C	0	T	G	G	0	4	T	T
G	A	1	T	G	C	C	G	C	0	T	G	G	2	4	C	T
G	A	N	T	A	C	G	C	C	0	T	G	C	2	6	T	C
T	A	0	N	N	N	N	N	N	N	N	N	N	N	N	N	N
T	A	1	N	N	N	N	N	N	8	T	G	C	2	4	T	C
G	A	0	T	A	C	C	G	T	0	T	C	G	2	4	C	T
T	A	0	T	A	C	G	G	T	0	T	C	G	0	4	T	C
T	A	0	T	A	C	C	G	T	0	T	C	G	2	4	C	T
G	A	0	T	A	C	C	G	T	N	N	N	N	N	N	N	N
G	A	N	T	G	C	C	G	C	8	T	G	C	2	4	C	T
G	A	1	C	G	C	C	G	C	8	T	G	G	0	4	T	C
T	A	0	N	N	N	N	N	N	N	N	N	N	N	N	N	N
G	A	0	T	A	C	C	G	T	0	T	G	C	0	4	T	C
G	A	1	T	G	C	C	G	C	0	T	G	G	0	4	T	C
T	A	0	N	N	N	N	N	N	0	T	G	C	2	4	T	C
G	A	1	T	G	C	G	G	C	0	T	G	G	2	4	C	T
T	C	0	T	G	C	C	G	C	0	T	G	G	2	4	C	T
T	C	0	T	G	C	G	G	C	0	T	G	G	0	2	T	C
T	A	1	T	G	C	C	G	C	0	T	G	G	2	4	C	T
T	C	0	T	G	C	G	G	C	0	T	G	G	2	4	C	T
G	A	1	C	G	C	C	G	C	0	T	G	C	0	4	T	C
T	A	0	T	A	C	C	C	C	0	T	C	G	2	6	T	T
G	A	1	T	G	C	C	G	T	0	T	G	C	2	6	T	C
T	A	0	T	G	C	C	C	C	0	T	C	G	0	4	T	C
T	A	0	T	G	C	G	G	T	0	T	G	G	2	4	C	T
T	C	0	N	N	N	N	N	N	0	T	C	G	2	6	T	T
G	A	N	T	A	C	G	G	C	0	C	G	G	2	6	T	T

N	N	N	T	A	C	G	G	C	0	T	C	G	0	4	T	C
N	N	N	T	G	A	G	C	C	N	N	N	N	N	N	N	N
G	A	1	T	G	C	C	G	C	8	T	G	G	2	4	C	T
G	A	0	T	G	C	G	G	C	0	T	G	G	2	4	C	T
T	A	0	T	G	C	C	C	C	0	T	G	G	0	4	T	C
G	A	1	C	G	C	G	N	N	0	T	G	G	0	4	T	C
G	A	1	T	G	C	C	G	C	0	T	G	G	2	4	C	T
G	A	N	T	G	C	C	G	C	0	T	C	G	2	6	T	T
T	A	0	T	G	C	G	G	T	0	C	G	G	2	4	C	T
T	A	0	N	N	N	N	N	N	N	N	N	N	N	N	N	N
G	A	1	T	A	C	G	G	T	0	T	C	C	2	4	C	T
G	A	1	N	N	N	N	N	N	8	T	G	G	2	4	C	T
T	A	0	N	N	N	N	N	N	0	T	C	G	0	4	T	C
G	A	1	C	G	C	C	G	C	0	T	C	G	2	6	T	T
G	A	1	T	G	C	C	G	C	8	T	G	G	2	4	C	T
T	A	0	T	A	C	G	G	T	0	C	G	G	2	6	T	T
T	A	0	N	N	N	N	N	N	0	T	C	G	0	4	T	C
G	A	1	N	N	N	N	N	N	N	N	N	N	N	N	N	N
T	C	0	T	G	C	C	C	C	0	T	C	G	0	4	T	C
G	A	1	N	N	N	N	N	N	N	N	N	N	N	N	N	N
T	C	0	T	A	C	C	G	T	8	T	G	G	2	4	C	T
G	A	1	T	G	A	G	C	C	0	T	G	G	2	4	T	C
T	C	0	T	G	C	C	G	C	0	T	G	G	0	4	T	C
T	A	0	T	G	C	C	C	C	0	T	G	G	2	4	C	T
G	A	1	T	G	A	G	C	C	0	T	G	G	2	4	C	T
T	A	0	T	G	C	C	G	C	0	C	G	G	2	6	T	C
T	C	0	T	G	C	C	C	C	0	T	G	G	2	4	C	T
T	A	1	T	G	C	C	C	C	0	T	G	G	2	4	C	T
G	A	1	C	G	C	G	N	N	0	T	G	G	2	4	C	T
T	A	0	N	N	N	N	N	N	N	N	N	N	N	N	N	N
G	A	1	T	G	C	C	C	C	0	T	G	G	2	4	C	T
T	C	0	T	G	C	G	C	C	0	T	G	G	2	4	C	T
T	A	0	T	A	C	C	C	C	0	C	G	G	2	6	T	T
T	A	0	T	G	C	C	C	C	0	C	G	G	2	6	T	T
G	A	1	T	G	A	G	C	C	0	C	G	G	2	6	T	T
G	A	1	C	G	C	G	C	C	0	T	G	G	2	4	C	T
T	C	0	C	A	C	C	G	T	8	T	G	C	2	4	C	T
G	A	0	C	A	C	C	G	T	0	T	G	G	2	4	C	T
G	A	1	N	N	N	N	N	N	0	T	C	G	2	4	C	T
T	A	0	T	A	C	C	G	T	8	T	G	C	2	4	C	T
G	A	0	T	A	C	C	G	T	0	C	G	G	2	6	T	T
T	A	0	C	G	C	G	G	T	0	T	G	C	2	4	T	C
G	A	1	N	N	N	N	N	N	N	N	N	N	N	N	N	N
T	C	0	T	A	C	C	G	T	0	T	G	G	2	4	C	T
T	A	0	T	G	A	G	C	C	N	T	C	G	2	6	T	T
T	A	0	T	A	C	C	G	T	0	T	G	G	2	4	C	T
G	A	1	T	A	C	G	G	C	0	T	G	G	2	4	T	C
G	A	1	T	A	C	C	G	T	0	T	C	G	0	4	T	C
G	A	1	T	G	C	G	G	C	0	C	G	G	2	6	T	T
T	C	0	T	A	C	C	C	C	0	T	C	G	0	4	T	C
N	N	N	T	A	C	C	C	C	0	T	C	G	0	4	T	C
N	N	N	T	A	C	C	G	T	0	T	C	G	0	4	T	C
T	A	0	N	N	N	N	N	N	N	N	N	N	N	N	N	N
T	A	0	N	N	N	N	N	N	0	C	G	C	2	4	C	T
T	A	0	C	G	N	N	N	N	0	T	C	G	2	6	T	T
G	A	1	N	N	N	N	N	N	0	C	G	G	2	4	C	T
G	A	0	T	A	C	G	G	C	0	T	G	G	2	4	C	T

G	A	0	N	N	N	N	N	N	8	T	G	G	2	4	C	T
G	A	1	T	G	C	C	G	C	8	T	G	C	2	0	C	T
T	A	0	N	N	N	N	N	N	0	T	C	G	2	6	N	N
T	A	0	C	A	C	G	G	T	0	T	G	G	0	4	T	C
T	A	1	T	A	C	G	G	T	0	T	G	G	0	4	T	C
T	A	0	T	G	C	C	C	C	0	T	G	G	0	4	T	C
G	A	1	N	N	N	N	N	N	0	C	G	G	2	6	T	T
G	A	1	C	G	C	C	G	C	0	T	G	G	0	4	T	T
G	A	0	T	G	C	C	G	C	0	T	G	G	2	4	C	T
T	A	0	T	G	C	C	C	C	0	T	G	G	2	4	C	T
G	A	0	N	N	N	N	N	N	N	N	N	N	N	N	N	N
G	A	1	N	N	N	N	N	N	N	N	N	N	N	N	N	N
T	C	0	T	G	C	C	G	C	0	T	G	G	2	4	C	T
T	A	0	T	A	C	C	G	T	0	C	G	G	2	6	T	T
T	C	0	N	N	N	N	N	N	0	T	C	G	2	6	T	T
T	A	0	T	A	C	C	G	T	0	T	C	G	2	6	T	T
G	A	1	C	G	C	C	G	C	0	C	G	G	2	6	T	T
T	A	0	C	G	C	C	G	C	0	T	G	G	2	4	C	T
G	A	1	T	A	C	C	G	T	0	T	C	G	2	6	T	T
G	A	1	N	N	N	N	N	N	0	T	C	G	0	4	T	C
G	A	1	T	A	C	C	G	T	0	T	G	G	2	4	T	C
G	A	1	T	A	C	C	C	C	0	T	G	G	2	4	C	T
G	A	1	T	A	C	C	G	T	0	T	G	G	2	4	T	C
T	A	0	N	N	N	N	N	N	0	T	G	G	2	4	C	T
G	A	0	T	A	C	C	G	T	8	T	G	C	2	6	T	C
G	A	0	T	A	C	C	G	T	8	T	G	C	2	6	T	C
T	A	0	T	A	C	C	G	T	0	T	G	G	2	4	C	T
G	A	N	T	A	C	G	G	C	0	T	G	G	2	4	C	T
T	A	0	T	A	C	C	G	T	8	T	G	C	2	6	T	C
T	A	0	T	G	C	C	G	C	8	T	G	C	2	6	T	C
T	A	0	T	G	A	G	C	C	0	T	G	G	2	4	C	T
G	A	1	T	G	A	G	N	N	0	T	G	G	2	4	C	T
T	A	0	T	G	A	G	C	C	0	T	G	G	2	4	C	T
G	A	0	T	A	C	C	C	C	0	T	G	G	2	4	C	T
G	A	1	T	A	C	C	C	C	0	T	C	G	2	6	T	T
T	A	0	T	G	A	G	C	C	0	T	G	G	2	4	C	T
G	A	0	T	G	A	G	C	C	0	T	G	G	2	4	C	T
G	A	0	T	G	A	G	C	C	0	T	G	G	2	4	C	T
T	A	0	N	N	N	N	N	N	0	T	C	G	2	6	T	T
G	A	1	N	N	N	N	N	N	0	T	G	C	2	6	T	C
T	C	0	C	A	C	C	C	C	8	T	G	C	2	6	T	C
G	A	0	T	A	C	G	G	C	0	T	G	G	2	4	C	T
G	A	N	N	N	N	N	N	N	N	N	N	N	N	N	N	N
G	A	1	N	N	N	N	N	N	N	N	N	N	N	N	N	N
G	A	1	T	A	C	C	G	T	8	T	G	C	2	6	T	C
T	A	0	T	A	N	N	N	N	0	T	G	G	2	4	C	T
G	A	1	N	N	N	N	N	N	0	T	G	C	2	6	T	C
G	A	1	T	A	C	C	G	T	0	T	G	C	2	6	T	C
G	A	1	N	N	N	N	N	N	8	T	G	G	0	4	C	T
T	C	0	C	A	C	C	G	T	0	T	G	G	2	4	C	T
G	A	1	C	G	C	C	G	C	N	N	N	N	N	N	N	N
T	A	0	T	G	C	G	G	C	N	N	N	N	N	N	N	N
G	A	1	T	A	C	C	G	T	0	T	G	G	2	4	C	T
G	A	1	T	A	C	C	G	T	0	T	G	G	2	4	C	T
G	A	0	C	G	A	C	C	C	0	T	G	G	2	4	C	T
T	A	0	C	G	C	C	G	C	0	T	G	G	2	4	C	T
T	A	0	T	G	C	N	N	N	0	T	G	G	0	4	T	T

T	A	0	T	G	C	C	C	C	0	T	G	G	2	4	C	T
T	A	0	T	A	C	G	G	T	0	C	G	G	2	6	T	T
G	A	0	T	A	C	G	G	T	0	T	C	G	0	4	T	C
G	A	1	T	G	C	C	G	C	0	T	G	G	2	4	C	T
G	A	0	C	A	C	C	C	C	0	T	G	G	2	4	T	C
N	N	N	T	A	C	G	G	C	0	T	G	G	0	4	T	C
G	A	1	C	G	C	G	N	N	0	T	G	G	2	4	C	T
T	A	0	T	A	C	G	G	T	0	C	G	G	2	6	T	T
T	A	0	T	A	C	G	G	C	0	C	G	G	2	6	T	T
T	C	0	N	N	N	N	N	N	0	T	G	G	0	4	T	T
T	A	0	C	G	C	C	G	C	0	T	C	G	0	4	T	C
G	A	0	T	G	C	C	G	T	0	T	G	G	0	4	T	T
G	A	1	C	G	C	C	G	C	0	C	G	G	2	6	T	T
T	A	1	T	G	A	G	C	C	0	T	C	G	0	4	T	C
G	A	0	T	G	A	G	C	C	0	T	G	G	2	4	C	T
G	A	0	T	G	A	G	C	C	0	C	G	G	2	6	T	T
G	A	1	T	G	C	N	N	N	0	T	G	G	2	4	C	T
G	A	0	T	A	C	C	G	T	0	T	C	C	2	4	C	T
G	A	0	T	G	C	G	G	C	0	T	C	G	2	6	T	T
T	A	0	N	N	N	N	N	N	0	T	C	G	0	4	T	C
G	A	1	T	G	C	C	G	T	0	T	C	C	2	4	N	N
G	A	0	N	N	N	N	N	N	0	T	C	G	0	4	T	C
T	C	0	T	G	C	C	C	C	0	T	G	G	2	4	C	T
T	C	0	T	G	C	C	C	C	0	T	C	G	0	4	T	C
T	A	0	T	G	C	C	G	T	0	C	G	G	2	6	T	T
T	C	0	N	N	N	N	N	N	0	T	G	G	2	4	C	T
T	A	0	T	G	C	G	G	N	0	T	C	G	0	4	T	C
T	A	0	N	N	N	N	N	N	0	T	G	G	0	4	T	T
G	A	0	T	A	C	C	N	N	0	T	G	G	2	4	C	T
T	A	0	T	A	C	C	G	T	0	T	G	G	2	6	N	N
G	A	0	N	N	N	N	N	N	0	T	G	G	0	4	T	C
T	A	0	N	N	N	N	N	N	0	T	G	C	2	4	T	C
N	C	1	T	G	C	C	G	C	0	T	G	G	2	4	C	T
T	A	0	T	G	C	C	G	C	8	T	G	C	2	4	C	T
T	A	0	T	G	C	G	G	C	0	T	C	G	2	6	T	T
G	A	1	N	N	N	N	N	N	0	T	G	G	0	4	T	T
T	A	0	C	A	C	C	G	T	0	T	C	G	0	4	T	C
T	A	0	T	A	C	C	G	T	0	T	C	G	2	6	T	T
T	A	0	N	N	N	N	N	N	0	T	C	G	0	4	T	C
T	A	0	C	G	C	C	G	C	N	N	N	N	N	N	N	N
G	A	0	C	G	C	C	G	C	0	T	C	G	0	4	T	C
N	N	N	T	A	C	G	G	T	0	T	G	G	0	4	T	T
T	A	0	T	A	C	C	G	T	0	T	C	G	0	4	T	C
T	A	0	T	A	C	C	G	T	0	T	C	G	2	6	T	T
G	A	1	C	G	C	C	G	C	0	T	C	G	2	6	T	T
T	A	0	C	G	C	C	G	C	0	T	C	G	0	4	T	C
T	A	0	C	G	A	C	C	C	0	T	C	G	0	4	T	C
T	C	0	C	G	C	C	G	C	0	C	C	G	0	4	T	C
T	A	0	T	A	C	G	G	T	0	T	C	G	2	6	T	T
T	C	0	T	G	C	C	C	C	0	T	C	G	2	6	T	T
G	A	0	T	G	C	C	C	C	0	T	C	G	2	6	T	T
N	N	N	T	G	C	C	C	C	0	T	C	G	2	6	T	T
G	A	1	C	G	C	C	G	C	0	T	C	G	0	4	T	C
T	C	0	T	G	C	C	C	C	0	T	C	G	0	4	T	C
T	A	0	C	G	C	C	N	N	0	T	C	G	0	4	T	C
N	N	N	N	N	N	N	N	N	0	T	C	G	2	6	T	T

ME9	ME10	ME11	ME12	P13K1	P13K2	P13K3	P13K4	P13K5	P13K6	P13K7	P13K8	P13K9
A	T	C	3	N	N	N	N	N	N	N	N	N
A	T	C	3	N	N	N	N	N	N	N	N	N
N	N	N	N	N	N	N	N	N	N	N	N	N
N	N	N	N	G	A	G	G	C	A	A	G	A
G	C	T	1	N	N	N	N	N	N	N	N	N
N	N	N	N	A	A	G	G	T	A	A	G	A
G	C	T	2	A	G	A	G	C	G	C	A	A
N	N	N	N	A	G	A	G	C	G	C	A	A
N	N	N	N	A	A	G	G	C	A	A	G	A
G	C	C	3	N	N	N	N	N	N	N	N	N
A	T	C	3	A	A	G	G	C	A	A	G	A
A	T	C	3	A	A	G	G	C	A	A	G	A
A	T	C	3	A	A	G	G	C	A	A	G	A
A	T	C	3	A	A	G	G	C	A	A	G	A
G	C	T	2	A	A	G	G	T	A	A	G	A
A	T	C	3	A	G	A	G	C	G	A	A	A
G	C	T	2	A	G	A	G	C	G	A	A	A
G	C	T	2	A	G	A	G	C	G	A	A	A
G	C	C	3	A	A	G	G	C	A	A	G	A
G	C	C	3	A	G	A	G	C	G	C	A	A
A	T	C	3	A	G	A	G	C	G	A	A	A
N	N	N	N	N	N	N	N	N	N	N	N	N
G	C	C	3	A	G	A	G	C	G	A	A	A
G	C	C	3	A	A	G	G	C	A	A	G	A
G	C	T	1	A	A	G	G	T	A	A	G	A
G	C	T	2	A	G	A	G	C	G	A	A	A
G	C	C	3	A	A	G	G	T	A	A	G	A
G	C	T	2	A	A	G	G	T	A	A	G	A
G	C	T	2	N	N	N	N	N	N	N	N	N
G	C	T	2	A	G	A	G	C	G	C	A	A
G	C	T	1	A	G	A	G	C	G	C	A	A
G	C	T	2	A	G	A	G	C	G	A	A	A
A	T	C	3	G	A	G	G	C	A	A	G	A
A	T	C	3	N	N	N	N	N	N	N	N	N
G	C	T	2	A	A	G	G	T	A	A	G	A
G	C	T	2	A	G	A	G	C	G	A	A	A
N	N	N	N	A	A	G	G	C	A	A	G	A
G	C	T	1	A	A	G	G	C	A	A	G	A
G	C	T	2	A	G	A	G	C	G	A	A	A
G	C	T	2	A	G	A	G	C	G	C	A	A
G	C	T	2	A	G	A	G	C	G	A	A	A
G	C	T	2	A	G	A	G	C	G	A	A	A
G	C	C	3	A	G	A	G	C	G	A	A	A
G	C	T	2	A	A	G	G	T	A	A	G	A
A	T	C	3	A	G	A	G	C	G	C	A	A
G	C	T	2	A	G	A	G	C	G	C	A	A
A	T	C	3	G	A	G	G	C	A	A	G	A
G	C	T	1	G	A	G	A	C	G	A	A	G
A	T	C	3	A	A	G	A	C	G	A	A	G
G	C	C	3	A	A	G	A	C	G	A	A	G
A	T	C	3	A	A	G	G	C	A	A	G	A
A	T	C	3	N	N	N	N	N	N	N	N	N
G	C	C	3	A	A	G	G	C	A	A	G	A
A	T	C	3	N	N	N	N	N	N	N	N	N
G	C	C	3	N	N	N	N	N	N	N	N	N
N	N	N	N	N	N	N	N	N	N	N	N	N

A	T	C	3	G	A	G	A	C	G	A	A	G
G	C	C	3	A	A	G	G	T	A	A	G	A
A	T	C	3	A	A	G	G	T	A	A	G	A
N	N	N	N	G	A	G	G	C	A	A	G	A
A	T	C	3	A	A	G	G	C	A	A	G	A
G	C	C	3	G	A	G	G	C	A	A	G	A
G	C	C	3	A	A	G	G	C	A	A	G	A
G	C	T	2	A	A	G	G	T	A	A	G	A
G	C	T	2	A	A	G	G	T	A	A	G	A
A	T	C	3	G	A	A	G	C	G	A	A	G
N	N	N	N	A	A	G	G	T	A	A	G	A
G	C	C	3	A	A	G	G	C	A	A	G	A
N	N	N	N	G	A	G	A	C	G	A	A	G
G	C	T	2	A	G	A	G	C	G	C	A	A
N	N	N	N	N	N	N	N	N	N	N	N	N
G	C	C	3	A	A	G	G	C	A	A	G	A
G	C	T	1	A	A	G	G	T	A	A	G	A
G	C	C	3	A	A	G	G	C	A	A	G	A
G	C	T	2	G	A	G	A	C	G	A	A	G
N	N	N	N	A	A	G	A	C	A	A	G	A
G	C	T	0	A	A	G	G	T	A	A	G	A

G	C	T	1	G	A	G	A	C	G	A	A	G
G	C	C	3	A	A	G	G	C	A	A	G	A
G	C	T	1	A	A	G	G	T	A	A	G	A
G	C	C	3	A	G	A	G	C	G	C	A	A
G	C	C	3	G	A	G	A	C	G	A	A	G
G	C	T	1	G	A	G	A	C	G	A	A	G
N	N	N	N	G	A	G	A	C	G	A	A	G
G	C	C	3	A	A	G	G	C	A	A	G	A
G	C	T	2	A	A	G	G	T	A	A	G	A
G	C	T	2	A	G	A	G	C	G	C	A	A
G	C	C	3	A	G	A	G	C	G	C	A	A
N	N	N	N	G	A	G	A	C	G	A	A	G
G	C	C	3	N	N	N	N	N	N	N	N	N
G	C	T	1	A	A	G	G	C	A	A	G	A
G	C	C	3	A	A	G	G	C	A	A	G	A
G	C	T	1	A	A	G	G	C	A	A	G	A
N	N	N	N	A	A	G	G	C	A	A	G	A
G	C	T	0	A	A	G	G	C	A	A	G	A
G	C	C	3	A	A	G	G	C	A	A	G	A
N	N	N	N	A	A	G	G	C	A	A	G	A
G	C	C	3	G	A	G	G	C	A	A	N	N
G	C	C	3	A	A	G	G	C	A	A	G	A
G	C	C	3	N	N	N	N	N	N	N	N	N
G	C	T	1	A	G	A	G	C	G	C	A	A
G	C	T	1	A	A	G	G	C	A	A	G	A
G	C	C	3	A	A	G	G	C	A	A	G	A
G	C	C	3	A	G	A	G	C	G	A	A	A
G	C	T	1	A	G	A	G	C	G	C	A	A
G	C	C	3	A	G	A	G	C	G	C	A	A
A	T	C	3	A	A	G	G	T	A	A	G	A
G	C	C	3	A	G	A	G	C	G	C	A	A
G	C	C	3	A	G	A	G	C	G	A	A	A
A	T	C	3	G	A	G	G	C	A	A	G	A
A	T	C	3	A	A	G	G	T	A	A	G	A
A	T	C	3	A	A	G	G	T	A	A	G	A

G	C	C	3	N	N	N	N	N	N	N	N	N
N	N	N	N	N	N	N	N	N	N	N	N	N
G	C	T	2	A	G	A	G	C	G	C	A	A
G	C	T	1	A	G	A	G	C	G	C	A	A
G	C	C	3	G	A	G	G	C	A	A	G	A
G	C	C	3	G	A	G	G	C	A	A	G	A
G	C	T	1	A	G	A	G	C	G	C	A	A
A	T	C	3	A	G	A	G	C	G	C	A	A
G	C	T	1	A	G	A	G	C	G	C	A	A
N	N	N	N	N	N	N	N	N	N	N	N	N
G	C	T	1	A	A	G	G	C	A	A	G	A
N	N	N	N	A	A	G	G	T	A	A	G	A
G	C	C	3	A	A	G	G	C	A	A	G	A
A	T	C	3	A	A	G	G	C	A	A	G	A
G	C	T	2	A	A	G	G	C	A	A	G	A
A	T	C	3	G	A	G	G	C	A	A	G	A
G	C	C	3	A	A	G	G	T	A	A	G	A
N	N	N	N	A	A	G	G	C	A	A	G	A
G	C	C	3	A	G	A	G	C	G	C	A	A
N	N	N	N	A	A	G	G	T	A	A	G	A
G	C	T	1	A	A	G	G	C	A	A	G	A
G	C	T	1	A	A	G	G	C	A	A	G	A
G	C	C	3	A	G	A	G	C	G	C	A	A
G	C	T	1	G	A	G	G	C	A	A	G	A
G	C	T	1	G	A	G	G	C	A	A	G	A
G	C	C	3	G	A	G	A	C	G	A	A	G
G	C	T	1	A	G	A	G	C	G	C	A	A
G	C	T	1	N	N	N	N	N	N	N	N	N
G	C	T	1	G	A	G	G	C	A	A	G	A
N	N	N	N	G	A	G	G	C	A	A	G	A
G	C	T	1	G	A	G	G	C	A	A	G	A
A	T	C	3	A	A	G	G	T	A	A	G	A
N	N	N	N	G	A	G	G	C	A	A	G	A
A	T	C	3	A	A	G	G	C	A	A	G	A
A	T	C	3	G	A	G	G	C	A	A	G	A
G	C	T	1	A	G	A	G	C	G	C	A	A
G	C	T	0	A	A	G	G	C	A	A	G	A
G	C	T	1	G	A	G	G	C	A	A	G	A
G	C	T	1	G	A	G	G	C	A	A	G	A
G	C	T	0	A	A	G	G	C	A	A	G	A
A	T	C	3	A	A	G	G	C	A	A	G	A
G	C	C	3	A	A	G	G	C	A	A	G	A
N	N	N	N	N	N	N	N	N	N	N	N	N
G	C	T	1	G	A	G	A	C	G	A	A	G
A	T	C	3	A	G	A	G	C	G	A	A	N
G	C	T	1	A	G	A	G	C	G	C	A	A
G	C	T	1	A	A	G	G	C	A	A	G	A
G	C	C	3	G	A	G	G	C	A	A	G	A
A	T	C	3	G	A	G	G	C	A	A	G	A
G	C	C	3	A	A	G	G	T	A	A	G	A
G	C	C	3	N	N	N	N	N	N	N	N	N
G	C	C	3	A	A	G	G	C	A	A	G	A
N	N	N	N	G	A	G	G	C	A	A	G	A
G	C	C	3	A	A	G	G	C	A	A	G	A
A	T	C	3	A	A	G	G	T	A	A	G	A
G	C	T	1	A	G	A	G	C	G	C	A	A
A	T	C	3	A	A	G	G	C	A	A	G	A

G	C	T	2	A	A	G	G	T	A	A	G	A
G	C	T	2	A	A	G	G	C	A	A	G	A
N	N	N	N	N	N	N	N	N	N	N	N	N
G	C	C	3	A	G	A	G	C	G	C	A	A
G	C	C	3	A	G	A	G	C	G	A	A	A
G	C	C	3	A	A	G	G	T	A	A	G	A
A	T	C	3	A	G	A	G	C	G	C	A	A
A	T	C	3	A	A	G	G	C	A	A	G	A
G	C	T	2	A	G	A	G	C	G	A	A	A
A	T	C	3	A	A	G	G	C	A	A	G	A
N	N	N	N	A	A	G	G	T	A	A	G	A
N	N	N	N	A	G	A	G	C	G	A	A	A
G	C	T	1	A	A	G	G	T	A	A	G	A
A	T	C	3	A	A	G	G	C	A	A	G	A
A	T	C	3	A	A	G	G	C	A	A	G	A
A	T	C	3	G	A	G	G	C	A	A	G	A
A	T	C	3	A	A	G	G	C	A	A	G	A
G	C	T	2	A	A	G	G	T	A	A	G	A
A	T	C	3	A	A	G	A	C	G	A	A	G
G	C	C	3	A	A	G	G	C	A	A	G	A
G	C	T	1	A	A	G	A	C	G	A	A	G
G	C	T	1	A	A	G	G	C	A	A	G	A
G	C	T	1	A	A	G	A	C	G	A	A	G
G	C	T	2	A	A	G	G	T	A	A	G	A
G	T	C	3	G	A	G	G	C	A	A	G	A
G	T	C	3	G	A	G	G	C	A	A	G	A
G	C	T	2	G	A	G	G	C	A	A	G	A
G	C	T	1	G	A	G	G	C	A	A	G	A
G	T	C	3	G	A	G	A	C	G	A	A	G
G	T	C	3	G	A	G	A	C	G	A	A	G
G	C	T	2	A	A	G	G	T	A	A	G	A
G	C	T	2	A	G	A	G	C	G	A	A	A
G	C	T	2	A	A	G	G	T	A	A	G	A
G	C	T	1	A	A	G	G	C	A	A	G	A
A	T	C	3	A	A	G	G	C	A	A	G	A
G	C	T	2	A	A	G	G	T	A	A	G	A
G	C	T	2	N	N	N	N	N	N	N	N	N
G	C	T	2	A	G	A	G	C	G	A	A	A
A	T	C	3	A	A	G	G	C	A	A	G	A
G	C	C	3	G	A	G	G	C	A	A	G	A
G	T	C	3	G	A	G	G	C	A	A	G	A
G	C	T	1	G	A	G	G	C	A	A	G	A
N	N	N	N	A	A	G	A	C	G	A	A	G
N	N	N	N	N	N	N	N	N	N	N	N	N
G	T	C	3	G	A	G	G	C	A	A	G	A
G	C	T	2	N	N	N	N	N	N	N	N	N
G	C	C	3	G	A	G	A	C	G	A	A	G
G	C	C	3	G	A	G	G	C	A	A	G	A
G	C	T	1	G	A	G	G	C	A	A	N	N
G	C	T	2	A	A	G	G	C	A	A	G	A
N	N	N	N	A	A	G	G	C	A	A	G	A
N	N	N	N	A	A	G	A	C	G	A	A	G
G	C	T	1	A	A	G	A	C	G	A	A	G
G	C	T	1	A	A	G	A	C	G	A	A	G
A	T	C	3	A	A	G	A	C	G	A	A	G
G	C	T	2	A	A	G	G	T	A	A	G	A
A	T	C	3	G	A	G	G	C	A	A	G	A

A	T	C	3	G	A	G	G	C	A	A	G	A
A	T	C	3	A	A	G	G	C	A	A	G	A
G	C	C	3	A	A	G	G	C	A	A	G	A
G	C	T	1	A	A	G	G	T	A	A	G	A
G	C	T	1	A	G	A	G	C	G	A	A	A
G	C	C	3	A	A	G	G	T	A	A	G	A
G	C	T	1	G	A	G	G	C	A	A	G	A
A	T	C	3	G	A	G	G	C	A	A	G	A
A	T	C	3	A	A	G	G	C	A	A	G	A
A	T	C	3	G	A	G	A	C	G	A	A	G
G	C	C	3	A	A	G	G	C	A	A	G	A
A	T	C	3	A	A	G	G	T	A	A	G	A
A	T	C	3	A	A	G	G	C	A	A	G	A
G	C	C	3	A	A	G	G	T	A	A	G	A
G	C	T	2	A	G	A	G	C	G	A	A	A
A	T	C	3	A	A	G	G	T	A	A	G	A
G	C	T	1	A	G	A	G	C	G	C	A	A
G	C	T	1	A	A	G	G	C	A	A	G	A
A	T	C	3	N	N	N	N	N	N	N	N	N
G	C	C	3	A	A	G	G	C	A	A	G	A
N	N	N	N	A	A	G	G	C	A	A	G	A
G	C	C	3	A	A	G	G	T	A	A	G	A
G	C	T	1	A	G	A	G	C	G	C	A	A
G	C	C	3	A	G	A	G	C	G	C	A	A
A	T	C	3	A	A	G	G	C	A	A	G	A
N	N	N	N	G	A	G	G	C	A	A	G	A
G	C	C	3	A	A	G	G	T	A	A	G	A
G	C	T	1	G	A	G	A	C	G	A	A	G
G	C	T	N	G	A	G	A	C	G	A	A	G
N	N	N	N	G	A	G	G	C	A	A	G	A
G	C	C	3	G	A	G	A	C	G	A	A	G
G	C	N	N	A	A	G	G	C	A	A	G	A
G	C	C	3	G	A	G	G	C	A	A	G	A
G	C	T	0	A	A	G	G	C	A	A	G	A
A	T	C	3	A	A	G	G	T	A	A	G	A
A	T	C	3	A	G	A	G	C	G	C	A	A
G	C	C	3	A	A	G	G	T	A	A	G	A
A	T	C	3	A	A	G	A	C	G	A	A	G
G	C	C	3	A	A	G	G	T	A	A	G	A
N	N	N	N	A	A	G	G	T	A	A	G	A
G	C	C	3	A	A	G	G	T	A	A	G	A
G	C	T	1	A	A	G	G	C	A	A	G	A
G	C	C	3	A	A	G	G	T	A	A	G	A
A	T	C	3	A	A	G	A	C	G	A	A	G
A	T	C	3	G	A	G	G	C	A	A	G	A
G	C	C	3	A	A	G	G	T	A	A	G	A
G	C	C	3	G	A	G	G	C	A	A	G	A
G	C	C	3	G	A	G	A	C	G	A	A	G
A	T	C	3	A	A	G	G	C	A	A	G	A
A	T	C	3	G	A	G	A	C	G	A	A	N
A	T	C	3	A	A	G	G	T	A	A	G	A
A	T	C	3	N	N	N	N	N	N	N	N	N
G	C	C	3	A	A	G	G	C	A	A	G	A
G	C	C	3	N	N	N	N	N	N	N	N	N
G	C	C	3	A	A	G	G	C	A	A	G	A
A	T	C	3	N	N	N	N	N	N	N	N	N

P13K10	PME1	PME2	PME3	SAH1	SAH2	SAH3	SAH4	SAH5	SAH6
N	N	N	N	C	C	T	T	G	N
N	N	N	N	C	C	T	T	G	N
N	N	N	N	C	C	C	C	C	N
T	A	A	C	C	C	C	C	C	G
N	A	A	C	C	C	C	C	T	G
T	A	A	C	C	C	C	C	C	N
T	A	A	C	N	N	N	N	N	N
T	A	G	C	C	C	C	C	C	A
T	A	A	C	N	N	N	N	N	A
N	N	N	N	C	C	C	C	N	N
T	A	G	C	C	C	C	C	T	A
T	A	A	C	C	C	C	C	T	N
T	A	G	C	C	C	T	T	G	A
N	G	A	T	C	C	C	C	T	A
T	A	A	C	G	T	C	C	T	N
T	A	A	C	N	N	N	N	N	N
T	A	A	C	N	N	N	N	N	N
T	A	A	C	C	T	C	C	G	N
T	A	G	C	N	N	N	N	N	N
N	G	A	T	C	C	C	C	C	N
T	A	A	C	N	N	N	N	N	N
N	G	A	T	C	C	C	C	T	A
N	G	A	T	C	C	C	C	T	A
T	G	A	T	C	C	C	C	C	A
T	A	A	C	C	C	T	T	G	G
T	A	G	C	C	C	C	C	C	A
T	G	A	T	C	C	T	T	G	A
T	A	A	C	N	N	N	N	N	A
N	A	A	C	C	C	C	C	T	A
T	A	A	C	C	C	C	C	T	A
T	A	A	C	C	C	C	C	C	N
T	A	A	C	N	N	N	N	N	N
N	A	G	C	C	C	C	C	C	N
N	N	N	N	C	C	T	T	G	N
T	A	A	C	C	C	C	C	T	A
N	A	G	C	G	T	C	C	T	N
N	A	A	C	C	C	C	C	T	N
T	A	A	C	G	T	C	C	T	N
N	A	A	C	G	T	C	C	T	N
T	A	A	C	N	N	N	N	N	N
T	A	A	C	C	C	C	C	T	G
T	A	A	C	C	C	C	C	T	A
T	A	A	C	C	C	T	T	G	N
T	A	A	C	C	C	C	C	T	A
T	A	A	C	C	C	C	C	C	N
T	A	A	C	C	C	C	C	C	N
T	A	A	C	C	C	C	C	T	A
C	A	G	C	C	C	T	T	G	G
C	G	A	T	C	C	T	T	G	A
C	A	G	C	C	C	T	T	G	A
T	A	A	C	G	T	C	C	T	A
N	N	N	N	C	C	C	C	T	N
T	A	A	C	C	C	C	C	T	A
N	N	N	N	C	C	C	C	C	N
N	N	N	N	N	N	N	N	N	N
N	A	A	C	G	T	C	C	T	N

N	A	A	C	C	C	T	T	G	N
N	A	A	C	C	C	C	C	T	N
T	A	A	N	G	T	C	C	T	A
T	A	A	C	C	C	C	C	T	N
T	A	A	C	N	N	N	N	N	N
T	G	A	T	G	T	C	C	T	G
T	A	A	C	C	C	C	C	C	G
T	A	A	C	C	C	C	C	T	N
T	A	A	C	N	N	N	N	N	N
N	A	A	C	C	C	C	C	N	A
T	A	A	C	C	C	T	T	G	A
T	A	A	T	G	T	C	C	T	N
N	A	A	C	C	C	C	C	C	G
T	A	A	C	G	T	C	C	T	G
N	A	A	T	G	T	C	C	T	A
T	A	A	C	G	T	C	C	T	N
T	A	A	C	G	T	C	C	T	A
T	A	A	C	N	N	N	N	N	N
C	A	A	C	C	C	T	T	G	N
T	A	A	C	N	N	N	N	N	N
T	A	G	C	G	T	C	C	T	A

N	A	A	C	N	N	N	N	N	G
N	A	G	C	C	C	C	C	C	A
T	A	A	T	G	T	C	C	T	N
N	A	G	C	G	T	C	C	T	N
C	A	A	C	N	N	N	N	N	N
C	A	A	C	C	C	T	C	G	N
C	A	A	C	N	N	N	N	N	A
T	A	G	C	C	C	T	C	C	G
T	A	A	C	C	C	C	C	C	N
T	A	A	T	G	T	C	C	T	N
T	A	G	C	C	C	T	T	G	N
N	A	A	C	N	N	N	N	N	N
N	A	A	C	N	N	N	N	N	N
N	A	A	C	G	T	C	C	T	G
T	A	G	C	G	T	C	C	T	N
T	A	A	C	G	T	C	C	T	A
N	A	A	C	N	N	N	N	N	N
T	A	A	C	G	T	C	C	T	N
T	A	A	C	C	C	C	C	C	G
T	A	A	T	C	C	T	T	G	N
N	A	A	C	C	C	T	T	G	N
N	G	A	T	C	C	T	C	C	N
N	A	A	C	C	T	C	C	T	N
T	A	A	C	G	T	C	C	T	G
T	A	G	C	G	T	C	C	T	A
T	A	G	C	C	C	T	C	G	N
T	A	G	C	C	C	T	C	C	N
N	A	G	C	N	N	N	N	N	N
T	G	A	T	C	C	C	C	C	N
T	A	A	C	C	C	C	C	C	N
T	A	G	C	C	C	C	C	C	A
T	A	A	C	G	T	C	C	T	A
N	A	A	C	C	C	C	C	T	A
T	A	G	C	C	C	C	C	C	G
T	A	G	C	C	C	T	T	G	N

N	N	N	N	C	C	T	T	G	N
N	N	N	N	G	T	C	C	T	N
N	A	A	C	N	N	N	N	N	N
T	A	A	C	C	C	T	T	G	N
T	A	A	C	G	T	C	C	T	A
T	A	A	C	C	C	T	T	G	G
T	A	A	C	C	C	C	C	N	N
T	A	A	C	C	T	C	C	G	N
T	A	A	C	C	C	C	C	T	N
N	A	A	C	N	N	N	N	N	A
T	A	A	C	G	T	C	C	T	N
T	A	A	C	G	T	C	C	T	N
T	A	A	C	N	N	N	N	N	A
T	A	A	C	C	C	T	T	G	A
N	A	A	C	N	N	N	N	N	N
T	G	A	T	C	T	C	C	G	G
T	A	A	C	N	N	N	N	N	A
T	A	A	C	G	T	C	C	T	G
T	A	A	C	N	N	N	N	N	G
T	A	A	C	C	C	C	C	C	N
T	A	A	C	C	C	T	T	G	A
T	A	A	C	N	N	N	N	N	N
T	A	G	C	N	N	N	N	N	A
T	A	A	C	C	C	C	C	C	N
T	A	A	C	C	C	T	T	G	A
C	A	A	C	C	C	C	C	C	A
T	A	A	C	N	N	N	N	N	A
N	A	A	C	N	N	N	N	N	N
T	A	A	C	C	T	C	C	G	N
T	A	A	C	C	C	C	C	C	G
T	A	A	C	C	C	C	C	C	A
T	A	A	C	C	C	C	C	C	N
T	A	A	C	C	C	C	C	C	A
T	A	A	C	N	N	N	N	N	N
T	A	A	C	C	C	C	C	T	N
N	G	A	T	C	C	T	T	G	A
T	G	A	T	N	N	N	N	N	N
T	A	A	C	N	N	N	N	N	A
T	A	G	C	C	C	C	C	C	G
T	A	A	C	C	C	C	C	C	G
N	G	A	T	N	N	N	N	N	N
T	A	G	C	C	C	C	C	C	A
N	A	A	C	G	T	C	C	T	N
N	A	A	C	C	C	T	C	C	N
N	A	G	T	C	C	T	C	G	N
T	G	A	T	C	C	C	C	C	A
T	G	A	T	N	N	N	N	N	N
T	A	A	C	G	T	C	C	T	G
T	A	A	C	C	C	T	T	G	G
T	A	A	C	C	C	C	C	C	G
N	N	N	N	C	C	C	C	C	N
T	G	A	N	C	C	C	C	C	G
T	A	G	C	C	C	T	C	C	N
T	A	G	C	C	C	C	C	T	N
N	A	A	C	C	C	C	C	C	A
T	A	G	C	C	C	T	T	G	A
T	A	A	C	C	C	C	C	C	A

T	A	A	C	C	C	C	C	T	A
T	A	A	C	C	C	C	C	T	N
N	A	A	C	C	C	C	C	C	A
T	A	A	C	C	C	C	C	C	A
T	A	G	C	N	N	N	N	N	A
T	A	G	C	N	N	N	N	N	A
T	G	A	T	C	C	T	T	G	G
T	A	G	C	N	N	N	N	N	A
T	A	A	C	N	N	N	N	N	A
T	A	A	C	G	T	C	C	T	N
T	A	A	C	N	N	N	N	N	A
T	A	A	C	N	N	N	N	N	N
T	A	A	C	N	N	N	N	N	N
T	A	A	C	N	N	N	N	N	N
T	A	A	C	N	N	N	N	N	A
T	A	A	C	N	N	N	N	N	N
N	A	A	C	G	T	C	C	T	N
T	A	A	C	C	C	C	C	T	N
C	G	A	T	C	C	C	C	C	N
T	A	G	C	N	N	N	N	N	A
N	A	G	C	C	C	T	T	G	N
N	A	A	C	C	C	T	T	G	G
C	A	G	C	C	C	T	T	G	N
T	N	N	N	C	C	C	C	T	A
N	A	G	C	N	N	N	N	N	A
T	A	G	C	N	N	N	N	N	A
T	A	G	C	N	N	N	N	N	N
T	A	A	C	G	T	C	C	T	N
C	A	G	C	N	N	N	N	N	N
C	A	A	C	N	N	N	N	N	N
N	A	A	C	C	C	C	C	T	A
T	A	A	C	C	C	C	C	T	A
T	A	A	C	C	C	C	C	T	A
T	A	A	C	C	C	T	T	G	N
T	A	A	C	N	N	N	N	N	N
T	A	A	C	C	C	C	C	T	A
N	A	A	C	C	C	C	C	T	A
T	A	A	C	C	C	C	C	T	A
T	A	A	C	G	T	C	C	N	N
N	A	G	C	N	N	N	N	N	N
T	A	G	C	N	N	N	N	N	N
T	A	A	C	G	T	C	C	T	N
N	A	G	C	C	C	T	T	G	A
N	A	G	C	C	C	T	T	G	N
T	A	G	C	N	N	N	N	N	A
N	A	G	C	N	N	N	N	N	A
C	A	G	C	N	N	N	N	N	G
T	A	G	C	N	N	N	N	N	N
N	A	G	C	N	N	N	N	N	N
T	A	G	C	C	C	C	C	C	N
T	A	G	C	C	C	T	T	G	G
C	A	G	C	C	C	T	T	G	G
C	A	G	C	C	C	T	T	G	N
C	A	G	C	C	C	T	T	G	N
C	A	G	C	C	C	T	T	G	N
T	A	A	C	C	C	C	C	T	A
N	A	G	T	G	T	C	C	T	A

T	G	A	T	G	T	C	C	T	A
T	G	A	T	C	C	C	C	T	G
T	G	A	T	C	C	C	C	T	A
T	A	A	C	N	N	N	N	N	G
T	A	G	C	G	T	C	C	T	G
N	A	G	C	C	C	T	T	G	N
T	A	A	C	C	C	C	C	C	G
T	G	A	T	G	T	C	C	T	N
T	A	A	C	C	C	C	C	T	N
C	A	G	C	G	T	C	C	T	N
T	G	A	T	C	C	C	C	T	N
T	A	A	C	N	N	N	N	N	N
T	G	A	T	C	C	T	T	G	A
T	G	A	T	N	N	N	N	N	N
T	A	A	C	C	C	C	C	T	N
T	A	A	C	N	N	N	N	N	A
T	A	A	C	N	N	N	N	N	N
T	A	A	C	G	T	C	C	T	N
N	A	A	C	C	C	C	C	C	N
T	A	A	C	C	C	T	T	G	A
N	A	A	C	G	T	C	C	T	A
T	A	A	C	C	T	C	C	G	A
T	A	A	C	N	N	N	N	N	A
T	A	A	C	N	N	N	N	N	A
T	A	A	C	G	T	C	C	T	N
N	A	A	C	C	C	C	C	C	N
T	A	A	C	N	N	N	N	N	N
N	A	G	C	G	T	C	C	G	G
N	A	A	C	G	T	C	C	T	A
T	A	A	C	N	N	N	N	N	N
C	A	G	C	C	C	T	C	T	N
T	A	A	C	G	T	C	C	N	N
T	A	A	N	C	C	T	C	G	N
N	A	A	C	G	T	C	C	T	N
T	A	A	C	C	C	T	C	T	G
T	A	A	C	C	C	T	C	C	A
T	A	G	C	C	C	C	C	C	N
C	A	A	C	N	N	N	N	N	A
T	A	A	C	N	N	N	N	N	N
T	A	A	C	N	N	N	N	N	N
T	G	A	T	N	N	N	N	N	N
N	G	A	T	C	C	C	C	T	A
T	A	A	C	N	N	N	N	N	N
C	A	A	N	C	C	C	C	C	N
T	G	A	T	G	T	C	C	T	A
T	G	A	T	N	N	N	N	N	N
T	G	A	T	C	C	T	T	G	N
C	A	A	C	C	C	C	C	C	N
N	A	A	C	C	C	C	C	T	N
N	A	G	C	C	C	C	C	C	N
T	N	N	N	G	T	C	C	T	N
N	N	N	N	N	N	N	N	N	G
N	A	A	C	C	C	C	C	T	A
N	A	G	C	C	C	C	C	C	N
T	A	A	C	C	C	C	C	T	G
N	N	N	N	G	T	C	C	T	N
