# Supplementary material for: Association and Linkage Analysis of Aluminum Tolerance Genes in Maize
Source: PLoS One. 2010 Apr 1;5(4):e9958. doi: 10.1371/journal.pone.0009958 (PMC2848604; doi:10.1371/journal.pone.0009958)
Supplement: Table S3 — Mixed Linear Model (MLM) based association analysis. MLM analysis was used to evaluate the importance for each polymorphic site in every candidate Al tolerance gene for NRG. Al-stress and control growth conditions were evaluated separately. All results are reported here. (0.18 MB PDF) [file pone.0009958.s003.pdf]

Trait	SASSNP2	Locus	Site	df	F	p	Model df	Error df	Error MS	Rsq_model	Rsq_marker	SAS p value (Ns)	df
lsm5	SNP79	ABC1	0	1	1.3256	0.2509	3	213	60.8476	0.5746	0.0026		
lsm5	SNP88	ABC10	0	1	0.6356	0.4261	3	234	59.5144	0.567	0.0012		
lsm5	SNP89	ABC11	0	1	0.6356	0.4261	3	234	59.5167	0.567	0.0012		
lsm5	SNP90	ABC12	0	1	1.9338	0.1657	3	235	58.7687	0.5706	0.0035		
lsm5	SNP91	ABC13	0	1	0.6246	0.4301	3	233	60.0572	0.5647	0.0012		
lsm5	SNP92	ABC14	0	1	0.0638	0.8009	3	235	59.503	0.5653	1.18E-04		
lsm5	SNP93	ABC15	0	1	0.2987	0.5853	3	229	60.5418	0.5563	5.79E-04		
lsm5	SNP94	ABC16	0	1	1.35E-04	0.9907	3	225	62.077	0.5502	2.70E-07		
lsm5	SNP95	ABC17	0	1	1.2779	0.2595	3	225	61.7923	0.5522	0.0025		
lsm5	SNP96	ABC18	0	1	1.2779	0.2595	3	225	61.7952	0.5522	0.0025		
lsm5	SNP97	ABC19	0	2	0.0875	0.9163	4	224	63.1014	0.5448	3.56E-04		
lsm5	SNP80	ABC2	0	1	1.3256	0.2509	3	213	60.8497	0.5746	0.0026		
lsm5	SNP98	ABC20	0	1	0.0342	0.8535	3	225	62.2257	0.5491	6.85E-05		
lsm5	SNP99	ABC21	0	1	0.9649	0.327	3	222	61.2755	0.5534	0.0019		
lsm5	SNP81	ABC3	0	1	0.1491	0.6997	3	219	63.268	0.5526	3.05E-04		
lsm5	SNP82	ABC4	0	3	1.4132	0.2397	5	227	57.9116	0.586	0.0077		
lsm5	SNP83	ABC5	0	1	0.6107	0.4353	3	234	59.545	0.5668	0.0011		
lsm5	SNP84	ABC6	0	1	0.3047	0.5815	3	234	59.3933	0.5679	5.63E-04		
lsm5	SNP85	ABC7	0	1	0.6356	0.4261	3	234	59.5061	0.5671	0.0012		
lsm5	SNP86	ABC8	0	1	0.6356	0.4261	3	234	59.5091	0.5671	0.0012		
lsm5	SNP87	ABC9	0	1	0.6356	0.4261	3	234	59.5119	0.5671	0.0012		
lsm5	SNP101	AL1_1	0	1	6.5933	0.0113	3	143	54.766	0.6274	0.0172	0.0376	244
lsm5	SNP110	AL1_10	0	2	0.1511	0.8599	4	132	90.2718	0.4337	0.0013		
lsm5	SNP111	AL1_11	0	1	1.1542	0.2846	3	133	87.7751	0.4452	0.0048		
lsm5	SNP112	AL1_12	0	1	5.973	0.0156	3	167	72.4174	0.5043	0.0177	0.0324	244
lsm5	SNP113	AL1_13	0	1	5.3082	0.0225	3	167	73.6174	0.4961	0.016		
lsm5	SNP114	AL1_14	0	1	5.3083	0.0225	3	167	73.6205	0.4961	0.016		
lsm5	SNP115	AL1_15	0	1	4.9024	0.0282	3	167	73.5244	0.4967	0.0148		
lsm5	SNP116	AL1_16	0	1	5.3084	0.0225	3	167	73.624	0.496	0.016		
lsm5	SNP117	AL1_17	0	1	5.3085	0.0225	3	167	73.6267	0.496	0.016		
lsm5	SNP118	AL1_18	0	1	5.3086	0.0225	3	167	73.6291	0.496	0.016		
lsm5	SNP119	AL1_19	0	1	4.2306	0.0413	3	166	74.3825	0.4936	0.0129		
lsm5	SNP102	AL1_2	0	1	0.7013	0.4037	3	144	48.8741	0.6669	0.0016		
lsm5	SNP120	AL1_20	0	1	5.2876	0.0227	3	165	67.8737	0.5369	0.0148		
lsm5	SNP121	AL1_21	0	1	5.2876	0.0227	3	165	67.8708	0.5369	0.0148		
lsm5	SNP122	AL1_22	0	1	5.2875	0.0227	3	165	67.8681	0.5369	0.0148	0.0286	244
lsm5	SNP123	AL1_23	0	1	4.8873	0.0284	3	164	68.6551	0.5299	0.014		
lsm5	SNP124	AL1_24	0	1	4.8873	0.0284	3	164	68.6578	0.5299	0.014		
lsm5	SNP125	AL1_25	0	1	4.3943	0.0376	3	164	68.859	0.5285	0.0126		
lsm5	SNP126	AL1_26	0	1	3.9644	0.0481	3	163	68.6832	0.532	0.0114		
lsm5	SNP127	AL1_27	0	1	5.8148	0.017	3	161	68.8553	0.5327	0.0169		
lsm5	SNP128	AL1_28	0	1	3.7661	0.0541	3	160	70.571	0.5216	0.0113		
lsm5	SNP129	AL1_29	0	1	4.6373	0.0328	3	160	70.4024	0.5227	0.0138		
lsm5	SNP103	AL1_3	0	1	5.487	0.0205	3	147	51.8704	0.6417	0.0134		
lsm5	SNP130	AL1_30	0	1	5.338	0.0221	3	160	69.3788	0.5297	0.0157		
lsm5	SNP131	AL1_31	0	1	4.8249	0.0295	3	158	73.6628	0.502	0.0152		
lsm5	SNP104	AL1_4	0	1	0.0692	0.7928	3	154	47.1344	0.669	1.49E-04		
lsm5	SNP105	AL1_5	0	1	1.5961	0.2082	3	165	61.9905	0.5676	0.0042		
lsm5	SNP106	AL1_6	0	2	2.4995	0.0853	4	163	62.4207	0.5682	0.0132		
lsm5	SNP107	AL1_7	0	2	0.0295	0.9709	4	131	80.3196	0.4799	2.34E-04		
lsm5	SNP108	AL1_8	0	1	0.1129	0.7374	3	133	89.829	0.4322	4.82E-04		
lsm5	SNP109	AL1_9	0	1	0.6799	0.4111	3	133	86.6486	0.4523	0.0028		
lsm5	SNP100	AL16_1	0	1	?	?	3	115	?	?	?	0.908	244
lsm5	SNP132	AL2_1	0	1	0.7444	0.3896	3	150	71.6093	0.4274	0.0028		
lsm5	SNP141	AL2_10	0	1	3.42E-04	0.9853	3	169	63.9212	0.5085	9.94E-07		
lsm5	SNP142	AL2_11	0	1	0.6185	0.4326	3	177	60.225	0.5281	0.0016		
lsm5	SNP143	AL2_12	0	2	4.8001	0.0093	4	175	58.4743	0.5431	0.0251	0.0215	244
lsm5	SNP144	AL2_13	0	1	1.0858	0.2988	3	176	62.5136	0.5088	0.003		
lsm5	SNP145	AL2_14	0	1	0.9232	0.338	3	176	60.356	0.5257	0.0025		
lsm5	SNP146	AL2_15	0	1	0.6705	0.414	3	175	58.5668	0.5378	0.0018		
lsm5	SNP147	AL2_16	0	1	0.933	0.3354	3	175	57.7418	0.5443	0.0024		
lsm5	SNP148	AL2_17	0	1	0.4951	0.4826	3	169	55.3674	0.5551	0.0013		
lsm5	SNP133	AL2_2	0	1	10.2463	0.0016	3	163	56.3496	0.5769	0.0266	0.0446	244
lsm5	SNP134	AL2_3	0	1	1.1992	0.2751	3	164	69.0733	0.4781	0.0038		
lsm5	SNP135	AL2_4	0	1	0.1629	0.687	3	166	65.3434	0.5016	4.89E-04		
lsm5	SNP136	AL2_5	0	1	0.369	0.5444	3	166	67.328	0.4864	0.0011		
lsm5	SNP137	AL2_6	0	1	0.9284	0.3367	3	166	64.5212	0.5078	0.0028		
lsm5	SNP138	AL2_7	0	1	2.1429	0.1451	3	166	64.0527	0.5114	0.0063		
lsm5	SNP139	AL2_8	0	1	0.3688	0.5445	3	166	67.3172	0.4865	0.0011		
lsm5	SNP140	AL2_9	0	2	1.4494	0.2377	4	164	67.1047	0.4937	0.0089		
lsm5	SNP149	AL3_1	0	1	0.2007	0.6548	3	158	65.8531	0.5669	5.50E-04		
lsm5	SNP150	AL3_2	0	1	0.4221	0.5168	3	158	62.1201	0.5915	0.0011		
lsm5	SNP151	AL3_3	0	1	0.2209	0.639	3	157	63.1974	0.587	5.81E-04		
lsm5	SNP152	AL3_4	0	1	7.2841	0.0077	3	158	60.7926	0.6002	0.0184	0.0085	244
lsm5	SNP153	AL3_5	0	2	1.4359	0.241	4	157	66.7658	0.5636	0.008		
lsm5	SNP154	AL3_6	0	1	0.4534	0.5016	3	174	60.2697	0.6017	0.001		
lsm5	SNP155	AL3_7	0	1	0.137	0.7118	3	173	61.8396	0.5871	3.27E-04		
lsm5	SNP156	AL3_8	0	1	0.5003	0.4803	3	174	61.0198	0.5968	0.0012		
lsm5	SNP157	AL3_9	0	2	0.3867	0.6799	4	173	61.2735	0.5974	0.0018		
lsm5	SNP158	AL5_1	0	1	0.7869	0.3764	3	150	34.3407	0.7733	0.0012		
lsm5	SNP167	AL5_10	0	1	0.0561	0.813	3	188	48.7933	0.6612	1.01E-04		
lsm5	SNP168	AL5_11	0	1	0.2809	0.5968	3	192	45.4781	0.6798	4.68E-04		
lsm5	SNP169	AL5_12	0	1	0.2809	0.5967	3	192	45.476	0.6798	4.68E-04		
lsm5	SNP170	AL5_13	0	3	0.2099	0.8894	5	189	45.9831	0.6784	0.0011		
lsm5	SNP171	AL5_14	0	1	0.3563	0.5512	3	192	45.3036	0.681	5.92E-04		
lsm5	SNP172	AL5_15	0	3	1.0653	0.365	5	196	45.2979	0.6789	0.0052		
lsm5	SNP173	AL5_16	0	1	1.7682	0.1851	3	199	44.9475	0.6765	0.0029		
lsm5	SNP174	AL5_17	0	1	1.1399	0.287	3	199	46.1604	0.6678	0.0019		
lsm5	SNP175	AL5_18	0	1	1.3257	0.251	3	199	46.2182	0.6674	0.0022		
lsm5	SNP176	AL5_19	0	2	1.033	0.3578	4	198	45.4844	0.6743	0.0034		

Ism5	SNP159	AL5_2	0	1	0.9263	0.3373	3	153	38.2863	0.7541	0.0015		
Ism5	SNP177	AL5_20	0	1	0.1442	0.7045	3	199	46.176	0.6677	2.41E-04		
Ism5	SNP178	AL5_21	0	1	1.7673	0.1852	3	199	45.0287	0.6759	0.0029		
Ism5	SNP179	AL5_22	0	1	0.3369	0.5623	3	199	45.7658	0.6706	5.58E-04		
Ism5	SNP180	AL5_23	0	1	0.3348	0.5635	3	198	45.9968	0.6706	5.57E-04		
Ism5	SNP181	AL5_24	0	1	1.2022	0.2745	3	161	39.8887	0.7213	0.0021		
Ism5	SNP160	AL5_3	0	1	8.67E-05	0.9926	3	172	40.7157	0.7183	1.42E-07		
Ism5	SNP161	AL5_4	0	1	0.2617	0.6096	3	185	47.4053	0.669	4.68E-04		
Ism5	SNP162	AL5_5	0	1	0.0557	0.8137	3	185	47.7019	0.6669	1.00E-04		
Ism5	SNP163	AL5_6	0	1	0.0896	0.765	3	185	49.021	0.6582	1.66E-04		
Ism5	SNP164	AL5_7	0	1	0.4876	0.4859	3	185	49.2342	0.6567	9.05E-04		
Ism5	SNP165	AL5_8	0	1	0.2467	0.62	3	188	48.3121	0.6646	4.40E-04		
Ism5	SNP166	AL5_9	0	1	0.3566	0.5511	3	188	48.4857	0.6634	6.39E-04		
Ism5	SNP182	AL8_1	0	1	2.1505	0.1443	3	174	62.2118	0.5444	0.0056		
Ism5	SNP191	AL8_10	0	1	0.2691	0.6046	3	187	71.6032	0.468	7.65E-04		
Ism5	SNP192	AL8_11	0	1	1.2435	0.2663	3	182	75.1254	0.4597	0.0037		
Ism5	SNP193	AL8_12	0	1	0.8384	0.361	3	188	73.6113	0.458	0.0024		
Ism5	SNP194	AL8_13	0	1	0.8383	0.361	3	188	73.6137	0.458	0.0024		
Ism5	SNP195	AL8_14	0	1	1.2076	0.2732	3	187	71.3542	0.4786	0.0034		
Ism5	SNP196	AL8_15	0	2	0.5217	0.5944	4	188	74.7471	0.4522	0.003		
Ism5	SNP197	AL8_16	0	1	0.536	0.465	3	189	73.6024	0.4578	0.0015		
Ism5	SNP198	AL8_17	0	1	0.9081	0.3418	3	189	73.2372	0.4604	0.0026		
Ism5	SNP199	AL8_18	0	2	0.0553	0.9463	4	188	74.7662	0.4521	3.22E-04		
Ism5	SNP200	AL8_19	0	1	0.908	0.3419	3	189	73.2436	0.4604	0.0026		
Ism5	SNP183	AL8_2	0	1	0.3794	0.5389	3	154	79.0593	0.4581	0.0013		
Ism5	SNP201	AL8_20	0	1	0.908	0.3419	3	189	73.2417	0.4604	0.0026		
Ism5	SNP202	AL8_21	0	1	0.9181	0.3392	3	188	73.2142	0.4634	0.0026		
Ism5	SNP203	AL8_22	0	1	0.9085	0.3417	3	189	73.164	0.461	0.0026		
Ism5	SNP204	AL8_23	0	1	0.4501	0.5031	3	188	73.9218	0.4543	0.0013		
Ism5	SNP205	AL8_24	0	1	1.1823	0.2783	3	186	74.5099	0.4514	0.0035		
Ism5	SNP206	AL8_25	0	1	1.0644	0.3036	3	182	62.7657	0.5316	0.0027		
Ism5	SNP207	AL8_26	0	1	0.0363	0.8492	3	151	49.0603	0.6475	8.47E-05		
Ism5	SNP184	AL8_3	0	1	0.9582	0.329	3	178	68.7775	0.4981	0.0027		
Ism5	SNP185	AL8_4	0	1	0.9582	0.329	3	178	68.7755	0.4981	0.0027		
Ism5	SNP186	AL8_5	0	1	0.965	0.3273	3	177	69.9602	0.4888	0.0028		
Ism5	SNP187	AL8_6	0	1	0.9581	0.329	3	178	68.8132	0.4978	0.0027		
Ism5	SNP188	AL8_7	0	1	0.9684	0.3264	3	185	70.4466	0.4804	0.0027		
Ism5	SNP189	AL8_8	0	1	0.9684	0.3264	3	185	70.4488	0.4804	0.0027		
Ism5	SNP190	AL8_9	0	1	0.967	0.3267	3	187	70.7342	0.4744	0.0027		
Ism5	SNP24	Al9_1	0	1	0.065	0.7989	3	243	66.6711	0.5363	1.24E-04		
Ism5	SNP25	Al9_2	0	1	0.067	0.796	3	244	69.4448	0.5156	1.33E-04		
Ism5	SNP208	anti1	0	1	3.7295	0.0547	3	226	73.3622	0.4753	0.0087		
Ism5	SNP217	anti10	0	2	0.2053	0.8146	4	195	63.398	0.5585	9.30E-04		
Ism5	SNP218	anti11	0	1	0.3097	0.5785	3	195	51.0542	0.6292	5.89E-04		
Ism5	SNP219	anti12	0	1	0.3097	0.5785	3	195	51.0511	0.6292	5.89E-04		
Ism5	SNP220	anti13	0	1	0.0531	0.818	3	195	51.1172	0.6287	1.01E-04		
Ism5	SNP221	anti14	0	1	0.0807	0.7767	3	187	50.8865	0.6217	1.63E-04		
Ism5	SNP209	anti2	0	1	0.3594	0.5495	3	229	79.7618	0.4327	8.90E-04		
Ism5	SNP210	anti3	0	1	1.2712	0.2607	3	229	78.7088	0.4402	0.0031		
Ism5	SNP211	anti4	0	1	0.0301	0.8623	3	221	75.9858	0.4543	7.44E-05		
Ism5	SNP212	anti5	0	1	0.1794	0.6723	3	216	70.9696	0.5024	4.13E-04		
Ism5	SNP213	anti6	0	1	0.2502	0.6175	3	216	70.2803	0.5072	5.71E-04		
Ism5	SNP214	anti7	0	2	1.0763	0.3428	4	204	72.8999	0.4897	0.0054		
Ism5	SNP215	anti8	0	1	0.0146	0.9038	3	205	74.6827	0.4747	3.75E-05		
Ism5	SNP216	anti9	0	1	0.6905	0.407	3	195	62.271	0.565	0.0015		
Ism5	SNP26	ASL1	0	1	3.0134	0.0841	3	209	72.9141	0.4742	0.0076		
Ism5	SNP35	ASL10	0	1	1.7329	0.1893	3	234	73.6477	0.4761	0.0039		
Ism5	SNP36	ASL11	0	1	6.0274	0.0148	3	239	77.2337	0.4447	0.014	0.0117	244
Ism5	SNP37	ASL12	0	2	3.6939	0.0263	4	238	73.6163	0.473	0.0164	0.0409	244
Ism5	SNP38	ASL13	0	1	1.1654	0.2814	3	239	76.1525	0.4525	0.0027		
Ism5	SNP39	ASL14	0	1	7.265	0.0075	3	239	77.4648	0.4431	0.0169	0.0057	244
Ism5	SNP40	ASL15	0	2	2.9516	0.0542	4	238	77.1522	0.4477	0.0137	0.0475	243
Ism5	SNP41	ASL16	0	1	5.3381	0.0218	3	219	79.0606	0.4219	0.0141	0.0236	244
Ism5	SNP42	ASL17	0	1	6.0291	0.0148	3	239	77.2701	0.4445	0.014	0.0117	244
Ism5	SNP43	ASL18	0	1	6.029	0.0148	3	239	77.2688	0.4445	0.014	0.0117	244
Ism5	SNP44	ASL19	0	1	6.029	0.0148	3	239	77.2675	0.4445	0.014	0.0117	244
Ism5	SNP27	ASL2	0	1	0.4247	0.5153	3	211	75.2946	0.4588	0.0011		
Ism5	SNP45	ASL20	0	1	5.0279	0.0259	3	239	77.583	0.4422	0.0117	0.0178	244
Ism5	SNP46	ASL21	0	1	8.807	0.0033	3	239	76.7235	0.4484	0.0203	0.0028	244
Ism5	SNP47	ASL22	0	1	8.8072	0.0033	3	239	76.7257	0.4484	0.0203	0.0028	244
Ism5	SNP48	ASL23	0	1	8.8073	0.0033	3	239	76.7277	0.4484	0.0203	0.0028	244
Ism5	SNP49	ASL24	0	1	8.6248	0.0036	3	238	76.9948	0.4487	0.02	0.0029	244
Ism5	SNP50	ASL25	0	1	8.855	0.0032	3	238	71.7591	0.4862	0.0191	0.0086	244
Ism5	SNP51	ASL26	0	1	9.5791	0.0022	3	213	56.9474	0.592	0.0183	0.0009	244
Ism5	SNP52	ASL27	0	1	7.9905	0.0051	3	216	56.3211	0.5932	0.015	0.0021	244
Ism5	SNP53	ASL28	0	1	7.9904	0.0051	3	216	56.3185	0.5932	0.015	0.0021	244
Ism5	SNP54	ASL29	0	1	4.7596	0.0302	3	222	67.0453	0.5273	0.0101	0.0362	244
Ism5	SNP28	ASL3	0	1	3.4107	0.0661	3	224	76.9957	0.4431	0.0085		
Ism5	SNP55	ASL30	0	2	1.3654	0.2575	4	219	69.9188	0.5114	0.0061		
Ism5	SNP56	ASL31	0	1	6.0091	0.015	3	210	66.4507	0.5216	0.0137	0.016	244
Ism5	SNP57	ASL32	0	1	4.3749	0.0377	3	210	67.4615	0.5143	0.0101	0.0258	244
Ism5	SNP58	ASL33	0	1	8.35E-04	0.977	3	220	87.6664	0.3736	2.38E-06		
Ism5	SNP59	ASL34	0	1	0.347	0.5564	3	222	86.3072	0.3966	9.43E-04		
Ism5	SNP60	ASL35	0	1	0.5781	0.4479	3	224	85.3223	0.3953	0.0016		
Ism5	SNP61	ASL36	0	3	0.6552	0.5805	5	219	81.2007	0.4333	0.0051		
Ism5	SNP62	ASL37	0	2	1.6235	0.2	4	188	88.1646	0.3861	0.0106		
Ism5	SNP63	ASL38	0	3	1.246	0.2944	5	186	89.0664	0.3861	0.0123		
Ism5	SNP64	ASL39	0	2	1.2473	0.2893	4	217	84.0323	0.416	0.0067		
Ism5	SNP29	ASL4	0	1	0.2984	0.5854	3	234	73.3998	0.4779	6.66E-04		
Ism5	SNP65	ASL40	0	1	7.1174	0.0082	3	217	81.6767	0.4324	0.0186	0.0369	244
Ism5	SNP66	ASL41	0	1	0.334	0.5639	3	216	80.1306	0.442	8.63E-04		

lsm5	SNP67	ASL42	0	1	3.4175	0.0659	3	208	83.8793	0.4264	0.0094		
lsm5	SNP68	ASL43	0	1	3.0369	0.0829	3	207	85.2194	0.4181	0.0085		
lsm5	SNP69	ASL44	0	1	3.0369	0.0829	3	207	85.2213	0.418	0.0085		
lsm5	SNP70	ASL45	0	1	3.1243	0.0786	3	205	85.4838	0.4176	0.0089		
lsm5	SNP71	ASL46	0	1	1.9303	0.1663	3	196	92.4276	0.3795	0.0061		
lsm5	SNP72	ASL47	0	1	1.9304	0.1663	3	196	92.4299	0.3795	0.0061		
lsm5	SNP73	ASL48	0	1	1.408	0.2368	3	195	93.1059	0.3728	0.0045		
lsm5	SNP74	ASL49	0	1	1.408	0.2368	3	195	93.1087	0.3728	0.0045		
lsm5	SNP30	ASL5	0	1	3.0003	0.0846	3	235	74.905	0.4649	0.0068		
lsm5	SNP75	ASL50	0	1	1.4081	0.2368	3	195	93.1114	0.3727	0.0045		
lsm5	SNP76	ASL51	0	2	1.655	0.1938	4	193	97.0528	0.352	0.0111		
lsm5	SNP77	ASL52	0	1	1.0508	0.3067	3	174	96.3917	0.3719	0.0038		
lsm5	SNP78	ASL53	0	1	1.3458	0.2477	3	167	100.0129	0.3508	0.0052		
lsm5	SNP31	ASL6	0	1	4.0883	0.0443	3	234	76.3808	0.4562	0.0095		
lsm5	SNP32	ASL7	0	1	4.0883	0.0443	3	234	76.382	0.4562	0.0095		
lsm5	SNP33	ASL8	0	1	3.1252	0.0784	3	235	75.3144	0.462	0.0072		
lsm5	SNP34	ASL9	0	1	3.9967	0.0467	3	235	75.6896	0.4593	0.0092		
lsm5	SNP1	AUX01	0	1	0.0613	0.8047	3	181	81.5337	0.393	2.06E-04		
lsm5	SNP2	AUX02	0	1	0.0916	0.7625	3	189	79.6598	0.4026	2.90E-04		
lsm5	SNP3	AUX03	0	1	1.4741	0.2262	3	191	80.6143	0.3923	0.0047	0.5481	244
lsm5	SNP4	AUX04	0	1	0.0225	0.8809	3	192	80.3261	0.394	7.10E-05		
lsm5	SNP5	AUX05	0	2	0.8358	0.4351	4	194	81.0244	0.3979	0.0052		
lsm5	SNP6	AUX06	0	2	0.6669	0.5145	4	194	82.0763	0.3901	0.0042		
lsm5	SNP7	AUX07	0	1	3.0946	0.0801	3	193	85.8213	0.3599	0.0103		
lsm5	SNP8	AUX08	0	2	0.6918	0.502	4	185	85.2952	0.3785	0.0046		
lsm5	SNP9	AUX09	0	1	0.8718	0.3517	3	185	90.1564	0.3337	0.0031	0.7558	244
lsm5	SNP10	AUX10	0	2	0.2034	0.8162	4	184	90.3575	0.3358	0.0015		
lsm5	SNP11	AUX11	0	2	1.5853	0.2077	4	184	91.5332	0.3272	0.0116		
lsm5	SNP12	AUX12	0	1	1.1914	0.2765	3	185	89.6329	0.3376	0.0043		
lsm5	SNP13	AUX13	0	2	0.2965	0.7438	4	183	90.3685	0.3367	0.0021		
lsm5	SNP14	AUX14	0	2	0.0358	0.9648	4	183	90.4534	0.3361	2.60E-04		
lsm5	SNP15	AUX15	0	1	3.5297	0.0619	3	181	93.6841	0.3125	0.0134		
lsm5	SNP16	AUX16	0	2	0.2332	0.7922	4	177	88.5241	0.3442	0.0017		
lsm5	SNP17	AUX17	0	1	1.6436	0.2015	3	178	90.7632	0.3238	0.0062		
lsm5	SNP18	AUX18	0	1	0.0019	0.9657	3	177	87.7789	0.345	6.85E-06		
lsm5	SNP19	AUX19	0	1	0.1137	0.7364	3	174	89.0433	0.3322	4.36E-04		
lsm5	SNP20	AUX20	0	1	2.1381	0.1455	3	169	91.1517	0.3276	0.0085		
lsm5	SNP21	AUX21	0	1	0.1289	0.72	3	169	91.4184	0.3257	5.14E-04		
lsm5	SNP22	AUX22	0	2	0.9085	0.4051	4	164	94.0424	0.3239	0.0075		
lsm5	SNP23	AUX23	0	2	0.2296	0.7951	4	164	93.1876	0.3301	0.0019		
lsm5	SNP222	cp1	0	1	1.5938	0.2081	3	221	49.9212	0.6339	0.0026		
lsm5	SNP231	cp10	0	1	0.2338	0.6292	3	232	59.8789	0.5643	4.39E-04		
lsm5	SNP232	cp11	0	1	0.1629	0.6869	3	232	59.8953	0.5641	3.06E-04		
lsm5	SNP233	cp12	0	1	0.7403	0.3905	3	229	59.2437	0.5707	0.0014		
lsm5	SNP234	cp13	0	1	0.2119	0.6458	3	208	46.4331	0.6498	3.57E-04		
lsm5	SNP235	cp14	0	1	0.3486	0.5555	3	224	63.5108	0.5367	7.21E-04		
lsm5	SNP223	cp2	0	1	0.4954	0.4823	3	230	58.0502	0.5768	9.11E-04		
lsm5	SNP224	cp3	0	1	0.1692	0.6812	3	232	60.0725	0.5629	3.19E-04		
lsm5	SNP225	cp4	0	1	0.1129	0.7372	3	232	59.864	0.5644	2.12E-04		
lsm5	SNP226	cp5	0	2	0.2896	0.7488	4	231	59.6352	0.5679	0.0011		
lsm5	SNP227	cp6	0	1	0.0885	0.7664	3	232	59.75	0.5652	1.66E-04		
lsm5	SNP228	cp7	0	1	0.4548	0.5007	3	232	59.4659	0.5673	8.48E-04		
lsm5	SNP229	cp8	0	1	0.0958	0.7572	3	231	60.4818	0.5603	1.82E-04		
lsm5	SNP230	cp9	0	1	0.2338	0.6292	3	232	59.8809	0.5643	4.39E-04		
lsm5	SNP236	Fe1	0	1	1.4709	0.2266	3	207	81.605	0.4265	0.0041		
lsm5	SNP245	Fe10	0	1	1.1227	0.2907	3	183	73.2906	0.495	0.0031		
lsm5	SNP246	Fe11	0	1	0.7603	0.3844	3	183	73.4353	0.494	0.0021		
lsm5	SNP237	Fe2	0	1	0.1225	0.7267	3	222	88.5511	0.3862	3.39E-04		
lsm5	SNP238	Fe3	0	1	0.7729	0.3803	3	222	89.3334	0.3807	0.0022		
lsm5	SNP239	Fe4	0	1	0.847	0.3584	3	223	88.8497	0.3815	0.0023		
lsm5	SNP240	Fe5	0	1	1.5562	0.2135	3	220	87.9831	0.3827	0.0044		
lsm5	SNP241	Fe6	0	1	1.5274	0.2178	3	219	88.3791	0.3827	0.0043		
lsm5	SNP242	Fe7	0	1	0.435	0.5102	3	210	89.8157	0.3705	0.0013		
lsm5	SNP243	Fe8	0	1	0.0243	0.8763	3	198	84.5492	0.4132	7.20E-05		
lsm5	SNP244	Fe9	0	1	2.0706	0.1518	3	196	82.1679	0.4334	0.006		
lsm5	SNP247	Fum1	0	1	2.5985	0.1083	3	234	80.8447	0.4361	0.0063		
lsm5	SNP248	Fum2	0	2	2.1609	0.1174	4	249	76.469	0.4606	0.0094		
lsm5	SNP249	Fum3	0	1	3.745	0.0541	3	252	79.9286	0.4396	0.0083		
lsm5	SNP250	Fum4	0	1	4.8782	0.0281	3	251	78.2382	0.4456	0.0108		
lsm5	SNP251	Fum5	0	1	3.0818	0.0804	3	249	76.7216	0.455	0.0067		
lsm5	SNP252	Fum6	0	1	3.4248	0.0654	3	249	77.0739	0.4525	0.0075		
lsm5	SNP253	Fum7	0	1	5.8353	0.0164	3	249	76.5609	0.4561	0.0127		
lsm5	SNP254	Fum8	0	1	5.3545	0.0215	3	249	76.7853	0.4545	0.0117		
lsm5	SNP255	Ger1	0	1	4.829	0.0294	3	163	79.3176	0.4295	0.0169		
lsm5	SNP264	Ger10	0	1	3.2009	0.0754	3	166	78.9186	0.4245	0.0111		
lsm5	SNP265	Ger11	0	1	3.2009	0.0754	3	166	78.9209	0.4245	0.0111		
lsm5	SNP266	Ger12	0	1	3.2009	0.0754	3	166	78.923	0.4245	0.0111		
lsm5	SNP267	Ger13	0	1	3.2009	0.0754	3	166	78.9249	0.4244	0.0111		
lsm5	SNP268	Ger14	0	1	3.1489	0.0778	3	167	78.3111	0.4282	0.0108		
lsm5	SNP269	Ger15	0	1	3.1489	0.0778	3	167	78.3085	0.4283	0.0108		
lsm5	SNP270	Ger16	0	1	0.2129	0.6451	3	167	81.5369	0.4047	7.59E-04		
lsm5	SNP271	Ger17	0	1	0.3437	0.5585	3	167	82.0261	0.4011	0.0012		
lsm5	SNP272	Ger18	0	1	3.5976	0.0596	3	166	78.8093	0.4115	0.0128		
lsm5	SNP273	Ger19	0	1	3.4753	0.0641	3	165	79.4174	0.4071	0.0125		
lsm5	SNP256	Ger2	0	1	0.5696	0.4515	3	164	79.886	0.4219	0.002		
lsm5	SNP274	Ger20	0	1	1.1344	0.2884	3	165	76.874	0.4261	0.0039		
lsm5	SNP275	Ger21	0	1	3.5976	0.0596	3	166	78.8064	0.4115	0.0128		
lsm5	SNP276	Ger22	0	1	3.5976	0.0596	3	166	78.8084	0.4115	0.0128		
lsm5	SNP277	Ger23	0	1	3.5976	0.0596	3	166	78.8104	0.4114	0.0128		
lsm5	SNP278	Ger24	0	1	0.0993	0.7531	3	166	82.0959	0.3869	3.67E-04		
lsm5	SNP279	Ger25	0	1	2.8134	0.0954	3	164	79.361	0.41	0.0101		

Ism5	SNP280	Ger26	0	2	2.3062	0.1031	4	151	85.3361	0.3687	0.0193		
Ism5	SNP257	Ger3	0	1	3.1873	0.0761	3	164	81.8208	0.4079	0.0115		
Ism5	SNP258	Ger4	0	1	0.2207	0.6391	3	164	85.0708	0.3844	8.28E-04		
Ism5	SNP259	Ger5	0	1	0.8541	0.3567	3	165	77.6627	0.4369	0.0029		
Ism5	SNP260	Ger6	0	1	0.6245	0.4305	3	165	78.1353	0.4335	0.0021		
Ism5	SNP261	Ger7	0	1	3.1831	0.0762	3	165	79.9816	0.4201	0.0112		
Ism5	SNP262	Ger8	0	1	3.1831	0.0762	3	165	79.9837	0.4201	0.0112		
Ism5	SNP263	Ger9	0	1	0.6339	0.4271	3	166	77.0583	0.438	0.0021		
Ism5	SNP281	ICD1	0	1	1.3407	0.2481	3	225	86.8502	0.399	0.0036		
Ism5	SNP290	ICD10	0	1	0.5306	0.467	3	249	77.0174	0.4634	0.0011		
Ism5	SNP291	ICD11	0	1	0.297	0.5863	3	249	77.0905	0.4629	6.41E-04		
Ism5	SNP292	ICD12	0	1	0.0057	0.9399	3	247	77.288	0.465	1.23E-05		
Ism5	SNP293	ICD13	0	1	0.6954	0.4051	3	248	76.9242	0.4662	0.0015		
Ism5	SNP294	ICD14	0	1	0.334	0.5638	3	240	73.8477	0.487	7.14E-04		
Ism5	SNP282	ICD2	0	2	0.2478	0.7807	4	233	87.9324	0.3821	0.0013		
Ism5	SNP283	ICD3	0	2	2.9987	0.0518	4	233	85.964	0.3959	0.0155		
Ism5	SNP284	ICD4	0	1	3.0396	0.0826	3	234	84.993	0.4003	0.0078		
Ism5	SNP285	ICD5	0	1	0.2128	0.645	3	234	86.1151	0.3924	5.52E-04		
Ism5	SNP286	ICD6	0	2	0.347	0.7072	4	222	97.1876	0.2848	0.0022		
Ism5	SNP287	ICD7	0	1	4.0652	0.045	3	223	94.1679	0.3039	0.0127		
Ism5	SNP288	ICD8	0	1	0.0429	0.8361	3	249	76.75	0.4653	9.21E-05		
Ism5	SNP289	ICD9	0	1	0.0429	0.8361	3	249	76.7522	0.4653	9.21E-05		
Ism5	SNP295	ISL1	0	1	5.3583	0.0216	3	214	85.0949	0.4002	0.015		
Ism5	SNP296	ISL2	0	1	1.8436	0.176	3	214	89.7514	0.3674	0.0054		
Ism5	SNP297	ISL3	0	1	0.3471	0.5564	3	212	89.3226	0.3758	0.001		
Ism5	SNP298	ISL4	0	1	0.1938	0.6602	3	210	89.2481	0.3819	5.70E-04	0.9917	244
Ism5	SNP299	ISL5	0	1	3.5373	0.0614	3	203	88.0067	0.4005	0.0104		
Ism5	SNP300	ISL6	0	1	2.79	0.0964	3	201	97.8119	0.3286	0.0093		
Ism5	SNP301	ME1	0	1	1.1112	0.2929	3	238	79.2156	0.4223	0.0027	0.1303	244
Ism5	SNP310	ME10	0	1	2.756	0.0983	3	229	75.2022	0.4495	0.0066	0.3929	244
Ism5	SNP311	ME11	0	1	4.2052	0.0414	3	228	75.2806	0.4476	0.0102	0.3669	244
Ism5	SNP312	ME12	0	3	1.4229	0.2369	5	225	75.545	0.453	0.0104	0.7142	244
Ism5	SNP302	ME2	0	1	0.3714	0.5428	3	239	78.5795	0.4245	8.94E-04		
Ism5	SNP303	ME3	0	1	0.4136	0.5208	3	239	79.5402	0.4175	0.001		
Ism5	SNP304	ME4	0	1	5.2703	0.0226	3	239	76.1278	0.4425	0.0123	0.0078	244
Ism5	SNP305	ME5	0	1	3.9009	0.0494	3	239	74.7787	0.4524	0.0089		
Ism5	SNP306	ME6	0	3	3.0127	0.0308	5	237	74.8904	0.4561	0.0207	0.1093	242
Ism5	SNP307	ME7	0	1	3.8111	0.0521	3	236	74.6606	0.4483	0.0089	0.4373	244
Ism5	SNP308	ME8	0	1	0.3772	0.5397	3	236	75.6678	0.4408	8.94E-04		
Ism5	SNP309	ME9	0	1	3.0702	0.0811	3	229	76.6064	0.4393	0.0075		
Ism5	SNP313	P13K1	0	1	5.5297	0.0195	3	238	77.602	0.4289	0.0133	0.0149	244
Ism5	SNP322	P13K10	0	1	0.0712	0.7899	3	193	55.9515	0.5815	1.54E-04		
Ism5	SNP314	P13K2	0	1	0.2147	0.6435	3	238	74.8436	0.4492	4.97E-04		
Ism5	SNP315	P13K3	0	1	0.3175	0.5737	3	238	74.8661	0.4491	7.35E-04		
Ism5	SNP316	P13K4	0	1	1.63E-04	0.9898	3	238	74.7011	0.4503	3.76E-07		
Ism5	SNP317	P13K5	0	1	0.0788	0.7792	3	238	74.9294	0.4486	1.83E-04		
Ism5	SNP318	P13K6	0	1	0.2063	0.6501	3	238	75.0769	0.4475	4.79E-04		
Ism5	SNP319	P13K7	0	1	0.0032	0.9553	3	238	74.9278	0.4486	7.30E-06		
Ism5	SNP320	P13K8	0	1	0.21	0.6472	3	236	75.1171	0.4494	4.90E-04		
Ism5	SNP321	P13K9	0	1	0.0019	0.965	3	235	74.8323	0.4538	4.49E-06		
Ism5	SNP323	PME1	0	1	1.0019	0.3178	3	253	74.2349	0.4824	0.002		
Ism5	SNP324	PME2	0	1	7.6932	0.006	3	253	74.9318	0.4776	0.0159	0.0096	244
Ism5	SNP325	PME3	0	1	2.656	0.1044	3	249	70.3137	0.515	0.0052	0.1581	244
Ism5	SNP326	SAH1	0	1	4.7992	0.0297	3	193	75.0362	0.487	0.0128	0.0847	244
Ism5	SNP327	SAH2	0	1	3.489	0.0633	3	193	76.006	0.4804	0.0094	0.1347	244
Ism5	SNP328	SAH3	0	1	0.8357	0.3618	3	193	76.9749	0.4738	0.0023		
Ism5	SNP329	SAH4	0	1	0.5204	0.4715	3	193	77.0992	0.4729	0.0014		
Ism5	SNP330	SAH5	0	2	3.0733	0.0486	4	187	70.1461	0.5232	0.0157	0.0671	243
Ism5	SNP331	SAH6	0	1	2.0299	0.1568	3	121	87.8011	0.2915	0.0119		
Ism5+c	SNP79	ABC1	0	1	0.7057	0.4019	4	199	43.5175	0.671	0.0012		
Ism5+c	SNP88	ABC10	0	1	0.0881	0.7669	4	217	44.4592	0.6524	1.41E-04		
Ism5+c	SNP89	ABC11	0	1	0.0881	0.7669	4	217	44.4616	0.6524	1.41E-04		
Ism5+c	SNP90	ABC12	0	1	1.0917	0.2973	4	217	43.9327	0.6565	0.0017		
Ism5+c	SNP91	ABC13	0	1	0.0888	0.766	4	215	45.1687	0.6498	1.45E-04		
Ism5+c	SNP92	ABC14	0	1	0.1415	0.7071	4	217	44.1555	0.6548	2.25E-04		
Ism5+c	SNP93	ABC15	0	1	0.1736	0.6773	4	212	44.6715	0.6476	2.89E-04		
Ism5+c	SNP94	ABC16	0	1	0.0291	0.8646	4	208	45.7143	0.6431	5.00E-05		
Ism5+c	SNP95	ABC17	0	1	0.7577	0.3851	4	208	45.3966	0.6456	0.0013		
Ism5+c	SNP96	ABC18	0	1	0.7577	0.3851	4	208	45.3941	0.6456	0.0013		
Ism5+c	SNP97	ABC19	0	2	0.5943	0.5529	5	207	47.2798	0.6327	0.0021		
Ism5+c	SNP80	ABC2	0	1	0.7058	0.4019	4	199	43.5159	0.671	0.0012		
Ism5+c	SNP98	ABC20	0	1	0.0011	0.9739	4	208	45.5811	0.6442	1.84E-06		
Ism5+c	SNP99	ABC21	0	1	0.709	0.4008	4	205	46.0181	0.6385	0.0013		
Ism5+c	SNP81	ABC3	0	1	0.0096	0.9222	4	203	44.1237	0.6629	1.59E-05		
Ism5+c	SNP82	ABC4	0	3	0.2181	0.8838	6	210	44.2429	0.6594	0.0011		
Ism5+c	SNP83	ABC5	0	1	0.0119	0.9133	4	217	44.4217	0.6527	1.90E-05		
Ism5+c	SNP84	ABC6	0	1	0.1013	0.7505	4	217	44.4413	0.6525	1.62E-04		
Ism5+c	SNP85	ABC7	0	1	0.088	0.767	4	217	44.4504	0.6525	1.41E-04		
Ism5+c	SNP86	ABC8	0	1	0.088	0.767	4	217	44.4536	0.6524	1.41E-04		
Ism5+c	SNP87	ABC9	0	1	0.088	0.767	4	217	44.4565	0.6524	1.41E-04		
Ism5+c	SNP101	AL1_1	0	1	2.4216	0.1221	4	132	2.0605	0.9851	2.73E-04		
Ism5+c	SNP110	AL1_10	0	2	0.5311	0.5893	5	120	83.6297	0.4413	0.0049		
Ism5+c	SNP111	AL1_11	0	1	0.0641	0.8006	4	121	82.9068	0.4415	2.96E-04		
Ism5+c	SNP112	AL1_12	0	1	3.6464	0.0581	4	151	71.5983	0.4803	0.0126		
Ism5+c	SNP113	AL1_13	0	1	5.0452	0.0261	4	151	70.2039	0.4904	0.017		
Ism5+c	SNP114	AL1_14	0	1	5.0452	0.0261	4	151	70.2032	0.4904	0.017		
Ism5+c	SNP115	AL1_15	0	1	1.4598	0.2289	4	151	70.247	0.4901	0.0049		
Ism5+c	SNP116	AL1_16	0	1	5.0437	0.0262	4	151	70.1598	0.4907	0.017		
Ism5+c	SNP117	AL1_17	0	1	5.0437	0.0262	4	151	70.1616	0.4907	0.017		
Ism5+c	SNP118	AL1_18	0	1	5.0438	0.0262	4	151	70.1632	0.4907	0.017		

lsm5+c	SNP119	AL1_19	0	1	1.2412	0.267	4	150	71.2417	0.4861	0.0043		
lsm5+c	SNP102	AL1_2	0	1	1.0287	0.3123	4	133	59.1773	0.5718	0.0033		
lsm5+c	SNP120	AL1_20	0	1	1.8514	0.1757	4	149	62.9402	0.5441	0.0057		
lsm5+c	SNP121	AL1_21	0	1	1.8514	0.1757	4	149	62.9377	0.5441	0.0057		
lsm5+c	SNP122	AL1_22	0	1	1.8513	0.1757	4	149	62.9354	0.5441	0.0057		
lsm5+c	SNP123	AL1_23	0	1	1.3189	0.2526	4	148	63.4824	0.5386	0.0041		
lsm5+c	SNP124	AL1_24	0	1	1.319	0.2526	4	148	63.4838	0.5386	0.0041		
lsm5+c	SNP125	AL1_25	0	1	2.3855	0.1246	4	148	62.3285	0.547	0.0073		
lsm5+c	SNP126	AL1_26	0	1	2.3855	0.1246	4	148	62.3273	0.547	0.0073		
lsm5+c	SNP127	AL1_27	0	1	1.8246	0.1789	4	146	64.3048	0.5343	0.0058		
lsm5+c	SNP128	AL1_28	0	1	1.9136	0.1687	4	145	63.67	0.539	0.0061		
lsm5+c	SNP129	AL1_29	0	1	2.1243	0.1471	4	145	63.0054	0.5438	0.0067		
lsm5+c	SNP103	AL1_3	0	1	5.1644	0.0246	4	136	59.6875	0.5617	0.0166		
lsm5+c	SNP130	AL1_30	0	1	1.4497	0.2305	4	145	64.7119	0.5314	0.0047		
lsm5+c	SNP131	AL1_31	0	1	2.1087	0.1486	4	144	63.7878	0.5354	0.0068		
lsm5+c	SNP104	AL1_4	0	1	0.5198	0.4721	4	142	55.3869	0.5902	0.0015		
lsm5+c	SNP105	AL1_5	0	1	0.5795	0.4477	4	150	68.8599	0.5032	0.0019		
lsm5+c	SNP106	AL1_6	0	2	2.5433	0.082	5	148	68.5197	0.5102	0.0168		
lsm5+c	SNP107	AL1_7	0	2	0.3144	0.7308	5	120	83.5776	0.4416	0.0029		
lsm5+c	SNP108	AL1_8	0	1	0.5092	0.4769	4	121	82.2234	0.4461	0.0023		
lsm5+c	SNP109	AL1_9	0	1	1.782	0.1844	4	121	81.37	0.4518	0.0081		
lsm5+c	SNP100	AL16_1	0	1			4	104					
lsm5+c	SNP132	AL2_1	0	1	2.0292	0.1566	4	136	55.7728	0.5447	0.0068		
lsm5+c	SNP141	AL2_10	0	1	0.0544	0.8159	4	154	45.0818	0.6381	1.28E-04		
lsm5+c	SNP142	AL2_11	0	1	0.1757	0.6756	4	162	41.4207	0.6611	3.68E-04		
lsm5+c	SNP143	AL2_12	0	2	1.9618	0.144	5	160	42.574	0.6529	0.0085		
lsm5+c	SNP144	AL2_13	0	1	0.8304	0.3635	4	161	42.2879	0.6531	0.0018		
lsm5+c	SNP145	AL2_14	0	1	0.4732	0.4925	4	161	41.7565	0.6574	0.001		
lsm5+c	SNP146	AL2_15	0	1	0.1324	0.7164	4	160	39.7221	0.6721	2.71E-04		
lsm5+c	SNP147	AL2_16	0	1	0.4745	0.4919	4	160	39.2299	0.6761	9.60E-04		
lsm5+c	SNP148	AL2_17	0	1	0.358	0.5505	4	155	40.0043	0.6723	7.57E-04		
lsm5+c	SNP133	AL2_2	0	1	2.9884	0.086	4	148	44.6066	0.6506	0.0071		
lsm5+c	SNP134	AL2_3	0	1	0.5432	0.4623	4	149	47.8	0.6231	0.0014		
lsm5+c	SNP135	AL2_4	0	1	0.0047	0.9457	4	151	46.5268	0.6295	1.14E-05		
lsm5+c	SNP136	AL2_5	0	1	0.1314	0.7175	4	151	47.0276	0.6256	3.26E-04		
lsm5+c	SNP137	AL2_6	0	1	0.3197	0.5726	4	151	46.0211	0.6336	7.76E-04		
lsm5+c	SNP138	AL2_7	0	1	0.9232	0.3382	4	151	45.8361	0.635	0.0022		
lsm5+c	SNP139	AL2_8	0	1	0.1314	0.7175	4	151	47.0377	0.6255	3.26E-04		
lsm5+c	SNP140	AL2_9	0	2	0.6494	0.5238	5	149	47.0636	0.6298	0.0032		
lsm5+c	SNP149	AL3_1	0	1	0.2861	0.5936	4	146	49.7445	0.663	6.60E-04		
lsm5+c	SNP150	AL3_2	0	1	2.1808	0.1419	4	146	42.1131	0.7147	0.0043		
lsm5+c	SNP151	AL3_3	0	1	1.6573	0.2	4	145	45.3376	0.6949	0.0035		
lsm5+c	SNP152	AL3_4	0	1	5.5358	0.02	4	146	45.2624	0.6933	0.0116	0.0233	225
lsm5+c	SNP153	AL3_5	0	2	1.3291	0.2679	5	145	50.985	0.6569	0.0063		
lsm5+c	SNP154	AL3_6	0	1	0.0229	0.88	4	162	47.314	0.68	4.51E-05		
lsm5+c	SNP155	AL3_7	0	1	1.4231	0.2346	4	161	46.6075	0.6807	0.0028		
lsm5+c	SNP156	AL3_8	0	1	0.0705	0.7909	4	162	47.3275	0.6799	1.39E-04		
lsm5+c	SNP157	AL3_9	0	2	0.0669	0.9353	5	161	47.6044	0.68	2.66E-04		
lsm5+c	SNP158	AL5_1	0	1	0.4593	0.4991	4	139	41.0304	0.7208	9.23E-04		
lsm5+c	SNP167	AL5_10	0	1	0.223	0.6374	4	174	49.6374	0.6473	4.52E-04		
lsm5+c	SNP168	AL5_11	0	1	0.1897	0.6637	4	178	47.0167	0.6611	3.61E-04		
lsm5+c	SNP169	AL5_12	0	1	0.1898	0.6636	4	178	47.014	0.6611	3.61E-04		
lsm5+c	SNP170	AL5_13	0	3	0.0838	0.9688	6	175	48.0928	0.656	4.94E-04		
lsm5+c	SNP171	AL5_14	0	1	0.1796	0.6723	4	178	47.1857	0.6599	3.43E-04		
lsm5+c	SNP172	AL5_15	0	3	0.0845	0.9685	6	182	51.5893	0.6257	5.21E-04		
lsm5+c	SNP173	AL5_16	0	1	0.3292	0.5668	4	185	49.8471	0.6324	6.54E-04		
lsm5+c	SNP174	AL5_17	0	1	0.0267	0.8703	4	185	50.3968	0.6284	5.37E-05		
lsm5+c	SNP175	AL5_18	0	1	0.2989	0.5852	4	185	50.2601	0.6294	5.99E-04		
lsm5+c	SNP176	AL5_19	0	2	0.2078	0.8126	5	184	50.1247	0.6324	8.30E-04		
lsm5+c	SNP159	AL5_2	0	1	0.3831	0.5369	4	142	42.7287	0.7175	7.62E-04		
lsm5+c	SNP177	AL5_20	0	1	0.0646	0.7997	4	185	50.2937	0.6291	1.29E-04		
lsm5+c	SNP178	AL5_21	0	1	0.3292	0.5668	4	185	49.8485	0.6324	6.54E-04		
lsm5+c	SNP179	AL5_22	0	1	0.2902	0.5908	4	185	49.7982	0.6328	5.76E-04		
lsm5+c	SNP180	AL5_23	0	1	0.2798	0.5975	4	184	49.6496	0.6358	5.54E-04		
lsm5+c	SNP181	AL5_24	0	1	1.0956	0.2969	4	149	40.6481	0.7063	0.0022		
lsm5+c	SNP160	AL5_3	0	1	0.2297	0.6324	4	161	41.8099	0.7019	4.25E-04		
lsm5+c	SNP161	AL5_4	0	1	0.484	0.4876	4	171	41.8428	0.7006	8.47E-04		
lsm5+c	SNP162	AL5_5	0	1	0.0424	0.8371	4	171	41.9883	0.6996	7.45E-05		
lsm5+c	SNP163	AL5_6	0	1	0.1055	0.7457	4	171	49.9889	0.6423	2.21E-04		
lsm5+c	SNP164	AL5_7	0	1	0.2975	0.5862	4	171	49.7594	0.6439	6.19E-04		
lsm5+c	SNP165	AL5_8	0	1	0.1469	0.702	4	174	48.8203	0.6531	2.93E-04		
lsm5+c	SNP166	AL5_9	0	1	0.015	0.9026	4	174	49.4675	0.6485	3.04E-05		
lsm5+c	SNP182	AL8_1	0	1	0.6317	0.4279	4	159	43.2475	0.6795	0.0013		
lsm5+c	SNP191	AL8_10	0	1	0.0311	0.8601	4	172	52.8646	0.6029	7.19E-05		
lsm5+c	SNP192	AL8_11	0	1	0.4356	0.5101	4	168	58.2914	0.5771	0.0011		
lsm5+c	SNP193	AL8_12	0	1	0.1731	0.6779	4	173	55.811	0.5854	4.15E-04		
lsm5+c	SNP194	AL8_13	0	1	0.1731	0.6779	4	173	55.8076	0.5854	4.15E-04		
lsm5+c	SNP195	AL8_14	0	1	0.1628	0.6871	4	172	54.5144	0.5983	3.80E-04		
lsm5+c	SNP196	AL8_15	0	2	0.1742	0.8403	5	173	56.5734	0.5816	8.43E-04		
lsm5+c	SNP197	AL8_16	0	1	0.0234	0.8786	4	174	55.3676	0.5882	5.54E-05		
lsm5+c	SNP198	AL8_17	0	1	0.1988	0.6563	4	174	55.5134	0.5871	4.72E-04		
lsm5+c	SNP199	AL8_18	0	2	0.356	0.701	5	173	55.8505	0.587	0.0017		
lsm5+c	SNP200	AL8_19	0	1	0.1987	0.6563	4	174	55.6027	0.5864	4.72E-04		
lsm5+c	SNP183	AL8_2	0	1	0.0166	0.8977	4	141	53.9013	0.628	4.38E-05		
lsm5+c	SNP201	AL8_20	0	1	0.1987	0.6563	4	174	55.5996	0.5865	4.72E-04		
lsm5+c	SNP202	AL8_21	0	1	0.1962	0.6583	4	173	55.7441	0.5878	4.68E-04		
lsm5+c	SNP203	AL8_22	0	1	0.1988	0.6562	4	174	55.5034	0.5872	4.72E-04		
lsm5+c	SNP204	AL8_23	0	1	0.0031	0.9555	4	173	52.4448	0.6088	7.05E-06		
lsm5+c	SNP205	AL8_24	0	1	0.3667	0.5456	4	171	54.3861	0.5954	8.68E-04		
lsm5+c	SNP206	AL8_25	0	1	0.3752	0.541	4	167	45.9805	0.6531	7.79E-04		
lsm5+c	SNP207	AL8_26	0	1	0.3489	0.5557	4	138	33.1693	0.7598	6.07E-04		

lsm5+c	SNP184	AL8_3	0	1	0.1423	0.7065	4	163	48.2348	0.6444	3.11E-04		
lsm5+c	SNP185	AL8_4	0	1	0.1423	0.7065	4	163	48.2313	0.6444	3.11E-04		
lsm5+c	SNP186	AL8_5	0	1	0.1403	0.7084	4	162	50.0618	0.6306	3.20E-04		
lsm5+c	SNP187	AL8_6	0	1	0.1423	0.7065	4	163	48.2091	0.6445	3.10E-04		
lsm5+c	SNP188	AL8_7	0	1	0.2126	0.6453	4	170	53.3566	0.6021	4.98E-04		
lsm5+c	SNP189	AL8_8	0	1	0.2126	0.6453	4	170	53.3588	0.6021	4.98E-04		
lsm5+c	SNP190	AL8_9	0	1	0.2259	0.6352	4	172	52.7605	0.6037	5.20E-04		
lsm5+c	SNP24	Al9_1	0	1	0.0035	0.9529	4	225	53.0835	0.6094	6.06E-06		
lsm5+c	SNP25	Al9_2	0	1	0.0029	0.9572	4	226	54.1289	0.6003	5.11E-06		
lsm5+c	SNP208	anti1	0	1	0.1556	0.6936	4	205	53.2121	0.594	3.08E-04		
lsm5+c	SNP217	anti10	0	2	0.5701	0.5665	5	177	43.5429	0.6715	0.0021		
lsm5+c	SNP218	anti11	0	1	0.024	0.877	4	178	42.5788	0.6769	4.36E-05		
lsm5+c	SNP219	anti12	0	1	0.024	0.877	4	178	42.5758	0.6769	4.36E-05		
lsm5+c	SNP220	anti13	0	1	1.2296	0.269	4	178	43.5909	0.6692	0.0023		
lsm5+c	SNP221	anti14	0	1	0.1936	0.6605	4	170	41.4309	0.6768	3.68E-04		
lsm5+c	SNP209	anti2	0	1	0.4867	0.4862	4	208	56.5363	0.5728	1.00E-03		
lsm5+c	SNP210	anti3	0	1	4.383	0.0375	4	208	55.2468	0.5825	0.0088		
lsm5+c	SNP211	anti4	0	1	3.344	0.0689	4	202	58.4615	0.5606	0.0073		
lsm5+c	SNP212	anti5	0	1	0.1371	0.7116	4	196	46.7649	0.6502	2.45E-04		
lsm5+c	SNP213	anti6	0	1	0.8925	0.346	4	196	47.3234	0.6461	0.0016		
lsm5+c	SNP214	anti7	0	2	0.512	0.6001	5	184	47.3619	0.6453	0.002		
lsm5+c	SNP215	anti8	0	1	1.0159	0.3148	4	185	48.545	0.6345	0.002		
lsm5+c	SNP216	anti9	0	1	0.1491	0.6999	4	176	44.1591	0.6773	2.73E-04		
lsm5+c	SNP26	ASL1	0	1	2.8087	0.0954	4	194	62.7086	0.5483	0.0065		
lsm5+c	SNP35	ASL10	0	1	0.9307	0.3357	4	217	58.7409	0.5657	0.0019		
lsm5+c	SNP36	ASL11	0	1	5.0492	0.0256	4	222	62.5449	0.5322	0.0106		
lsm5+c	SNP37	ASL12	0	2	3.5464	0.0305	5	221	61.3177	0.5434	0.0147		
lsm5+c	SNP38	ASL13	0	1	0.4815	0.4885	4	222	61.7654	0.538	0.001		
lsm5+c	SNP39	ASL14	0	1	6.8691	0.0094	4	222	62.8912	0.5296	0.0146	0.1502	225
lsm5+c	SNP40	ASL15	0	2	2.4814	0.086	5	221	62.6158	0.5337	0.0105		
lsm5+c	SNP41	ASL16	0	1	7.0685	0.0085	4	202	64.5037	0.5082	0.0172	0.0088	225
lsm5+c	SNP42	ASL17	0	1	5.051	0.0256	4	222	62.5813	0.5319	0.0107	0.0191	225
lsm5+c	SNP43	ASL18	0	1	5.0509	0.0256	4	222	62.5798	0.5319	0.0106	0.0191	225
lsm5+c	SNP44	ASL19	0	1	5.0508	0.0256	4	222	62.5785	0.5319	0.0106	0.0191	225
lsm5+c	SNP27	ASL2	0	1	0.1871	0.6658	4	196	65.3296	0.5315	4.47E-04		
lsm5+c	SNP45	ASL20	0	1	4.0596	0.0451	4	222	62.817	0.5301	0.0086		
lsm5+c	SNP46	ASL21	0	1	6.5019	0.0114	4	222	61.9493	0.5366	0.0136	0.0107	225
lsm5+c	SNP47	ASL22	0	1	6.5019	0.0115	4	222	61.9481	0.5366	0.0136	0.0107	225
lsm5+c	SNP48	ASL23	0	1	6.5018	0.0115	4	222	61.9469	0.5366	0.0136	0.0107	225
lsm5+c	SNP49	ASL24	0	1	6.0807	0.0144	4	221	61.8234	0.5396	0.0127	0.0139	225
lsm5+c	SNP50	ASL25	0	1	6.3575	0.0124	4	221	58.5211	0.5642	0.0125	0.0309	225
lsm5+c	SNP51	ASL26	0	1	7.7416	0.0059	4	199	53.4162	0.5983	0.0156	0.004	225
lsm5+c	SNP52	ASL27	0	1	6.803	0.0098	4	201	53.0686	0.5991	0.0136	0.0055	225
lsm5+c	SNP53	ASL28	0	1	6.8029	0.0098	4	201	53.0661	0.5991	0.0136	0.0055	225
lsm5+c	SNP54	ASL29	0	1	2.3174	0.1295	4	205	50.3262	0.6175	0.0043		
lsm5+c	SNP28	ASL3	0	1	1.2969	0.2561	4	209	64.4414	0.5224	0.003		
lsm5+c	SNP55	ASL30	0	2	1.8149	0.1655	5	202	52.3846	0.6058	0.0071		
lsm5+c	SNP56	ASL31	0	1	4.2717	0.0401	4	193	48.903	0.619	0.0084		
lsm5+c	SNP57	ASL32	0	1	2.1337	0.1457	4	193	49.4391	0.6148	0.0043		
lsm5+c	SNP58	ASL33	0	1	0.3234	0.5702	4	205	65.7401	0.4965	7.94E-04		
lsm5+c	SNP59	ASL34	0	1	1.031	0.3111	4	206	64.0883	0.5146	0.0024		
lsm5+c	SNP60	ASL35	0	1	1.5856	0.2094	4	208	62.5556	0.5191	0.0037		
lsm5+c	SNP61	ASL36	0	3	1.1129	0.3449	6	203	62.3067	0.5291	0.0077		
lsm5+c	SNP62	ASL37	0	2	2.5156	0.0838	5	173	76.7626	0.4113	0.0171		
lsm5+c	SNP63	ASL38	0	3	0.7241	0.5389	6	171	75.3709	0.4285	0.0073		
lsm5+c	SNP29	ASL39	0	2	1.0902	0.3381	5	201	62.7861	0.5271	0.0051		
lsm5+c	SNP64	ASL4	0	1	0.6975	0.4045	4	217	59.2981	0.5615	0.0014		
lsm5+c	SNP65	ASL40	0	1	2.3775	0.1247	4	201	62.3927	0.5301	0.0056	0.2835	225
lsm5+c	SNP66	ASL41	0	1	2.2097	0.1387	4	200	63.413	0.5205	0.0053		
lsm5+c	SNP67	ASL42	0	1	2.8593	0.0925	4	193	63.2461	0.5291	0.007		
lsm5+c	SNP68	ASL43	0	1	2.42	0.1214	4	192	63.8457	0.5249	0.006		
lsm5+c	SNP69	ASL44	0	1	2.42	0.1214	4	192	63.8473	0.5249	0.006		
lsm5+c	SNP70	ASL45	0	1	2.1067	0.1483	4	190	64.4087	0.5217	0.0053		
lsm5+c	SNP71	ASL46	0	1	1.0331	0.3108	4	181	68.2336	0.5005	0.0029		
lsm5+c	SNP72	ASL47	0	1	1.0331	0.3108	4	181	68.2345	0.5005	0.0029		
lsm5+c	SNP73	ASL48	0	1	0.7026	0.403	4	180	68.4618	0.4958	0.002		
lsm5+c	SNP30	ASL49	0	1	0.7026	0.403	4	180	68.4631	0.4958	0.002		
lsm5+c	SNP74	ASL5	0	1	1.3855	0.2404	4	218	59.9917	0.5544	0.0028		
lsm5+c	SNP75	ASL50	0	1	0.7026	0.403	4	180	68.4643	0.4958	0.002		
lsm5+c	SNP76	ASL51	0	2	1.0281	0.3598	5	178	70.9993	0.4823	0.006		
lsm5+c	SNP77	ASL52	0	1	0.5583	0.456	4	160	65.7617	0.5277	0.0016		
lsm5+c	SNP31	ASL53	0	1	1.6155	0.2056	4	154	71.198	0.4998	0.0052		
lsm5+c	SNP78	ASL6	0	1	2.4458	0.1193	4	217	61.0985	0.5479	0.0051		
lsm5+c	SNP32	ASL7	0	1	2.4458	0.1193	4	217	61.0994	0.5479	0.0051		
lsm5+c	SNP33	ASL8	0	1	1.4606	0.2281	4	218	60.2124	0.5527	0.003		
lsm5+c	SNP34	ASL9	0	1	2.4372	0.1199	4	218	60.7141	0.549	0.005		
lsm5+c	SNP1	AUX01	0	1	0.9213	0.3385	4	169	55.7951	0.5489	0.0025		
lsm5+c	SNP2	AUX02	0	1	0.159	0.6906	4	176	54.449	0.5604	3.97E-04		
lsm5+c	SNP3	AUX03	0	1	0.0015	0.9693	4	178	54.875	0.5547	3.71E-06	0.5048	225
lsm5+c	SNP4	AUX04	0	1	0.2445	0.6216	4	179	55.6611	0.5479	6.18E-04		
lsm5+c	SNP5	AUX05	0	2	0.6828	0.5065	5	180	55.6019	0.5597	0.0033		
lsm5+c	SNP6	AUX06	0	2	1.2598	0.2862	5	180	56.2311	0.5547	0.0062		
lsm5+c	SNP7	AUX07	0	1	0.4304	0.5126	4	179	58.1249	0.5375	0.0011		
lsm5+c	SNP8	AUX08	0	2	0.0738	0.9289	5	171	57.9795	0.5498	3.89E-04		
lsm5+c	SNP9	AUX09	0	1	0.1927	0.6612	4	172	58.8354	0.5336	5.23E-04	0.3104	225
lsm5+c	SNP10	AUX10	0	2	0.4034	0.6687	5	171	58.394	0.5398	0.0022		
lsm5+c	SNP11	AUX11	0	2	0.0763	0.9266	5	171	59.0778	0.5344	4.15E-04		
lsm5+c	SNP12	AUX12	0	1	0.1421	0.7066	4	172	58.6907	0.5347	3.84E-04		
lsm5+c	SNP13	AUX13	0	2	0.0983	0.9065	5	170	58.2968	0.5409	5.31E-04		
lsm5+c	SNP14	AUX14	0	2	0.7982	0.4518	5	170	59.539	0.5311	0.0044		
lsm5+c	SNP15	AUX15	0	1	0.6507	0.421	4	168	60.6308	0.5226	0.0018		

lsm5+c	SNP16	AUX16	0	2	0.1904	0.8268	5	164	58.5273	0.5328	0.0011		
lsm5+c	SNP17	AUX17	0	1	0.0248	0.8751	4	165	58.9602	0.5264	7.11E-05		
lsm5+c	SNP18	AUX18	0	1	5.88E-04	0.9807	4	164	58.2048	0.5318	1.68E-06		
lsm5+c	SNP19	AUX19	0	1	1.3353	0.2496	4	161	58.2126	0.5274	0.0039		
lsm5+c	SNP20	AUX20	0	1	0.0057	0.9398	4	156	58.589	0.5323	1.71E-05		
lsm5+c	SNP21	AUX21	0	1	0.8478	0.3586	4	156	58.1966	0.5354	0.0025		
lsm5+c	SNP22	AUX22	0	2	0.5465	0.5801	5	153	55.1902	0.5654	0.0031		
lsm5+c	SNP23	AUX23	0	2	0.5198	0.5957	5	153	55.3104	0.5644	0.003		
lsm5+c	SNP222	cp1	0	1	0.3632	0.5474	4	207	36.391	0.7216	4.88E-04		
lsm5+c	SNP231	cp10	0	1	0.3712	0.543	4	218	45.277	0.6566	5.85E-04		
lsm5+c	SNP232	cp11	0	1	0.2123	0.6454	4	218	45.2508	0.6568	3.34E-04		
lsm5+c	SNP233	cp12	0	1	0.2717	0.6027	4	215	45.9775	0.6523	4.39E-04		
lsm5+c	SNP234	cp13	0	1	0.0019	0.9657	4	194	32.9264	0.7381	2.51E-06		
lsm5+c	SNP235	cp14	0	1	0.0445	0.8332	4	210	48.3134	0.6321	7.79E-05		
lsm5+c	SNP223	cp2	0	1	0.0965	0.7564	4	216	43.7699	0.6675	1.49E-04		
lsm5+c	SNP224	cp3	0	1	2.5421	0.1123	4	218	44.0767	0.6657	0.0039		
lsm5+c	SNP225	cp4	0	1	0.3827	0.5368	4	218	45.1936	0.6572	6.02E-04		
lsm5+c	SNP226	cp5	0	2	0.3636	0.6956	5	217	45.3305	0.6577	0.0011		
lsm5+c	SNP227	cp6	0	1	0.0498	0.8236	4	218	45.3206	0.6562	7.86E-05		
lsm5+c	SNP228	cp7	0	1	0.006	0.9384	4	218	45.4452	0.6553	9.47E-06		
lsm5+c	SNP229	cp8	0	1	0.0531	0.818	4	217	45.4917	0.6553	8.43E-05		
lsm5+c	SNP230	cp9	0	1	0.3712	0.543	4	218	45.2742	0.6566	5.85E-04		
lsm5+c	SNP236	Fe1	0	1	0.3204	0.572	4	190	61.59	0.5466	7.65E-04		
lsm5+c	SNP245	Fe10	0	1	0.0239	0.8773	4	165	58.0574	0.5658	6.29E-05		
lsm5+c	SNP246	Fe11	0	1	0.0254	0.8736	4	165	58.0552	0.5658	6.68E-05		
lsm5+c	SNP237	Fe2	0	1	0.0445	0.8331	4	203	61.7096	0.5442	9.99E-05		
lsm5+c	SNP238	Fe3	0	1	0.2117	0.6459	4	203	62.1971	0.5406	4.79E-04		
lsm5+c	SNP239	Fe4	0	1	0.2422	0.6232	4	204	61.8828	0.5407	5.45E-04		
lsm5+c	SNP240	Fe5	0	1	0.2559	0.6135	4	201	61.5751	0.5382	5.88E-04		
lsm5+c	SNP241	Fe6	0	1	0.372	0.5426	4	200	61.6055	0.5402	8.55E-04		
lsm5+c	SNP242	Fe7	0	1	0.0463	0.8299	4	191	60.8398	0.5417	1.11E-04		
lsm5+c	SNP243	Fe8	0	1	0.2937	0.5885	4	179	62.3583	0.5338	7.65E-04		
lsm5+c	SNP244	Fe9	0	1	0.7362	0.392	4	177	61.7194	0.5421	0.0019		
lsm5+c	SNP247	Fum1	0	1	1.1051	0.2943	4	218	59.0022	0.5644	0.0022		
lsm5+c	SNP248	Fum2	0	2	2.0093	0.1364	5	232	58.0406	0.5675	0.0075		
lsm5+c	SNP249	Fum3	0	1	2.8977	0.09	4	234	58.9327	0.5589	0.0055		
lsm5+c	SNP250	Fum4	0	1	3.8834	0.0499	4	234	58.325	0.5635	0.0072		
lsm5+c	SNP251	Fum5	0	1	3.2224	0.0739	4	232	56.1671	0.5781	0.0059		
lsm5+c	SNP252	Fum6	0	1	3.002	0.0845	4	232	56.3516	0.5767	0.0055		
lsm5+c	SNP253	Fum7	0	1	5.503	0.0198	4	232	55.9219	0.5799	0.01		
lsm5+c	SNP254	Fum8	0	1	5.2631	0.0227	4	232	56.269	0.5773	0.0096		
lsm5+c	SNP255	Ger1	0	1	2.7241	0.1009	4	152	72.0186	0.4657	0.0096		
lsm5+c	SNP264	Ger10	0	1	1.1122	0.2932	4	155	71.0301	0.4655	0.0038		
lsm5+c	SNP265	Ger11	0	1	1.1122	0.2932	4	155	71.0312	0.4654	0.0038		
lsm5+c	SNP266	Ger12	0	1	1.1122	0.2932	4	155	71.0323	0.4654	0.0038		
lsm5+c	SNP267	Ger13	0	1	1.1122	0.2932	4	155	71.0334	0.4654	0.0038		
lsm5+c	SNP268	Ger14	0	1	1.0663	0.3034	4	156	70.3008	0.4706	0.0036		
lsm5+c	SNP269	Ger15	0	1	1.0663	0.3034	4	156	70.2993	0.4706	0.0036		
lsm5+c	SNP270	Ger16	0	1	0.0141	0.9056	4	156	69.673	0.4753	4.75E-05		
lsm5+c	SNP271	Ger17	0	1	0.0013	0.9708	4	156	69.948	0.4732	4.54E-06		
lsm5+c	SNP272	Ger18	0	1	1.0661	0.3034	4	156	70.2509	0.4709	0.0036		
lsm5+c	SNP273	Ger19	0	1	1.0262	0.3126	4	155	70.6176	0.4685	0.0035		
lsm5+c	SNP256	Ger2	0	1	0.735	0.3926	4	153	70.2866	0.4751	0.0025		
lsm5+c	SNP274	Ger20	0	1	0.72	0.3975	4	155	67.1753	0.4944	0.0023		
lsm5+c	SNP275	Ger21	0	1	1.0661	0.3034	4	156	70.2403	0.471	0.0036		
lsm5+c	SNP276	Ger22	0	1	1.0661	0.3034	4	156	70.2415	0.471	0.0036		
lsm5+c	SNP277	Ger23	0	1	1.0661	0.3034	4	156	70.2427	0.471	0.0036		
lsm5+c	SNP278	Ger24	0	1	0.0141	0.9056	4	156	69.6721	0.4753	4.75E-05		
lsm5+c	SNP279	Ger25	0	1	0.8024	0.3718	4	154	71.3456	0.4654	0.0028		
lsm5+c	SNP280	Ger26	0	2	0.8731	0.4199	5	141	75.8688	0.4334	0.007		
lsm5+c	SNP257	Ger3	0	1	1.1668	0.2818	4	153	74.0058	0.4473	0.0042		
lsm5+c	SNP258	Ger4	0	1	0.016	0.8995	4	153	73.2275	0.4531	5.72E-05		
lsm5+c	SNP259	Ger5	0	1	0.9534	0.3304	4	154	68.6952	0.4862	0.0032		
lsm5+c	SNP260	Ger6	0	1	0.794	0.3743	4	154	69.0005	0.4839	0.0027		
lsm5+c	SNP261	Ger7	0	1	1.1295	0.2896	4	154	72.5652	0.4572	0.004		
lsm5+c	SNP262	Ger8	0	1	1.1295	0.2896	4	154	72.5662	0.4572	0.004		
lsm5+c	SNP263	Ger9	0	1	0.7156	0.3989	4	155	67.674	0.4907	0.0024		
lsm5+c	SNP281	ICD1	0	1	2.1739	0.1419	4	208	62.26	0.5396	0.0048		
lsm5+c	SNP290	ICD10	0	1	0.6267	0.4294	4	231	56.9026	0.5761	0.0011		
lsm5+c	SNP291	ICD11	0	1	0.4017	0.5268	4	231	56.9597	0.5757	7.38E-04		
lsm5+c	SNP292	ICD12	0	1	3.60E-06	0.9985	4	229	57.497	0.5744	6.69E-09		
lsm5+c	SNP293	ICD13	0	1	0.0181	0.8932	4	230	57.0999	0.5765	3.33E-05		
lsm5+c	SNP294	ICD14	0	1	0.2905	0.5904	4	223	58.0568	0.577	5.51E-04		
lsm5+c	SNP282	ICD2	0	2	0.4202	0.6575	5	215	63.391	0.5223	0.0019		
lsm5+c	SNP283	ICD3	0	1	0.084	0.7722	4	216	63.4615	0.5196	1.87E-04		
lsm5+c	SNP284	ICD4	0	1	4.8477	0.0287	4	216	60.8545	0.5394	0.0103		
lsm5+c	SNP285	ICD5	0	1	0.3138	0.576	4	216	62.6887	0.5255	6.89E-04		
lsm5+c	SNP286	ICD6	0	2	0.3875	0.6793	5	205	66.8589	0.4675	0.002		
lsm5+c	SNP287	ICD7	0	1	4.8231	0.0292	4	206	64.848	0.481	0.0122		
lsm5+c	SNP288	ICD8	0	1	0.6438	0.4232	4	231	56.8941	0.5762	0.0012		
lsm5+c	SNP289	ICD9	0	1	0.6438	0.4232	4	231	56.8925	0.5762	0.0012		
lsm5+c	SNP295	ISL1	0	1	5.8655	0.0164	4	194	58.1892	0.5623	0.0132	0.0591	225
lsm5+c	SNP296	ISL2	0	1	2.7262	0.1003	4	194	62.6514	0.5288	0.0066		
lsm5+c	SNP297	ISL3	0	1	0.1231	0.7261	4	192	63.23	0.529	3.02E-04		
lsm5+c	SNP298	ISL4	0	1	0.2596	0.611	4	190	63.4018	0.5325	6.39E-04	0.9833	225
lsm5+c	SNP299	ISL5	0	1	1.7409	0.1887	4	184	64.2948	0.533	0.0044		
lsm5+c	SNP300	ISL6	0	1	4.2973	0.0396	4	183	68.4147	0.4969	0.0118		
lsm5+c	SNP301	ME1	0	1	4.7312	0.0307	4	219	53.2757	0.5849	0.009		
lsm5+c	SNP310	ME10	0	1	4.2121	0.0414	4	210	49.955	0.6073	0.0079		
lsm5+c	SNP311	ME11	0	1	7.2687	0.0076	4	209	48.3986	0.6186	0.0133	0.2464	225
lsm5+c	SNP312	ME12	0	3	2.7403	0.0444	6	206	47.7298	0.6293	0.0148		

lsm5+c	SNP302	ME2	0	1	0.5603	0.4549	4	219	54.1271	0.5783	0.0011		
lsm5+c	SNP303	ME3	0	1	0.1736	0.6773	4	219	54.991	0.5715	3.40E-04		
lsm5+c	SNP304	ME4	0	1	7.6259	0.0062	4	219	52.1745	0.5935	0.0142	0.0015	225
lsm5+c	SNP305	ME5	0	1	1.7688	0.1849	4	219	52.5491	0.5906	0.0033		
lsm5+c	SNP306	ME6	0	3	3.2355	0.0231	6	217	51.9839	0.5987	0.018		
lsm5+c	SNP307	ME7	0	1	6.9211	0.0091	4	216	49.8868	0.6066	0.0126	0.1653	225
lsm5+c	SNP308	ME8	0	1	0.8588	0.3551	4	216	51.694	0.5923	0.0016		
lsm5+c	SNP309	ME9	0	1	4.6948	0.0314	4	210	51.1172	0.5982	0.009	0.1416	244
lsm5+c	SNP313	P13K1	0	1	7.0734	0.0084	4	222	59.8212	0.5391	0.0147	0.0022	225
lsm5+c	SNP322	P13K10	0	1	0.3655	0.5462	4	184	45.1828	0.6479	7.00E-04		
lsm5+c	SNP314	P13K2	0	1	0.5712	0.4506	4	222	57.6459	0.5559	0.0011		
lsm5+c	SNP315	P13K3	0	1	0.8358	0.3616	4	222	57.6592	0.5558	0.0017		
lsm5+c	SNP316	P13K4	0	1	0.1989	0.6561	4	222	57.4792	0.5572	3.97E-04		
lsm5+c	SNP317	P13K5	0	1	0.39	0.5329	4	222	57.8661	0.5542	7.83E-04		
lsm5+c	SNP318	P13K6	0	1	0.1935	0.6605	4	222	57.8458	0.5544	3.88E-04		
lsm5+c	SNP319	P13K7	0	1	0.5558	0.4567	4	222	58.1873	0.5517	0.0011		
lsm5+c	SNP320	P13K8	0	1	0.1851	0.6675	4	220	57.9563	0.555	3.74E-04		
lsm5+c	SNP321	P13K9	0	1	0.1121	0.7381	4	220	57.554	0.5581	2.25E-04		
lsm5+c	SNP323	PME1	0	1	0.0404	0.8409	4	235	56.4695	0.5789	7.24E-05		
lsm5+c	SNP324	PME2	0	1	7.7381	0.0058	4	235	54.4372	0.5941	0.0134	0.0157	225
lsm5+c	SNP325	PME3	0	1	0.6768	0.4115	4	231	52.1621	0.6149	0.0011		
lsm5+c	SNP326	SAH1	0	1	9.0768	0.003	4	175	52.911	0.6006	0.0207	0.0124	225
lsm5+c	SNP327	SAH2	0	1	7.8781	0.0056	4	175	54.1857	0.591	0.0184	0.0111	225
lsm5+c	SNP328	SAH3	0	1	2.9374	0.0883	4	175	51.3059	0.6127	0.0065		
lsm5+c	SNP329	SAH4	0	1	2.6397	0.106	4	175	52.7977	0.6014	0.006		
lsm5+c	SNP330	SAH5	0	2	3.4545	0.0338	5	170	51.2831	0.6134	0.0157	0.0433	224
lsm5+c	SNP331	SAH6	0	1	1.5508	0.2156	4	112	54.8598	0.5186	0.0067		
lsmc	SNP79	ABC1	0	1	4.3739	0.0378	3	200	78.7639	0.4064	0.013		
lsmc	SNP88	ABC10	0	1	3.3472	0.0687	3	218	75.1785	0.4455	0.0085		
lsmc	SNP89	ABC11	0	1	3.3472	0.0687	3	218	75.1804	0.4455	0.0085		
lsmc	SNP90	ABC12	0	1	4.4645	0.0357	3	218	75.8552	0.4405	0.0115		
lsmc	SNP91	ABC13	0	1	3.2608	0.0723	3	216	76.6279	0.4376	0.0085		
lsmc	SNP92	ABC14	0	1	5.82E-04	0.9808	3	218	76.5793	0.4352	1.51E-06		
lsmc	SNP93	ABC15	0	1	1.0419	0.3085	3	213	73.7953	0.4643	0.0026		
lsmc	SNP94	ABC16	0	1	0.0506	0.8223	3	209	69.4532	0.4966	1.22E-04		
lsmc	SNP95	ABC17	0	1	3.958	0.048	3	209	69.7208	0.4947	0.0096		
lsmc	SNP96	ABC18	0	1	3.958	0.048	3	209	69.7222	0.4947	0.0096		
lsmc	SNP97	ABC19	0	2	0.2353	0.7905	4	208	70.3732	0.4924	0.0011		
lsmc	SNP80	ABC2	0	1	4.3739	0.0378	3	200	78.7662	0.4064	0.013		
lsmc	SNP98	ABC20	0	1	0.1104	0.74	3	209	69.3098	0.4976	2.65E-04		
lsmc	SNP99	ABC21	0	1	3.1467	0.0776	3	206	60.5402	0.5526	0.0068		
lsmc	SNP81	ABC3	0	1	4.206	0.0416	3	204	71.6326	0.4721	0.0109		
lsmc	SNP82	ABC4	0	3	1.703	0.1675	5	211	75.6073	0.4455	0.0134		
lsmc	SNP83	ABC5	0	1	4.7334	0.0307	3	218	75.0057	0.4468	0.012		
lsmc	SNP84	ABC6	0	1	3.1755	0.0761	3	218	71.8403	0.4701	0.0077		
lsmc	SNP85	ABC7	0	1	3.3472	0.0687	3	218	75.1723	0.4456	0.0085		
lsmc	SNP86	ABC8	0	1	3.3472	0.0687	3	218	75.1745	0.4456	0.0085		
lsmc	SNP87	ABC9	0	1	3.3472	0.0687	3	218	75.1765	0.4455	0.0085		
lsmc	SNP101	AL1_1	0	1	0.1396	0.7093	3	133	141.3566	0.0714	9.75E-04		
lsmc	SNP110	AL1_10	0	2	0.128	0.88	4	121	73.4161	0.548	9.56E-04		
lsmc	SNP111	AL1_11	0	1	1.1363	0.2885	3	122	71.123	0.5585	0.0041		
lsmc	SNP112	AL1_12	0	1	0.1324	0.7165	3	152	78.2595	0.4746	4.58E-04		
lsmc	SNP113	AL1_13	0	1	0.2823	0.596	3	152	76.6745	0.4852	9.56E-04		
lsmc	SNP114	AL1_14	0	1	0.2823	0.596	3	152	76.6707	0.4852	9.56E-04		
lsmc	SNP115	AL1_15	0	1	0.3317	0.5655	3	152	79.0484	0.4693	0.0012		
lsmc	SNP116	AL1_16	0	1	0.2825	0.5959	3	152	76.6461	0.4854	9.56E-04		
lsmc	SNP117	AL1_17	0	1	0.2825	0.5958	3	152	76.644	0.4854	9.56E-04		
lsmc	SNP118	AL1_18	0	1	0.2825	0.5958	3	152	76.6421	0.4854	9.56E-04		
lsmc	SNP119	AL1_19	0	1	0.2543	0.6148	3	151	76.3689	0.4906	8.58E-04		
lsmc	SNP102	AL1_2	0	1	1.2455	0.2664	3	134	64.6756	0.575	0.004		
lsmc	SNP120	AL1_20	0	1	0.3619	0.5484	3	150	81.5551	0.4526	0.0013		
lsmc	SNP121	AL1_21	0	1	0.3619	0.5484	3	150	81.5564	0.4526	0.0013		
lsmc	SNP122	AL1_22	0	1	0.3619	0.5483	3	150	81.5576	0.4526	0.0013		
lsmc	SNP123	AL1_23	0	1	0.7921	0.3749	3	149	75.5087	0.4715	0.0028		
lsmc	SNP124	AL1_24	0	1	0.7921	0.3749	3	149	75.5063	0.4715	0.0028		
lsmc	SNP125	AL1_25	0	1	0.0283	0.8667	3	149	74.3475	0.4796	9.87E-05		
lsmc	SNP126	AL1_26	0	1	0.0283	0.8667	3	149	74.3442	0.4797	9.87E-05		
lsmc	SNP127	AL1_27	0	1	1.2034	0.2744	3	147	72.3746	0.4827	0.0042		
lsmc	SNP128	AL1_28	0	1	0.0995	0.7529	3	146	72.1059	0.4881	3.49E-04		
lsmc	SNP129	AL1_29	0	1	0.6357	0.4266	3	146	73.0662	0.4813	0.0023		
lsmc	SNP103	AL1_3	0	1	0.188	0.6652	3	137	71.95	0.5176	6.62E-04		
lsmc	SNP130	AL1_30	0	1	1.2355	0.2682	3	146	72.8572	0.4828	0.0044		
lsmc	SNP131	AL1_31	0	1	0.6381	0.4257	3	145	73.9554	0.4766	0.0023		
lsmc	SNP104	AL1_4	0	1	0.0122	0.9122	3	143	76.3067	0.4999	4.27E-05		
lsmc	SNP105	AL1_5	0	1	0.0508	0.822	3	151	79.1243	0.4706	1.78E-04		
lsmc	SNP106	AL1_6	0	2	0.2799	0.7563	4	149	79.5664	0.4723	0.002		
lsmc	SNP107	AL1_7	0	2	1.0469	0.3542	4	121	68.2027	0.5801	0.0073		
lsmc	SNP108	AL1_8	0	1	0.223	0.6376	3	122	73.5589	0.5434	8.35E-04		
lsmc	SNP109	AL1_9	0	1	0.2377	0.6268	3	122	69.2447	0.5701	8.37E-04		
lsmc	SNP100	AL16_1	0	1	2.6935	0.1038	3	105	96.8387	0.216	0.0201		
lsmc	SNP132	AL2_1	0	1	0.0542	0.8163	3	137	106.3141	0.3612	2.53E-04		
lsmc	SNP141	AL2_10	0	1	0.3759	0.5407	3	155	112.1032	0.2865	0.0017		
lsmc	SNP142	AL2_11	0	1	0.8263	0.3647	3	163	106.9061	0.3022	0.0035		
lsmc	SNP143	AL2_12	0	2	4.6552	0.0108	4	161	111.5434	0.2803	0.0416		
lsmc	SNP144	AL2_13	0	1	1.2873	0.2582	3	162	112.2782	0.2711	0.0058		
lsmc	SNP145	AL2_14	0	1	0.9959	0.3198	3	162	109.2508	0.2907	0.0044		
lsmc	SNP146	AL2_15	0	1	1.0045	0.3177	3	161	112.0209	0.2646	0.0046		
lsmc	SNP147	AL2_16	0	1	1.065	0.3036	3	161	113.1929	0.2569	0.0049		
lsmc	SNP148	AL2_17	0	1	0.1995	0.6557	3	156	108.2892	0.2927	9.05E-04		
lsmc	SNP133	AL2_2	0	1	6.3497	0.0128	3	149	96.5597	0.4012	0.0255		

lsmc	SNP134	AL2_3	0	1	1.9394	0.1658	3	150	112.8862	0.3007	0.009
lsmc	SNP135	AL2_4	0	1	0.0139	0.9062	3	152	105.1761	0.3401	6.04E-05
lsmc	SNP136	AL2_5	0	1	1.0393	0.3096	3	152	109.5635	0.3126	0.0047
lsmc	SNP137	AL2_6	0	1	0.8869	0.3478	3	152	106.4424	0.3322	0.0039
lsmc	SNP138	AL2_7	0	1	1.8051	0.1811	3	152	106.3898	0.3325	0.0079
lsmc	SNP139	AL2_8	0	1	1.0401	0.3094	3	152	109.6203	0.3122	0.0047
lsmc	SNP140	AL2_9	0	2	2.0238	0.1357	4	150	119.4177	0.26	0.02
lsmc	SNP149	AL3_1	0	1	0.5188	0.4725	3	147	1.5673	0.9876	4.39E-05
lsmc	SNP150	AL3_2	0	1	0.6798	0.411	3	147	63.8051	0.4937	0.0023
lsmc	SNP151	AL3_3	0	1	0.3187	0.5733	3	146	65.4808	0.4815	0.0011
lsmc	SNP152	AL3_4	0	1	0.36	0.5494	3	147	63.0422	0.4998	0.0012
lsmc	SNP153	AL3_5	0	2	0.0935	0.9108	4	146	64.4277	0.4943	6.48E-04
lsmc	SNP154	AL3_6	0	1	0.8056	0.3708	3	163	54.5768	0.5793	0.0021
lsmc	SNP155	AL3_7	0	1	0.0067	0.9348	3	162	53.5311	0.5898	1.70E-05
lsmc	SNP156	AL3_8	0	1	1.3645	0.2445	3	163	54.063	0.5832	0.0035
lsmc	SNP157	AL3_9	0	2	0.8834	0.4154	4	162	52.1784	0.6002	0.0044
lsmc	SNP158	AL5_1	0	1	0.1003	0.7519	3	140	76.8799	0.4697	3.80E-04
lsmc	SNP167	AL5_10	0	1	1.8429	0.1764	3	175	78.4893	0.4569	0.0057
lsmc	SNP168	AL5_11	0	1	0.6292	0.4287	3	179	84.8334	0.411	0.0021
lsmc	SNP169	AL5_12	0	1	0.6293	0.4287	3	179	84.8364	0.411	0.0021
lsmc	SNP170	AL5_13	0	3	1.1359	0.336	5	176	85.2529	0.4093	0.0114
lsmc	SNP171	AL5_14	0	1	3.0255	0.0837	3	179	83.5352	0.42	0.0098
lsmc	SNP172	AL5_15	0	3	3.0833	0.0287	5	183	79.4401	0.4473	0.0279
lsmc	SNP173	AL5_16	0	1	3.8315	0.0518	3	186	76.0572	0.4622	0.0111
lsmc	SNP174	AL5_17	0	1	1.3607	0.2449	3	186	76.7615	0.4572	0.004
lsmc	SNP175	AL5_18	0	1	2.3341	0.1283	3	186	72.8266	0.485	0.0065
lsmc	SNP176	AL5_19	0	2	2.0951	0.126	4	185	73.7778	0.4811	0.0118
lsmc	SNP159	AL5_2	0	1	0.2958	0.5874	3	143	83.7757	0.4364	0.0012
lsmc	SNP177	AL5_20	0	1	0.6075	0.4367	3	186	83.2681	0.4112	0.0019
lsmc	SNP178	AL5_21	0	1	3.8314	0.0518	3	186	76.0704	0.4621	0.0111
lsmc	SNP179	AL5_22	0	1	0.554	0.4576	3	186	83.597	0.4089	0.0018
lsmc	SNP180	AL5_23	0	1	0.5904	0.4432	3	185	83.8095	0.4082	0.0019
lsmc	SNP181	AL5_24	0	1	1.2778	0.2601	3	150	105.0083	0.235	0.0065
lsmc	SNP160	AL5_3	0	1	1.3284	0.2508	3	162	87.0159	0.3969	0.0049
lsmc	SNP161	AL5_4	0	1	2.0217	0.1569	3	172	77.2375	0.4501	0.0065
lsmc	SNP162	AL5_5	0	1	0.0103	0.9193	3	172	81.2524	0.4215	3.46E-05
lsmc	SNP163	AL5_6	0	1	0.1899	0.6635	3	172	81.0363	0.4463	6.11E-04
lsmc	SNP164	AL5_7	0	1	0.6785	0.4112	3	172	81.3054	0.4445	0.0022
lsmc	SNP165	AL5_8	0	1	0.5639	0.4537	3	175	82.25	0.4309	0.0018
lsmc	SNP166	AL5_9	0	1	2.2793	0.1329	3	175	79.9246	0.447	0.0072
lsmc	SNP182	AL8_1	0	1	2.0149	0.1577	3	160	73.9937	0.466	0.0067
lsmc	SNP191	AL8_10	0	1	0.1312	0.7177	3	173	83.8485	0.4288	4.33E-04
lsmc	SNP192	AL8_11	0	1	1.6412	0.2019	3	169	74.2602	0.4962	0.0049
lsmc	SNP193	AL8_12	0	1	0.74	0.3909	3	174	79.9707	0.4527	0.0023
lsmc	SNP194	AL8_13	0	1	0.7399	0.3909	3	174	79.9723	0.4527	0.0023
lsmc	SNP195	AL8_14	0	1	1.7529	0.1873	3	173	80.734	0.4402	0.0057
lsmc	SNP196	AL8_15	0	2	0.4207	0.6572	4	174	84.5475	0.4214	0.0028
lsmc	SNP197	AL8_16	0	1	0.2121	0.6457	3	175	84.261	0.4201	7.03E-04
lsmc	SNP198	AL8_17	0	1	0.6997	0.404	3	175	80.8382	0.4436	0.0022
lsmc	SNP199	AL8_18	0	2	1.6726	0.1908	4	174	76.4095	0.4771	0.0101
lsmc	SNP200	AL8_19	0	1	0.7005	0.4038	3	175	80.7466	0.4443	0.0022
lsmc	SNP183	AL8_2	0	1	0.0856	0.7702	3	142	77.5645	0.4262	3.46E-04
lsmc	SNP201	AL8_20	0	1	0.7005	0.4038	3	175	80.7489	0.4443	0.0022
lsmc	SNP202	AL8_21	0	1	0.7896	0.3755	3	174	77.6191	0.4664	0.0024
lsmc	SNP203	AL8_22	0	1	0.7004	0.4038	3	175	80.7609	0.4442	0.0022
lsmc	SNP204	AL8_23	0	1	0.2365	0.6273	3	174	84.8922	0.4182	7.91E-04
lsmc	SNP205	AL8_24	0	1	0.7823	0.3777	3	172	80.1475	0.4521	0.0025
lsmc	SNP206	AL8_25	0	1	0.3746	0.5413	3	168	79.6791	0.4623	0.0012
lsmc	SNP207	AL8_26	0	1	0.2185	0.6409	3	139	90.3877	0.4489	8.66E-04
lsmc	SNP184	AL8_3	0	1	1.0211	0.3137	3	164	72.4593	0.4717	0.0033
lsmc	SNP185	AL8_4	0	1	1.0211	0.3137	3	164	72.4571	0.4717	0.0033
lsmc	SNP186	AL8_5	0	1	1.0167	0.3148	3	163	72.7931	0.4723	0.0033
lsmc	SNP187	AL8_6	0	1	1.0221	0.3135	3	164	72.3814	0.4723	0.0033
lsmc	SNP188	AL8_7	0	1	0.7942	0.3741	3	171	75.1499	0.469	0.0025
lsmc	SNP189	AL8_8	0	1	0.7943	0.3741	3	171	75.1481	0.469	0.0025
lsmc	SNP190	AL8_9	0	1	0.7215	0.3968	3	173	80.5131	0.4515	0.0023
lsmc	SNP24	Al9_1	0	1	1.4046	0.2372	3	226	87.5361	0.3659	0.0039
lsmc	SNP25	Al9_2	0	1	1.4009	0.2378	3	227	87.0279	0.3671	0.0039
lsmc	SNP208	anti1	0	1	4.4956	0.0352	3	206	76.944	0.4159	0.0127
lsmc	SNP217	anti10	0	2	0.8093	0.4468	4	178	94.6857	0.2474	0.0068
lsmc	SNP218	anti11	0	1	1.0174	0.3145	3	179	95.5229	0.2365	0.0043
lsmc	SNP219	anti12	0	1	1.0174	0.3145	3	179	95.5234	0.2365	0.0043
lsmc	SNP220	anti13	0	1	1.516	0.2198	3	179	93.329	0.254	0.0063
lsmc	SNP221	anti14	0	1	0.3811	0.5378	3	171	95.8999	0.2393	0.0017
lsmc	SNP209	anti2	0	1	0.2068	0.6497	3	209	88.4229	0.3242	6.69E-04
lsmc	SNP210	anti3	0	1	0.106	0.7451	3	209	88.0113	0.3273	3.41E-04
lsmc	SNP211	anti4	0	1	0.8918	0.3461	3	203	92.351	0.2997	0.0031
lsmc	SNP212	anti5	0	1	0.535	0.4654	3	197	84.7488	0.3595	0.0017
lsmc	SNP213	anti6	0	1	0.1869	0.666	3	197	84.7882	0.3592	6.08E-04
lsmc	SNP214	anti7	0	2	1.3931	0.2509	4	185	91.0139	0.2693	0.011
lsmc	SNP215	anti8	0	1	0.8183	0.3669	3	186	91.0149	0.2653	0.0032
lsmc	SNP216	anti9	0	1	2.167	0.1428	3	177	92.3182	0.2733	0.0089
lsmc	SNP26	ASL1	0	1	0.0043	0.9477	3	195	77.842	0.4339	1.25E-05
lsmc	SNP35	ASL10	0	1	0.5793	0.4474	3	218	84.9374	0.3695	0.0017
lsmc	SNP36	ASL11	0	1	2.1825	0.141	3	223	83.2293	0.3788	0.0061
lsmc	SNP37	ASL12	0	2	1.1585	0.3159	4	222	83.2076	0.3818	0.0065
lsmc	SNP38	ASL13	0	1	0.449	0.5035	3	223	83.9649	0.3733	0.0013
lsmc	SNP39	ASL14	0	1	1.4849	0.2243	3	223	83.4346	0.3773	0.0041
lsmc	SNP40	ASL15	0	2	0.7155	0.4901	4	222	85.6746	0.3634	0.0041
lsmc	SNP41	ASL16	0	1	0.3924	0.5318	3	203	85.1246	0.382	0.0012
lsmc	SNP42	ASL17	0	1	2.1826	0.141	3	223	83.1687	0.3793	0.0061

lsmc	SNP43	ASL18	0	1	2.1826	0.141	3	223	83.1703	0.3793	0.0061		
lsmc	SNP44	ASL19	0	1	2.1826	0.141	3	223	83.1719	0.3793	0.0061		
lsmc	SNP27	ASL2	0	1	1.9873	0.1602	3	197	73.6119	0.4619	0.0054	0.6997	244
lsmc	SNP45	ASL20	0	1	2.0327	0.1553	3	223	83.2284	0.3788	0.0057		
lsmc	SNP46	ASL21	0	1	2.9167	0.0891	3	223	83.5	0.3768	0.0082		
lsmc	SNP47	ASL22	0	1	2.9167	0.0891	3	223	83.5021	0.3768	0.0082		
lsmc	SNP48	ASL23	0	1	2.9167	0.0891	3	223	83.504	0.3768	0.0082		
lsmc	SNP49	ASL24	0	1	3.3452	0.0687	3	222	82.8761	0.3815	0.0093		
lsmc	SNP50	ASL25	0	1	0.2831	0.5952	3	222	86.7833	0.3523	8.26E-04		
lsmc	SNP51	ASL26	0	1	1.2875	0.2579	3	200	91.1591	0.3483	0.0042		
lsmc	SNP52	ASL27	0	1	0.9252	0.3373	3	202	90.6157	0.3492	0.003		
lsmc	SNP53	ASL28	0	1	0.9252	0.3373	3	202	90.6148	0.3492	0.003		
lsmc	SNP54	ASL29	0	1	0.6547	0.4194	3	206	91.6325	0.3286	0.0021		
lsmc	SNP28	ASL3	0	1	3.1587	0.077	3	210	73.0561	0.4547	0.0082		
lsmc	SNP55	ASL30	0	2	0.0437	0.9573	4	203	91.5898	0.3354	2.86E-04		
lsmc	SNP56	ASL31	0	1	1.0879	0.2982	3	194	91.5703	0.3363	0.0037		
lsmc	SNP57	ASL32	0	1	2.9151	0.0894	3	194	91.3322	0.3381	0.0099		
lsmc	SNP58	ASL33											
lsmc	SNP59	ASL34	0	1	0.6141	0.4342	3	207	90.967	0.3606	0.0019		
lsmc	SNP60	ASL35	0	1	0.222	0.638	3	209	93.7017	0.3483	6.92E-04		
lsmc	SNP61	ASL36	0	3	0.7782	0.5074	5	204	95.6875	0.3436	0.0075		
lsmc	SNP62	ASL37	0	2	0.2989	0.742	4	174	97.7	0.3481	0.0022		
lsmc	SNP63	ASL38	0	3	0.9587	0.4136	5	172	97.3393	0.3579	0.0107		
lsmc	SNP64	ASL39	0	2	0.1109	0.895	4	202	101.5873	0.3096	7.58E-04		
lsmc	SNP29	ASL4	0	1	0.4279	0.5137	3	218	86.1363	0.3613	0.0013		
lsmc	SNP65	ASL40	0	1	4.7084	0.0312	3	202	100.4909	0.311	0.0161		
lsmc	SNP66	ASL41	0	1	0.2213	0.6386	3	201	103.9488	0.2811	7.91E-04		
lsmc	SNP67	ASL42	0	1	0.9175	0.3393	3	194	105.0042	0.2714	0.0034		
lsmc	SNP68	ASL43	0	1	0.5577	0.4561	3	193	107.1032	0.2579	0.0021		
lsmc	SNP69	ASL44	0	1	0.5577	0.4561	3	193	107.1036	0.2579	0.0021		
lsmc	SNP70	ASL45	0	1	1.0233	0.313	3	191	98.8187	0.3189	0.0036		
lsmc	SNP71	ASL46	0	1	0.6861	0.4086	3	182	103.9387	0.2999	0.0026		
lsmc	SNP72	ASL47	0	1	0.6861	0.4086	3	182	103.9398	0.2999	0.0026		
lsmc	SNP73	ASL48	0	1	0.2821	0.596	3	181	102.7131	0.3067	0.0011		
lsmc	SNP74	ASL49	0	1	0.2821	0.596	3	181	102.7118	0.3067	0.0011		
lsmc	SNP30	ASL5	0	1	3.2654	0.0721	3	219	84.2534	0.3724	0.0094		
lsmc	SNP75	ASL50	0	1	0.2821	0.596	3	181	102.7107	0.3067	0.0011		
lsmc	SNP76	ASL51	0	2	2.2141	0.1122	4	179	97.2709	0.3504	0.0161		
lsmc	SNP77	ASL52	0	1	0.1476	0.7014	3	161	112.1866	0.2372	6.99E-04		
lsmc	SNP78	ASL53	0	1	2.7949	0.0966	3	155	107.8195	0.2606	0.0133		
lsmc	SNP31	ASL6	0	1	2.3653	0.1255	3	218	85.7416	0.3638	0.0069		
lsmc	SNP32	ASL7	0	1	2.3653	0.1255	3	218	85.7441	0.3638	0.0069		
lsmc	SNP33	ASL8	0	1	3.1305	0.0782	3	219	85.1004	0.3661	0.0091		
lsmc	SNP34	ASL9	0	1	2.2992	0.1309	3	219	85.5121	0.3631	0.0067		
lsmc	SNP1	AUX01	0	1	0.0648	0.7994	3	170	95.1642	0.2171	2.98E-04		
lsmc	SNP2	AUX02	0	1	0.464	0.4967	3	177	93.5007	0.2265	0.002		
lsmc	SNP3	AUX03	0	1	10.0717	0.0018	3	179	103.251	0.1393	0.0484	0.0214	225
lsmc	SNP4	AUX04	0	1	0.6619	0.417	3	180	111.0767	0.069	0.0034		
lsmc	SNP5	AUX05	0	2	0.959	0.3852	4	181	93.8301	0.2125	0.0083		
lsmc	SNP6	AUX06	0	2	0.5271	0.5912	4	181	91.4937	0.2321	0.0045		
lsmc	SNP7	AUX07	0	1	10.0122	0.0018	3	180	104.6417	0.1242	0.0487	0.0327	225
lsmc	SNP8	AUX08	0	2	6.3373	0.0022	4	172	98.4834	0.1994	0.059	0.0235	225
lsmc	SNP9	AUX09	0	1	12.2757	5.84E-04	3	173	99.6622	0.1703	0.0589	0.0056	225
lsmc	SNP10	AUX10	0	2	1.0551	0.3504	4	172	111.2416	0.0793	0.0113		
lsmc	SNP11	AUX11	0	2	7.8749	5.34E-04	4	172	103.1031	0.1466	0.0781	0.0046	225
lsmc	SNP12	AUX12	0	1	7.974	0.0053	3	173	105.8811	0.1186	0.0406	0.0297	225
lsmc	SNP13	AUX13	0	2	0.2839	0.7532	4	171	97.1809	0.2003	0.0027		
lsmc	SNP14	AUX14	0	2	1.1124	0.3311	4	171	101.2967	0.1665	0.0108		
lsmc	SNP15	AUX15	0	1	8.3791	0.0043	3	169	108.4134	0.1149	0.0439	0.0336	225
lsmc	SNP16	AUX16	0	2	0.377	0.6865	4	165	103.0055	0.1727	0.0038		
lsmc	SNP17	AUX17	0	1	6.3053	0.013	3	166	110.4914	0.1071	0.0339		
lsmc	SNP18	AUX18	0	1	0.195	0.6594	3	165	96.0967	0.2242	9.17E-04		
lsmc	SNP19	AUX19	0	1	1.6071	0.2067	3	162	100.5545	0.1804	0.0081		
lsmc	SNP20	AUX20	0	1	6.8095	0.0099	3	157	108.8215	0.1056	0.0388	0.0553	225
lsmc	SNP21	AUX21	0	1	0.2238	0.6368	3	157	113.3355	0.0685	0.0013		
lsmc	SNP22	AUX22	0	2	6.7756	0.0015	4	154	105.9232	0.1415	0.0755	0.0136	225
lsmc	SNP23	AUX23	0	2	0.2199	0.8029	4	154	114.841	0.0693	0.0027		
lsmc	SNP222	cp1	0	1	0.1062	0.7449	3	208	81.8946	0.4192	2.96E-04		
lsmc	SNP231	cp10	0	1	1.1133	0.2925	3	219	83.6377	0.3876	0.0031		
lsmc	SNP232	cp11	0	1	0.0106	0.918	3	219	84.5826	0.3807	3.00E-05		
lsmc	SNP233	cp12	0	1	0.226	0.635	3	216	86.8364	0.3624	6.67E-04		
lsmc	SNP234	cp13	0	1	0.7424	0.3899	3	195	96.6228	0.3075	0.0026		
lsmc	SNP235	cp14	0	1	0.0412	0.8393	3	211	78.4211	0.4211	1.13E-04		
lsmc	SNP223	cp2	0	1	0.1681	0.6822	3	217	83.432	0.3912	4.72E-04		
lsmc	SNP224	cp3	0	1	0.9919	0.3204	3	219	83.1046	0.3915	0.0028		
lsmc	SNP225	cp4	0	1	0.1679	0.6824	3	219	83.5681	0.3881	4.69E-04		
lsmc	SNP226	cp5	0	2	0.0115	0.9886	4	218	84.6768	0.3828	6.52E-05		
lsmc	SNP227	cp6	0	1	0.0041	0.9491	3	219	84.4649	0.3815	1.15E-05		
lsmc	SNP228	cp7	0	1	0.1616	0.6881	3	219	83.6236	0.3877	4.52E-04		
lsmc	SNP229	cp8	0	1	0.0121	0.9126	3	218	84.1115	0.3854	3.40E-05		
lsmc	SNP230	cp9	0	1	1.1133	0.2925	3	219	83.636	0.3876	0.0031		
lsmc	SNP236	Fe1	0	1	2.2682	0.1337	3	191	92.2474	0.306	0.0082		
lsmc	SNP245	Fe10	0	1	3.4397	0.0654	3	166	76.832	0.3401	0.0137		
lsmc	SNP246	Fe11	0	1	1.8865	0.1715	3	166	76.2764	0.3449	0.0074		
lsmc	SNP237	Fe2	0	1	1.139	0.2871	3	204	90.109	0.3143	0.0038		
lsmc	SNP238	Fe3	0	1	1.0049	0.3173	3	204	90.0272	0.3149	0.0034		
lsmc	SNP239	Fe4	0	1	1.0407	0.3089	3	205	89.4386	0.3166	0.0035		
lsmc	SNP240	Fe5	0	1	2.8228	0.0945	3	202	88.6093	0.3261	0.0094		
lsmc	SNP241	Fe6	0	1	2.0606	0.1527	3	201	83.9844	0.3601	0.0066		
lsmc	SNP242	Fe7	0	1	0.8952	0.3453	3	192	84.3968	0.3131	0.0032		
lsmc	SNP243	Fe8	0	1	0.7038	0.4026	3	180	67.4847	0.4319	0.0022		

lsmc	SNP244	Fe9	0	1	1.3734	0.2428	3	178	63.15	0.4707	0.0041		
lsmc	SNP247	Fum1	0	1	0.9772	0.324	3	219	78.6629	0.4201	0.0026		
lsmc	SNP248	Fum2	0	2	0.3961	0.6734	4	233	81.0705	0.3905	0.0021		
lsmc	SNP249	Fum3	0	1	0.023	0.8797	3	235	83.4248	0.3806	6.05E-05		
lsmc	SNP250	Fum4	0	1	0.6354	0.4262	3	235	84.3552	0.3737	0.0017		
lsmc	SNP251	Fum5	0	1	0.0024	0.9608	3	233	83.3865	0.3818	6.42E-06		
lsmc	SNP252	Fum6	0	1	0.098	0.7546	3	233	83.7448	0.3792	2.61E-04		
lsmc	SNP253	Fum7	0	1	0.1504	0.6985	3	233	83.3671	0.382	3.99E-04		
lsmc	SNP254	Fum8	0	1	0.0105	0.9184	3	233	83.3495	0.3821	2.79E-05		
lsmc	SNP255	Ger1	0	1	3.1034	0.0801	3	153	74.8094	0.4706	0.0107		
lsmc	SNP264	Ger10	0	1	4.0834	0.045	3	156	72.2793	0.4831	0.0135		
lsmc	SNP265	Ger11	0	1	4.0834	0.045	3	156	72.2775	0.4831	0.0135		
lsmc	SNP266	Ger12	0	1	4.0835	0.045	3	156	72.2757	0.4831	0.0135		
lsmc	SNP267	Ger13	0	1	4.0835	0.045	3	156	72.2741	0.4831	0.0135		
lsmc	SNP268	Ger14	0	1	3.9876	0.0476	3	157	72.7932	0.4768	0.0133		
lsmc	SNP269	Ger15	0	1	3.9876	0.0476	3	157	72.7949	0.4768	0.0133		
lsmc	SNP270	Ger16	0	1	1.513	0.2205	3	157	81.1623	0.4166	0.0056		
lsmc	SNP271	Ger17	0	1	1.743	0.1887	3	157	81.6864	0.4128	0.0065		
lsmc	SNP272	Ger18	0	1	3.9849	0.0476	3	157	72.8679	0.4762	0.0133		
lsmc	SNP273	Ger19	0	1	3.8341	0.052	3	156	74.3353	0.4669	0.0131		
lsmc	SNP256	Ger2	0	1	0.0054	0.9415	3	154	82.3128	0.4162	2.05E-05		
lsmc	SNP274	Ger20	0	1	1.12E-04	0.9916	3	156	84.9308	0.3909	4.37E-07		
lsmc	SNP275	Ger21	0	1	3.9848	0.0476	3	157	72.8701	0.4762	0.0133		
lsmc	SNP276	Ger22	0	1	3.9848	0.0476	3	157	72.8684	0.4762	0.0133		
lsmc	SNP277	Ger23	0	1	3.9849	0.0476	3	157	72.8668	0.4762	0.0133		
lsmc	SNP278	Ger24	0	1	1.513	0.2205	3	157	81.1628	0.4166	0.0056		
lsmc	SNP279	Ger25	0	1	3.3773	0.068	3	155	68.7555	0.5065	0.0108		
lsmc	SNP280	Ger26	0	2	2.0782	0.1289	4	142	72.792	0.4972	0.0147		
lsmc	SNP257	Ger3	0	1	4.0126	0.0469	3	154	71.8028	0.4908	0.0133		
lsmc	SNP258	Ger4	0	1	1.6629	0.1992	3	154	79.895	0.4334	0.0061		
lsmc	SNP259	Ger5	0	1	0.0019	0.9654	3	155	80.6664	0.4249	6.99E-06		
lsmc	SNP260	Ger6	0	1	0.0037	0.9518	3	155	81.0422	0.4222	1.37E-05		
lsmc	SNP261	Ger7	0	1	4.0619	0.0456	3	155	70.4525	0.4977	0.0132		
lsmc	SNP262	Ger8	0	1	4.0619	0.0456	3	155	70.4501	0.4977	0.0132		
lsmc	SNP263	Ger9	0	1	7.53E-04	0.9781	3	156	82.7401	0.4083	2.86E-06		
lsmc	SNP281	ICD1	0	1	0.0899	0.7646	3	209	96.8471	0.2622	3.17E-04		
lsmc	SNP290	ICD10	0	1	0.3381	0.5615	3	232	87.111	0.3575	9.36E-04		
lsmc	SNP291	ICD11	0	1	0.2905	0.5904	3	232	87.0695	0.3578	8.04E-04		
lsmc	SNP292	ICD12	0	1	0.1756	0.6756	3	230	88.5296	0.3501	4.96E-04		
lsmc	SNP293	ICD13	0	1	2.9592	0.0867	3	231	90.1291	0.3356	0.0085		
lsmc	SNP294	ICD14	0	1	0.9212	0.3382	3	224	88.8704	0.3476	0.0027		
lsmc	SNP282	ICD2	0	2	0.2439	0.7838	4	216	91.3921	0.3234	0.0015		
lsmc	SNP283	ICD3	0	1	0.3674	0.5451	3	217	90.8851	0.324	0.0011		
lsmc	SNP284	ICD4	0	1	0.0057	0.9397	3	217	90.2018	0.3317	1.77E-05		
lsmc	SNP285	ICD5	0	1	0.2143	0.6438	3	217	90.2999	0.331	6.61E-04		
lsmc	SNP286	ICD6	0	2	0.9793	0.3773	4	206	75.4469	0.4239	0.0055		
lsmc	SNP287	ICD7	0	1	0.2526	0.6158	3	207	79.7248	0.3883	7.47E-04		
lsmc	SNP288	ICD8	0	1	0.2884	0.5917	3	232	85.8235	0.367	7.87E-04		
lsmc	SNP289	ICD9	0	1	0.2884	0.5917	3	232	85.825	0.367	7.87E-04		
lsmc	SNP295	ISL1	0	1	0.249	0.6183	3	195	94.391	0.3145	8.75E-04		
lsmc	SNP296	ISL2	0	1	0.8474	0.3584	3	195	91.7974	0.3334	0.0029		
lsmc	SNP297	ISL3	0	1	3.8024	0.0526	3	193	87.5283	0.3683	0.0124		
lsmc	SNP298	ISL4	0	1	9.624	0.0022	3	191	92.0578	0.3338	0.0336	0.0137	225
lsmc	SNP299	ISL5	0	1	2.2891	0.132	3	185	86.9297	0.3849	0.0076		
lsmc	SNP300	ISL6	0	1	0.4621	0.4975	3	184	96.3468	0.3146	0.0017		
lsmc	SNP301	ME1	0	1	0.2577	0.6122	3	220	81.4329	0.3888	7.16E-04		
lsmc	SNP310	ME10	0	1	0.041	0.8397	3	211	80.7026	0.3955	1.18E-04		
lsmc	SNP311	ME11	0	1	0.7528	0.3866	3	210	78.8999	0.4111	0.0021		
lsmc	SNP312	ME12	0	3	0.7943	0.4983	5	207	76.6887	0.4356	0.0065		
lsmc	SNP302	ME2	0	1	0.4051	0.5251	3	220	82.8008	0.3785	0.0011		
lsmc	SNP303	ME3	0	1	0.0472	0.8281	3	220	83.7488	0.3714	1.35E-04		
lsmc	SNP304	ME4	0	1	0.3604	0.5489	3	220	83.0196	0.3769	0.001		
lsmc	SNP305	ME5	0	1	1.6753	0.1969	3	220	83.1357	0.376	0.0048		
lsmc	SNP306	ME6	0	3	1.2785	0.2826	5	218	83.6662	0.3777	0.0109		
lsmc	SNP307	ME7	0	1	0.1124	0.7378	3	217	82.0005	0.3905	3.16E-04		
lsmc	SNP308	ME8	0	1	0.0857	0.77	3	217	82.347	0.3879	2.42E-04		
lsmc	SNP309	ME9	0	1	0.0468	0.829	3	211	80.5389	0.3968	1.34E-04		
lsmc	SNP313	P13K1	0	1	1.5486	0.2146	3	223	91.8344	0.3329	0.0046		
lsmc	SNP322	P13K10	0	1	0.0023	0.962	3	185	67.8236	0.5151	5.96E-06		
lsmc	SNP314	P13K2	0	1	0.1042	0.7472	3	223	88.9716	0.3537	3.02E-04		
lsmc	SNP315	P13K3	0	1	0.0394	0.8428	3	223	89.2051	0.352	1.15E-04		
lsmc	SNP316	P13K4	0	1	1.067	0.3027	3	223	90.3982	0.3433	0.0031		
lsmc	SNP317	P13K5	0	1	0.2488	0.6184	3	223	88.9368	0.3539	7.21E-04		
lsmc	SNP318	P13K6	0	1	0.7577	0.385	3	223	88.3666	0.3581	0.0022		
lsmc	SNP319	P13K7	0	1	0.0609	0.8054	3	223	89.3954	0.3506	1.77E-04		
lsmc	SNP320	P13K8	0	1	0.7425	0.3898	3	221	89.2851	0.3572	0.0022		
lsmc	SNP321	P13K9	0	1	0.7457	0.3888	3	221	91.371	0.3422	0.0022		
lsmc	SNP323	PME1	0	1	0.7824	0.3773	3	236	83.9828	0.377	0.0021		
lsmc	SNP324	PME2	0	1	1.5861	0.2091	3	236	87.0263	0.3544	0.0043		
lsmc	SNP325	PME3	0	1	1.688	0.1952	3	232	88.2092	0.3459	0.0048		
lsmc	SNP326	SAH1	0	1	0.0421	0.8377	3	176	100.5551	0.2473	1.80E-04		
lsmc	SNP327	SAH2	0	1	0.0889	0.7659	3	176	101.483	0.2403	3.84E-04		
lsmc	SNP328	SAH3	0	1	0.0081	0.9283	3	176	101.2837	0.2418	3.50E-05		
lsmc	SNP329	SAH4	0	1	0.1435	0.7053	3	176	100.5072	0.2476	6.13E-04		
lsmc	SNP330	SAH5	0	2	0.4934	0.6114	4	171	102.1021	0.2259	0.0045		
lsmc	SNP331	SAH6	0	1	0.0546	0.8157	3	113	99.4956	0.2066	3.83E-04		
