# Supplementary material for: Association and Linkage Analysis of Aluminum Tolerance Genes in Maize
Source: PLoS One. 2010 Apr 1;5(4):e9958. doi: 10.1371/journal.pone.0009958 (PMC2848604; doi:10.1371/journal.pone.0009958)
Supplement: Table S4 — Validation of association mapping via linkage mapping. Association mapping results were validated using linkage mapping of F2 populations segregating for the candidate Al tolerance genes. This table reports phenotypic and genotypic information for the linkage experiments. (0.05 MB PDF) [file pone.0009958.s004.pdf]

FAMILY	INDIVIDUAL	IRG	FRG	NRG	PME	ME	ISL	ALMT	SAHH	ZmASL
B73xCML247	B73xCML247_003	81	161	80					H	
B73xCML247	B73xCML247_004	54	107	53					H	
B73xCML247	B73xCML247_005	61	126	65					B	A
B73xCML247	B73xCML247_007	82	163	81					A	H
B73xCML247	B73xCML247_008	74	143	69					A	H
B73xCML247	B73xCML247_009	104	194	90					A	H
B73xCML247	B73xCML247_010	57	106	49					H	H
B73xCML247	B73xCML247_011	52	108	56					B	A
B73xCML247	B73xCML247_013	97	193	96					A	H
B73xCML247	B73xCML247_014	62	117	55					B	A
B73xCML247	B73xCML247_015	98	172	74					A	
B73xCML247	B73xCML247_016	94	142	48					A	H
B73xCML247	B73xCML247_017	65	109	44					B	B
B73xCML247	B73xCML247_018	75	127	52					B	B
B73xCML247	B73xCML247_019	77	172	95					A	H
B73xCML247	B73xCML247_020	87	172	85					B	B
B73xCML247	B73xCML247_021	70	107	37					H	
B73xCML247	B73xCML247_024	75	158	83						A
B73xCML247	B73xCML247_025	85	161	76					H	
B73xCML247	B73xCML247_026	76	139	63					A	H
B73xCML247	B73xCML247_027	63	116	53					A	
B73xCML247	B73xCML247_029	87	146	59					B	H
B73xCML247	B73xCML247_030	85	149	64					B	H
B73xCML247	B73xCML247_033	67	122	55					B	A
B73xCML247	B73xCML247_035	59	117	58					B	H
B73xCML247	B73xCML247_036	61	122	61					H	H
B73xCML247	B73xCML247_037	74	140	66					H	A
B73xCML247	B73xCML247_038	60	142	82					H	
B73xCML247	B73xCML247_039	103	198	95					H	A
B73xCML247	B73xCML247_040	70	122	52					H	B
B73xCML247	B73xCML247_041	66	122	56					A	H
B73xCML247	B73xCML247_042	68	134	66					H	A
B73xCML247	B73xCML247_043	98	188	90					A	
B73xCML247	B73xCML247_044	56	127	71					A	H
B73xCML247	B73xCML247_045	52	120	68					H	H
B73xCML247	B73xCML247_046	74	129	55					B	
B73xCML247	B73xCML247_047	72	155	83					A	A
B73xCML247	B73xCML247_048	89	171	82						B
B73xCML247	B73xCML247_049	73	152	79					A	H
B73xCML247	B73xCML247_051	85	153	68					A	B
B73xCML247	B73xCML247_052	61	153	92					A	A
B73xCML247	B73xCML247_054	65	117	52					A	
B73xCML247	B73xCML247_055	65	151	86					A	
B73xCML247	B73xCML247_056	72	146	74					A	A
B73xCML247	B73xCML247_057	68	157	89					A	
B73xCML247	B73xCML247_058	76	145	69					B	H
B73xCML247	B73xCML247_059	47	95	48					B	
B73xCML247	B73xCML247_061	54	135	81						A
B73xCML247	B73xCML247_062	66	134	68					A	H
B73xCML247	B73xCML247_063	66	136	70					A	B
B73xCML247	B73xCML247_064	58	102	44					B	H
B73xCML247	B73xCML247_065	64	97	33					H	B

B73xCML247	B73xCML247_066	71	136	65	H	
B73xCML247	B73xCML247_067	68	132	64	B	H
B73xCML247	B73xCML247_068	57	119	62	H	A
B73xCML247	B73xCML247_069	62	112	50	B	B
B73xCML247	B73xCML247_070	90	170	80	H	B
B73xCML247	B73xCML247_072	91	160	69	B	
B73xCML247	B73xCML247_073	70	135	65	B	A
B73xCML247	B73xCML247_074	75	147	72	B	H
B73xCML247	B73xCML247_075	98	150	52	B	H
B73xCML247	B73xCML247_076	75	128	53	H	A
B73xCML247	B73xCML247_077	58	99	41	H	B
B73xCML247	B73xCML247_078	61	127	66	H	
B73xCML247	B73xCML247_079	62	95	33	H	H
B73xCML247	B73xCML247_080	69	141	72	H	B
B73xCML247	B73xCML247_081	82	138	56	H	B
B73xCML247	B73xCML247_082	80	172	92	B	H
B73xCML247	B73xCML247_083	54	130	76	H	H
B73xCML247	B73xCML247_084	95	177	82	H	
B73xCML247	B73xCML247_085	106	173	67	B	H
B73xCML247	B73xCML247_089	90	145	55	H	
B73xCML247	B73xCML247_091	75	164	89		A
B73xCML247	B73xCML247_092	64	131	67		B
B73xCML247	B73xCML247_093	73	154	81	H	
B73xCML247	B73xCML247_094	63	133	70		A
B73xCML247	B73xCML247_095	64	129	65	A	
B73xCML247	B73xCML247_096	67	145	78	H	
B73xCML247	B73xCML247_097	86	162	76		H
B73xCML247	B73xCML247_098	53	103	50	H	B
B73xCML247	B73xCML247_100	84	170	86	B	H
B73xCML247	B73xCML247_101	91	191	100		H
B73xCML247	B73xCML247_102	48	104	56	H	B
B73xCML247	B73xCML247_103	67	107	40		H
B73xCML247	B73xCML247_108	54	116	62		A
B73xCML247	B73xCML247_109	84	165	81		H
B73xCML247	B73xCML247_111	49	100	51	A	
B73xCML247	B73xCML247_112	65	129	64	H	A
B73xCML247	B73xCML247_115	69	151	82	A	B
B73xCML247	B73xCML247_116	78	156	78	H	B
B73xCML247	B73xCML247_117	59	119	60	H	B
B73xCML247	B73xCML247_118	56	117	61		A
B73xCML247	B73xCML247_120	69	164	95		B
B73xCML247	B73xCML247_121	88	158	70	H	H
B73xCML247	B73xCML247_122	62	128	66	H	B
B73xCML247	B73xCML247_123	51	115	64	B	B
B73xCML247	B73xCML247_124	58	106	48	B	A
B73xCML247	B73xCML247_125	63	111	48	H	B
B73xCML247	B73xCML247_126	73	137	64	H	H
B73xCML247	B73xCML247_127	73	148	75	B	H
B73xCML247	B73xCML247_129	30	70	40	A	B
B73xCML247	B73xCML247_130	77	160	83	H	H
B73xCML247	B73xCML247_132	66	125	59	H	H
B73xCML247	B73xCML247_133	81	163	82	B	H
B73xCML247	B73xCML247_135	66	155	89	H	H

B73xCML247	B73xCML247_136	65	149	84			B	H
B73xCML247	B73xCML247_138	69	150	81			H	B
B73xCML247	B73xCML247_144	70	157	87			H	A
B73xCML247	B73xCML247_145	76	147	71			B	B
B73xCML247	B73xCML247_146	58	97	39				H
B73xCML247	B73xCML247_147	76	170	94				A
B73xCML247	B73xCML247_148	58	119	61			H	B
B73xCML247	B73xCML247_149	68	115	47			H	A
B73xCML247	B73xCML247_150	71	151	80			H	H
B73xCML247	B73xCML247_151	83	165	82			H	A
B73xCML247	B73xCML247_152	100	170	70			B	A
B73xCML247	B73xCML247_153	57	137	80			B	A
B73xCML247	B73xCML247_154	83	173	90			H	H
B73xCML247	B73xCML247_156	84	158	74			A	H
B73xCML247	B73xCML247_157	68	135	67			H	H
B73xCML247	B73xCML247_158	65	153	88			A	H
B73xCML247	B73xCML247_159	83	143	60			H	B
B73xCML247	B73xCML247_161	73	132	59			H	H
B73xCML247	B73xCML247_162	81	162	81			H	H
B73xCML247	B73xCML247_163	73	119	46			A	H
B73xCML247	B73xCML247_164	89	181	92			H	H
B73xCML247	B73xCML247_165	45	96	51			H	B
B73xCML247	B73xCML247_166	55	107	52			H	A
B73xCML247	B73xCML247_168	71	160	89			A	A
B73xCML247	B73xCML247_170	60	115	55			B	H
B73xCML247	B73xCML247_171	54	118	64			B	A
B73xCML247	B73xCML247_172	62	148	86			H	A
B73xCML247	B73xCML247_173	70	162	92			A	H
B73xCML247	B73xCML247_174	75	150	75			B	H
B73xCML247	B73xCML247_175	53	103	50			H	B
B73xCML247	B73xCML247_176	83	131	48			B	B
B73xCML247	B73xCML247_177	102	147	45			H	B
B73xCML247	B73xCML247_178	70	142	72			A	A
B73xCML247	B73xCML247_180	79	161	82				A
B73xCML247	B73xCML247_181	71	127	56			H	B
B73xCML247	B73xCML247_182	80	131	51			H	H
B73xCML247	B73xCML247_183	94	171	77			H	H
B73xCML247	B73xCML247_185	59	94	35			B	A
B73xCML247	B73xCML247_186	79	157	78			A	H
B73xCML247	B73xCML247_187	70	125	55			H	A
B73xCML247	B73xCML247_188	75	139	64			B	H
B73xCML247	B73xCML247_189	85	167	82			B	B
B73xCML247	B73xCML247_190	67	111	44				B
B73xCML333	B73xCML333_339	40	175	135	A			
B73xCML333	B73xCML333_340	99	165	66	A	H		
B73xCML333	B73xCML333_341	128	190	62	B			
B73xCML333	B73xCML333_342	99	144	45	H			
B73xCML333	B73xCML333_343	108	180	72	A			
B73xCML333	B73xCML333_344	101	181	80	A	H		
B73xCML333	B73xCML333_345	81	129	48	B			
B73xCML333	B73xCML333_346	91	159	68	B			
B73xCML333	B73xCML333_347	71	144	73	H			
B73xCML333	B73xCML333_349	68	115	47	A			

B73xCML333	B73xCML333_350	49	85	36	B	H
B73xCML333	B73xCML333_351	57	85	28	B	H
B73xCML333	B73xCML333_354	50	130	80	A	
B73xCML333	B73xCML333_355	88	117	29	A	B
B73xCML333	B73xCML333_356	78	109	31	B	H
B73xCML333	B73xCML333_359	78	127	49	A	
B73xCML333	B73xCML333_360	54	95	41	H	H
B73xCML333	B73xCML333_361	79	140	61	H	
B73xCML333	B73xCML333_362	68	135	67	B	H
B73xCML333	B73xCML333_363	102	153	51	A	H
B73xCML333	B73xCML333_364	88	150	62	B	A
B73xCML333	B73xCML333_365	76	147	71	A	A
B73xCML333	B73xCML333_366	65	139	74	A	B
B73xCML333	B73xCML333_367	98	137	39	H	
B73xCML333	B73xCML333_368	85	165	80	B	
B73xCML333	B73xCML333_369	105	143	38	H	A
B73xCML333	B73xCML333_370	51	95	44	H	H
B73xCML333	B73xCML333_371	74	139	65	H	A
B73xCML333	B73xCML333_372	87	107	20	H	
B73xCML333	B73xCML333_373	86	139	53	H	
B73xCML333	B73xCML333_374	68	128	60	H	H
B73xCML333	B73xCML333_375	74	128	54	H	B
B73xCML333	B73xCML333_376	98	143	45	A	
B73xCML333	B73xCML333_377	91	127	36	H	
B73xCML333	B73xCML333_378	92	124	32	H	H
B73xCML333	B73xCML333_379	86	172	86	A	H
B73xCML333	B73xCML333_380	56	146	90	A	H
B73xCML333	B73xCML333_381	93	147	54	A	B
B73xCML333	B73xCML333_382	99	149	50	H	
B73xCML333	B73xCML333_383	68	105	37	H	
B73xCML333	B73xCML333_384	79	178	99	H	A
B73xCML333	B73xCML333_385	78	162	84	A	A
B73xCML333	B73xCML333_386	71	161	90	A	B
B73xCML333	B73xCML333_387	78	149	71	B	B
B73xCML333	B73xCML333_388	74	142	68	B	H
B73xCML333	B73xCML333_389	93	147	54	H	A
B73xCML333	B73xCML333_390	77	122	45	A	A
B73xCML333	B73xCML333_391	80	127	47	H	A
B73xCML333	B73xCML333_392	100	150	50	B	H
B73xCML333	B73xCML333_393	62	173	111	H	H
B73xCML333	B73xCML333_394	43	129	86	H	H
B73xCML333	B73xCML333_395	75	159	84	B	H
B73xCML333	B73xCML333_396	57	148	91	A	
B73xCML333	B73xCML333_397	106	130	24	B	H
B73xCML333	B73xCML333_398	50	124	74	H	H
B73xCML333	B73xCML333_399	105	158	53	B	
B73xCML333	B73xCML333_400	95	136	41	A	B
B73xCML333	B73xCML333_401	62	93	31		H
B73xCML333	B73xCML333_402	81	135	54	B	H
B73xCML333	B73xCML333_403	90	145	55	A	H
B73xCML333	B73xCML333_404	91	140	49	A	A
B73xCML333	B73xCML333_405	85	132	47	A	
B73xCML333	B73xCML333_406	86	115	29	B	A

B73xCML333	B73xCML333_407	59	151	92	H	A
B73xCML333	B73xCML333_408	114	131	17	H	A
B73xCML333	B73xCML333_409	82	159	77	H	
B73xCML333	B73xCML333_410	84	119	35	A	
B73xCML333	B73xCML333_411	108	140	32	H	B
B73xCML333	B73xCML333_412	85	158	73	H	H
B73xCML333	B73xCML333_413	39	157	118	H	H
B73xCML333	B73xCML333_414	79	170	91	A	
B73xCML333	B73xCML333_415	77	125	48	A	A
B73xCML333	B73xCML333_416	88	148	60	B	B
B73xCML333	B73xCML333_417	103	171	68	A	
B73xCML333	B73xCML333_418	108	168	60	B	
B73xCML333	B73xCML333_420	109	200	91	H	H
B73xCML333	B73xCML333_421	68	156	88	A	
B73xCML333	B73xCML333_422	97	154	57	B	
B73xCML333	B73xCML333_423	95	180	85	B	B
B73xCML333	B73xCML333_424	70	130	60	A	
B73xCML333	B73xCML333_425	72	123	51	H	A
B73xCML333	B73xCML333_426	102	183	81	H	A
B73xCML333	B73xCML333_427	70	145	75	A	A
B73xCML333	B73xCML333_428	93	152	59	A	
B73xCML333	B73xCML333_429	82	153	71	H	A
B73xCML333	B73xCML333_430	73	94	21	B	H
B73xCML333	B73xCML333_432	62	105	43	H	
B73xCML333	B73xCML333_433	84	150	66	H	A
B73xCML333	B73xCML333_434	66	137	71	H	
B73xCML333	B73xCML333_435	83	144	61	A	A
B73xCML333	B73xCML333_436	91	133	42	H	
B73xCML333	B73xCML333_437	97	167	70	A	
B73xCML333	B73xCML333_438	71	138	67	H	H
B73xCML333	B73xCML333_440	78	142	64	H	H
B73xCML333	B73xCML333_441	81	156	75	A	
B73xCML333	B73xCML333_442	74	102	28	B	
B73xCML333	B73xCML333_443	120	198	78	H	
B73xCML333	B73xCML333_444	82	139	57	A	
B73xCML333	B73xCML333_445	77	134	57	A	
B73xCML333	B73xCML333_446	109	165	56	H	A
B73xCML333	B73xCML333_447	114	204	90	A	
B73xCML333	B73xCML333_448	61	102	41	B	B
B73xCML333	B73xCML333_449	104	163	59	B	H
B73xCML333	B73xCML333_450	92	155	63		B
B73xCML333	B73xCML333_455	35	90	55	H	
B73xCML333	B73xCML333_456	73	153	80	H	A
B73xCML333	B73xCML333_458	68	125	57	A	B
B73xCML333	B73xCML333_459	117	171	54	A	
B73xCML333	B73xCML333_460	92	139	47	H	B
B73xCML333	B73xCML333_461	82	145	63	H	
B73xCML333	B73xCML333_463	52	110	58	H	A
B73xCML333	B73xCML333_464	74	129	55	H	H
B73xCML333	B73xCML333_465	57	121	64	A	B
B73xCML333	B73xCML333_467	71	127	56	A	
B73xCML333	B73xCML333_468	73	140	67	B	B
B73xCML333	B73xCML333_469	89	128	39	B	B

B73xCML333	B73xCML333_470	98	143	45	H	A	
B73xCML333	B73xCML333_471	89	138	49	B		
B73xCML333	B73xCML333_472	93	135	42	H		
B73xCML333	B73xCML333_473	85	149	64	B		
B73xCML333	B73xCML333_474	84	142	58	H		
B73xCML333	B73xCML333_475	79	110	31	H	H	
B73xCML333	B73xCML333_476	93	155	62	B		
B73xCML333	B73xCML333_477	84	105	21	H		
B73xCML333	B73xCML333_478	98	179	81	A		
B73xCML333	B73xCML333_479	94	153	59		H	
B73xCML333	B73xCML333_480	65	102	37	H	B	
B73xCML333	B73xCML333_481	72	109	37	A	A	
B73xCML333	B73xCML333_482	76	133	57	A		
B73xCML333	B73xCML333_483	84	112	28	H		
B73xCML333	B73xCML333_484	79	130	51	H		
B73xCML333	B73xCML333_486	57	111	54	A		
B73xCML333	B73xCML333_489	96	152	56	H		
B73xNC350	B73xNC350_001	65	105	40			A
B73xNC350	B73xNC350_002	58	102	44			B
B73xNC350	B73xNC350_005	63	125	62			A
B73xNC350	B73xNC350_006	100	145	45			H
B73xNC350	B73xNC350_007	70	125	55			B
B73xNC350	B73xNC350_008	66	114	48			H
B73xNC350	B73xNC350_009	79	113	34			H
B73xNC350	B73xNC350_010	68	126	58			B
B73xNC350	B73xNC350_011	80	121	41			H
B73xNC350	B73xNC350_012	70	120	50			H
B73xNC350	B73xNC350_013	75	119	44			A
B73xNC350	B73xNC350_014	70	119	49			H
B73xNC350	B73xNC350_015	75	120	45			A
B73xNC350	B73xNC350_016	82	145	63			H
B73xNC350	B73xNC350_017	67	125	58			B
B73xNC350	B73xNC350_018	66	115	49			A
B73xNC350	B73xNC350_019	65	114	49			H
B73xNC350	B73xNC350_020	60	106	46			H
B73xNC350	B73xNC350_021	77	133	56			H
B73xNC350	B73xNC350_025	65	114	49			H
B73xNC350	B73xNC350_026	67	115	48			H
B73xNC350	B73xNC350_030	42	92	50			B
B73xNC350	B73xNC350_031	68	95	27			H
B73xNC350	B73xNC350_032	72	128	56			H
B73xNC350	B73xNC350_033	46	82	36			H
B73xNC350	B73xNC350_034	54	102	48			A
B73xNC350	B73xNC350_036	76	121	45			B
B73xNC350	B73xNC350_037	70	106	36			H
B73xNC350	B73xNC350_038	67	131	64			H
B73xNC350	B73xNC350_039	50	66	16			H
B73xNC350	B73xNC350_040	55	80	25			H
B73xNC350	B73xNC350_041	87	138	51			A
B73xNC350	B73xNC350_042	74	126	52			H
B73xNC350	B73xNC350_043	66	124	58			H
B73xNC350	B73xNC350_045	67	95	28			A
B73xNC350	B73xNC350_047	68	110	42			A

B73xNC350	B73xNC350_048	67	115	48		B
B73xNC350	B73xNC350_049	54	102	48		B
B73xNC350	B73xNC350_050	76	104	28		H
B73xNC350	B73xNC350_051	66	95	29		A
B73xNC350	B73xNC350_052	66	91	25		B
B73xNC350	B73xNC350_053	72	115	43		H
B73xNC350	B73xNC350_055	78	109	31		B
B73xNC350	B73xNC350_056	50	82	32		B
B73xNC350	B73xNC350_057	60	115	55		A
B73xNC350	B73xNC350_058	60	92	32		H
B73xNC350	B73xNC350_059	67	106	39		A
B73xNC350	B73xNC350_061	75	122	47		A
B73xNC350	B73xNC350_062	60	108	48		H
B73xNC350	B73xNC350_063	62	100	38		H
B73xNC350	B73xNC350_064	59	86	27		B
B73xNC350	B73xNC350_066	75	135	60		B
B73xNC350	B73xNC350_067	90	112	22		B
B73xNC350	B73xNC350_068	68	112	44		A
B73xNC350	B73xNC350_069	80	132	52		H
B73xNC350	B73xNC350_070	77	120	43		A
B73xNC350	B73xNC350_071	76	121	45		H
B73xNC350	B73xNC350_072	76	119	43		B
B73xNC350	B73xNC350_073	60	95	35		H
B73xNC350	B73xNC350_074	82	120	38		B
B73xNC350	B73xNC350_075	82	105	23		B
B73xNC350	B73xNC350_076	51	81	30		B
B73xNC350	B73xNC350_077	78	105	27		B
B73xNC350	B73xNC350_078	56	112	56		H
B73xNC350	B73xNC350_079	45	85	40		A
B73xNC350	B73xNC350_080	60	100	40		A
B73xNC350	B73xNC350_081	58	102	44		A
B73xNC350	B73xNC350_082	70	125	55		H
B73xNC350	B73xNC350_083	52	95	43		H
B73xNC350	B73xNC350_084	85	125	40		H
B73xNC350	B73xNC350_085	56	82	26		B
B73xNC350	B73xNC350_086	74	115	41		H
B73xNC350	B73xNC350_087	55	100	45		H
B73xNC350	B73xNC350_089	66	125	59		B
B73xNC350	B73xNC350_090	46	97	51		H
B73xNC350	B73xNC350_091	45	82	37		H
B73xNC350	B73xNC350_092	60	96	36		A
B73xNC350	B73xNC350_093	56	105	49		A
B73xNC350	B73xNC350_095	68	85	17		H
B73xNC350	B73xNC350_096	73	135	62		A
B73xNC350	B73xNC350_097	70	113	43		A
B73xNC350	B73xNC350_098	84	125	41		H
B73xNC350	B73xNC350_099	65	128	63		B
B73xNC350	B73xNC350_100	77	106	29		H
B73xNC350	B73xNC350_101	68	134	66		H
B73xNC350	B73xNC350_102	70	125	55	B	H
B73xNC350	B73xNC350_103	50	107	57	H	B
B73xNC350	B73xNC350_104	71	119	48	H	A
B73xNC350	B73xNC350_106	75	122	47	A	H

B73xNC350	B73xNC350_108	76	121	45	B	H
B73xNC350	B73xNC350_109	75	127	52	B	B
B73xNC350	B73xNC350_111	73	113	40	H	A
B73xNC350	B73xNC350_112	64	117	53		A
B73xNC350	B73xNC350_114	71	122	51	A	H
B73xNC350	B73xNC350_115	90	115	25	H	B
B73xNC350	B73xNC350_117	76	142	66	H	A
B73xNC350	B73xNC350_119	68	99	31	H	B
B73xNC350	B73xNC350_120	83	116	33	H	
B73xNC350	B73xNC350_121	65	113	48	H	A
B73xNC350	B73xNC350_122	54	119	65	H	A
B73xNC350	B73xNC350_123	82	120	38	A	B
B73xNC350	B73xNC350_124	65	113	48	A	
B73xNC350	B73xNC350_125	74	115	41	B	H
B73xNC350	B73xNC350_126	69	115	46	A	
B73xNC350	B73xNC350_127	66	167	101	H	A
B73xNC350	B73xNC350_133	74	152	78	H	H
B73xNC350	B73xNC350_134	81	113	32	H	B
B73xNC350	B73xNC350_135	75	120	45	B	B
B73xNC350	B73xNC350_136	35	98	63	B	
B73xNC350	B73xNC350_137	72	110	38	A	B
B73xNC350	B73xNC350_138	90	155	65	H	
B73xNC350	B73xNC350_139	51	122	71	H	
B73xNC350	B73xNC350_140	76	125	49	B	H
B73xNC350	B73xNC350_141	62	105	43	B	
B73xNC350	B73xNC350_142	71	128	57	B	
B73xNC350	B73xNC350_143	56	102	46	H	
B73xNC350	B73xNC350_144	70	113	43	H	A
B73xNC350	B73xNC350_145	80	113	33	H	
B73xNC350	B73xNC350_146	75	125	50	H	
B73xNC350	B73xNC350_147	77	130	53	B	
B73xNC350	B73xNC350_148	74	125	51	H	
B73xNC350	B73xNC350_150	55	115	60	H	
B73xNC350	B73xNC350_152	56	77	21	H	
B73xNC350	B73xNC350_153	85	157	72	B	
B73xNC350	B73xNC350_154	80	123	43	B	
B73xNC350	B73xNC350_155	64	122	58	H	
B73xNC350	B73xNC350_157	87	125	38	H	
B73xNC350	B73xNC350_158	75	115	40	H	
B73xNC350	B73xNC350_159	78	141	63	B	
B73xNC350	B73xNC350_160	60	135	75	H	H
B73xNC350	B73xNC350_161	42	105	63	H	
B73xNC350	B73xNC350_162	42	90	48	A	
B73xNC350	B73xNC350_163	60	105	45	A	
B73xNC350	B73xNC350_164	46	92	46	H	
B73xNC350	B73xNC350_166	52	82	30	B	
B73xNC350	B73xNC350_167	88	130	42	H	A
B73xNC350	B73xNC350_168	57	109	52	H	
B73xNC350	B73xNC350_169	88	160	72	A	H
B73xNC350	B73xNC350_170	69	130	61	H	B
B73xNC350	B73xNC350_172	78	125	47	H	
B73xNC350	B73xNC350_173	70	127	57	H	
B73xNC350	B73xNC350_174	63	120	57	H	H

B73xNC350	B73xNC350_175	76	101	25	A	
B73xNC350	B73xNC350_176	76	120	44	A	
B73xNC350	B73xNC350_178	78	115	37		H
B73xNC350	B73xNC350_179	67	110	43	H	
B73xNC350	B73xNC350_180	70	105	35	H	
B73xNC350	B73xNC350_181	66	128	62	H	H
B73xNC350	B73xNC350_183	71	115	44	B	H
B73xNC350	B73xNC350_184	90	147	57	H	H
B73xNC350	B73xNC350_185	80	115	35	B	H
B73xNC350	B73xNC350_186	63	100	37	H	H
B73xNC350	B73xNC350_188	65	103	38	H	
B73xNC350	B73xNC350_189	63	102	39		A
B73xNC350	B73xNC350_190	64	93	29		B
B73xNC350	B73xNC350_191	32	84	52	H	H
B73xNC350	B73xNC350_192	75	128	53	H	H
B73xNC350	B73xNC350_193	68	120	52	A	B
B73xNC350	B73xNC350_194	56	90	34	H	B
B73xNC350	B73xNC350_195	75	130	55	B	
B73xNC350	B73xNC350_196	78	132	54	B	
B73xNC350	B73xNC350_197	80	132	52		A
B73xNC350	B73xNC350_198	70	135	65	H	B
B73xNC350	B73xNC350_199	10	42	32	A	B
B73xNC350	B73xNC350_200	57	110	53	H	
B73xNC350	B73xNC350_201	71	94	23	A	B
