# Supplementary material for: Association and Linkage Analysis of Aluminum Tolerance Genes in Maize
Source: PLoS One. 2010 Apr 1;5(4):e9958. doi: 10.1371/journal.pone.0009958 (PMC2848604; doi:10.1371/journal.pone.0009958)
Supplement: Table S5 — PCR primers utilized for linkage mapping. (0.02 MB PDF) [file pone.0009958.s005.pdf]

Gene abbreviation	MAGI designation	Gene name	Primer Name	Primer Sequence	Indel used as polymorphism
PME	4.0_158804	Pectin Methylesterase	mzb00202_F	CCGGAAGTCCACATCAGAAT	In 3rd exon
			mzb00202_R	GATGCTTGGATAAAACAAATCG	
ME	3.1_47445	Malic Enzyme	mzb00198_F	ACACACACCACCTTTCCACA	Site #1
			mzb00198_R	ACGGTGACGCGGATTTATAG	
ASL	3.1_41691	ZmASL1	MateF2_F1	GACCCCATTATTCCTGCTGA	Site #47
			MateF2_R1	AAACAGAAAGGATGCCGTTG	
SAHH	4.0_116767	SAH hyrdrolase	ADHCF2_F1	AACCTTGGGTGTGCTACTGG	In 1st intron
			ADHCF2_R1	CTTGGGGAGCACATACACCT	
ISL	4.0_124671	Isocitrate Lyase	mzb00203_F	CGTGCACCGCTGACTATAAA	In 2nd intron
			mzb00204_R	CTTCCTCCTCCATGATCTGC	
AL2	3.1_93496	ZmALMT2	ALMT93496_F1	TCAGACTCACAAGGGTGTGG	Site #11
			ALMT93496_R1	CAGCAAAAGGCTTCTGATACAA	
